# Synthesis of Sterically Encumbered Thiourea *S*‐Oxides through Direct Thiourea Oxidation

**DOI:** 10.1002/chem.202203005

**Published:** 2022-12-01

**Authors:** Serhii Medvedko, Markus Ströbele, J. Philipp Wagner

**Affiliations:** ^1^ Institut für Organische Chemie Eberhard-Karls-Universität Tübingen Auf der Morgenstelle 18 72076 Tübingen Germany; ^2^ Department of Organic Chemistry National Technical University of Ukraine Igor Sikorsky Kyiv Polytechnic Institute Peremohy Ave 37 03056 Kyiv Ukraine; ^3^ Institut für Anorganische Chemie Eberhard-Karls-Universität Tübingen Auf der Morgenstelle 18 72076 Tübingen Germany

**Keywords:** DFT calculations, oxidation, reactive intermediates, sulfines, thioureas

## Abstract

Thiourea *S*‐oxides can be viewed as formal analogs of the currently unknown diamino‐substituted Criegee intermediates (urea *O*‐oxides). However, the preparation of such *S*‐oxides is rather challenging, and the direct oxidation of thioureas typically only leads to formation of desulfurized products. Employing the accurate revDSD‐PBEP86‐D4 double hybrid density functional, it was found that the peracid mediated oxidation of thiourea *S*‐oxides exhibits a lower reaction barrier than the oxidation of the corresponding thiourea itself in contrast to most other ordinary thioketones. The undesired overoxidation reactivity, which is associated with strong π‐donation from the thiourea's nitrogen atoms, can be partially suppressed by introduction of bulky substituents and the utilization of protic solvents. In this regard, we managed to prepare two sterically encumbered thiourea *S*‐oxides in isolated yields of 35–40 %. The *S*‐oxides are stable in the solid state and in alcoholic solutions at room temperature for extended periods of time, but swiftly decompose in aprotic solvents by disproportionation. A dimesityl‐substituted thiourea *S*‐oxide complexed with residual *m*CBA could be characterized by means of X‐ray crystallography, confirming the importance of hydrogen bonding in the stabilization of the amino‐substituted C=S^+^−O^−^ moiety.

## Graphical abstract

**Bulking up**: Thiourea *S*‐oxides are usually short‐lived reactive intermediates in the oxidation of thioureas and can be considered as heavier homologues of the elusive N‐heterocyclic Criegee intermediates. Preparation and isolation was possible by introduction of bulky substituents on the nitrogen atoms.

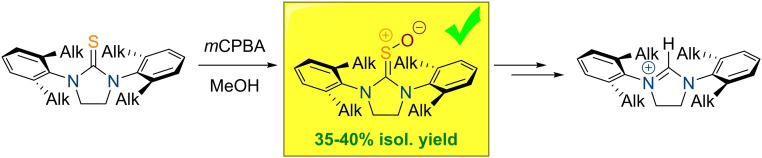

## Introduction

**Figure 1:**
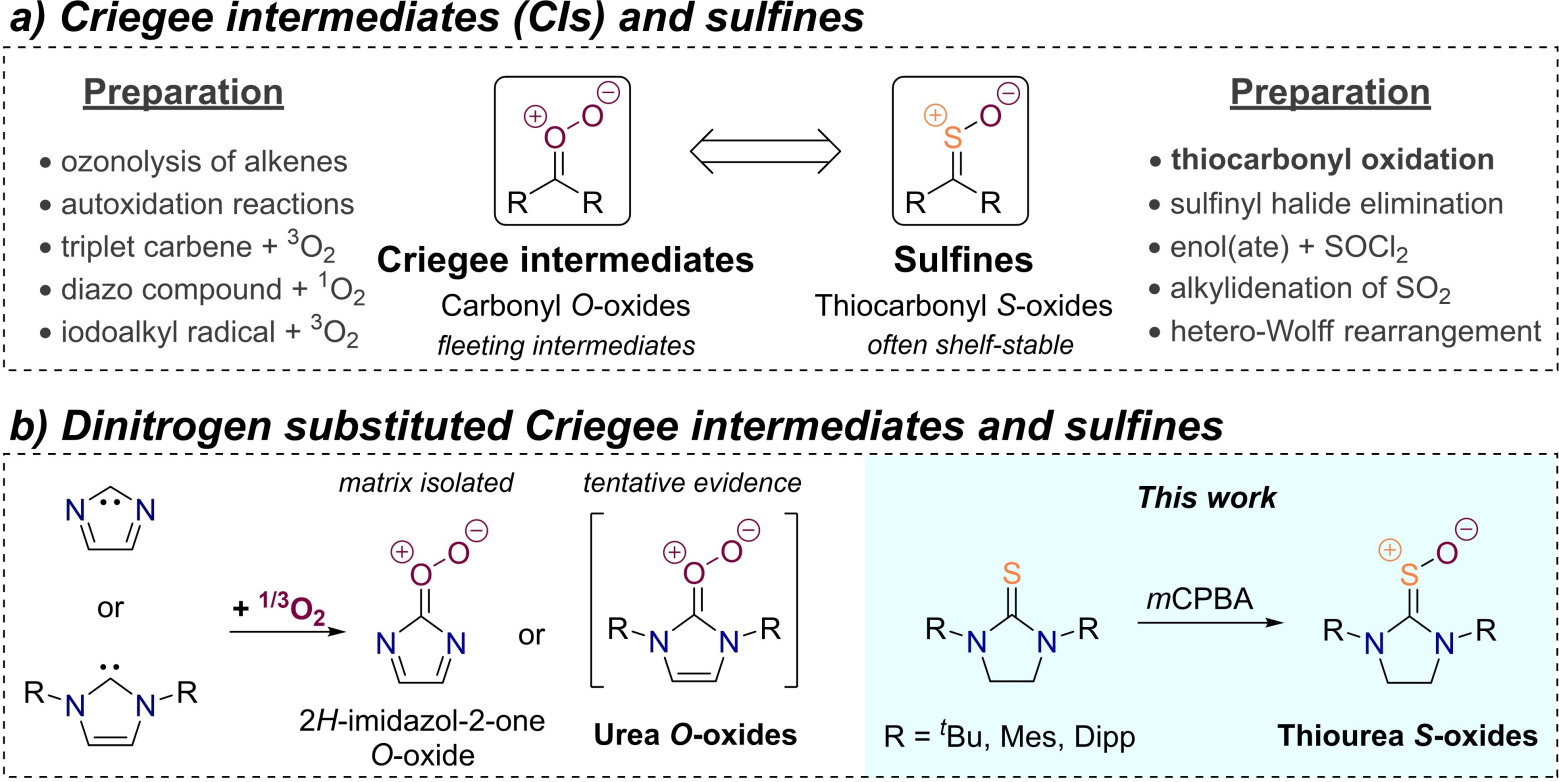
(a) Preparation and structure of Criegee intermediates in comparison to their mono‐sulfur analogues, the sulfines, as well as their respective dinitrogen derivatives (b).

Sulfines are the *S*‐monoxides of thiocarbonyl compounds and therefore possess a characteristic, nonlinear C=S^+^−O^−^ moiety (Figure 1a).^[1]^ They can formally (and practically)^[2]^ be derived from sulfur dioxide by substitution of an oxygen atom with a CR_2_ group and are often isolable, bench‐stable and at times crystalline compounds.^[3]^ There are even a few naturally occurring sulfines such as the lachrymator propanethial *S*‐oxide, which is released from freshly cut onions upon enzymatic action.^[4]^ Sulfines have generated interest, among others, because of their participation in dipolar^[5]^ and Diels‐Alder cycloadditions,^[6]^ their reactions with nucleophiles on sulfur^[7]^ and carbon^[8]^ as well as their potential utility in photochemical sulfur transfer reactions.^[9]^

In contrast to the relative longevity of sulfines, their dioxygen analogues, known as carbonyl *O*‐oxides or Criegee intermediates (CIs, Figure 1a), are ephemeral transients in ozonolysis and autoxidation reactions.^[10]^ CIs can be studied spectroscopically when they are prepared without vibrational excess energy in a thermoneutral reaction in the gas‐phase^[11]^ or when their lifetime gets prolonged by trapping in cryogenic environments.[^10d^, ^12^] In another approach, protection by steric encumbrance makes it possible to observe Criegee intermediates for extended periods of time in low‐temperature solutions as realized in Sander's dimesitylketone *O*‐oxide.^[13]^ In addition to steric effects, Criegee intermediates might enjoy significant electronic stabilization when substituted with nitrogen donor atoms.^[14]^ However, access to these urea *O*‐oxides from the reaction of N‐heterocyclic carbenes (NHCs) with molecular oxygen is challenging because the O_2_ addition does not occur or is rather sluggish in the case of triplet ground state dioxygen,^[15]^ or it was merely possible to observe decomposition products of urea oxides in the reaction employing singlet O_2_ (Figure 1b).^[16]^ So far, 2*H*‐imidazole‐2‐one *O*‐oxide is the only known N‐heterocyclic Criegee intermediate which could be prepared from a σ^0^π^2^ singlet ground state carbene under matrix isolation conditions (Figure 1b).^[17]^ However, the nitrogen atoms cannot act as donors in this case, because the respective lone‐pairs are perpendicular to the molecule's π‐system. Due to a lack of accessibility of urea oxides, we reckoned that their mono‐sulfur analogues, namely thiourea *S*‐oxides, might serve as more easily available and potentially persistent model compounds for CIs;^[18]^ therefore, we set out to examine the viability of their preparation *via* direct thiourea monoxidation (Figure 1b).

The synthesis of sulfines is well established nowadays with several complementary methods coexisting next to each other (Figure 1a).^[1]^ While the first sulfine was reported as early as 1923 in the reaction of camphorsulfonyl chloride with pyridine or triethylamine,^[19]^ this method did not find much use thereafter.^[1c]^ Instead, synthetic diversity is engendered by a plethora of preparation methods entailing the dehydrohalogenation of sulfinyl halides,^[20]^ the sulfenylation of activated methylene compounds with SOCl_2_,[^3c^, ^21^] the alkylidenation of sulfur dioxide,^[2]^ and the hetero‐Wolff rearrangement in α‐diazo sulfoxides.^[22]^ Still, the most versatile sulfine synthesis corresponds to the direct oxidation of thiocarbonyl compounds, which was pioneered by Walter utilizing hydrogen peroxide to oxidize thioacetamide.^[23]^ The method was made broadly applicable thereafter, when Zwanenburg introduced the use of aromatic peracid as an oxidant.^[24]^ Thereby, the preparation of a variety of sulfines became possible including alkyl‐,^[25]^ aryl‐,^[24]^ alkylmercapto‐,^[26]^ as well as chlorosulfines,^[27]^ and even a thioketene *S*‐oxide.^[28]^ While *m*CPBA evolved to a standard oxidizing agent in sulfine synthesis, alternative reagents with varying benefits became available later. For instance, the utilization of dimethyldioxirane promises an easy, non‐aqueous work‐up,^[1b]^ while the high‐yielding oxidation of thiobenzophenones with hydrogen peroxide becomes possible by addition of catalytic amounts of methylrhenium trioxide.^[29]^ For our purposes, direct thiocarbonyl oxidation was the method of choice because the thiourea starting materials are either commercially available or easily synthetically accessible.

**Figure 2:**
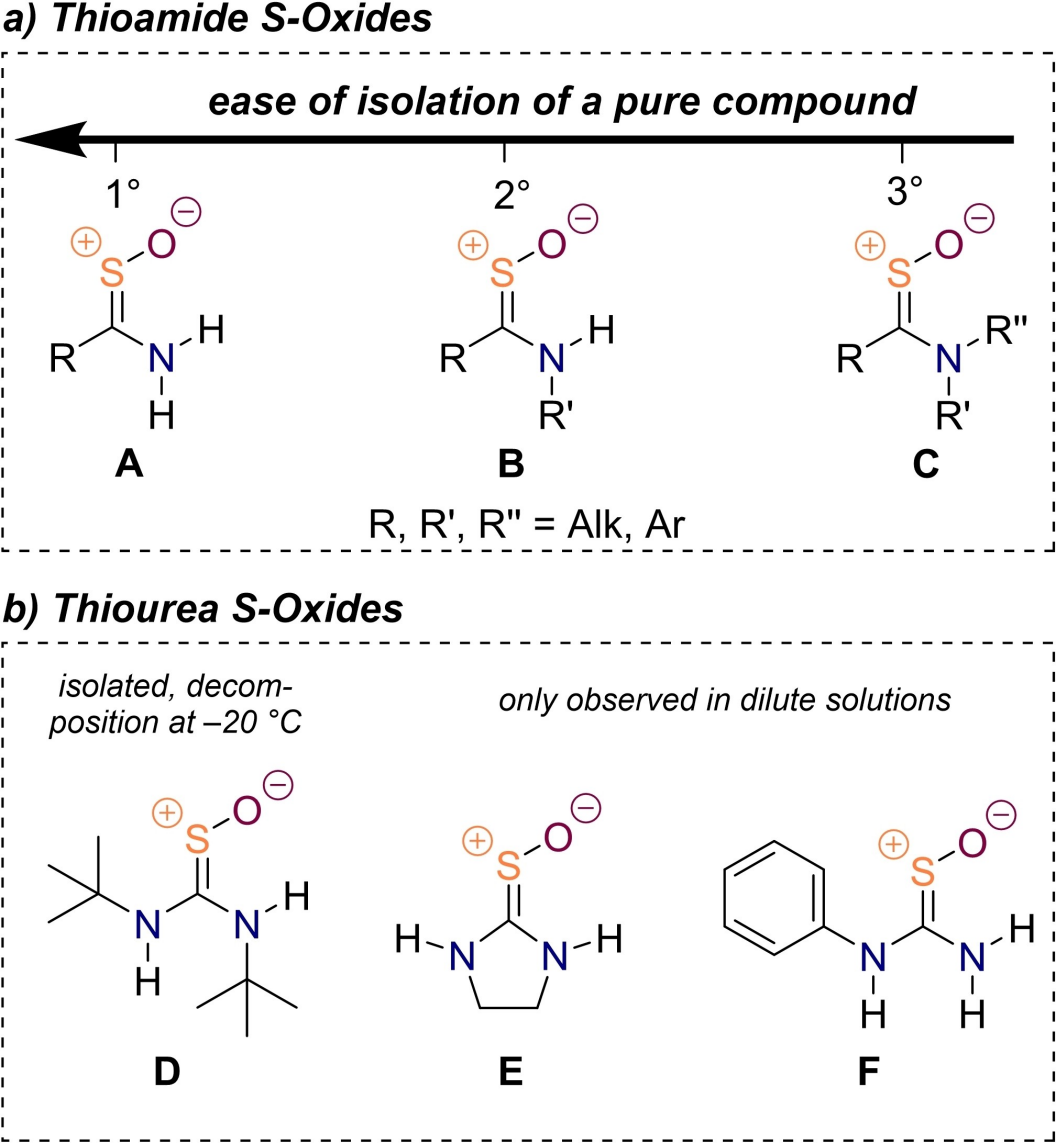
Examples of thioamide *S*‐oxides (a) and thiourea *S*‐oxides (b), which could be obtained by direct thiocarbonyl oxidation reactions.

The oxidation of nitrogen substituted thiocarbonyl compounds has been studied in detail by Walter's group utilizing mostly hydrogen peroxide as the oxidant. Starting with the isolation of thioacetamide *S*‐oxide (Figure 2a), **A**, R=CH_3_), Walter conclusively demonstrated that the reaction product exhibits the structure of an *S*‐oxide and does not correspond to its sulfenic acid tautomer.[^23^, ^30^] Thioacetamide oxide is rather stable and can be stored at 0 °C for many months with little to no signs of decomposition, however, the compound disintegrates upon melting at 136–137 °C.^[23]^ While the oxidation protocol can easily be extended to primary thioamides independent of the nature of the substituent on carbon,^[31]^ difficulties to isolate pure products emerge for secondary, **B**, and even more so for tertiary thioamide *S*‐oxides, **C** (Figure 2a).^[32]^ After a detailed kinetic analysis, Walter later on reported an improved oxidation protocol in acetic acid as a solvent recognizing that tertiary thioamides exhibit a reduced tendency towards oxidation while their corresponding *S*‐oxides are more prone to undergo further oxidative decomposition.^[33]^ The authors conjectured that the lack of acidic hydrogens in tertiary thioamides causes their lability (towards oxidation), but the details remain unclear.

Walter and Randau then went on to study the oxidation of diamino thiocarbonyl compounds, i.e., thioureas, and even managed to isolate *N*,*N’*‐disubstituted *S*‐oxides when they contained bulky alkyl groups such as *tert*‐butyl (**D**, Figure 2b).^[34]^ This is remarkable because, in the case of parent thiourea, the oxidation does not stop at the corresponding *S*‐oxide, but proceeds to the stable *S*,*S*‐dioxide,^[35]^ which is nowadays used as a common bleaching agent in the textile industry.^[36]^ Interestingly, thiourea dioxide does not exhibit hydrogen atoms on the SO_2_ moiety under any conditions^[37]^ and might be viewed as a Lewis acid‐base adduct of diaminocarbene and sulfur dioxide.^[38]^ Although thiourea monoxides like **D** exhibit intramolecular hydrogen bonding, they are unstable and decompose at temperatures as low as −20 °C over the course of weeks.^[34a]^ In a later study, Poulsen *et al*. attempted to prepare thiourea *S*‐oxides with less steric bulk and had some success with ethylenethiourea oxide **E** and phenylthiourea oxide **F** (Figure 2b). However, the latter were reported to be only stable in solution decomposing upon isolation attempts “apparently by disproportionation”.^[39]^ When it comes to thioureas without amide hydrogens, other workers have only observed the formation of amidine salts under oxidizing conditions, and no thiourea *S*‐oxides were prepared this way so far to the best of our knowledge.^[40]^

**Scheme 1:**
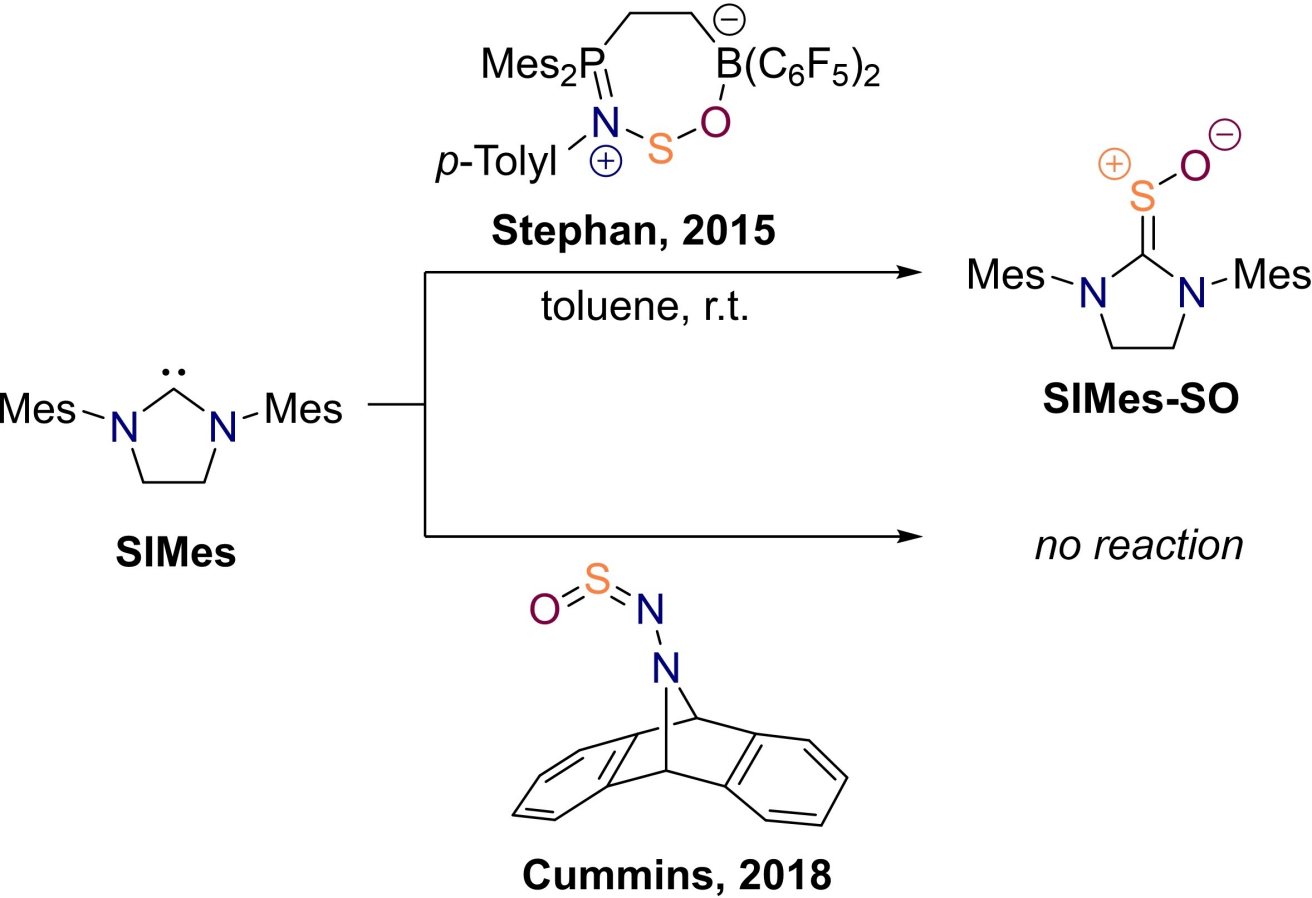
Alternative (attempted) preparations of thiourea *S*‐oxides through formal sulfur monoxide transfer.

Although the direct oxidation method did not yet provide access to thiourea *S*‐oxides without amide hydrogens, these compounds are sufficiently stable for isolation which was demonstrated by Stephan's group in 2015 when they prepared the sulfine **SIMes‐SO** bearing bulky mesityl groups on the nitrogens (Scheme 1).^[41]^ These authors reacted the N‐heterocyclic carbene (NHC) **SIMes** with *N*‐sulfinylamine activated by a P/B frustrated Lewis pair under formal transfer of sulfur monoxide. The structure of the resulting thiourea *S*‐oxide could be confirmed by means of NMR spectroscopy and X‐ray crystallography, although the compound decomposed in solution as expected from previously prepared diaminosulfines. In another attempt to prepare thiourea *S*‐oxides, Cummins's group reacted their anthracene‐based sulfur monoxide releasing precursor with NHCs but, unfortunately, no clean reaction could be observed (Scheme 1).^[42]^

Considering these previous findings, it was our goal to extend the direct oxidation procedure to thioureas based on imidazole and imidazolidine scaffolds which do not bear amide hydrogens. We had hoped that variation of the steric demand of the ancillary substituents on the nitrogens might facilitate the isolation of diaminosulfines as realized in the previously prepared dimesityl derivative **SIMes‐SO**.^[41]^ Moreover, we aimed to comprehend the difficulties encountered in the deliberate monoxidation of nitrogen substituted thiocarbonyls by means of computational quantum chemistry and elaborate on which sulfines can and which cannot be prepared this way due to a facile overoxidation reaction.

## Results and Discussion

**Scheme 2:**
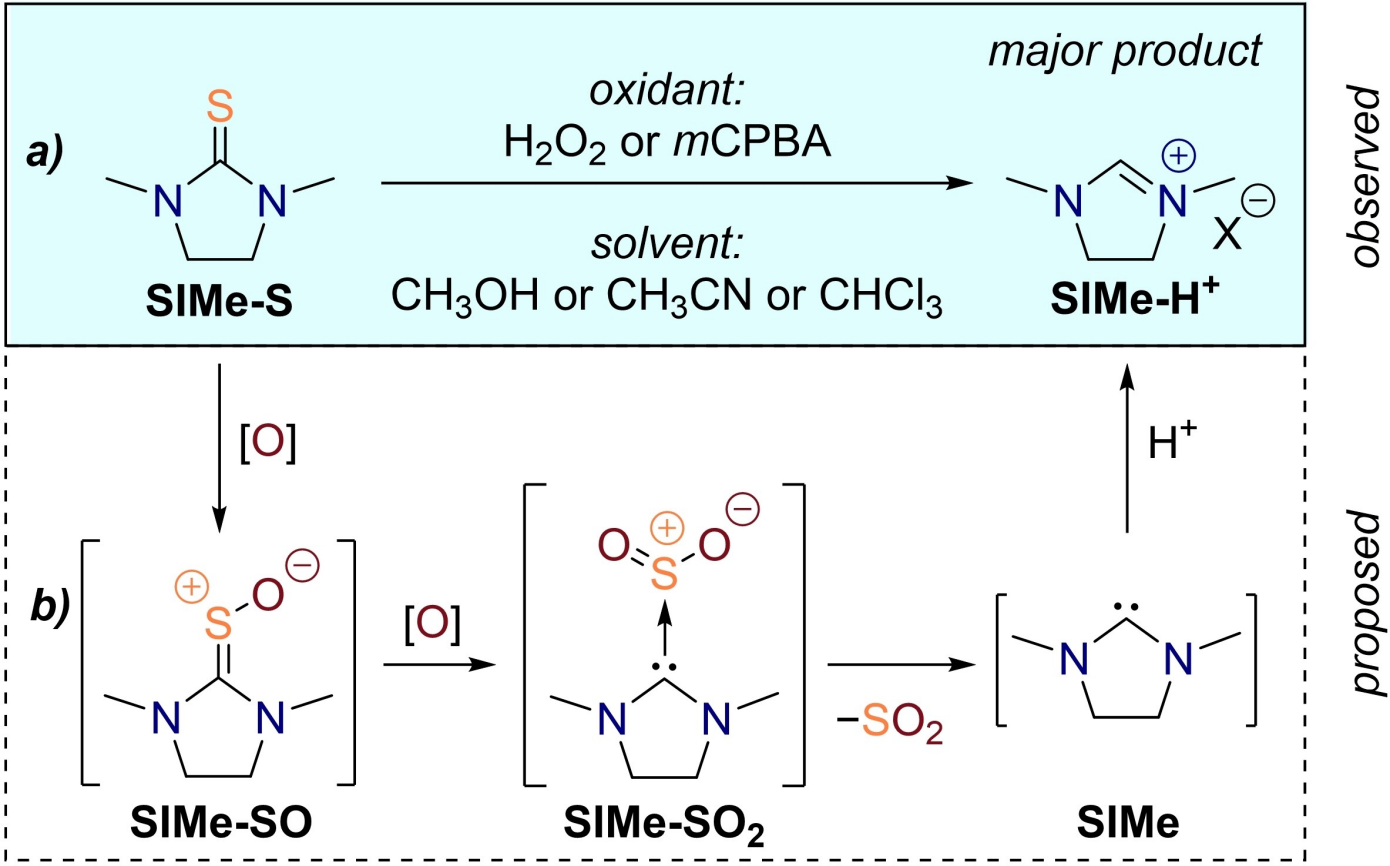
Observed overoxidation of 1,3‐dimethyl‐imidazolidine‐2‐thione upon exposition to different oxidizing conditions **(**a) together with the proposed mechanism of the reaction (b).

For an initial screening of suitable reaction conditions and in order to re‐confirm the difficulties associated with the intentional preparation of thiourea *S*‐oxides, we decided to utilize 1,3‐dimethyl‐imidazolidine‐2‐thione, **SIMe‐S**, as a model substrate and expose it to different oxidizing conditions (Scheme 2a). We opted for aqueous hydrogen peroxide and *m*CPBA as well‐established oxidants in thiocarbonyl oxidation reactions. Regarding solvents, chloroform, acetonitrile, and methanol were chosen as representatives of weakly polar, polar aprotic, and polar protic reaction media, respectively. Commercial availability of their deuterated variants made it possible to conveniently track the reaction progress by means of NMR spectroscopy on a milligram scale. Upon utilization of hydrogen peroxide, we observed a decrease of the thiourea NMR signals and appearance of a new set of signals which were assigned to the **SIMe‐H^+^** ion based on correspondence of the chemical shifts to literature known values and the characteristic low‐field shift of the imidazolidinium proton which is present in non‐protic solvents. The reaction was rather sluggish employing an equimolar amount of hydrogen peroxide and required several hours to reach a total conversion of approximately 33 %. Likewise, the addition of a total amount of three equiv. H_2_O_2_ resulted in the complete conversion of the substrate to the corresponding imidazolidinium ion and no transient reaction intermediate of any kind could be detected in the NMR independent of the employed solvent. Moving on to *m*CPBA as an oxidant, we also only observed the formation of the **SIMe‐H^+^** ion as a main product and never found evidence for any intermediate species. However, the reactions proceeded at a much higher rate and conversions were essentially complete within the period of time it takes to prepare the sample and measure the NMR spectrum (∼10 minutes). Once more, only the addition of a threefold excess of *m*CPBA led to a complete consumption of the thiourea starting material. In general, it appears that the outcome of model thiourea **SIMe‐S** oxidation does not largely depend on the nature of the oxidant, apart from the time it takes to reach completion, with *m*CPBA being the most active one. Considering our goal to prepare sterically encumbered thiourea oxides, it is reasonable to assume that hydrogen peroxide oxidation would be even more sluggish for bulky substates. Henceforth, *m*CPBA was selected as the reagent of choice for all further purposes.

Although we only observed the formation of the imidazolidinium ion **SIMe‐H^+^** in line with preceding literature reports,^[40]^ we reckoned that the desired thiourea monoxide **SIMe‐SO** is still traversed in the course of the reaction (Scheme 2b). Yet, a facile overoxidation of the monoxide could lead to the unstable *S*,*S*‐dioxide **SIMe‐SO_2_**, which can be comprehended as a weakly bound carbene‐SO_2_ Lewis acid‐base adduct.^[38]^ Dissociation of the latter leads to liberation of the free NHC, which in turn gets protonated to the main product **SIMe‐H^+^**. Although the consumption of three equiv. of oxidant would also be in line with the formation of an intermediate thiourea *S,S,S*‐trioxide, these species are generally more stable than the corresponding dioxides and transformation to the observed products under the employed reaction conditions seems unlikely.[^34c^, ^43^]

**Figure 3:**
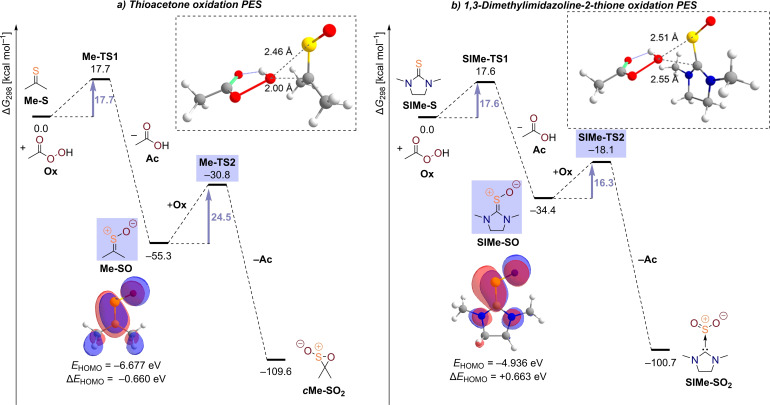
Potential free energy surfaces of the first and second oxidation of thioacetone (a) and 1,3‐dimethylimidazolidine‐2‐thione (b) with peracetic acid at the revDSD‐PBEP86‐D4/def2‐QZVPP(SMD:dichloromethane)//B3LYP‐D3/def2‐TZVPP level of theory.

Our initial screening experiments reemphasize the difficulties to prevent overoxidation in the case of thioureas, which is in stark contrast to the ease of preparation of many other sulfines with, for instance, aryl and alkyl substituents.[^24^, ^25^] Since the involved thioketones often contain bulky groups, it appears that steric hindrance plays a major role in the preparative accessibility of sulfines. However, the difficulties which emerge when amino substituents are involved first and foremost point to the importance of electronic effects which are at play. For this reason, we deployed density functional theory computations at the accurate revDSD‐PBEP86‐D4/def2‐QZVPP(SMD:dichloromethane)//B3LYP‐D3/def2‐TZVPP level of theory and wanted to see whether theory can reproduce this result and help to develop a qualitative understanding of the reaction trends. We started out by computing the first and second oxygen atom transfer reactions onto our model substrate, **SIMe‐S**, in comparison to thioacetone (**Me‐S**) as a simple dialkyl thioketone. For computer time's sake, we used peracetic acid (**Ox**) as an oxidant, in the hope of it being a close enough analog of *m*CPBA. This assumption was verified by comparing the energetics of the **Me‐S** oxidation by peroxyacetic acid and *m*CPBA. Both, reaction heats and barrier heights, differed by no more than 1.5 kcal mol^−1^ for the two peracids, which was deemed acceptable for our purposes (Supporting Information, Table S7). Our computational results are depicted in the comparative potential energy surfaces (PESs) in Figure 3.

The first oxidation step in thioacetone, **Me‐S**, is associated with a free energy barrier of 17.7 kcal mol^−1^ and an exoergicity of −55.3 kcal mol^−1^ (Figure 3a), which is in excellent agreement with the employed experimental temperature of 0 °C and reaction times of 1–5 minutes in the preparation of dialkyl thioketones.^[25]^ The subsequent oxidation of the sulfine results in the formation of an oxathiirane *S*‐oxide, ***c*** **Me‐SO_2_**, and exhibits a significantly higher barrier of 24.5 kcal mol^−1^, while the reaction free energy change is comparable to the first step. We note that there is also an alternative transition state at a free energy of 29.2 kcal mol^−1^ leading to the isomeric, ring‐opened *S*,*S*‐dioxide, however, this reaction path is deemed irrelevant due to the high associated barrier. Given that stoichiometric amounts of oxidant are employed, the sluggish second oxidation step prevents an overoxidation of the substrate even at late reaction stages when sulfine concentrations are elevated. The formation of oxathiirane *S*‐oxide as the final product is already reflected in the transition state geometry of the second oxidation step exhibiting a shorter O−C (2.00 Å) than O−S (2.46 Å) distance between peroxyacid and sulfine. Hence, the oxygen is not transferred onto the sulfur atom, which would result in the formation of an *S*,*S*‐dioxide or sulfene.^[44]^ The tendency of the carbon atom being more nucleophilic than sulfur is in good agreement with the shape of the sulfine's HOMO (inset, Figure 3a) which essentially corresponds to the C=S double bond's π orbital and features a large coefficient of the 2p atomic orbital on carbon. From our computations, which do not consider any sulfine decomposition reactions other than overoxidation, it is understandable that direct peracid oxidation is a powerful preparation method for dialkyl sulfines. Bulky alkyl groups might still be necessary to avoid dimerization and self‐reactions of the generated sulfine.^[45]^

When it comes to the oxidation of thiourea **SIMe‐S**, the barrier height of 17.6 kcal mol^−1^ of the first oxidation step is comparable to the case of thioacetone, albeit the reaction proceeds with a reduced free energy change of only −34.4 kcal mol^−1^ (Figure 3b). The major difference lies in the low barrier height of the second oxidation step of only 16.3 kcal mol^−1^, which is 1.3 kcal mol^−1^ lower than the first barrier in this reaction sequence. The free energy change of the second oxidation step is also almost twice as high as in the first step and provides a considerable driving force (−66.3 kcal mol^−1^). Especially when more sulfine builds up during the course of the reaction, the second oxidation will be faster than the first one preventing an efficient accumulation of the desired monoxide. The product of the overoxidation reaction is the thiourea *S*,*S*‐dioxide, **SIMe‐SO_2_**, which already becomes apparent in the transition state's O−C distance between oxygen atom donor and acceptor molecules being 0.55 Å longer than in **Me‐TS2**. This time, the sulfine's HOMO exhibits a larger coefficient on sulfur highlighting this atom's increased nucleophilicity which is caused by conjugative electron donation from the neighboring nitrogen substituents (inset, Figure 3b). The thiourea dioxide exhibits a non‐planar structure around the sulfur atom in agreement with viewing this compound as a Lewis acid‐base adduct of sulfur dioxide with an NHC.^[38]^ We computed a free energy change of only +6.4 kcal mol^−1^ for the dissociation of the complex. Once even small amounts of NHC are released in the equilibrium, the free carbene could get easily protonated or oxidized under the employed reaction conditions providing additional driving force and explaining the observed reaction outcome (Scheme 2a).

Although our DFT computations are in good agreement with the experimental findings, it still seems bewildering at first that the *S*‐oxidized thiourea **SIMe‐SO** reacts faster with the electrophilic oxidant peracetic acid than the thiourea **SIMe‐S** itself. However, this can be understood by interpreting the oxidation as a nucleophile‐electrophile reaction and looking at the involved frontier orbital interactions and energies. While the thiourea exhibits a HOMO energy of −5.599 eV at the B3LYP/def2‐TZVPP level, the sulfine's HOMO lies much higher at an energy of −4.936 eV making it more amenable for interaction with the peroxy moiety's antibonding σ*‐orbital. Likewise, the orbital energy ordering is the other way around in thioacetone (−6.020 eV) and dimethylsulfine (−6.677 eV). Therefore, the orbital energy difference (Δ*E*_HOMO_) of the HOMOs of sulfine and thiocarbonyl is possibly a simple but meaningful quantity to gauge the utility of the direct oxidation method independent of the nature of the oxidant.

**Figure 4:**
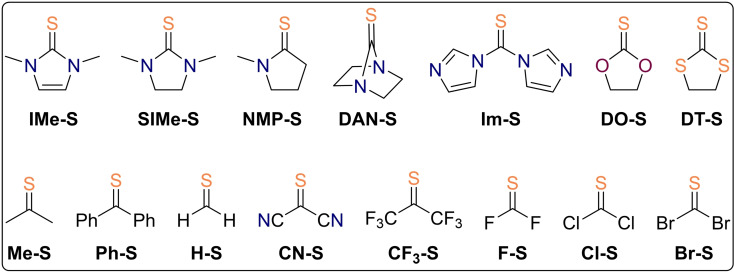
First and second oxidation reactions with peracetic acid were computed for the displayed thiocarbonyl model compounds.

Due to the convincing agreement of our DFT computations with available experimental results, we decided to include more model systems with different substituents into our study in order to get a grasp of which thiocarbonyls can and which ones cannot be selectively oxidized to the corresponding sulfines by direct peracid oxidation (Figure 4). For all the displayed model substrates, we computed the first and second peracetic acid mediated oxidation reactions to the corresponding sulfine and its overoxidation product, respectively. The free energy barriers, Δ*G* ^≠^, and reaction free energies, Δ_r_ *G*, of the two reaction steps are reported in Table 1 together with the nature of the overoxidation product (*S*,*S*‐dioxide vs. oxathiirane *S*‐oxide) and the orbital energy differences Δ*E*_HOMO_ of the HOMOs of the respective sulfine and its corresponding thiocarbonyl.

**Table 1:** Free reaction energies and barrier heights of the two‐step thiocarbonyl oxidation by peracetic acid at the revDSD‐PBEP86‐D4/def2‐QZVPP(SMD:dichloromethane)//B3LYP−D3/def2‐TZVPP level of theory given in units of kcal mol^−1^. HOMO energy differences were calculated at the B3LYP‐D3/def2‐TZVPP level of theory and given in units of eV.

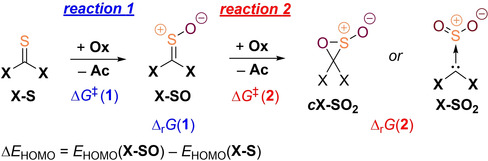						
**X (species)**	Δ*G* ^≠^(**1**)	Δ_r_ *G*(**1**)	Δ*G* ^≠^(**2**)^[a,b]^	Δ_r_ *G*(**2**)	Δ*E*_HOMO_	nature of product
**IMe‐S**	17.2	−31.9	**15.1**	−70.3	+0.741	SO_2_ adduct
**SIMe‐S**	17.6	−34.4	**16.3**	−66.3	+0.663	SO_2_ adduct
**NMP‐S^[c]^**	16.8	−39.7	**18.1**	−58.0	+0.330	SO_2_ adduct
**DAN‐S**	24.3	−49.0	**25.4**	−61.3	−0.560	cyclic
**Im‐S**	25.5	−44.5	**26.1**	−54.2	−0.033	cyclic
**DO‐S**	20.7	−35.4	**21.0**	−59.5	+0.566	SO_2_ adduct
**DT‐S**	18.4	−46.2	**22.7**	−47.5	+0.262	SO_2_ adduct
**Me‐S**	17.7	−55.3	**24.5**	−54.3	−0.657	cyclic
**Ph‐S**	19.3	−53.8	**24.5**	−51.7	−0.290	cyclic
**H‐S**	19.3	−59.0	**25.7**	−52.4	−1.127	cyclic
**CN‐S**	22.9	−58.8	**29.3**	−45.8	−0.421	cyclic
**CF_3_‐S**	25.4	−54.3	**30.2**	−53.6	−0.991	cyclic
**F‐S**	29.6	−35.1	**24.0**	−68.9	+0.386	cyclic
**Cl‐S**	24.9	−48.0	**28.8**	−55.6	+0.096	cyclic
**Br‐S**	25.0	−49.8	**28.6**	−53.7	+0.048	cyclic

[a] When multiple geometries of the sulfine oxidation **TS2** are possible, the barriers relate to the ones with the lowest energy. [b] Traffic light color coding indicates the facility of a selective thiocarbonyl monoxidation with higher ( **green** ), comparable ( **yellow** ) and lower overoxidation barriers ( **red** ). [c] Energetics shown herein relate to the formation and oxidation of the kinetically preferred (by 1.8 kcal mol^−1^) *E*‐isomer.

When going to the structurally related, imidazole‐based thiourea **IMe‐S**, the activation and reaction free energies are very much comparable to the case of saturated derivative **SIMe‐S** (Table 1). The overoxidation reaction barrier is 2.1 kcal mol^−1^ lower than the initial oxidation step's barrier indicating that it will be even more difficult to prepare such an unsaturated diaminosulfine. In the case of monoamino substitution, thioamide **NMP‐S** exhibits first and second oxidation step barrier heights of 16.8 and 18.1 kcal mol^−1^, respectively. Thus, it seems possible that increased amounts of sulfine can accumulate during direct thioamide oxidation, although a selective preparation will still be challenging. This is in agreement with Walter's preparation of tertiary thioamide oxides, although these authors used hydrogen peroxide and not peracid in this transformation.[^32^, ^33^] It is clear that the actual experimental reality is much more complex than we imply in our simple model computations – especially regarding the still unclear role of the amide hydrogens and the involvement of ionic species – however, the observed trends are nicely reproduced.

As was discussed earlier, we reckoned that the +M‐effect exerted by the nitrogen atoms in aminosulfines leads to low overoxidation barriers. In order to further corroborate this assumption, we computed two dinitrogen substituted derivatives in which the resonance of the heteroatomic lone pairs is greatly reduced: the diazanorbornane derivative **DAN‐S**, in which the lone pairs are forced out of the plane of the thiocarbonyl π‐system by its bicyclic structure, and thiocarbonyl‐1,1’‐diimidazole, **Im‐S**, in which the lone pairs partake in the aromatic resonance of the imidazole rings. In both cases, the second oxidation barrier is higher than the first one, which is in good agreement with our hypothesis. In addition, the barrier heights of the first and second oxidation reaction in **DAN‐S** and **Im‐S** are substantially increased exceeding 24 kcal mol^−1^ without exception. This can likely be ascribed to an increased importance of nitrogen's −I‐effect when the +M‐effect is impeded; electronically depleted substrates will be less prone to oxidation by an electrophilic oxidant.

Other lone‐pair donating atoms like oxygen in dioxolane thione **DO‐S** also have a detrimental effect towards the selective preparation of the corresponding sulfine. The first and second oxidation reactions both exhibit barriers of approximately 21 kcal mol^−1^ rendering the utility of the direct oxidation method at least questionable. Indeed, sulfines with two adjacent oxygen atoms are unknown. An improvement can be made by formal replacement of one oxygen atom by sulfur, resulting in xanthates, the oxidation of which successfully provided access to the corresponding *S*‐oxides as a mixture of *E*‐ and *Z*‐isomers in practice.^[46]^ In case of two sulfur donors, the preparation of dithiolane thione *S*‐oxide **DT‐SO** appears practical due to a difference in activation free energies of 4.3 kcal mol^−1^ in the first and second reaction step. This can be expected due to the size mismatch of the involved carbon and sulfur *p*‐orbitals reducing the π‐donating ability of the latter element. Indeed, **DT‐SO** could be prepared in 82 % yield utilizing *m*CPBA as the oxidant.^[26]^ We note that Δ*E*_HOMO_ exhibits a positive sign and therefore fails as a predictor for the applicability of direct thiourea oxidation in this case.

Going away from strong π‐donor substituents, thiobenzophenone **Ph‐S** and parent thioformaldehyde **H‐S** both need 19.3 kcal mol^−1^ of activation when oxidized to the corresponding sulfine, while the overoxidation is associated with an at least 5.2 kcal mol^−1^ higher penalty. In accordance with our computations, aryl derivatives were among the first sulfines prepared by the direct oxidation method.^[24]^ Obviously, peracid oxidation of thioformaldehyde will remain impractical due to the highly reactive nature of substrate and product.^[47]^ Electron‐withdrawing group substituted thiocarbonyl compounds **CN‐S** and **CF_3_‐S** exhibit relatively high barriers towards oxidation of 22.9 and 25.4 kcal mol^−1^, respectively. Their preparation appears feasible because the overoxidation reactions were computed to be rather slow.

The halogen substituted thiocarbonyl compounds make a special case. Their first and second oxidation barriers range among the highest of all considered compounds which can likely be traced back to the pronounced inductive effect of these electronegative elements. While the bar towards the second oxidation step is at least 3.6 kcal mol^−1^ higher in the cases of **Cl‐S** and **Br‐S**, the opposite holds true for **F‐S** emphasizing fluorine's strong +M‐effect. Therefore, once more in agreement with previously reported synthetic procedures, thiophosgene can be oxidized to its *S*‐oxide **Cl‐SO** in a reaction with *m*CPBA.^[27a]^

Summing up the results of our computational study so far, all model substrates bearing lone‐pair donor atoms from the second row of the periodic table exhibit second oxidation barrier heights that are close to or even lower than the first one rendering an intentional monoxidation at least difficult. The substituent effect is reverberated in the nature of the final overoxidation products, where strong +M substituents often lead to the formation of carbene‐SO_2_ adducts instead of isomeric oxathiirane *S*‐oxides, except for the case of difluoro substitution. The predicted formation of SO_2_‐adducts is likely related to the increased stability of the respective donor‐substituted carbenes.^[48]^ The difference in HOMO energy between the thiocarbonyl and its respective *S*‐oxide, ▵*E*_HOMO_, can help to predict when the overoxidation is facile, however, the sign of this number does not provide a strict criterion for the viability of a selective monoxidation. For instance, the ▵*E*_HOMO_ predictor fails badly in case of dithiolane thione **DT‐S** and we must conclude that other factors like electrostatic and steric interactions can come into play, too.

**Figure 5:**
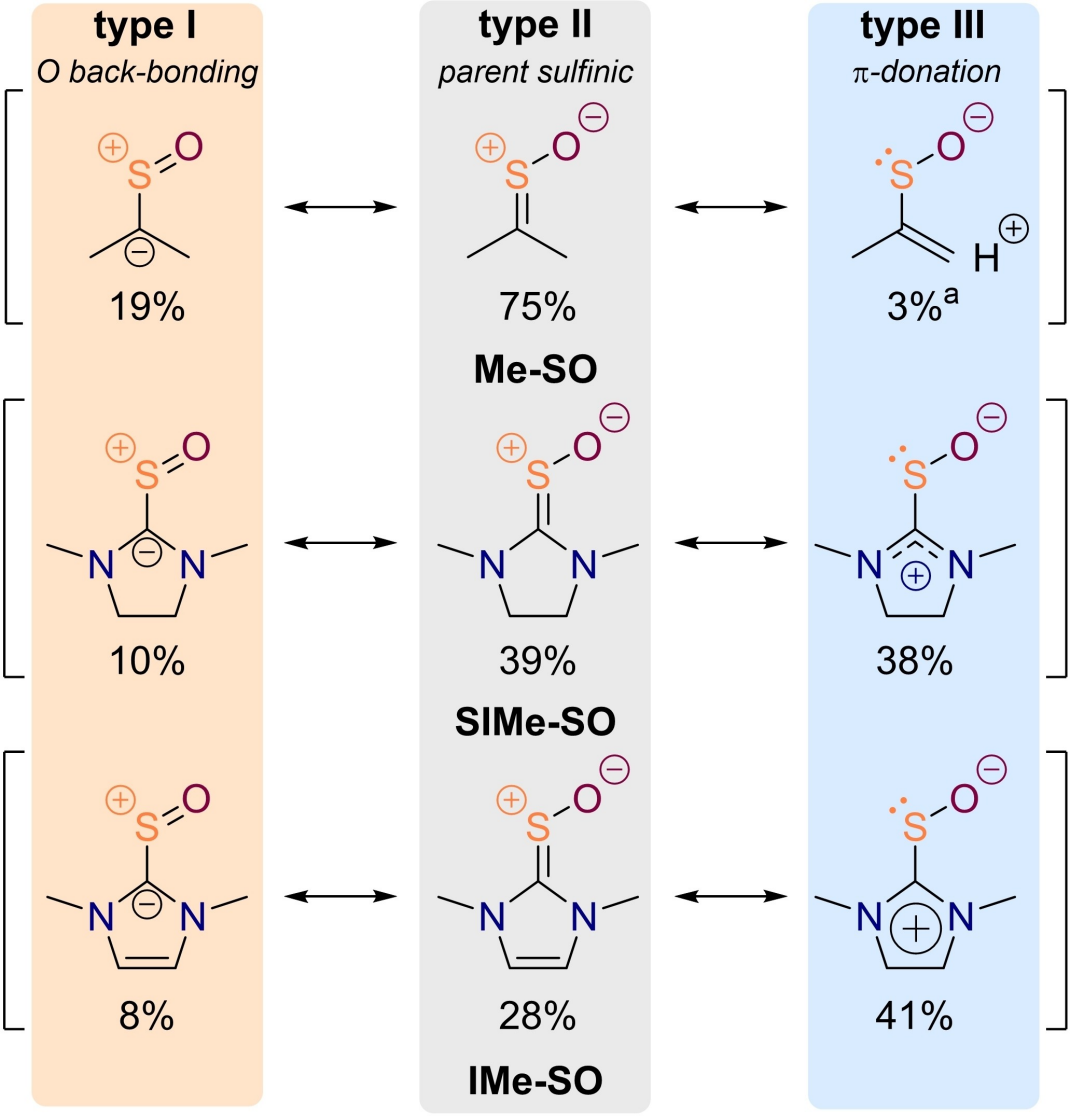
Comparative analysis of the electronic structures of representative sulfines with the help of natural resonance theory at the B3LYP‐D3/def2‐TZVPP level of theory. [a] The weight is summed over four resonance structures of the same character.

Furthermore, to gain a better understanding of the bonding in dinitrogen‐substituted thiocarbonyl *S*‐oxides as compared to simple dialkyl sulfines, we performed a natural resonance theory (NRT) analysis with the NBO 6 program.^[49]^ In the classical dialkyl sulfine **Me‐SO**, the thiocarbonyl *S*‐oxide resonance structure (C=S^+^−O^−^, type II, Figure 5) is the dominant contributor to the electronic structure of the molecule exhibiting a weight of 75 %. Back‐bonding of the terminal oxygen (C^−^−S^+^=O, type I, Figure 5) affords a negative formal charge on the carbon atom in agreement with the increased nucleophilicity of this atom in the peracetic acid oxidation reaction which becomes evident in the close C−O distance in transition state structure **Me‐TS2** (Figure 3). Another resonance structure, in which the sulfur atom accommodates the former C=S double bond as a lone pair (type III, Figure 5), gets stabilized by hyperconjugation from the methyl groups. However, the summed weight over all four qualitatively comparable resonance structures amounts to merely 3 %. Consequently, the “natural polarity” of sulfines is at odds with the installation of strong nitrogen donor substituents on the carbon atom and the weights of both the sulfinic (type II) and oxygen back‐bonding structures (type I) decrease to about a half in thioureas **SIMe‐SO** and **IMe‐SO**. Instead, mesomeric structures with iminium ion character and a C−S single bond become more prevalent (type III). An important characteristic of the latter structures is the fact that the sulfur atom gains an additional lone pair bestowing this center with a higher nucleophilicity. This becomes possible because the formal positive charge gets carried by a nitrogen atom, a rationale that was suggested decades ago by Walter^[34a]^ and is now corroborated by our computational investigations.

**Scheme 3:**
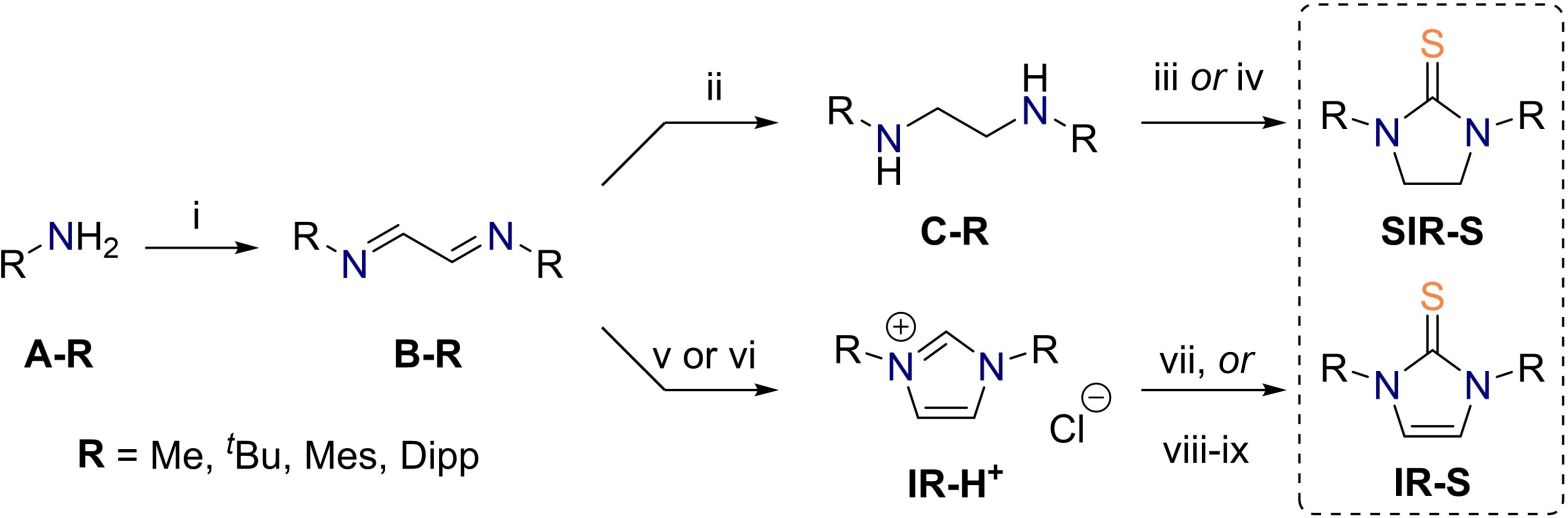
General scheme of saturated **SIR‐S** and unsaturated **IR‐S** thiourea syntheses. (i) Aqueous glyoxal solution, EtOH, r.t. or 0 °C, 1–16 h; (ii) NaBH_4_, MeOH, r.t., 16 h; (iii) CSCl_2_, DIEA, CH_2_Cl_2_, 0 °C, 8–16 h; (iv) CS_2_, I_2_, pyridine, reflux, 8 h; (v) paraformaldehyde, TMSCl, EtOAc, 50 °C, 5 h; (vi) bis(dimethylamino)methane and acetyl chloride, CH_2_Cl_2_ 0 °C, 10 min, then 70 °C, 30 min; (vii) DBU, S_8_, MeOH, pyridine, 70 °C, 6 h; (viii) ^*t*^BuOK, argon, THF, r.t., 1 h; (ix) S_8_, THF, r.t., 8 h.

While our DFT computations make the difficulties of a selective thiourea monoxide preparation understandable due to nitrogen's strong +M‐effect, we conjectured that the desired compounds could still become experimentally available if only they are protected by sufficient steric encumbrance. The assumption held here is that for thioureas with bulkier groups it might be easier to hinder the second oxidation step by blocking the oxidant's π‐type approach to the reactive center of the respective sulfine. A similar strategy was applied by Goto's group, who performed the direct oxidation of thiols to sterically protected sulfenic acids, a likewise elusive monoxidized sulfur species.^[50]^ Therefore, we prepared a series of saturated (**SIR‐S**) and unsaturated cyclic thioureas (**IR‐S**) according to the general procedure outlined in Scheme 3. Both series contain four compounds bearing substituents on the nitrogens of varying steric demand including methyl (**Me**), *tert*‐butyl (^***t***^ **Bu**), mesityl (**Mes**), and 2,6‐diisopropylphenyl (**Dipp**) groups. Unless the diamines **C‐R** or the imidazolium salts **IR‐H^+^** were commercially available, they were synthesized from the respective amines **A‐R** and glyoxal, furnishing us with the diimines **B‐R**.^[51]^ Thereafter, the latter were reduced to respective diamines **C‐R** and cyclized into saturated thioureas **SIR‐S** with thiophosgene^[52]^ or carbon disulfide.^[53]^ Alternatively, the diimines **B‐R** were converted into the imidazolium salts **IR‐H^+^**,^[54]^ which were eventually transformed into the unsaturated thioureas **IR‐S** *via* reaction of the liberated respective carbene with elemental sulfur.[^40a^, ^55^]

**Figure 6:**
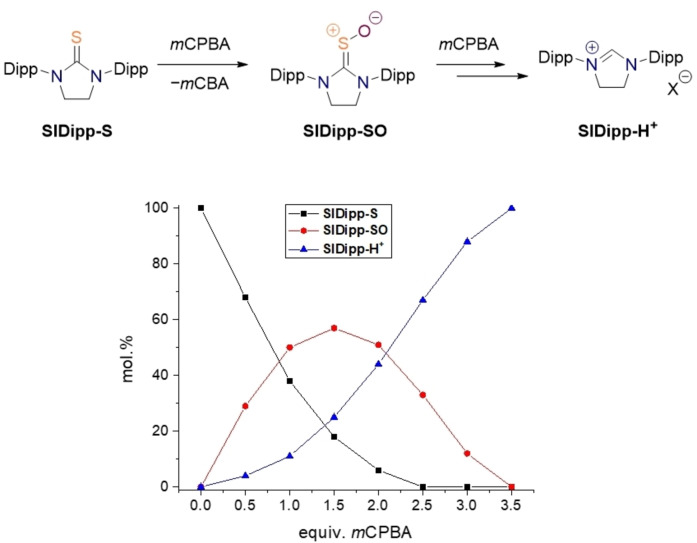
NMR‐scale screening of the **SIDipp‐S** oxidation adding *m*CPBA in increments of 0.5 equiv. in [D_4_]‐methanol. Molar ratios were calculated from the integrals of the corresponding CH_2_‐proton signals.

Starting out with the saturated thioureas **SIR‐S**, we subjected the individual compounds of this series to another NMR‐scale reaction screening adding the oxidant in increments of 0.5 equiv. The best and most consistent results were obtained for the *m*CPBA/methanol reaction system; hence, the results reported in the following refer to these conditions. In all cases, we once more observed the formation of the imidazolidinium ions **SIR‐H^+^** as the main final products. However, for thioureas **SI** ^***t***^ **Bu‐S**, **SIMes‐S** and **SIDipp‐S**, a new set of peaks was detected in the ^1^H NMR spectra which clearly did not correspond to a urea by‐product from comparison to literature‐known chemical shifts. The new compounds rather behaved like an intermediate which would first appear in low concentrations, increase in amount upon addition of more equiv. of *m*CPBA, peak at 23 mol % for **SItBu‐S**, 56 mol % for **SIMes‐S**, and 58 mol % for **SIDipp‐S**, respectively, in order to finally disappear completely in an excess of oxidant (Figure 6, also the Supporting Information, Figure S1b–c). The molecular masses of the detected intermediates were found to conform to the respective protonated thiourea *S*‐oxides by means of HPLC‐ESI–MS and ESI‐HR‐MS lending confidence into this assignment. When the NMR‐scale oxidations were stopped at an intermediate stage, the *S*‐oxide **SI** ^***t***^ **Bu‐SO** bearing the least steric bulk was found to be unstable in solution. The compound disappeared within 24 h after the reaction was performed, leaving only thiourea and imidazolidinium ion behind. In contrast, aryl‐substituted thiourea *S*‐oxides were perfectly stable in methanol solution and showed no signs of decomposition even after weeks at room temperature. Since we suspected that the *ortho*‐alkyl substituents on the aromatic rings are important in preventing the overoxidation and providing kinetic stability, we additionally prepared plain diphenylethylenethiourea (**SIPh‐S**) in analogy to the other saturated thioureas and subjected it to oxidation. As expected, no traces of an intermediate thiourea *S*‐oxide could be detected in line with our hypothesis. Curiously, the oxidation of **SIPh‐S** also leads to the formation of a substantial amount of the corresponding urea, which is not the case for all other studied substrates.

**Scheme 4:**
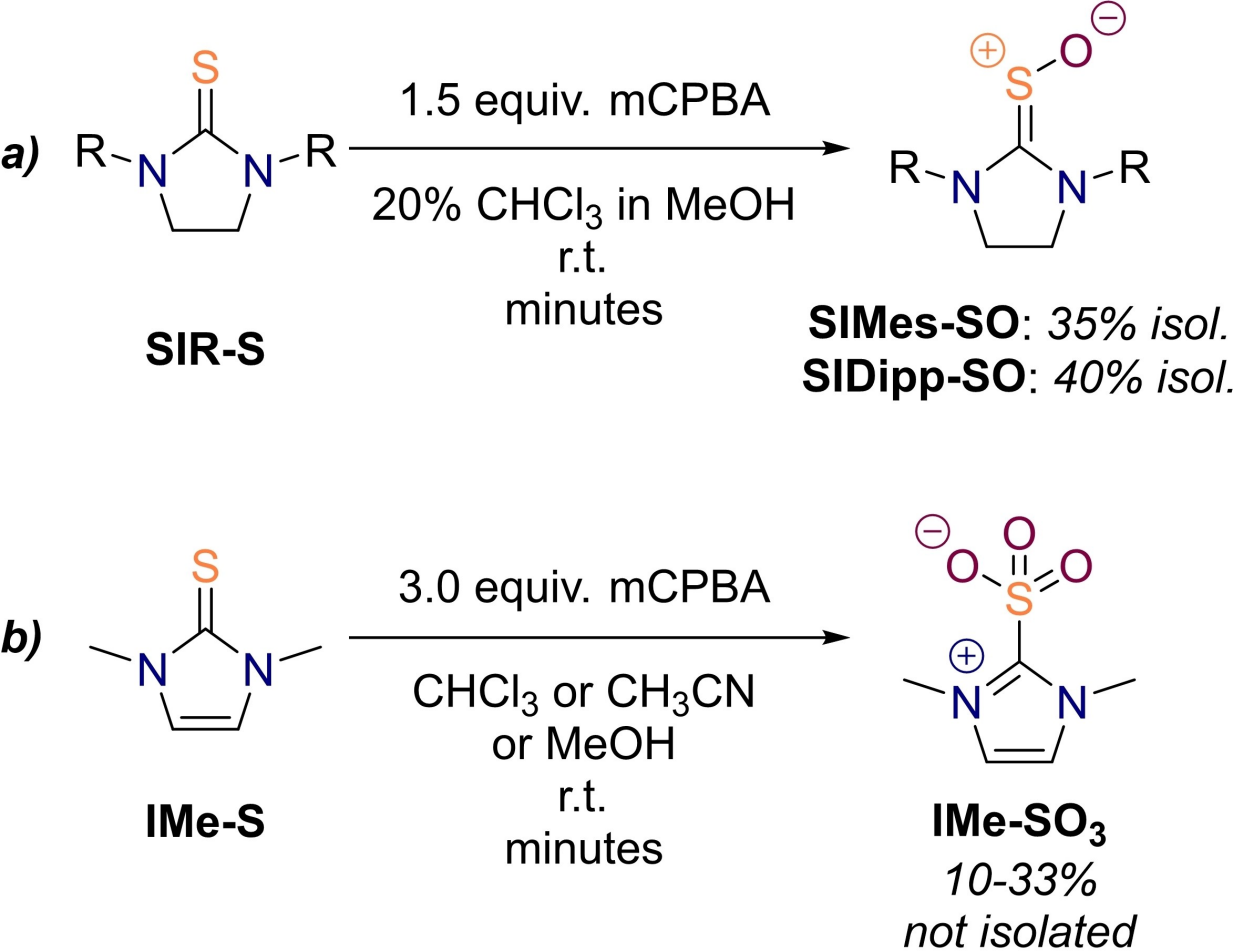
Selected results from the oxidation of cyclic (a) saturated and (b) unsaturated thioureas.

Encouraged by the success of our screening experiments, we attempted to prepare sterically encumbered thiourea oxides on a ∼100 mg scale in order to isolate them as pure substances. Due to the limited stability of **SI** ^***t***^ **Bu‐SO** in solution, we decided to undertake the preparative oxidation of only the aryl substituted thioureas (**SIMes‐S** and **SIDipp‐S**). Since an incomplete dissolution of the starting material turned out to be detrimental to the oxidation, we added up to 20 vol % of chloroform or dichloromethane to the reaction mixture. After dropwise addition of an *m*CPBA solution, the reaction mixture was allowed to stir for a couple of minutes before concentration *in vacuo*. Performing the reaction at lower temperatures had no benefit and was in fact counterproductive due to precipitation of the starting material. The crude reaction mixture was thereafter subjected to reversed‐phase medium pressure liquid chromatography with aqueous methanol as a mobile phase leading to isolation of the sulfines **SIMes‐SO** and **SIDipp‐SO** in 35 % and 40 % yield, respectively (Scheme 4a). It is remarkable that the herein prepared thiourea *S*‐oxides appear symmetric on the NMR as opposed to most other ordinary sulfines. A variable‐temperature ^1^H NMR study of **SIDipp‐SO** in methanol solution indicated that the rotation around the formal C=S double bond cannot even be fully frozen out at 183 K (Figure S77, Supporting Information).

**Figure 7:**
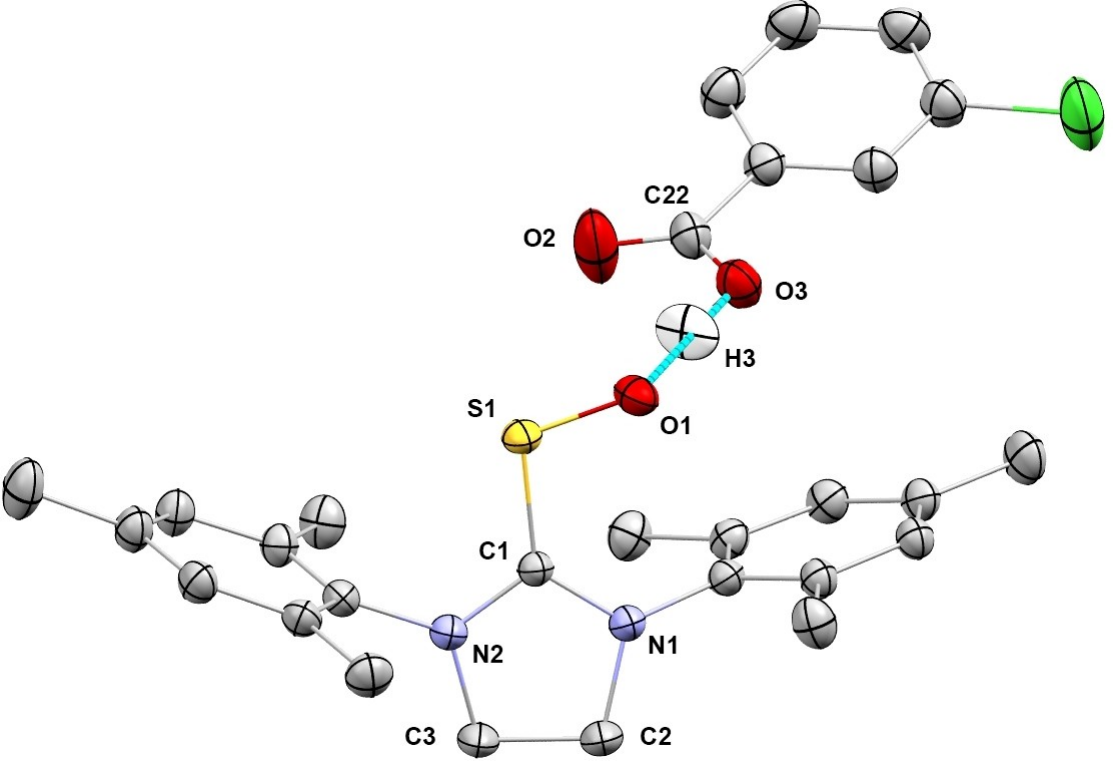
Molecular structure of **SIMes‐SO⋅*m*CBA** in the solid state. Thermal ellipsoids are shown at the 50 % probability level. Hydrogen atoms, except the one partaking in hydrogen bonding, have been omitted for clarity. Selected distances [pm] and angles [°]: C1−N1 134.1, C1−N2 133.2, C1−S1 171.3, S1−O1 155.6, C1−S1−O1 106.8, O1−O3 247.1, O3−C22 130.8, O2−C22 120.4.

Single crystals suitable for X‐ray crystallography were grown at room temperature from an equimolar mixture of **SIMes‐SO** and residual **mCBA** in an ethanol solution which we had obtained from an initial unsuccessful chromatography run. The resulting X‐ray structure in Figure 7 confirms the importance of steric shielding provided by the ancillary mesityl substituents. The aryl rings assume a conformation that is approximately perpendicular to the N‐C‐N plane, so that the protruding methyl groups can protect the C=S^+^−O^−^ moiety thereby accounting for the kinetic stability of the *S*‐oxide. Moreover, the molecular packing of the thiourea *S*‐oxide and the carboxylic acid in a co‐crystal results in a close O⋅⋅⋅H−O (O1−O3) distance of only 2.471 Å, which is well within the range associated with hydrogen bonding.^[56]^ The prominent H‐bond interaction in the solid state might also be present in solution, offering additional stabilization in protic solvents as observed in our experiments (see below). Perhaps this is not surprising, since all thiourea *S*‐oxides obtained earlier by the direct oxidation method contained amidic hydrogens making the stabilization by intramolecular hydrogen bonding possible.[^34a^, ^39^]

**Scheme 5:**
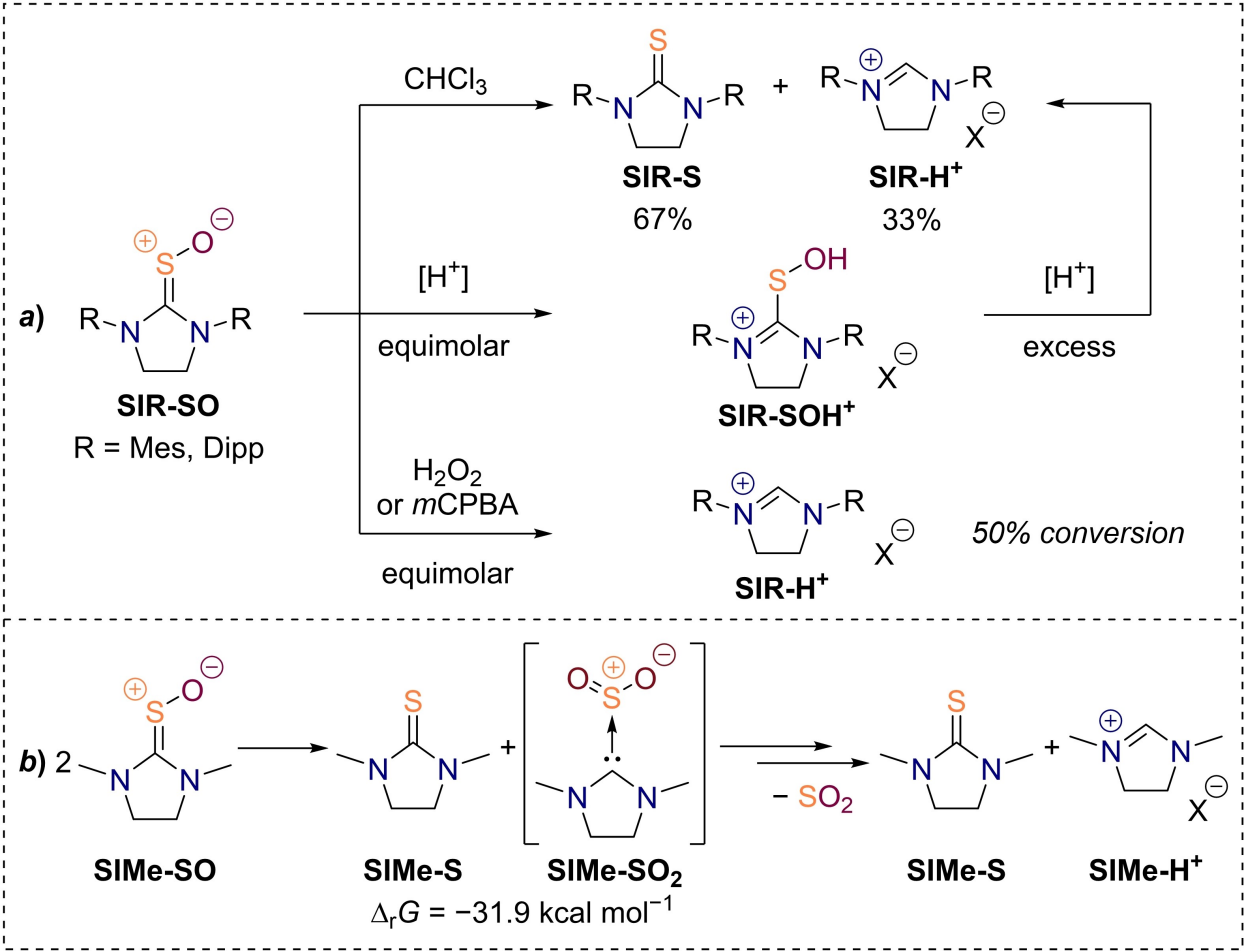
Stability of the isolated thiourea *S*‐oxides **(**a) and their suggested routes of decomposition (b). Free energy change of initial disproportionation step was computed at the revDSD‐PBEP86‐D4/def2‐QZVPP(SMD:dichloromethane)//B3LYP‐D3/def2‐TZVPP level of theory.

The isolated saturated thiourea *S*‐oxides **SIMes‐SO** and **SIDipp‐SO** are stable for weeks as solids and in alcohol solutions at room temperature. Moreover, they are not destroyed by exposure to strong bases, such as potassium hydroxide. Addition of an equimolar amount of *m*CPBA to the isolated thiourea *S*‐oxides oxidizes half of it to the corresponding imidazolidinium salt within minutes, while the addition of one equiv. of aqueous hydrogen peroxide leads to the same result albeit only over the course of a week (Scheme 5a). As opposed to their relative longevity in methanol, the thiourea oxides are quite unstable in weakly polar solvents like chloroform or dichloromethane, decaying into the corresponding thioureas and imidazolidinium salts in an approximate ratio of 2 : 1. Upon addition of one equiv. of hydrochloric acid to a methanolic solution of **SIDipp‐SO**, a significant change of the ^1^H NMR chemical shifts is observed, while the ESI‐MS spectrum remains unchanged. Thus, we attribute the observed spectral changes to an *O*‐protonation of the thiourea oxide. When an excess of hydrochloric acid gets added, the compound eventually begins to decay into thiourea and imidazolidinium salt over the course of several days, once again in a ratio of 2 : 1. Likewise, Walter observed the decomposition of tertiary thioamide *S*‐oxides into a mixture of the respective thioamides and amides, which he ascribed to an initial oxygen atom transfer reaction from one sulfine molecule to another.^[33]^ We computed the free energy change of said disproportionation reaction for the thiourea *S*‐oxide **SIMe‐SO** and found a substantial driving force of −31.9 kcal mol^−1^ (Scheme 5b). Unfortunately, we were not able to compute a barrier of the reaction at the utilized level of theory.

Finally, we studied the incremental oxidation of the unsaturated cyclic thioureas **IMe‐S** through **IDipp‐S**. We found the reaction to proceed quickly to the corresponding imidazolium salts **IR‐H^+^** as well, but no intermediate whatsoever could be detected in the ^1^H NMR spectra, unfortunately. This might be expected from our computational analysis, which revealed an even lower overoxidation barrier than in saturated thioureas (*cf*. Table 1). However, we observed a strong yellow coloration of the reaction mixture upon addition of the first portions of oxidant, which only vanished when the calculated excess of 3.0 equiv. of *m*CPBA was reached. This might possibly be ascribed to the intermediacy of conjugated thiourea *S*‐oxides at a concentration below the NMR detection threshold. Thus, we are afraid that the steric shielding provided by the employed substituents is not sufficient to counteract nitrogen's +M effect when it is combined with the conjugation to a remote double bond.

Despite the failure to prepare unsaturated thiourea oxides, a new set of signals was found in the ^1^H NMR spectra of the incremental **IMe‐S** oxidation, which did not disappear in an excess of oxidant, but was growing in intensity instead (Supporting Information, Figure S1a). The oxidation by‐product could be partially characterized and was assigned to the *S*,*S*,*S*‐trioxide **IMe‐SO_3_** (Scheme 4b). A similar compound, namely 1,3‐diisopropyl‐4,5‐dimethylimidazolethione‐*S*,*S*,*S*‐trioxide, was previously synthesized by Kuhn's group.^[43]^ The chemical shift of the quaternary carbon atom in our compound **IMe‐SO_3_** amounts to 147.0 ppm, which is reasonably close to the 145.9 ppm resonance reported in the aforementioned study.

## Conclusions and Outlook

We managed to successfully prepare and isolate bulky thiourea *S*‐oxides without amidic hydrogens through direct thiourea monoxidation. This is remarkable, because thioureas usually tend to easily over‐oxidize leading to the production of desulfurized products. We were able to describe this undesired reactivity by means of a comparative DFT study, which revealed striking differences between thioureas and thioketones. Although the oxidation of sulfines from ordinary thioketones typically faces significant barriers, the reaction of thiourea *S*‐oxides with peracids necessitates less activation energy than the oxidation of thioureas themselves. This can be understood from the appearance of new resonance structures in thiourea oxides, in which strong π‐donation from the nitrogen atoms places a lone pair of electrons on the sulfur center, effectively making this atom more nucleophilic and thereby more susceptible to peracid oxidation. We have discovered that thiourea *S*‐oxides based on saturated five‐membered rings can still be isolated on the condition that they contain bulky mesityl or diisopropylphenyl groups, and that the yields and their stability are greater in protic solvents. The structure of the *S*‐oxide **SIMes‐SO** as a complex with *m*‐chlorobenzoic acid could be confirmed by X‐ray crystallography, which emphasizes the stabilization of the nitrogen‐substituted C=S^+^−O^−^ group by hydrogen bonding. In this regard, we even observed the protonation of the thiourea *S*‐oxide **SIDipp‐SO** by aqueous hydrochloric acid. Unlike classical sulfines, our sterically hindered thiourea *S*‐oxides exhibit low rotational barriers of the bent thiocarbonyl *S*‐oxide moiety and easily undergo disproportionation reactions in aprotic solvents. The steric protection by the employed bulky groups is not sufficient, however, to make the isolation of unsaturated, imidazole‐based thiourea *S*‐oxides **IR‐SO** possible. Therefore, we are currently developing a new preparation method of said compounds. With regards to the currently elusive urea *O*‐oxides, we hypothesize that their detection might also become feasible when stabilized by sufficient steric bulk and solvation in protic reaction media.

## Methods Section

**Computations**: All geometries were fully optimized at B3LYP‐D3/def2‐TZVPP level of theory and characterized as either minimum energy structures or transition states by vibrational frequency computations.^[57]^ The reaction paths associated with the optimized transition states were further confirmed by following the intrinsic reaction coordinate (IRC) which aided to determine the nature of the overoxidation product (*S*,*S*‐dioxide *vs*. oxathiirane *S*‐oxide). Thermal and free energy corrections were obtained at the same level of theory applying a rigid rotor‐harmonic oscillator approximation as implemented in the *Gaussian16* electronic structure code.^[58]^ The free energies were further corrected regarding the 1 M standard state by adding 1.89 kcal mol^−1^ to the energies of all structures. In order to improve the electronic energy, single points were computed with the revDSD‐PBEP86‐D4 double hybrid functional in combination with the def2‐QZVPP basis set and the RIJK approximation.^[59]^ Solvent effects were considered implicitly in the single point energy computations employing Truhlar's SMD model for dichloromethane.^[60]^

**Exemplary procedure for the preparation of SIDipp‐SO**: In a 100 mL round‐bottom flask, 295 mg (0.70 mmol) of **SIDipp‐S** were dissolved in a mixture of 15 mL of chloroform and 60 mL of methanol. Then, a solution of 235 mg (1.05 mmol, 77 % pur.) of *m*CPBA in 2 mL of methanol was added dropwise at room temperature. The reaction mixture was allowed to stir for 2 minutes followed by concentration *in vacuo*. The residue was redissolved in methanol, deposited onto 4.0 g of silica, and the solvent was evaporated. The silica was then packed into a pre‐column and the desired thiourea *S*‐oxide was purified by means reversed‐phase medium pressure liquid chromatography on a PF‐15C18HQ‐F0012 column using aqueous methanol as a mobile phase. The product can be identified by an absorption feature around 300 nm in the UV/visible spectrum, where no substantial absorption is observed for any other compound in the mixture. After removing the solvent under reduced pressure, **SIDipp‐SO** was obtained as an off‐white solid in 40 % yield (122 mg). ^1^H NMR (400 MHz, methanol‐d_4_, 25 °C): δ=1.38 (d, J=6.9 Hz, 12H, CH_3_), 1.43 (d, J=6.9 Hz, 12H, CH_3_), 3.27 (hept, J=6.9 Hz, 4H, CH), 4.19 (s, 4H, CH_2_), 7.27 (d, J=7.7 Hz, 4H, Ar−H), 7.41 ppm (t, J=7.7 Hz, 2H, Ar−H). ^13^C NMR (partial, 100 MHz, methanol‐d_4_, 25 °C): δ=25.3 (CH_3_), 26.0 (CH_3_), 31.2 (CH), 55.5 (CH_2_), 126.3 (Ar−H), 132.0 (Ar−H), 148.8, 185.1 ppm (CSO, obtained from HMBC). IR (ATR): ν=916 (s, S−O stretch, composite), 936 (vs, S−O stretch, composite), 973 cm^−1^ (s, S−O stretch, composite). HRMS (ESI): m/z calcd. for C_27_H_39_N_2_OS^+^: 439.27776 [*M*+H]^+^; found: 439.27805.

**X‐ray crystallography**: 2201282 **SIMes‐SO⋅** ***m*** **CBA** contains the supplementary crystallographic data for this paper. These data are provided free of charge by the joint Cambridge Crystallographic Data Centre and Fachinformationszentrum Karlsruhe Access Structures service.

## Conflict of interest

The authors declare no conflict of financial interest.

1

## Supporting information

As a service to our authors and readers, this journal provides supporting information supplied by the authors. Such materials are peer reviewed and may be re‐organized for online delivery, but are not copy‐edited or typeset. Technical support issues arising from supporting information (other than missing files) should be addressed to the authors.

Supporting Information

## Data Availability

The data that support the findings of this study are available in the supplementary material of this article.
