# Supplementary material for: Synthesis of Sterically Encumbered Thiourea S‐Oxides through Direct Thiourea Oxidation
Source: Chemistry. 2022 Dec 1;29(4):e202203005. doi: 10.1002/chem.202203005 (PMC10107457; doi:10.1002/chem.202203005)
Supplement: Supplementary file 1 — Supporting Information [file CHEM-29-0-s001.pdf]

Chemistry–A European Journal

Supporting Information

Synthesis of Sterically Encumbered Thiourea *S*-Oxides through Direct Thiourea Oxidation

Serhii Medvedko, Markus Ströbele, and J. Philipp Wagner*

Table of Contents

I. Preparation of thioureas S3
1. Preparation of 1,3-dimethylimidazolidine-2-thione (**SIMe-S**) S3
2. Preparation of 1,3-di-*tert*-butylimidazolidine-2-thione (**SI^{t}Bu-S**) S3
3. Preparation of 1,3-diphenylimidazolidine-2-thione (**SIPh-S**) S3
4. Preparation of *N,N′*-dimesitylethanediimine (**B-Mes**) S4
5. Preparation of *N,N′*-dimesitylethanediamine (**C-Mes**) S4
6. Preparation of 1,3-dimesitylimidazolidine-2-thione (**SIMes-S**) S4
7. Preparation of *N,N′*-bis(2,6-diisopropylphenyl)ethanediimine (**B-Dipp**) S5
8. Preparation of *N,N′*-bis(2,6-diisopropylphenyl)ethanediamine (**C-Dipp**) S5
9. Preparation of 1,3-bis(2,6-diisopropylphenyl)imidazolidine-2-thione (**SIDipp-S**) S5
10. Preparation of 1,3-dimethylimidazole-2-thione (**IMe-S**) S6
11. Preparation of *N,N′*-di-*tert*-butylethanediimine (**B-^{t}Bu**) S6
12. Preparation of 1,3-di-*tert*-butylimidazolium chloride (**I^{t}Bu-H$^+$**) S6
13. Preparation of 1,3-di-*tert*-butylimidazole-2-thione (**I^{t}Bu-S**) S7
14. Preparation of 1,3-dimesitylimidazolium chloride (**IMes-H$^+$**) S7
15. Preparation of 1,3-dimesitylimidazole-2-thione (**IMes-S**) S7
16. Preparation of 1,3-bis(2,6-diisopropylphenyl)imidazolium chloride (**IDipp-H$^+$**) S8
17. Preparation of 1,3-bis(2,6-diisopropylphenyl)imidazole-2-thione (**IDipp-S**) S8
II. Oxidation of thioureas. S8
1. General procedure of NMR-scale screening of thioureas S8
2. General procedure of NMR-scale incremental oxidation of selected thioureas S8
3. Oxidation of 1,3-dimethylimidazol-2-thione (**IMe-S**) S9
4. Oxidation of 1,3-di-*tert*-butylimidazolidine-2-thione (**SI^{t}Bu-S**) S10
5. Preparation of **SIMes-SO** S10
6. Preparation of **SIDipp-SO** S10
III. Spectral data S12
1. 1,3-Dimethylimidazolidine-2-thione (**SIMe-S**) S12
2. 1,3-Di-*tert*-butylimidazolidine-2-thione (**SI^{t}Bu-S**) S14
3. 1,3-Diphenylimidazolidine-2-thione (**SIPh-S**) S16
4. *N,N′*-Dimesitylethanediimine (**B-Mes**) S18
5. *N,N′*-Dimesitylethanediamine (**C-Mes**) S20
6. 1,3-Dimesitylimidazolidine-2-thione (**SIMes-S**) S22
7. *N,N′*-Bis(2,6-diisopropylphenyl)ethanediimine (**B-Dipp**) S24
8. *N,N′*-Bis(2,6-diisopropylphenyl)ethanediamine (**C-Dipp**) S26
9. 1,3-Bis(2,6-diisopropylphenyl)imidazolidine-2-thione (**SIDipp-S**) S28
10. 1,3-Dimethylimidazole-2-thione (**IMe-S**) S30
11. *N,N′*-Di-*tert*-butylethanediimine (**B-^{t}Bu**) S32
12. 1,3-Di-*tert*-butylimidazolium chloride (**I^{t}Bu-H$^+$**) S34

13. 1,3-Di-*tert*-butylimidazole-2-thione (**I^{t}Bu-S**) S36
14. 1,3-Dimesitylimidazolium chloride (**IMes-H$^+$**) S40
15. 1,3-Dimesitylimidazole-2-thione (**IMes-S**) S42
16. 1,3-Bis(2,6-diisopropylphenyl)imidazolium chloride (**IDipp-H$^+$**) S44
17. 1,3-Bis(2,6-diisopropylphenyl)imidazole-2-thione (**IDipp-S**) S46
18. **IMe-SO$_3$** S48
19. **SI^{t}Bu-SO** S50
20. **SIMes-SO** S52
21. **SIDipp-SO** S56
22. Temperature-dependent ^{1}H-NMR experiment of **SIDipp-SO** in MeOD S59
23. Decay of **SIDipp-SO** in $CDCl_3$ S59
24. Oxidation of **SIDipp-SO** with equimolar H_2O_2 in MeOD S60
25. Reaction of **SIDipp-SO** with aqueous HCl in MeOD S60
25. **SIDipp-SO** before and after addition of 1 eq. aqueous HCl in MeOD S61
IV. X-ray crystallography data and experimental description S62
V. Computational details S68
1. General remarks S68
2. Comparison of energetics of **Me-S** oxidation by acetic acid (**Ox**) and *m*-chloroperoxybenzoic acid (***m*CPBA**). S68
3. Optimized geometries (in Å) and energies (in E_h) S68
REFERENCES S98

I. Preparation of thioureas

1. Preparation of 1,3-dimethylimidazolidine-2-thione (SIMe-S)

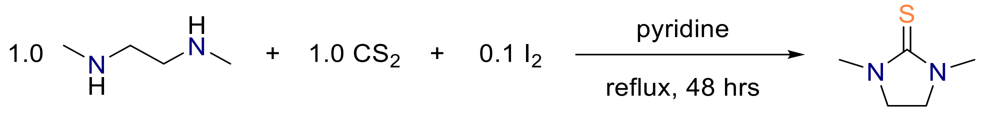

An experimental procedure devised by Denk *et.al.* was used.[1] To a cooled solution of 1.0 mL (0.819 g, 9.2 mmol) of commercially available diamine **C-Me** in pyridine (10 mL), 0.55 mL of carbon disulfide (0.700 g, 9.2 mmol) were added. The mixture was then refluxed for 24 hours. After cooling down, iodine was added (0.233 g, 0.9 mmol), and the mixture was kept refluxing for an additional 24 hours. The solvent was removed *in vacuo*, and the brown residue was dissolved in dichloromethane and washed with a solution of $Na_2S_2O_3$. The dichloromethane solution was concentrated under reduced pressure, and the resulting brown solid was recrystallized from ethanol to give thiourea **SIMe-S** in the form of brown needle-shaped crystals (0.32 g, 27%).
^{1}H–NMR (400 MHz, $CDCl_3$, 25 °C): δ=3.10 (s, 6H; CH_3), 3.50 ppm (s, 4H; CH_2).
^{13}C–NMR (100 MHz, $CDCl_3$, 25 °C): δ=35.2 (CH_3), 48.3 (CH_2), 183.5 ppm (CS).
EI-MS (75 eV): m/z (%): 130.0 (100) $[M]^+$, 115.0 (5) $[M-CH_3]^+$, 74 (20).
The analytical data match the literature description.[1]

2. Preparation of 1,3-di-*tert*-butylimidazolidine-2-thione (SI^{t}Bu-S)

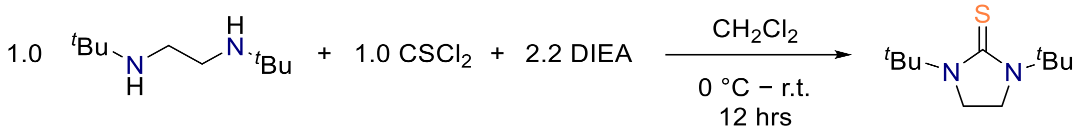

A modified version of the procedure devised by Li *et.al.* was used.[2] A solution of commercially available diamine **C-^{t}Bu** (11.75 g, 68.2 mmol) and 26 mL of diisopropylethylamine (19.39 g, 150.0 mmol) in 200 mL of dichloromethane was cooled down on an ice bath, and a solution of 5.22 mL of thiophosgene (7.84 g, 68.2 mmol) in 20 mL of dichloromethane was added dropwise over the course of 15 minutes. After addition, the mixture was allowed to keep stirring overnight and warm up to room temperature. After completion of the reaction, the mixture was washed three times with a saturated aqueous solution of NH_4Cl. The organic layer was dried and concentrated in vacuo to give a red semi-solid residue. The crude product was recrystallized from ethanol to give a brown crystalline solid, which was then additionally purified utilizing column chromatography with 25% chloroform in petroleum ether as eluent. Thiourea **SI^{t}Bu-S** was obtained as a pale-yellow crystalline solid (12.24 g, 84%).
^{1}H–NMR (400 MHz, $CDCl_3$, 25 °C): δ=1.56 (s, 18H; CH_3), 3.40 ppm (s, 4H; CH_2).
^{13}C–NMR (100 MHz, $CDCl_3$, 25 °C): δ=28.1 (CH_3), 44.5 (CH_2), 56.7 (*C*CH_3), 183.7 ppm (CS).
EI-MS (75 eV): m/z (%): 214.2 (75) $[M]^+$, 157.1 (40) $[M-C_4H_9]^+$, 143.1 (50) $[M-C_6H_{11}]^+$, 102.0 (100).
The analytical data match the literature description.[1]

3. Preparation of 1,3-diphenylimidazolidine-2-thione (SIPh-S)

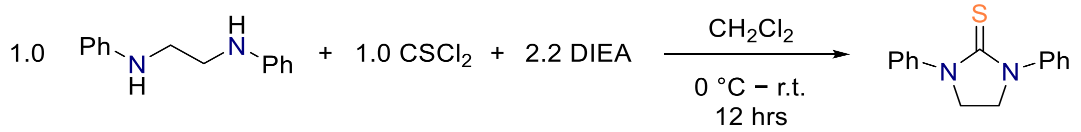

A modified version of the procedure devised by Li *et.al.* was used.[2] A solution of the commercially available diamine **C-Ph** (16.20 g, 76.3 mmol) and 29 mL of diisopropylethylamine (21.62 g, 167.3 mmol) in 200 mL of dichloromethane was cooled down on an ice bath, and a solution of 5.85 mL of thiophosgene (8.78 g, 76.4 mmol) in 20 mL of dichloromethane was added dropwise over the course of 15 minutes. After addition, the mixture was allowed to keep stirring overnight and warm up to room temperature. After completion of the reaction, the mixture was washed three times with a saturated aqueous solution of NH_4Cl. The organic layer was dried and concentrated in vacuo to give a brown solid residue. The crude product was recrystallized from ethanol to give a light brown crystalline solid, which was then additionally purified by washing with ethyl acetate. Thiourea **SIPh-S** was obtained as a flaky beige crystalline solid (14.21 g, 73%).
^{1}H–NMR (400 MHz, $CDCl_3$, 25 °C): δ=4.14 (s, 4H, CH_2), 7.24-7.28 (m, 2H, Ar-H), 7.39-7.44 (m, 4H, Ar-H), 7.54-7.57 ppm (m, 4H, Ar-H).
^{13}C–NMR (100 MHz, $CDCl_3$, 25 °C): 49.4 (CH_2), 125.5, 126.7, 128.9, 140.9, 181.4 ppm (C=S).
EI-MS (75 eV): m/z (%): 253.1 (100) $[M-H]^+$, 127.0 (5) $[M/2]^+$, 91.0 (15) $[C_7H_7]^+$, 77.0 (25) $[C_6H_5]^+$.
The analytical data match the literature description.[1, 3]

4. Preparation of *N,N′*-dimesitylethanediimine (B-Mes)

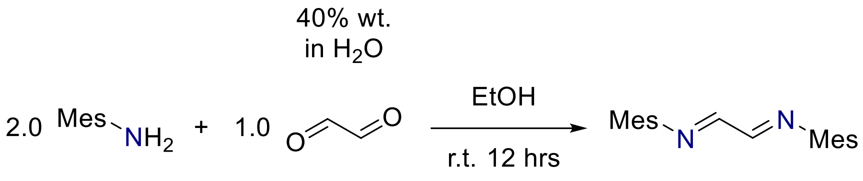

A-Mes **B-Mes**

A modified version of a procedure developed by Arguengo *et.al.* was used.[4] To a solution of 2.0 mL (1.97 g, 14.5 mmol) of aniline **A-Mes** in 50 mL of ethanol, 0.84 mL (40 wt.%, 1.05 g, 7.3 mmol) of aqueous glyoxal solution was added in one portion and left to stir overnight. The next day, a big amount of precipitate could be observed, and the yellow suspension was concentrated *in vacuo*. The residue was recrystallized from ethanol to give diimine **B-Mes** as bright yellow crystalline solid (1.61 g, 76%).
^{1}H–NMR (400 MHz, $CDCl_3$, 25 °C): δ=2.14 (s, 12H; CH_3), 2.28 (s, 6H; CH_3), 6.89 (s, 4H; Ar–H), 8.08 ppm (s, 2H; CH).
^{13}C–NMR (100 MHz, $CDCl_3$, 25 °C): δ=18.2 (*o*-CH_3), 20.8 (*p*-CH_3), 126.6, 129.0 (CH), 134.3, 147.5, 163.5 ppm (CH).
EI-MS (75 eV): m/z (%): 277.1 (100) $[M{-}CH_3]^+$, 146.1 (50) $[M/2]^+$, 131.0 (20), 119.1 (15), 91.0 (15) $[C_7H_7]^+$.
The analytical data match the literature description.[5]

5. Preparation of *N,N′*-dimesitylethanediamine (C-Mes)

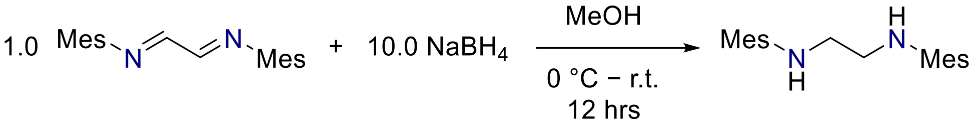

B-Mes **C-Mes**

A solution of 1.0 g (3.4 mmol) of diimine **B-Mes** in methanol was cooled down on an ice bath, and 1.3 g (34.2 mmol) of sodium hydroborate were added in small portions over the course of 5 minutes. The reaction was left stirring overnight, allowing it to warm up to room temperature. By completion of the reaction, most of the coloration had disappeared, and the solution was concentrated *in vacuo*. A saturated aqueous solution of NH_4Cl was added to the residue and extracted with dichloromethane three times. The combined organic layers were dried over anhydrous magnesium sulfate and concentrated *in vacuo* again to give diamine **C-Mes** as a pale-yellow liquid that solidified in the fridge (1.05 g, >99%). The product was used in following step without purification.
^{1}H–NMR (400 MHz, DMSO-d_6, 25 °C): δ=2.18 (s, 6H; CH_3), 2.22 (s, 12H; CH_3), 3.03 (s, 4H; CH_2), 3.82 (s, 2H; NH), 6.88 ppm (s, 4H; Ar–H).
^{13}C–NMR (100 MHz, DMSO-d_6, 25 °C): δ=18.7 (*o*-CH_3), 20.7 (*p*-CH_3), 48.9 (CH_2), 129.5, 129.8 (CH), 130.2, 144.2 ppm.
EI-MS (75 eV): m/z (%): 296.2 (15) $[M]^+$, 148.1 (100) $[M/2]^+$, 134 (15), 119.1 (15), 91.0 (15) $[C_7H_7]^+$.
The analytical data match the literature description.[6]

6. Preparation of 1,3-dimesitylimidazolidine-2-thione (SIMes-S)

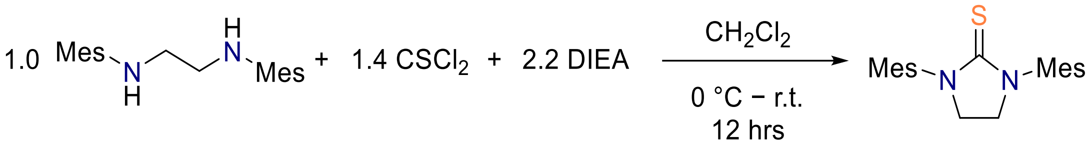

C-Mes **SIMes-S**

A modified version of a procedure devised by Li *et.al.* was used.[2] A solution of diamine **C-Mes** (3.75 g, 12.7 mmol) and 4.8 mL of diisopropylethylamine (2.2 eq., 3.60 g, 27.9 mmol) in 200 mL of dichloromethane was cooled down on an ice bath, and a solution of 1.0 mL of thiophosgene (1.0 eq., 1.45 g, 12.6 mmol) in 10 mL of dichloromethane was added dropwise over the course of 5 minutes. After addition, the mixture was allowed to keep stirring overnight and warm up to room temperature. An NMR aliquot revealed that only 70 mol.% of diamine **C-Mes** were converted into thiourea **SIMes-S**. Additional 0.4 mL of thiophosgene (0.4 eq., 0.59 g, 5.2 mmol) in 5 mL of dichloromethane were added dropwise at room temperature and the mixture was left stirring overnight. After completion of the reaction, the mixture was washed three times with a saturated aqueous solution of NH_4Cl. The organic layer was dried and concentrated *in vacuo* to give a brown solid residue. The crude product was purified by filtration on silica gel using ethyl acetate as an eluent, followed by recrystallization from ethanol, resulting in 2.6 g of thiourea **SIMes-S** as a pale beige crystalline solid. Additional 1.0 g of thiourea were recovered from the mother liquor in the form of a beige crystalline solid. The total yield of thiourea **SIMes-S** amounted to 3.60 g (84%).
^{1}H–NMR (400 MHz, $CDCl_3$, 25 °C): δ=2.28 (s, 6H; CH_3), 2.30 (s, 12H; CH_3), 3.98 (s, 4H; CH_2), 6.95 ppm (s, 4H; Ar–H).
^{13}C–NMR (100 MHz, $CDCl_3$, 25 °C): δ=17.8 (*o*-CH_3), 21.2 (*p*-CH_3), 47.7 (CH_2), 129.5 (CH), 134.6, 136.6, 138.3, 181.1 ppm (CS).
EI-MS (75 eV): m/z (%): 338.2 (75) $[M]^+$, 323.1 (100) $[M{-}CH_3]^+$, 305.2 (30) 187.1 (20), 146.1 (30), 91.1 (20) $[C_7H_7]^+$.
The analytical data match the literature description.[7]

7. Preparation of *N,N′*-bis(2,6-diisopropylphenyl)ethanediimine (B-Dipp)

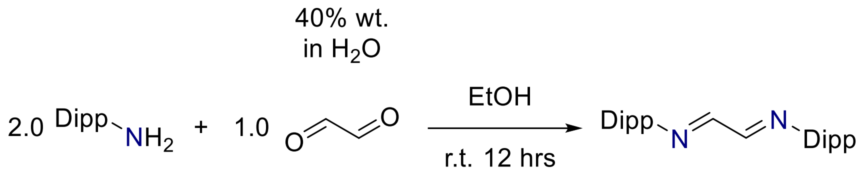

A-Dipp **B-Dipp**

A modified version of the procedure developed by Arguengo *et.al.* was used.[4] To a solution of 4.8 mL (4.51 g, 24.4 mmol) of aniline **A-Dipp** in 150 mL of ethanol, 1.5 mL (40 wt.%, 1.89 g, 12.7 mmol) of aqueous glyoxal solution was added in one portion and left to stir overnight. The next day, the yellow solution was concentrated *in vacuo* and the residue was recrystallized from ethanol to give diimine **B-Dipp** as a bright yellow crystalline solid (3.44 g, 76%).

^{1}H–NMR (400 MHz, $CDCl_3$, 25 °C): δ=1.20 (d, J=6.9 Hz, 24H; CH_3), 2.93 (hept, J=6.8 Hz, 4H; CH), 7.12-7.19 (m, 6H; Ar–H), 8.09 ppm (s, 2H; CH).

^{13}C–NMR (100 MHz, $CDCl_3$, 25 °C): δ=23.4 (CH_3), 28.1 (CH), 123.2 (CH), 125.1 (CH), 136.8, 148.1, 163.1 ppm (CH).

EI-MS (75 eV): m/z (%): 333.2 (100) $[M{-}C_3H_7]^+$, 188.1 (25) $[M/2]^+$, 174.1 (25), 146.1 (30), 132.1 (25) 91.1 (10) $[C_7H_7]^+$.

The analytical data match the literature description.[8]

8. Preparation of *N,N′*-bis(2,6-diisopropylphenyl)ethanediamine (C-Dipp)

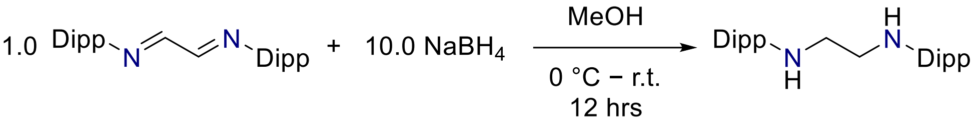

B-Dipp **C-Dipp**

A solution of 2.0 g (5.3 mmol) of diimine **B-Dipp** in methanol was cooled on an ice bath, and 2.0 g (52.8 mmol) of sodium hydroborate were added in small portions over the course of 15 minutes. The reaction was left stirring overnight, allowing it to warm up to room temperature. By completion of the reaction, most of the yellow color had disappeared, and the solution was concentrated *in vacuo*. A saturated aqueous solution of NH_4Cl was added to the residue and extracted with dichloromethane three times. The combined organic layers were dried with anhydrous magnesium sulfate and concentrated *in vacuo* to give diamine **C-Dipp** as a pale-yellow solid (2.10 g, >99%). The product was used in the following step without purification.

^{1}H–NMR (400 MHz, DMSO-d_6, 25 °C): δ=1.16 (d, J=6.8 Hz, 24H; CH_3), 3.03 (s, 4H; CH_2), 3.39 (hept, J=6.8 Hz, 4H; CH), 3.84 (s, 2H; NH) 6.95-7.05 ppm (m, 6H; Ar–H).

^{13}C–NMR (100 MHz, DMSO-d_6, 25 °C): δ=24.7 (CH_3), 27.3 (CH), 52.3 (CH_2), 123.6 (CH), 123.7 (CH), 142.8, 144.0 ppm.

EI-MS (75 eV): m/z (%): 380.3 (5) $[M]^+$, 190.2 (100) $[M/2]^+$, 175.1 (15), 160.1 (20).

The analytical data match the literature description.[9]

9. Preparation of 1,3-bis(2,6-diisopropylphenyl)imidazolidine-2-thione (SIDipp-S)

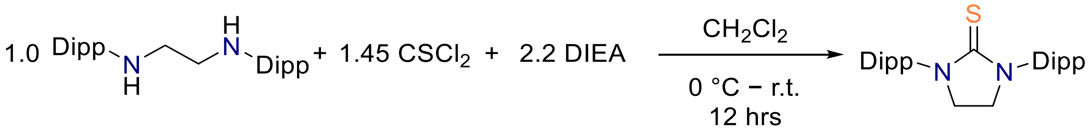

C-Dipp **SIDipp-S**

A modified version of the procedure devised by Li *et.al.* was used.[2] A solution of diamine **C-Dipp** (3.75 g, 9.9 mmol) and 3.8 mL of diisopropylethylamine (2.2 eq., 2.80 g, 21.7 mmol) in 200 mL of dichloromethane was cooled down on an ice bath, and a solution of 0.8 mL of thiophosgene (1.05 eq., 1.19 g, 10.4 mmol) in 10 mL of dichloromethane was added dropwise over the course of 5 minutes. After addition, the mixture was allowed to keep stirring overnight and warm up to room temperature. An NMR aliquot revealed that only 75 mol.% of diamine **C-Dipp** were converted into thiourea **SIDipp-S**. Additional 0.3 mL of thiophosgene (0.4 eq., 0.45 g, 4.0 mmol) in 5 mL of dichloromethane were added dropwise at room temperature and left stirring overnight. After completion of the reaction, the mixture was washed three times with a saturated aqueous solution of NH_4Cl. After completion of the reaction, the mixture was washed three times with a saturated aqueous solution of NH_4Cl. The organic layer was dried and concentrated *in vacuo* to give a brown solid residue. The crude product was purified by filtration on silica gel using ethyl acetate as an eluent, followed by recrystallization from ethanol, resulting in 2.76 g of thiourea **SIDipp-S** as an off-white crystalline solid. Additional 0.44 g of thiourea were recovered from mother liquor in form of a light brown crystalline solid. Total yield of thiourea **SIDipp-S** amounted to 3.20 g (77%).

^{1}H–NMR (400 MHz, $CDCl_3$, 25 °C): δ=1.30 (d, J=6.9 Hz, 12H; CH_3), 1.32 (d, J=6.9 Hz, 12H; CH_3), 3.06 (hept, J=6.9 Hz, 4H; CH), 4.01 (s, 4H; CH_2), 7.22 (d, J=7.7 Hz, 4H; Ar–H), 7.36 ppm (t, J=7.8 Hz, 2H; Ar–H).

^{13}C–NMR (100 MHz, $CDCl_3$, 25 °C): δ=24.4 (CH_3), 24.6 (CH_3), 29.1 (CH), 50.3 (CH_2), 124.4 (CH), 129.3 (CH), 134.9, 147.5, 184.4 ppm (CS).

ESI-MS: m/z (%): 421.35 $[M{+}H]^+$.

The analytical data match the literature description.[10]

10. Preparation of 1,3-dimethylimidazole-2-thione (IMe-S)

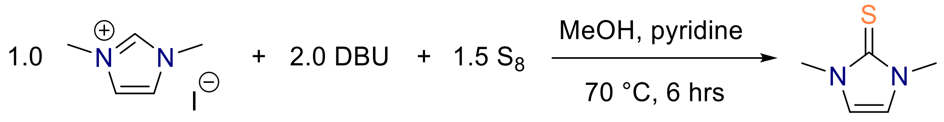

IMe-H⁺ **IMe-S**

A procedure devised by Karkhanis and Field was used.[3] In a mixture of 50 mL of methanol, 6 mL of pyridine, 6.2 mL of DBU (6.35 g, 41.7 mmol), 4.67 g (20.9 mmol) of commercially available imidazolium salt **IMe-H**$^+$ along with 1.0 g (31.3 mmol) of elemental sulfur were added, and then the magnetically stirred mixture was left heating at 70 °C for 6 hours. After cooling down, the solution was diluted with water and extracted with chloroform three times. Combined organic layers were washed with diluted hydrochloric acid, dried over anhydrous magnesium sulfate, and concentrated *in vacuo* to give 2.2 g of crude thiourea **IMe-S** as a brown solid. The compound was recrystallized from ethanol to give thiourea **IMe-S** in the form of light brown needle-shaped crystals (1.70 g, 63%).
^{1}H–NMR (400 MHz, $CDCl_3$, 25 °C): δ=3.55 (s, 6H; CH_3), 6.63 ppm (s, 2H; CH).
^{13}C–NMR (100 MHz, $CDCl_3$, 25 °C): δ=35.3 (CH_3), 117.6 (CH), 162.7 ppm (CS).
EI-MS (75 eV): m/z (%): 128.0 (100) $[M]^+$, 95 (15).
The analytical data match the literature description.[11]

11. Preparation of *N,N′*-di-*tert*-butylethanediimine (B-^{t}Bu)

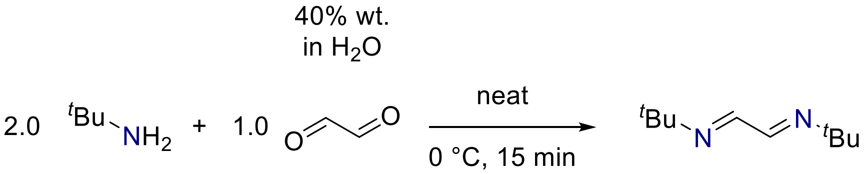

A-^{t}Bu **B-^{t}Bu**

A modified version of an experimental procedure devised by Caroccia *et.al.* was used.[12] Amine **A-^{t}Bu** in a quantity of 2.86 mL (2.0 g, 27 mmol) was added dropwise to an aqueous solution of 1.57 mL (40 wt.%, 1.98 g, 14 mmol) glyoxal cooled down on an ice bath. Within minutes, enough solid had precipitated to make stirring impossible. The mixture was left on the ice bath for an additional 15 minutes, and then the solid was filtered off and left in the desiccator with calcium chloride at 150 mbar to remove traces of water. The yellow residue was then purified by means of sublimation at 20 mbar, resulting in diimine **B-^{t}Bu** as an off-white crystalline solid (1.85 g, 78%).
^{1}H–NMR (400 MHz, $CDCl_3$, 25 °C): δ=1.23 (s, 18H; CH_3), 7.91 ppm (s, 2H; CH).
^{13}C–NMR (100 MHz, $CDCl_3$, 25 °C): δ=29.4 (CH_3), 58.2 (CCH_3), 157.9 ppm (CH=N).
EI-MS (75 eV): m/z (%): 153 (5) $[M{-}CH_3]^+$, 141 (15), 112 (30), 97 (30), 57 (100) $[C_4H_9]^+$.
The analytical data match the literature description.[13]

12. Preparation of 1,3-di-*tert*-butylimidazolium chloride (I^{t}Bu-H$^+$)

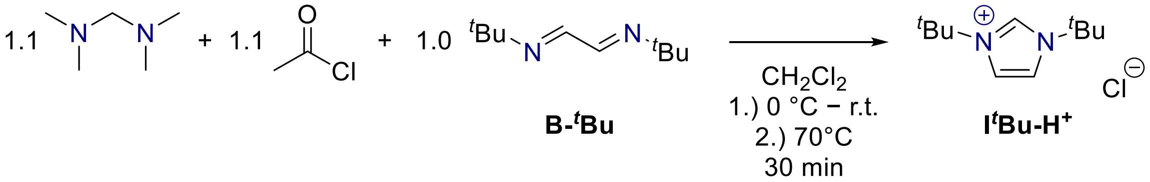

A modified experimental procedure devised by Mistryukov was used.[14] To a cooled on an ice bath solution of 0.76 mL (0.56 g, 5.6 mmol) of bis(dimethylamino)methane in 5 mL of dichloroethane, 0.4 mL (0.44 g, 5.6 mmol) of acetyl chloride was added. After a few minutes, a solution of 0.86 g (5.1 mmol) of diimine **B-^{t}Bu** in a small amount of dichloromethane was added. The ice bath was removed, and then replaced by an oil bath. The reacting mixture was left stirring for 30 minutes at 70 °C, then it was cooled down and concentrated *in vacuo* to give an oily orange residue. It was washed with ethyl acetate to remove the *N,N*-dimethylacetamide impurity and the crude product was filtered off and dried *in vacuo*. The crude product was purified by recrystallization from CH_2Cl_2/EA to give the imidazolium salt **I^{t}Bu-H$^+$** as an off-white powder (0.66 g, 60%). The product contained impurities, which were identified as *tert*-butylamine and dimethylamine hydrochlorides. The product was deemed pure enough for the next step of synthesis.
^{1}H–NMR (400 MHz, DMSO-d_6, 25 °C): δ=1.62 (s, 18H; CH_3), 8.09 (d, J=1.7 Hz, 2H; CH), 9.15 ppm (t, J=1.7 Hz, 1H; CH).
^{13}C–NMR (100 MHz, DMSO-d_6, 25 °C): δ=29.6 (CH_3), 60.1 (CCH_3), 120.9 (CH), 132.8 ppm (CH).
ESI-MS: m/z (%): 181.09 $[Cat]^+$.
The analytical data match the literature description.[15]

13. Preparation of 1,3-di-*tert*-butylimidazole-2-thione (I^{t}Bu-S)

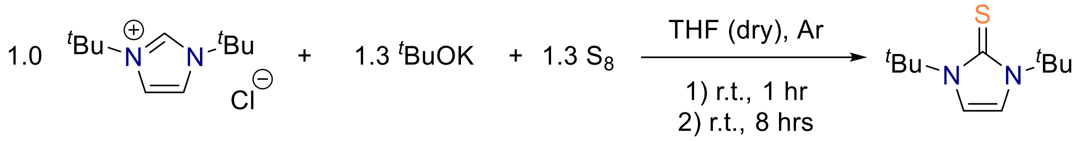

I^{t}Bu-H$^+$ **I^{t}Bu-S**

A modified version of a procedure devised by Ruamps *et.al.* was used.[16] Into an oven dried Schlenk flask flushed with argon, 0.64 g (3.0 mmol) of imidazolium salt **I^{t}Bu-H$^+$** were added, and suspended in 10 mL of dry THF, then 0.43 g (3.8 mmol) of potassium *tert*-butoxide were added. The mixture was left to stir for an hour to achieve carbene liberation. After that, 0.12 g (3.8 mmol) of elemental sulfur were added. The mixture was left stirring overnight. After completion of the reaction, the suspension was filtered through a pad of Celite and concentrated in vacuo. The dark red residue was purified by means of filtration on silica gel using 25% ethyl acetate in *n*-hexane as an eluent. The collected solution was concentrated *in vacuo* to give thiourea **I^{t}Bu-S** as a yellow crystalline solid (0.44 g, 71%).
The purity of the product was deemed sufficient for the planned experiments.
^{1}H–NMR (400 MHz, $CDCl_3$, 25 °C): δ=1.80 (s, 18H; CH_3), 6.78 ppm (s, 2H; CH).
^{13}C–NMR (100 MHz, $CDCl_3$, 25 °C): δ=28.3 (CH_3), 59.4 (CCH_3), 113.5 (CH), 160.5 ppm (CS, obtained from HMBC).
IR (ATR): $\tilde{\nu}$=1200 (vs, C=S stretch, composite), 1357 (vs) cm^{-1} (C=S stretch, composite).
EI-MS (75 eV): m/z (%): 212.1 (25) $[M]^+$, 156.0 (10) $[M-C_4H_8]^+$, 100.0 (100) $[M-C_8H_{16}]^+$, 57.1 (10) $[C_4H_9]^+$.
HRMS (ESI): m/z calcd for $C_{11}H_{21}N_2S^+$: 213.14200 $[M+1]^+$; found: 213.14200.

14. Preparation of 1,3-dimesitylimidazolium chloride (IMes-H$^+$)

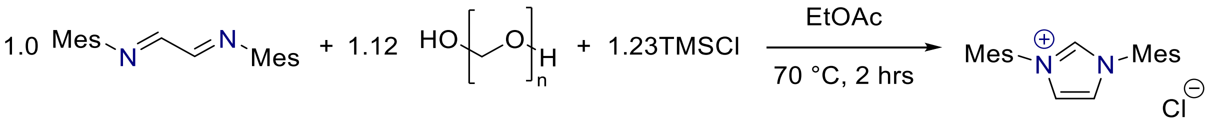

B-Mes **IMes-H$^+$**

An experimental procedure devised by Hintermann was used.[17] Diimine **B-Mes** in a quantity of 8.00 g (27.2 mmol) and 0.93 g (31 mmol) of paraformaldehyde were added to 300 mL of ethyl acetate. Under vigorous stirring, 4.10 mL (3.60 g, 33.7 mmol) of trimethylsilyl chloride dissolved in 5 mL of ethyl acetate were added dropwise to the mixture and then heating was maintained at 70 °C for two hours. By completion of the reaction, a suspension was formed. The suspension was cooled to −10 °C, filtered through a frit, and the precipitate was washed with ethyl acetate and diethyl ether. The solid was collected and dried *in vacuo*, to give imidazolium salt **IMes-H$^+$** as a pale-yellow powder (6.86 g, 73%).
^{1}H–NMR (400 MHz, DMSO-d_6, 25 °C): δ=2.13 (s, 12H; CH_3), 2.36 (s, 6H; CH_3), 7.21 (s, 4H; Ar–H), 8.31 (d, J=1.4 Hz, 2H; CH), 9.78 ppm (t, J=1.4 Hz, 1H; CH).
^{13}C–NMR (100 MHz, DMSO-d_6, 25 °C): δ=17.4 (*o*-CH_3), 21.1 (*p*-CH_3), 125.3 (CH), 129.8 (CH), 131.5, 134.8, 139.0 (CH), 141.0 ppm.
ESI-MS: m/z (%): 305.26 $[Cat]^+$.
The analytical data match the literature description.[4]

15. Preparation of 1,3-dimesitylimidazole-2-thione (IMes-S)

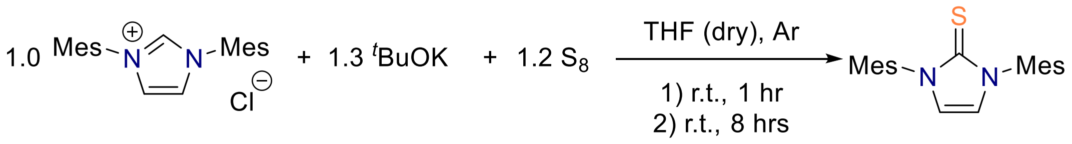

IMes-H$^+$ **IMes-S**

A modified version of a procedure devised by Ruamps *et.al.* was used.[16] Into an oven dried Schlenk flask flushed with argon, 300 mg (0.89 mmol) imidazolium salt **IMes-H$^+$** were added. First, 20 mL of dry THF were added and then, 130 mg (1.16 mmol) of potassium *tert*-butoxide were added. The suspension quickly assumed a yellow color and was left stirring for an extra hour until most of the carbene was liberated. Then 37 mg (1.16 mmol) of elemental sulfur were added, and the fine yellow suspension quickly became light brown. The mixture was left stirring overnight. The next day, the mixture was concentrated *in vacuo*. In order to remove remaining inorganic compounds, the residue was redissolved in chloroform, filtered through Celite, and evaporated again. The crude product was recrystallized from ethanol to obtain thiourea **IMes-S** as a pale yellow crystalline solid (201 mg, 68%).
^{1}H–NMR (400 MHz, $CDCl_3$, 25 °C): δ=2.12 (s, 12H; CH_3), 2.32 (s, 6H; CH_3), 6.77 (s, 2H; CH), 6.99 ppm (s, 4H; Ar–H).
^{13}C–NMR (100 MHz, $CDCl_3$, 25 °C): δ=18.0 (*o*-CH_3), 21.3 (*p*-CH_3), 118.1 (CH), 129.3 (CH), 133.7, 135.7, 139.3, 163.4 ppm (CS).
EI-MS (75 eV): m/z (%): 336.1 (55) $[M]^+$, 321.1 (33) $[M-CH_3]^+$, 303.2 (100), 185.1 (20), 119.2 (20), 91.0 (20) $[C_7H_7]^+$.
The analytical data match the literature description.[16]

16. Preparation of 1,3-bis(2,6-diisopropylphenyl)imidazolium chloride (IDipp-H+)

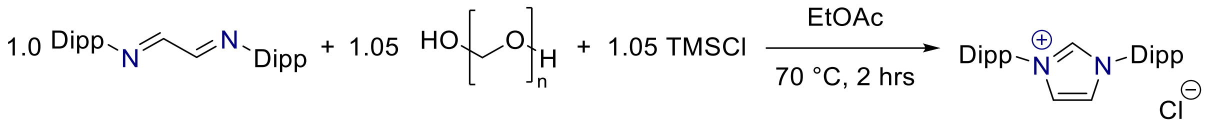

B-Dipp **IDipp-H+**

An experimental procedure devised by Hintermann was used.[17] Diimine **B-Dipp** in a quantity of 2.0 g (5.3 mmol) and 0.16 g (5.6 mmol) of paraformaldehyde were added to 150 mL of ethyl acetate and then, 0.71 mL (0.61 g, 5.6 mmol) of trimethylsilyl chloride dissolved in 5 mL of ethyl acetate were added dropwise to the mixture. Thereafter, the reaction mixture was kept at 70 °C for two hours. When the reaction was complete, a suspension was formed. The suspension was cooled to −10 °C, filtered through a frit, and the precipitate was washed with ethyl acetate and diethyl ether. The solid was collected and dried in an oil pump vacuum. The product was found to contain ethyl acetate, so it was then dissolved in acetonitrile and dried *in vacuo* again to give imidazolium salt **IDipp-H+** as pale-yellow powder (1.94 g, 87%).
^{1}H–NMR (400 MHz, DMSO-d$_6$, 25 °C): δ=1.19 (d, J=6.9 Hz, 12H; CH_3), 1.28 (d, J=6.9 Hz, 12H; CH_3), 2.37 (hept, J=6.8 Hz, 4H; CH), 7.54 (d, J=7.8 Hz, 4H; Ar–H), 7.70 (t, J=7.8 Hz. 2H; Ar–H), 8.60 (d, J=1.4 Hz, 2H; CH), 10.43 ppm (t, J=1.4 Hz, 1H; CH).
^{13}C–NMR (100 MHz, DMSO-d$_6$, 25 °C): δ=23.6 (CH_3), 24.6 (CH_3), 29.1 (CH), 125.1 (CH), 126.7 (CH), 130.5, 132.3 (CH), 140.0 (CH), 145.3 ppm.
ESI-MS: m/z (%): 389.41 [Cat]$^+$.
The analytical data match the literature description.[4]

17. Preparation of 1,3-bis(2,6-diisopropylphenyl)imidazole-2-thione (IDipp-S)

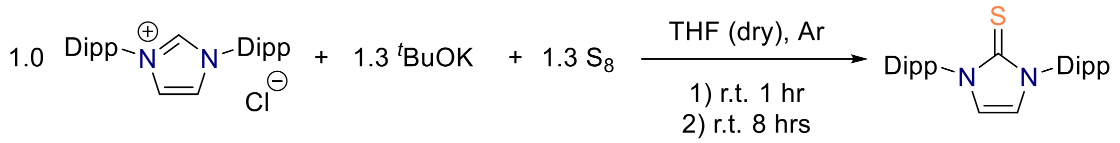

IDipp-H+ **IDipp-S**

A modified version of the procedure devised by Ruamps *et.al.* was used.[16] Into an oven dried Schlenk flask flushed with argon, 300 mg (0.71 mmol) imidazolium salt **IDipp-H+** were added. First, 20 mL of dry THF were added and then, 104 mg (0.93 mmol) of potassium *tert*-butoxide were added. The suspension quickly assumed a pink color and was kept stirring for an extra hour. Then, 30 mg (0.93 mmol) of elemental sulfur were added, and the fine pink suspension quickly became dark brown. The mixture was left stirring overnight. On the next day, the mixture was concentrated *in vacuo*. In order to remove the remaining inorganic compounds, the residue was redissolved in chloroform, filtered through Celite, and evaporated again. The crude product was recrystallized from ethanol to obtain thiourea **IDipp-S** as off-white flaky crystals (158 mg, 53%).
^{1}H–NMR (400 MHz, $CDCl_3$, 25 °C): δ=1.19 (d, J=6.9 Hz, 12H; CH_3), 1.29 (d, J=6.9 Hz, 12H; CH_3), 2.73 (hept, J=6.9 Hz, 4H; CH), 6.82 (s, 2H; CH), 7.27 (d, J=7.8 Hz, 4H; Ar–H), 7.44 ppm (t, J=7.8 Hz, 2H; Ar–H).
^{13}C–NMR (100 MHz, $CDCl_3$, 25 °C): δ=23.4 (CH_3), 24.2 (CH_3), 28.9 (CH), 119.0 (CH), 124.2 (CH), 130.1 (CH), 133.8, 146.5, 167.1 ppm (CS).
ESI-MS: m/z (%): 421.36 [*M*+H]$^+$.
The analytical data match the literature description.[18]

II. Oxidation of thioureas.

1. General procedure of NMR-scale screening of thioureas

To a weighed amount (2-5 mg) of thiourea **(S)IR-S** in an NMR tube, 0.4 mL of deuterated solvent ($CDCl_3$ or CD_3CN or CD_3OD*) were added. The NMR tube was shaken until the thiourea was dissolved, so that a clean ^{1}H-NMR spectrum could be measured. Then, a defined amount (0.5, 1.0 or 3.0 eq.) of oxidant (aqueous H_2O_2, or *m*CPBA dissolved in 0.1 mL of corresponding deuterated solvent) was added to the NMR tube, which was shaken again after addition to guarantee good mixing. A ^{1}H-NMR spectrum was measured, usually within 10 minutes after addition of the oxidant. The NMR signals were identified based on comparison with the clean starting compound spectra and literature data of known products.

2. General procedure of NMR-scale incremental oxidation of selected thioureas

Prior to performing the oxidation reactions, the purchased *m*CPBA was dried in an oil pump vacuum. The purity of the dried mCPBA was estimated to be 90%. A weighed amount (5-12 mg) of the corresponding thiourea (a: **IMe-S**; b: **SItBu-S**, **SIMes-S**, **SIDipp-S**) was dissolved in 0.3 mL of deuterated solvent (a: $CDCl_3$; b: CD_3OD*) in an NMR tube, and a clean ^{1}H-NMR spectrum was measured. Then, increments of 0.5 eq. of dry *m*CPBA dissolved in 0.1 mL of the corresponding solvent were added. On each addition, the NMR tube was shaken to guarantee good mixing, and a ^{1}H-NMR spectrum was measured. The time it took from addition of the oxidant to acquisition of a spectrum was approximately 10 minutes. The NMR signals were compared to the known chemical shifts of the starting compounds and literature data of known products. The molar fraction of starting compounds and

products was estimated from the ratio of the integrals of the relevant characteristic NMR signals over their sum and plotted versus the number of *m*CPBA equivalents that were added to the mixture in total (Figure S1).
**To assist the dissolution of aryl-substituted thioureas in CD_3OD, additional 0.1 mL of $CDCl_3$ was added.*

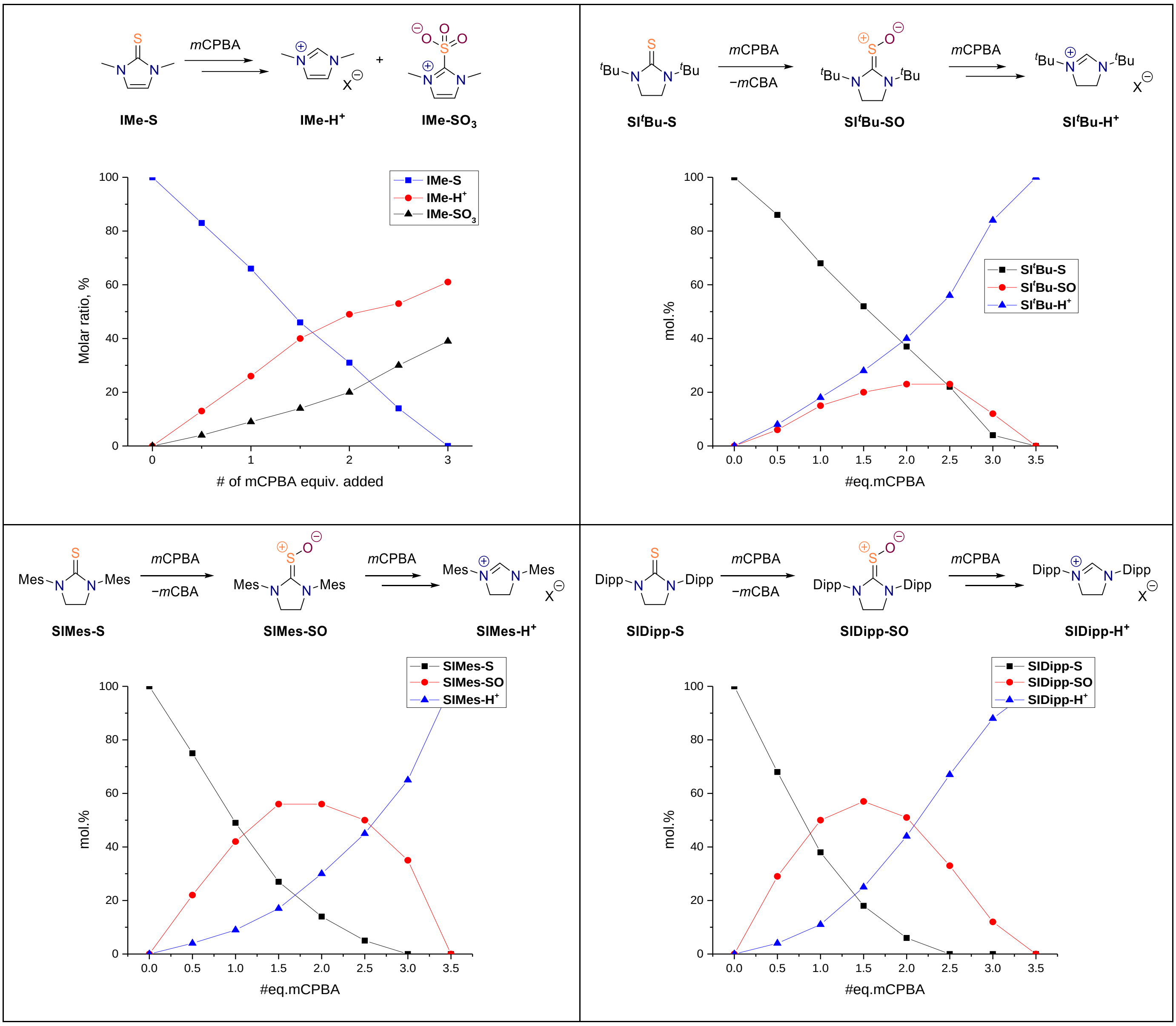

Figure S1. NMR-scale screening of **IMe-S** (a)), **SI^{t}Bu-S** (b)), **SIMes-S** (c)), **SIDipp-S** (d)) oxidation by 0.5 eq increments of mCPBA in methanol-d4. Molar ratios relate to integrals under signals of corresponding CH_3 (a)) or CH_2 (b)-d)) protons in partaking compounds.

3. Oxidation of 1,3-dimethylimidazol-2-thione (IMe-S)

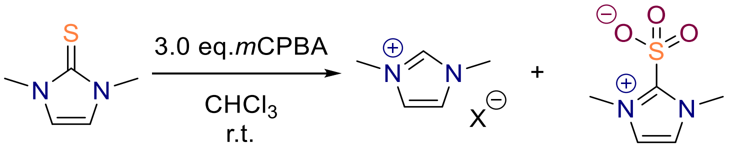

IMe-S **IMe-H$^+$** **IMe-SO$_3$**

To a solution of thiourea **IMe-S** (97 mg, 0.75 mmol) in $CHCl_3$, dried *m*CPBA (assumed 90% pure, 434 mg, 2.26 mmol) was added in portions. The solution briefly became yellow, and then, within seconds, the coloration disappeared. A solid precipitated from the solution when diluting it with diethyl ether. The solid was washed with $CHCl_3$, dried *in vacuo*, and dissolved in CD_3CN. The ^{1}H-NMR revealed a mixture of imidazolium salt **IMe-H$^+$** and trioxide **IMe-SO$_3$** in an approximate molar ration of 1:1. The isolation by means of reversed-phase chromatography was impossible due to the extremely close retention factors of the trioxide and respective imidazolium salt.
^{1}H–NMR (400 MHz, CD_3CN, 25 °C): δ=4.04 (s, 6H, CH_3), 7.28 ppm (s, 2H, CH).
^{13}C–NMR (100 MHz, CD_3CN, 25 °C, obtained from 2D-NMRs): δ=36.7 (CH_3), 123.2 (CH), 147.0 ppm (CSO_3).

HRMS (ESI): m/z calcd. for $C_5H_8N_2NaO_3S^+$: 199.01478 [M+Na]$^+$; found: 199.01514; m/z calcd. for $C_5H_9N_2O_3S^+$: 177.03284 [M+H]$^+$; found: 177.03316.

4. Oxidation of 1,3-di-*tert*-butylimidazolidine-2-thione (SI^{t}Bu-S)

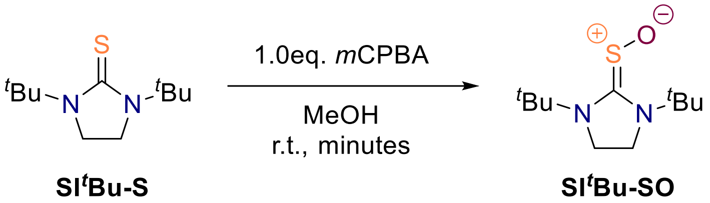

Into a 10 mL flask, 11.1 mg (0.05 mmol) of **SI^{t}Bu-S** were added and dissolved in 1.0 mL of methanol. Then, a solution of 11.2 mg (0.05 mmol, 77% pur.) of *m*CPBA in 0.1 mL of methanol was added at room temperature. The mixture was allowed to stir for 2 minutes, and then, it was concentrated *in vacuo*. An aliquot of the reaction mixture was analyzed by NMR spectroscopy. According to ^{1}H-NMR, the residue contained 15 mol.% of corresponding thiourea *S*-oxide. Within a day, the signals assigned to **SI^{t}Bu-SO** were absent from the NMR spectra. Thus, the compound was deemed too unstable to be isolated through reversed-phase chromatography.
^{1}H–NMR (400 MHz, methanol-d$_4$, 25 °C): δ=1.55 (s, 18H, CH_3), 3.76 ppm (s, 4H, CH_2).
^{13}C–NMR (100 MHz, methanol-d$_4$, 25 °C, obtained from HMBC): δ=30.3 (CH_3), 48.4 (CH_2), 60.2 (CCH$_3$), 182.4 ppm (CSO)
HRMS (ESI): m/z calcd for $C_{11}H_{23}N_2OS^+$: 231.15256 [M+H]$^+$; found: 231.15273.

5. Preparation of SIMes-SO

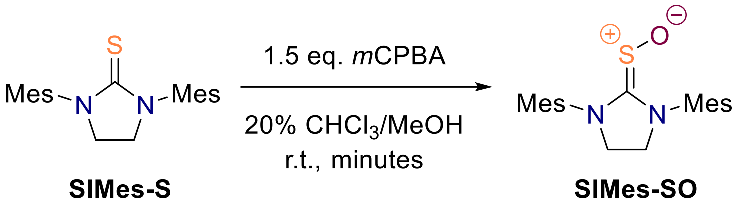

Into a 250 mL round-bottom flask, 250 mg (0.73 mmol) of **SIMes-S** were added and dissolved in a mixture of 20 mL of chloroform and 80 mL of methanol. Then, a solution of 250 mg (1.12 mmol, 77% pur.) of *m*CPBA in 2 mL of methanol was added dropwise to the thiourea solution at room temperature. The mixture was allowed to stir for 2 minutes. Then, 10 mL of ethanol were added to the solution,* and it was concentrated *in vacuo*. The ^{1}H-NMR spectrum of an aliquot revealed a 59% conversion (usually ranging between 50 and 55%) of thiourea **SIMes-S** to the desired *S*-oxide. The residue was dissolved in methanol, deposited on 12.0 g of silica, and evaporated. The silica was then packed into a pre-column and the desired thiourea *S*-oxide was isolated by means of reversed-phase medium pressure liquid chromatography on a PF-15C18HQ-F0025 column. The product can be identified by an absorption feature at 300 nm in the UV/visible absorption spectrum, where significant absorption is not observed for any other compound in the mixture. After concentration *in vacuo*, **SIMes-SO** was obtained as a pale-yellow powder (93 mg, 35%).
^{1}H–NMR (400 MHz, methanol-d$_4$, 25 °C): δ=2.31 (s, 6H, CH_3), 2.43 (s, 12H, CH_3), 4.19 (s, 4H, CH_2), 6.98 ppm (s, 4H, Ar-H).
^{13}C–NMR (100 MHz, methanol-d$_4$, 25 °C): δ=18.7 (*o*-CH$_3$), 22.0 (*p*-CH$_3$), 52.9 (CH_2), 131.3 (Ar-H), 133.7 (obtained from HMBC), 137.9, 141.2, 182.2 ppm (CSO, obtained from HMBC).
IR (ATR): $\tilde{\nu}$=924 (vs, S−O stretch, composite), 947 (vs, S−O stretch, composite), 973 cm^{-1} (s, S−O stretch, composite).
HRMS (ESI): m/z calcd. for $C_{21}H_{27}N_2OS^+$: 355.18386 [M+H]$^+$; found: 355.18423.
Our analytical data are in good agreement with the NMR-data reported in the literature, which were measured in CD_2Cl_2.[19]

We found that the addition of an alcohol with a higher boiling point enhanced stability of the S-oxide during evaporation. Without ethanol, fraction of* ***SIMes-SO* *can drop to 40 mol.%.*

6. Preparation of SIDipp-SO

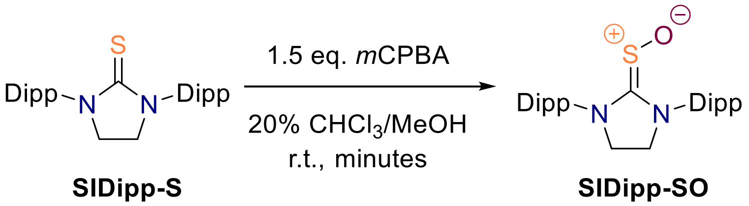

Into a 100 mL round-bottom flask, 295 mg (0.70 mmol) of **SIDipp-S** were added and dissolved in 15 mL of chloroform and 60 mL of methanol. Then, a solution of 235 mg (1.05 mmol, 77% pur.) of *m*CPBA in 2 mL of methanol was added dropwise at room temperature. The mixture was allowed to stir for 2 minutes followed by concentration *in vacuo*. The ^{1}H-NMR spectrum of an aliquot

revealed a 55% conversion (commonly ranging from 54% to 58%) of thiourea **SIDipp-S** to the corresponding *S*-oxide. The residue was dissolved in methanol, deposited on 4.0 g of silica, and the solvent was evaporated. The silica was then packed into a pre-column and the desired thiourea *S*-oxide was isolated by means of reversed-phase chromatography on a PF-15C18HQ-F0012 column. The product can be identified by an absorption feature at 300 nm in the UV/visible spectrum, where no substantial absorption is observed for any other compound in the mixture. After concentration *in vacuo,* **SIDipp-SO** was obtained as an off-white solid (122 mg, 40%).

^{1}H–NMR (400 MHz, methanol-d$_4$, 25 °C): δ=1.38 (d, J=6.9 Hz, 12H, CH_3), 1.43 (d, J=6.9 Hz, 12H, CH_3), 3.27 (hept, J=6.9 Hz, 4H, CH), 4.19 (s, 4H, CH_2), 7.27 (d, J=7.7 Hz, 4H, Ar-H), 7.41 ppm (t, J=7.7 Hz, 2H, Ar-H).

^{13}C–NMR (partial, 100 MHz, methanol-d$_4$, 25 °C): δ=25.3 (CH_3), 26.0 (CH_3), 31.2 (CH), 55.5 (CH_2), 126.3 (Ar-H), 132.0 (Ar-H), 148.8, 185.1 ppm (CSO, obtained from HMBC).

IR (ATR): $\tilde{\nu}$=916 (s, S−O stretch, composite), 936 (vs, S−O stretch, composite), 973 cm^{-1} (s, S−O stretch, composite).

HRMS (ESI): m/z calcd for $C_{27}H_{39}N_2OS^+$: 439.27776 [*M*+H]$^+$; found: 439.27805.

Note: Valid elemental analysis data could not be obtained due to admixture of colloidal reversed-phase silica in the isolated product, which resulted in an extremely increased mass fraction of Carbon (found 83.66% instead of calcd. 73.96%), and the sum of CHNS fractions totaled to over 100%.

According to ^{1}H-NMR, even after drying under oil pump vacuum, the solid contained 25 mol.% of methanol, amounting to 2.5 wt.% content of methanol.

III. Spectral data

1. 1,3-Dimethylimidazolidine-2-thione (SIMe-S)

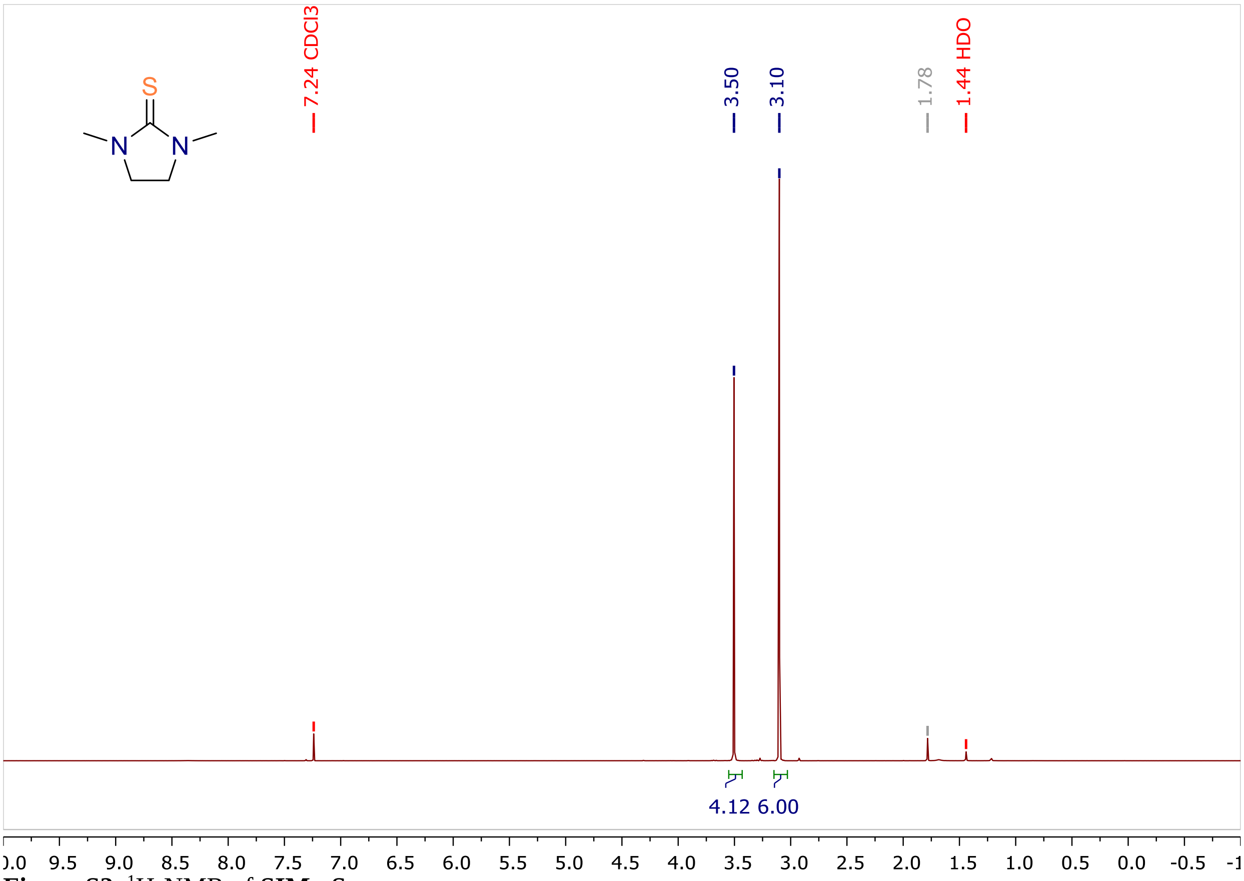

Figure S2. ^{1}H-NMR of **SIMe-S**.

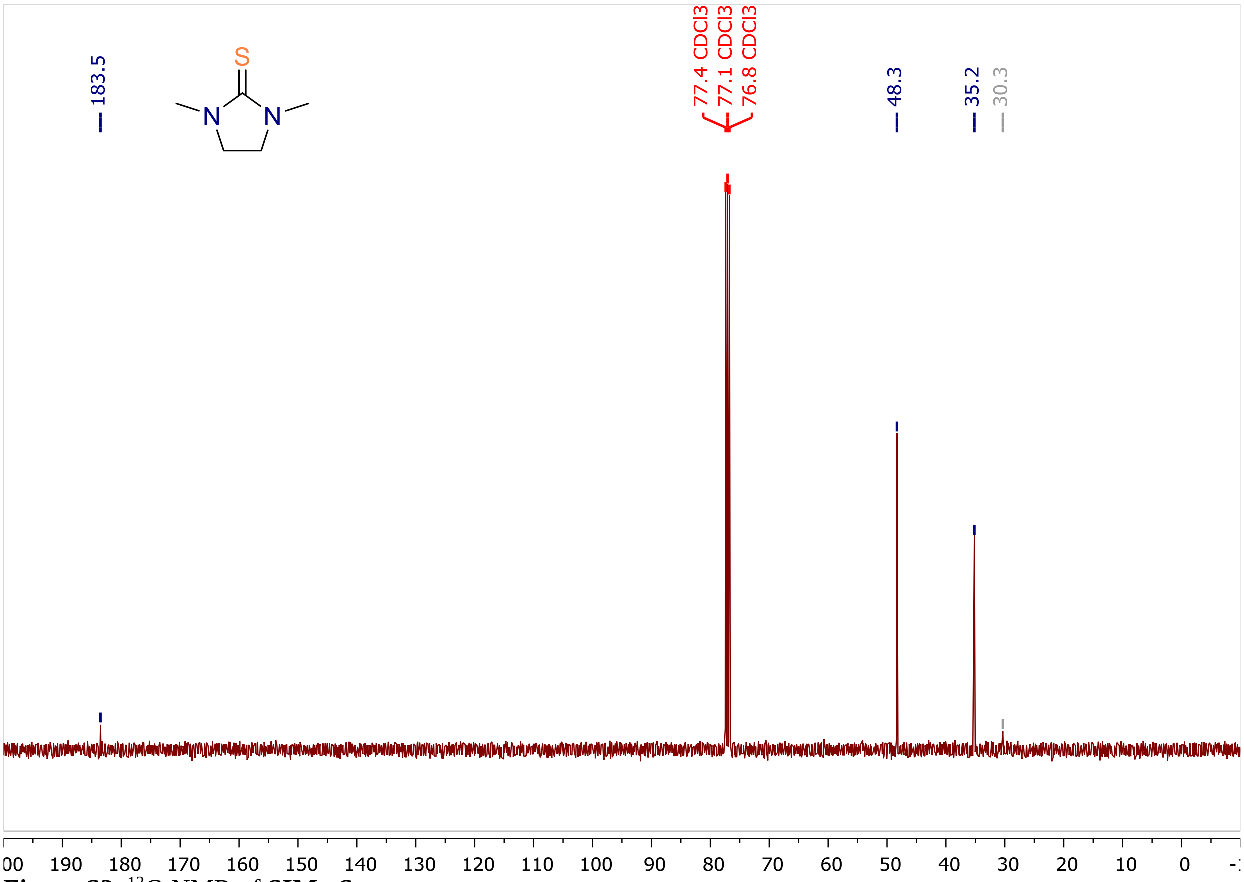

Figure S3. ^{13}C-NMR of **SIMe-S**.

Chromatograms

Fragmentor Voltage **Collision Energy** 0 **Ionization Mode** EI

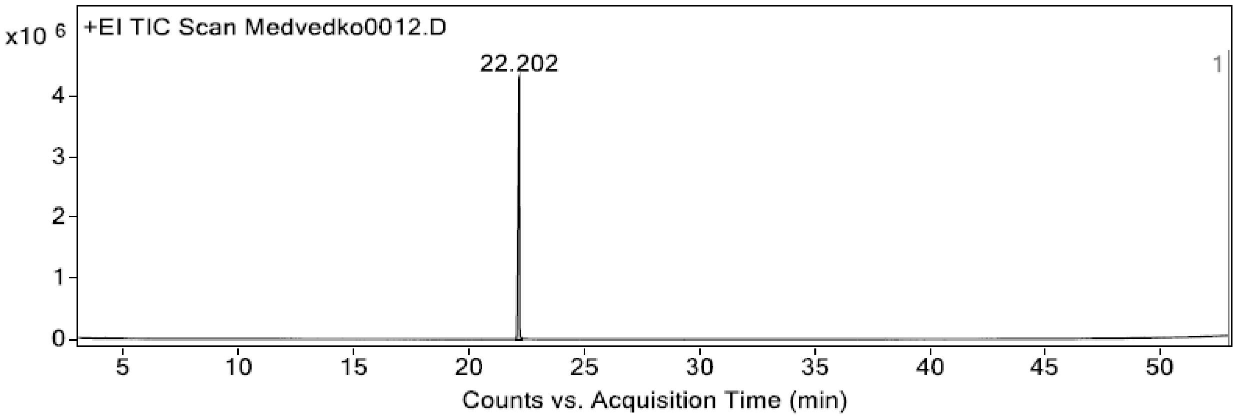

Integration Peak List

Peak	Start	RT	End	Height	Area	AreaSumPercent
1	22.05	22.202	22.313	4312718.22	19703073.53	100

Spectra

Spectrum Source **Collision Energy** **Ionization Mode**
Peak (1) in "+ TIC Scan" 0 EI

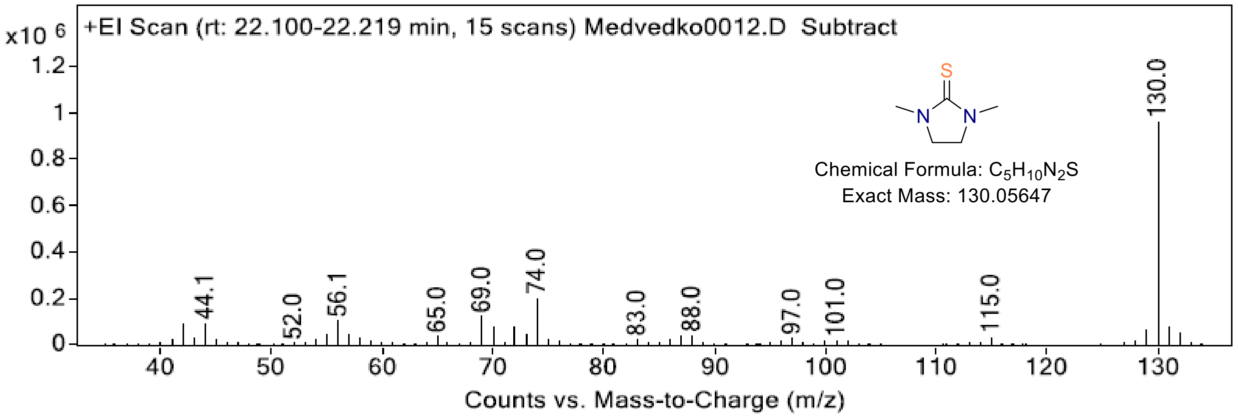

Figure S4. GC-MS of **SIMe-S**.

2. 1,3-Di-*tert*-butylimidazolidine-2-thione (SI^{t}Bu-S)

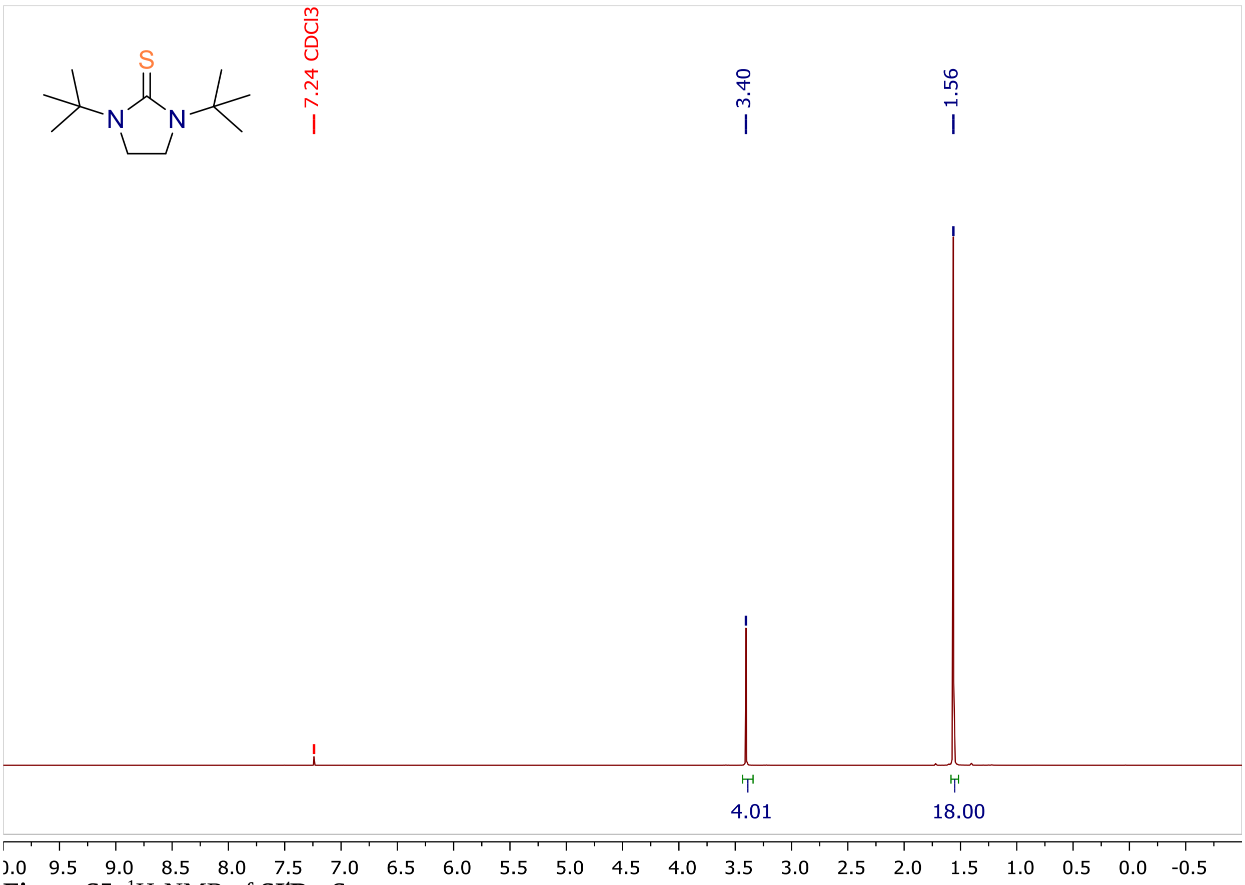

Figure S5. ^{1}H-NMR of **SI^{t}Bu-S**.

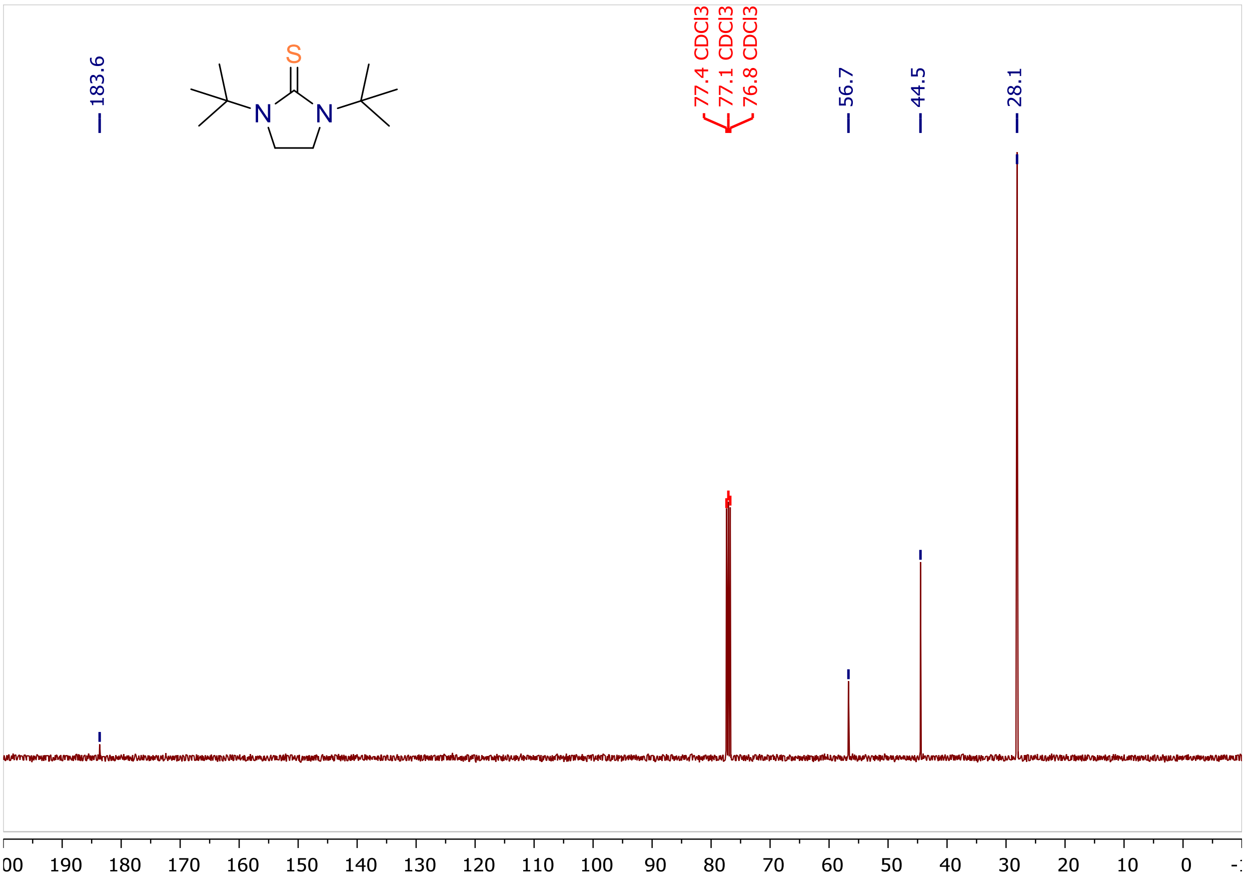

Figure S6. ^{13}C-NMR of **SI^{t}Bu-S**.

Chromatograms

Fragmentor Voltage **Collision Energy** 0 **Ionization Mode** EI

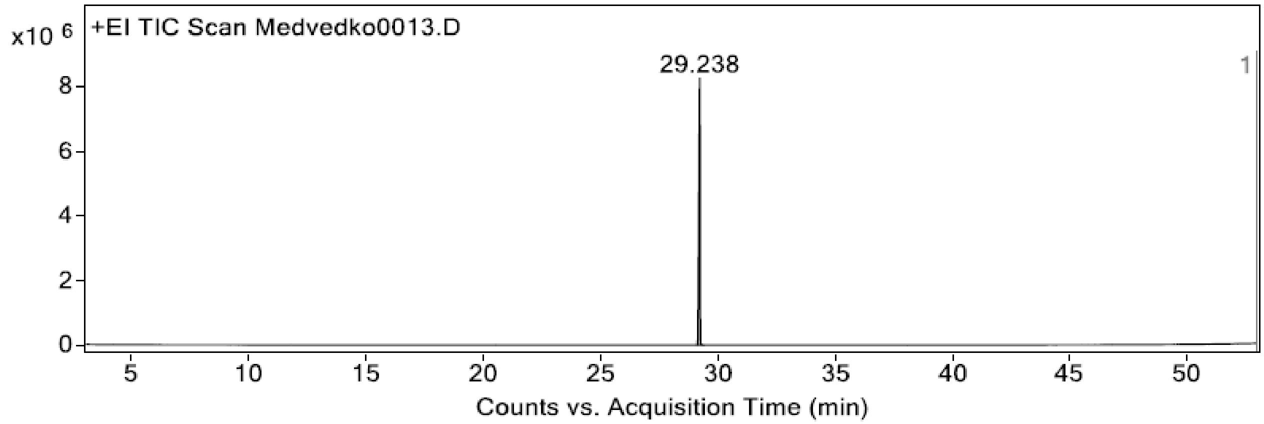

Integration Peak List

Peak	Start	RT	End	Height	Area	AreaSumPercent
1	29.111	29.238	29.392	8277866.63	31349231.87	100

Spectra

Spectrum Source	Collision Energy	Ionization Mode
Peak (1) in "+ TIC Scan"	0	EI

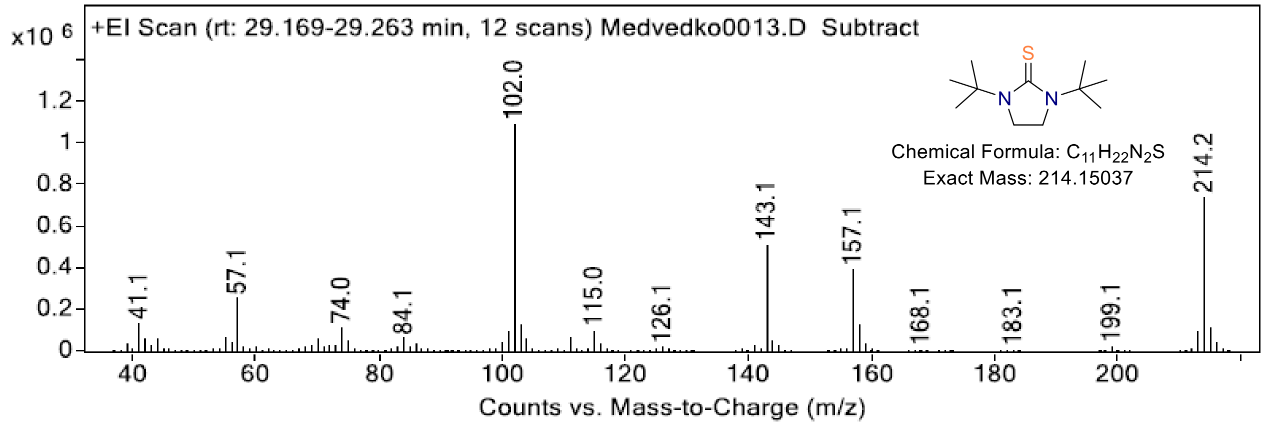

Figure S7. GC-MS of **SI^{t}Bu-S**.

3. 1,3-Diphenylimidazolidine-2-thione (SIPh-S)

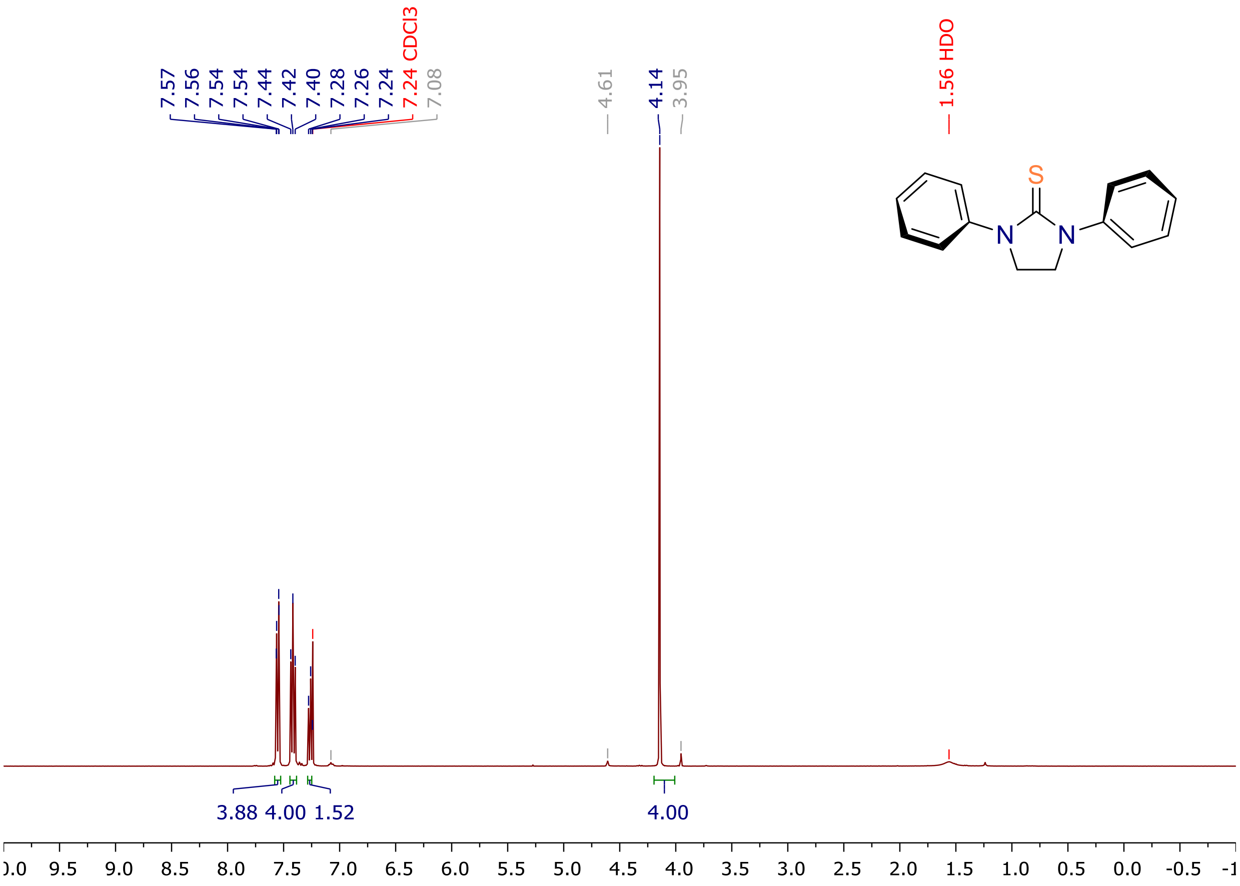

Figure S8. ^{1}H-NMR of **SIPh-S**.

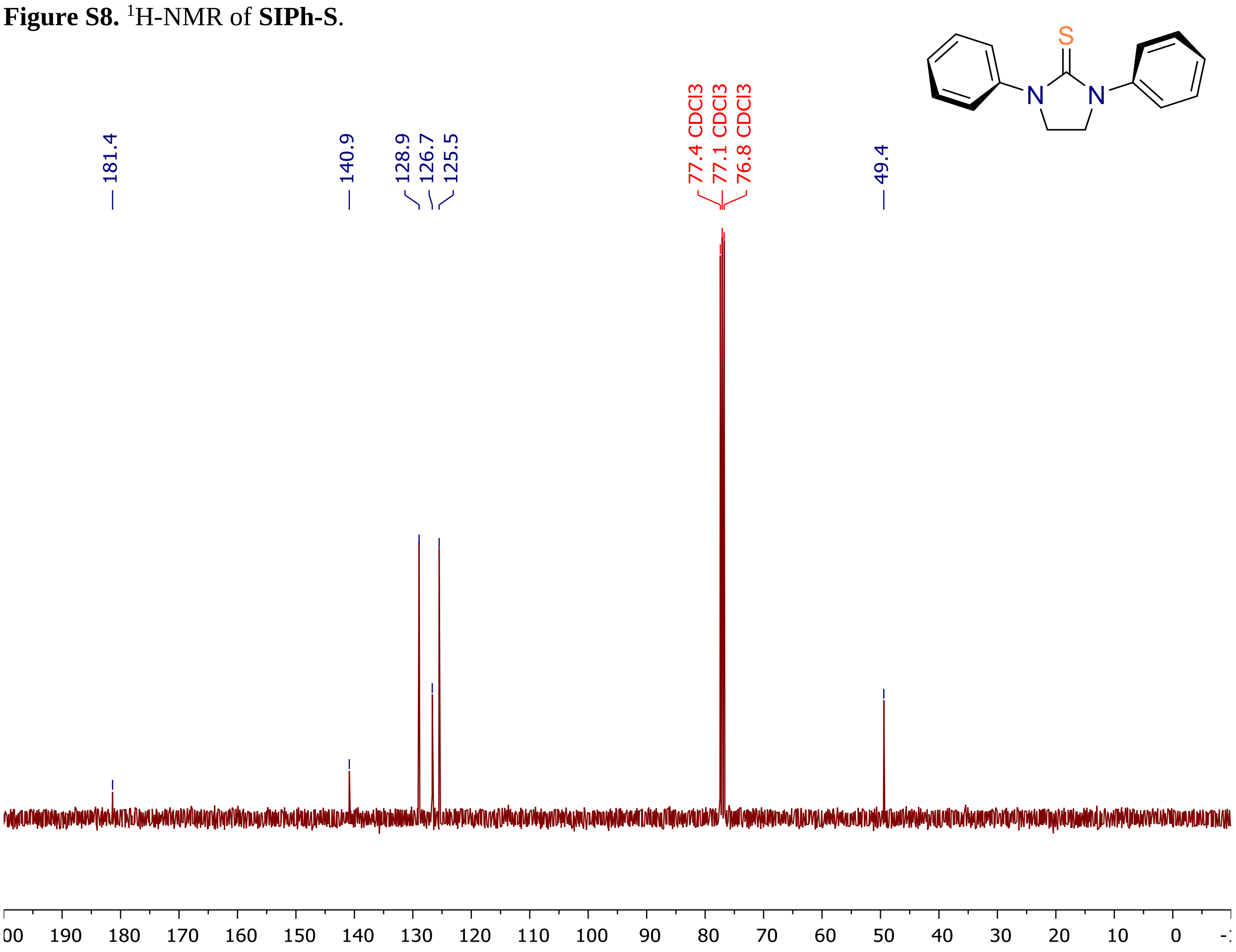

Figure S9. ^{13}C-NMR of **SIPh-S**.

Qualitative Analysis Report

**Data Filename**	Medvedko0027.D	**Sample Name**	medv0838
**Sample Type**		**Position**	3
**Instrument Name**	GCMS5977B	**User Name**	NG
**Acq Method**	S1016_11.M	**Acquired Time**	8/22/2022 12:21:22 PM (UTC+02:00)
**IRM Calibration Status**	Not Applicable	**DA Method**	TheSysNG.m
**Comment**	S1016_11.M		

**Expected Barcode**		**Sample Amount**	
**Dual Inj Vol**	1	**TuneName**	etune.u
**TunePath**	D:\MassHunter\GCMS\1\5977 \	**TuneDateStamp**	2022-04-20T09:02:24+02:00
**MSFirmwareVersion**	6.00.34	**OperatorName**	NG
**RunCompletedFlag**	True	**Acquisition Time (Local)**	8/22/2022 12:21:22 PM (UTC+02:00)
**Acquisition SW Version**	MassHunter GC/MS Acquisition 10.0.368 14-Feb-2019 Copyright © 1989-2018 Agilent Technologies, Inc	**SingleQuadrupole Driver Version**	10.0.0.0
**SingleQuadrupole Firmware Version**	6.00.34		

Chromatograms

Fragmentor Voltage **Collision Energy** 0 **Ionization Mode** EI

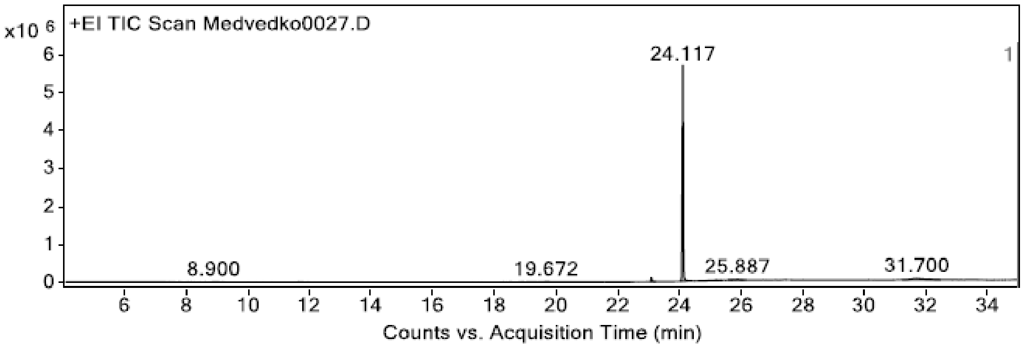

Qualitative Analysis Report

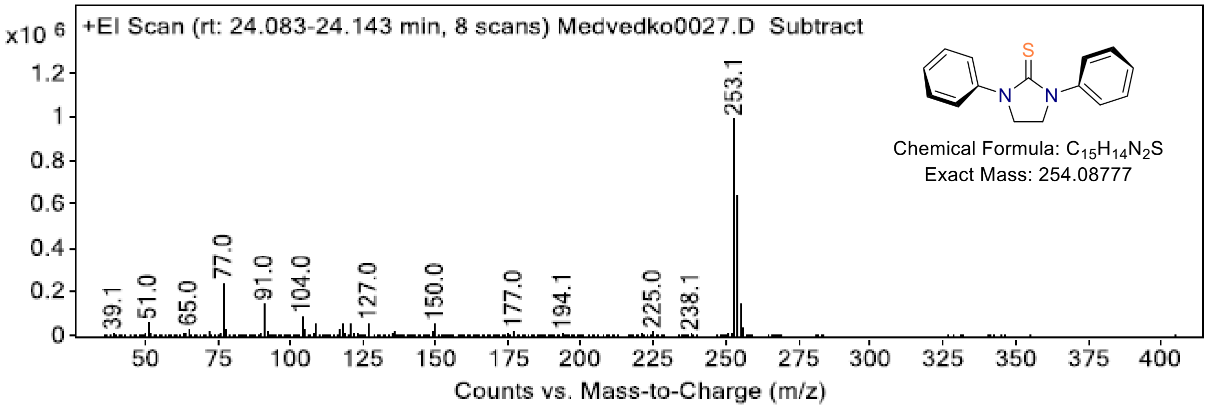

Figure S10. GC-MS of **SIPh-S**.

4. *N,N′*-Dimesitylethanediimine (B-Mes)

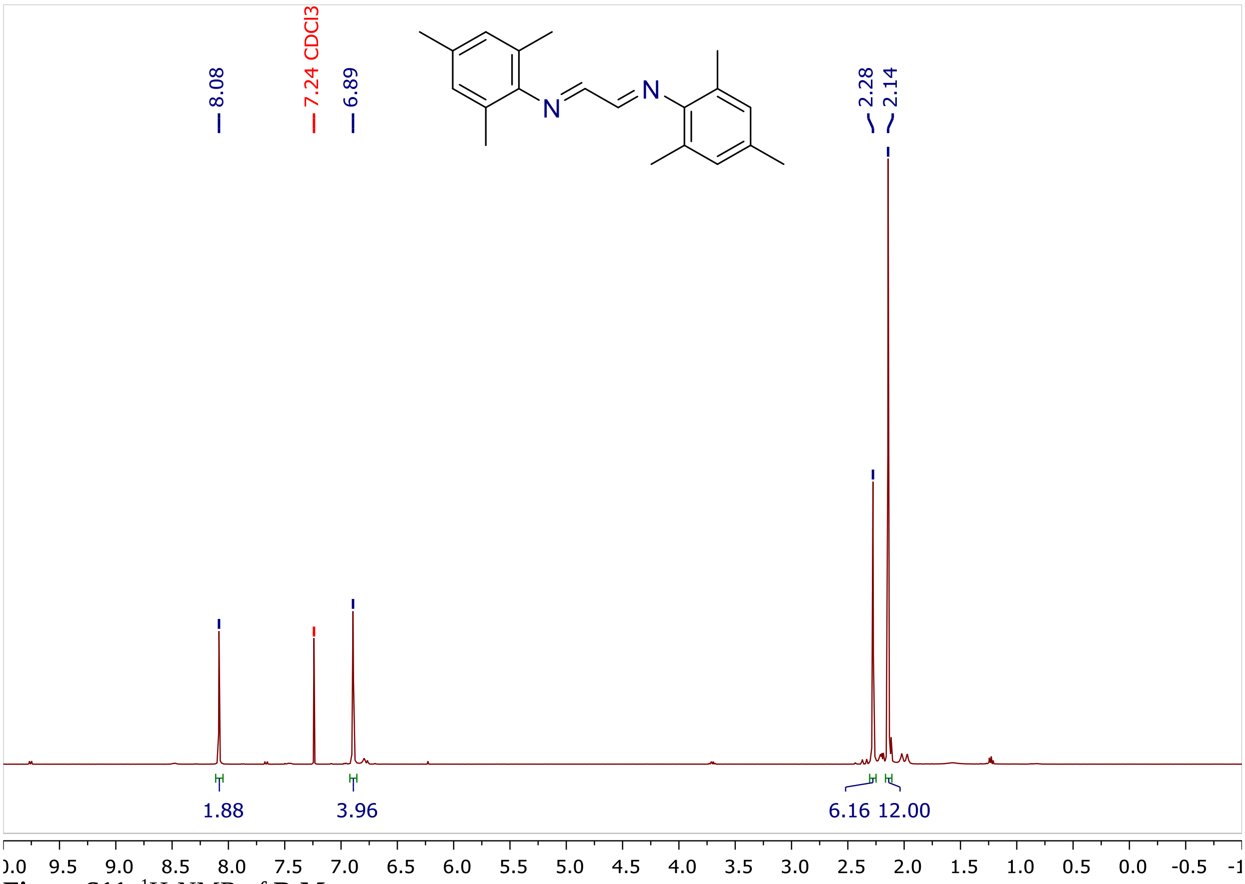

Figure S11. ^{1}H-NMR of **B-Mes**.

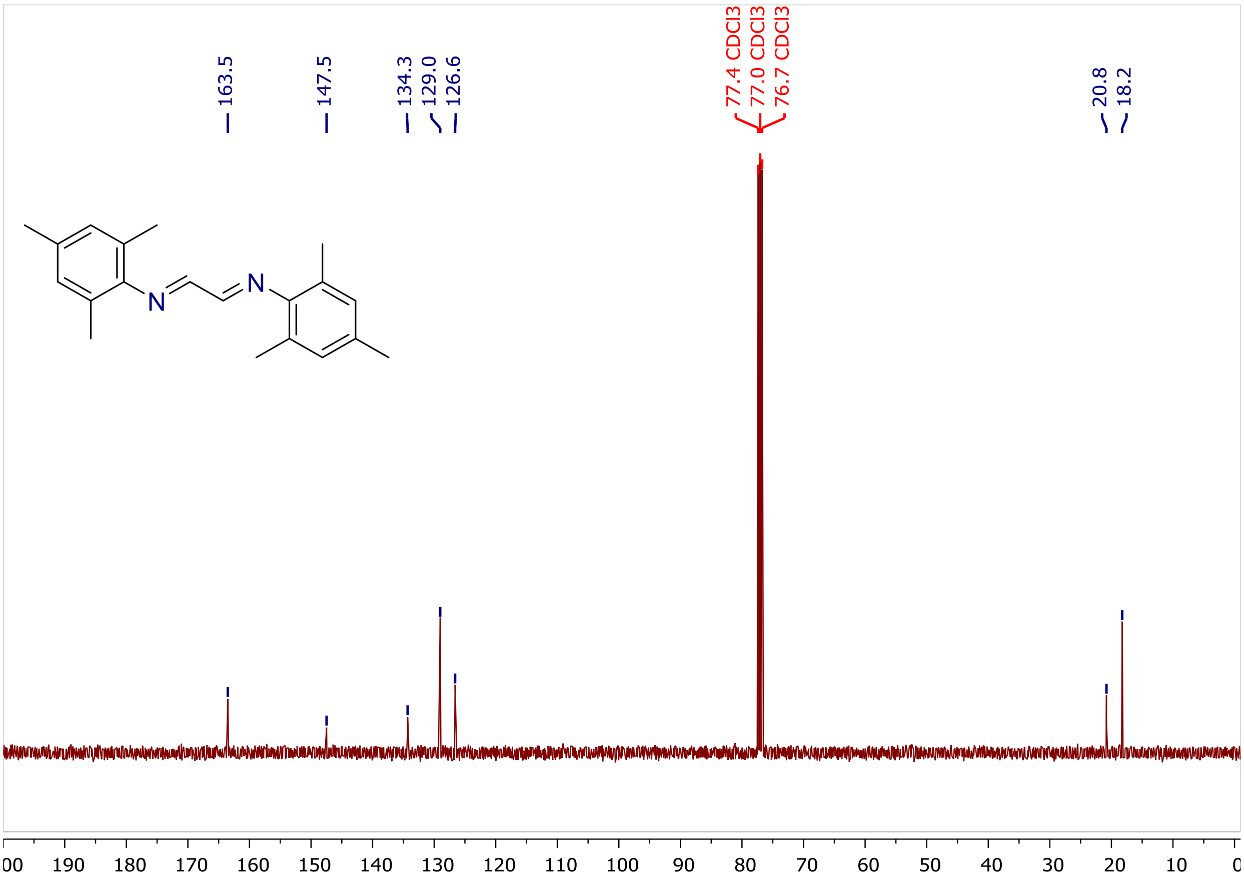

Figure S12. ^{13}C-NMR of **B-Mes**.

Chromatograms

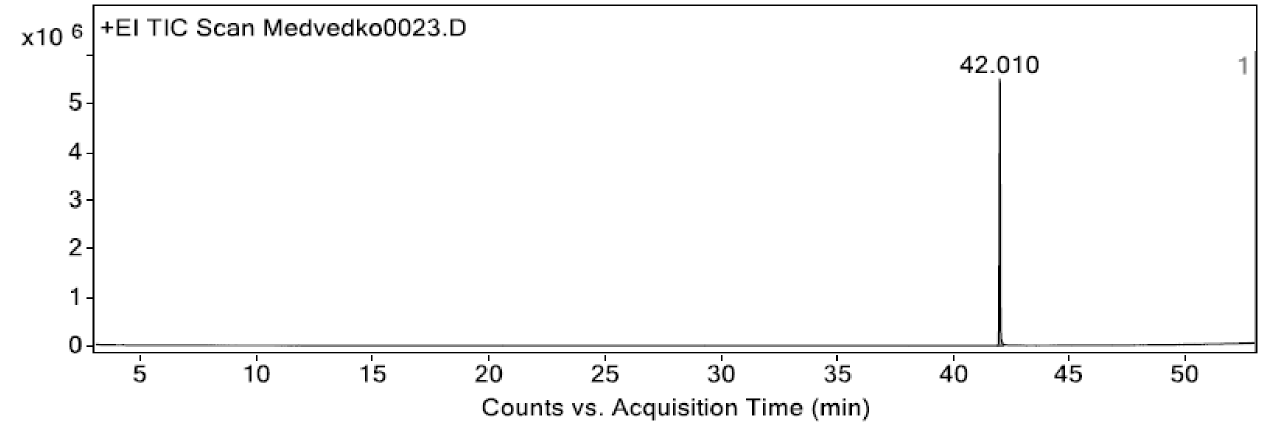

Integration Peak List

Peak	Start	RT	End	Height	Area	AreaSumPercent
1	41.924	42.01	42.172	5516331.98	16228993.72	100

Spectra

Spectrum Source	Collision Energy	Ionization Mode
Peak (1) in "+ TIC Scan"	0	EI

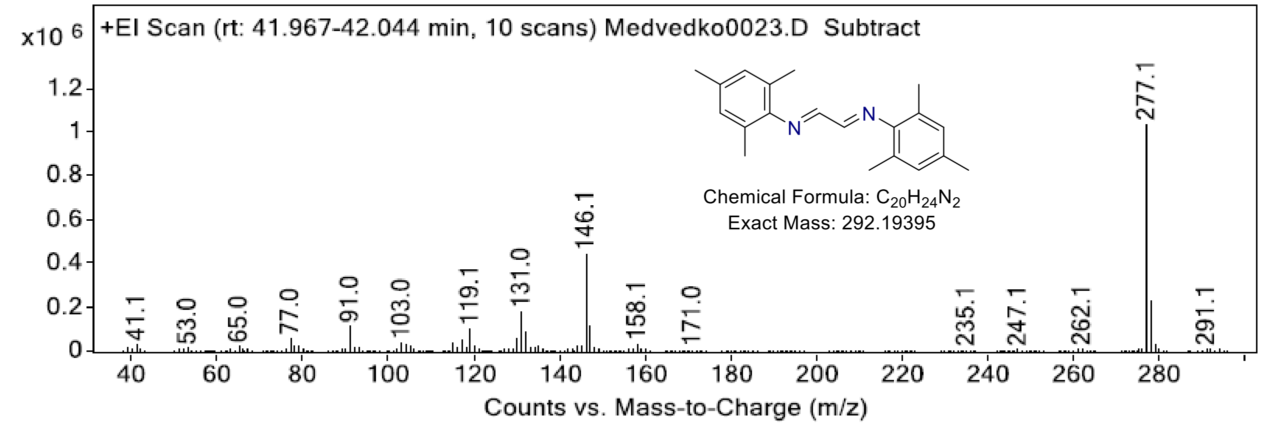

Figure S13. GC-MS of **B-Mes**.

5. *N,N′*-Dimesitylethanediamine (C-Mes)

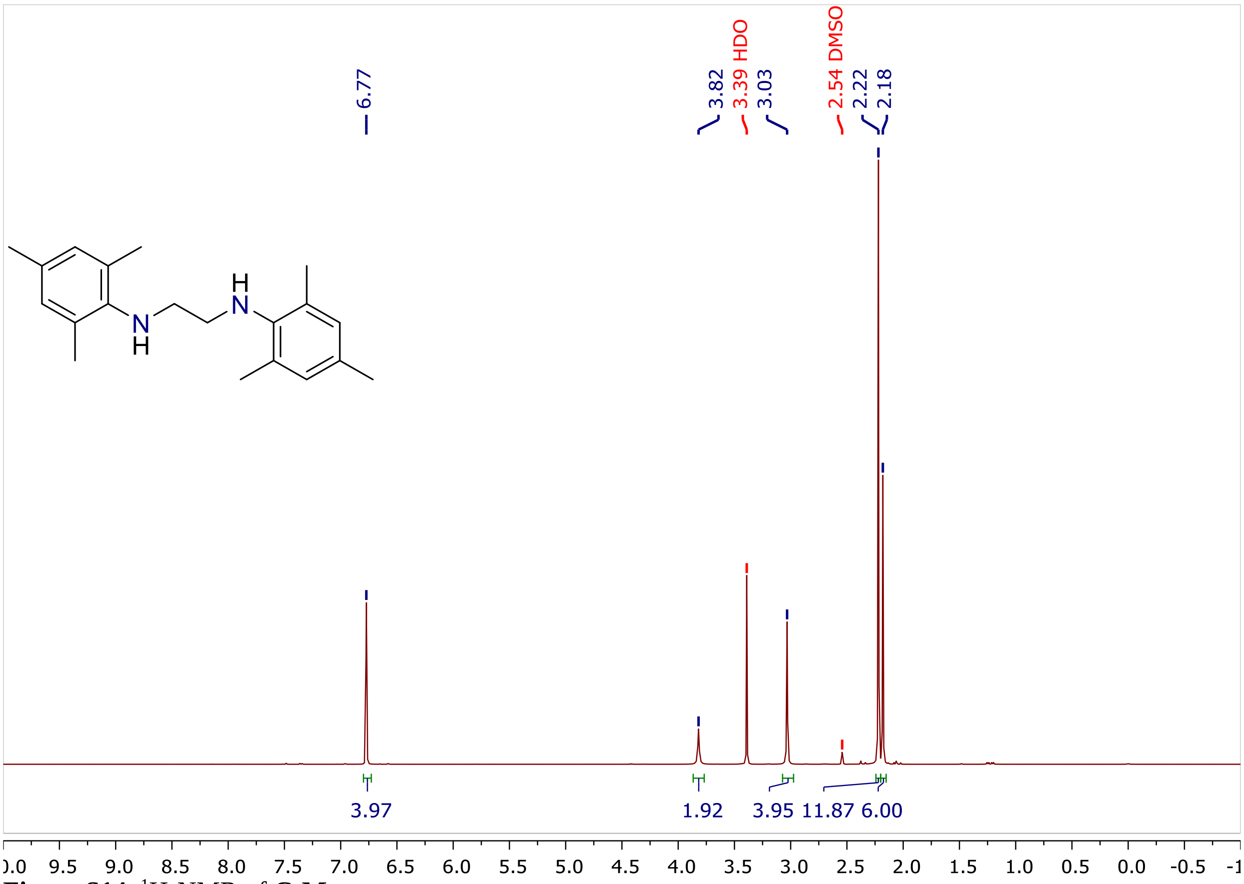

Figure S14. ^{1}H-NMR of **C-Mes**.

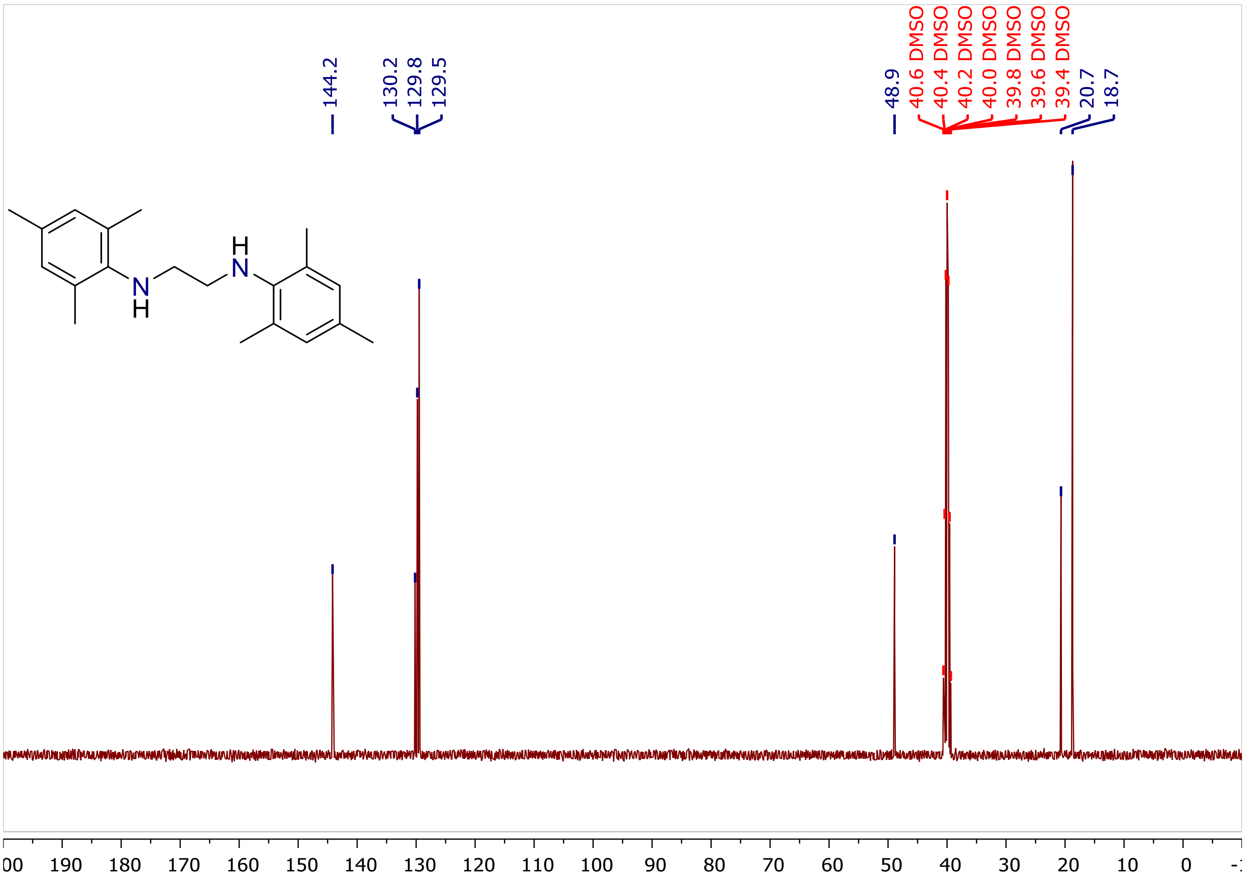

Figure S15. ^{13}C-NMR of **C-Mes**.

Chromatograms

Fragmentor Voltage **Collision Energy** 0 **Ionization Mode** EI

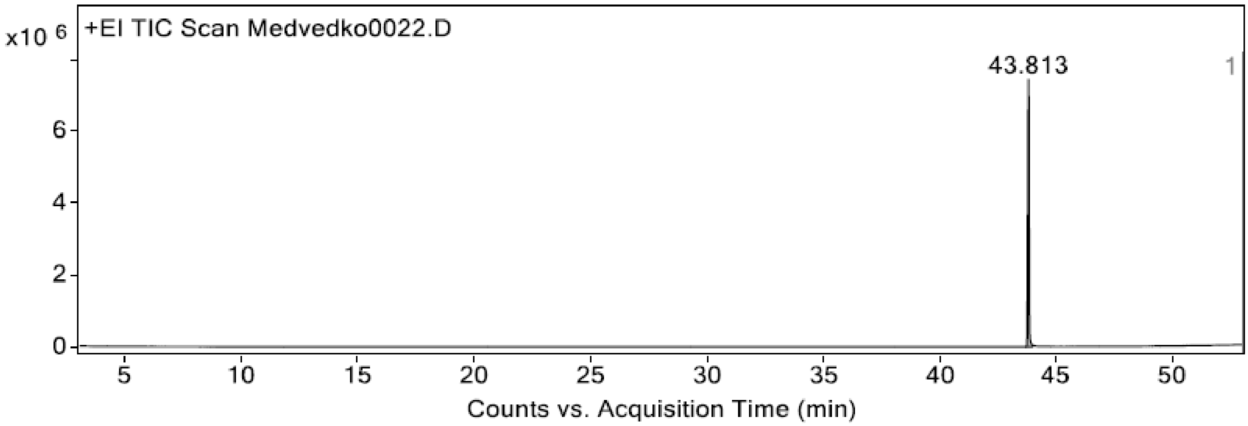

Integration Peak List

Peak	Start	RT	End	Height	Area	AreaSumPercent
1	43.711	43.813	43.976	7452828.97	23988551.31	100

Spectra

**Spectrum Source**	**Collision Energy**	**Ionization Mode**
Peak (1) in "+ TIC Scan"	0	EI

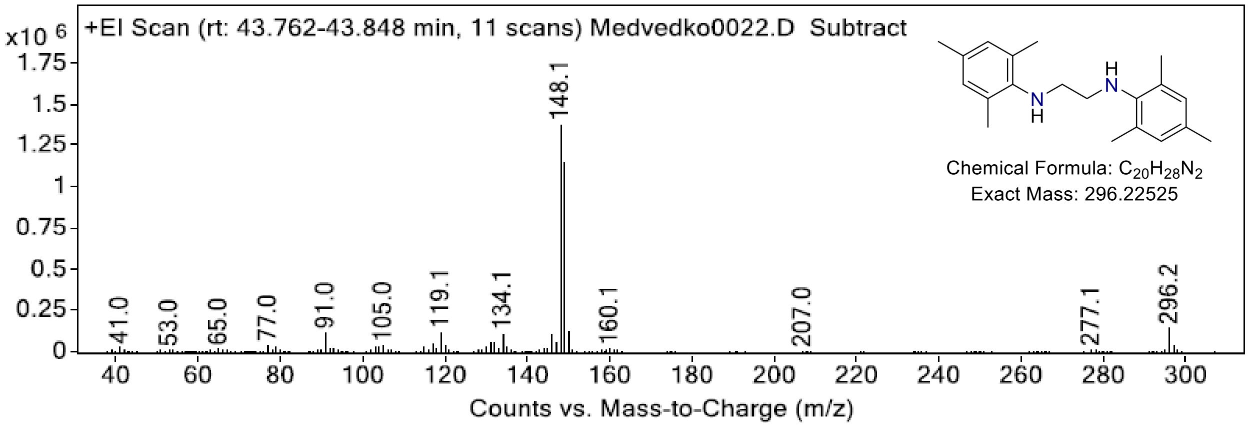

Figure S16. GC-MS of **C-Mes**.

6. 1,3-Dimesitylimidazolidine-2-thione (SIMes-S)

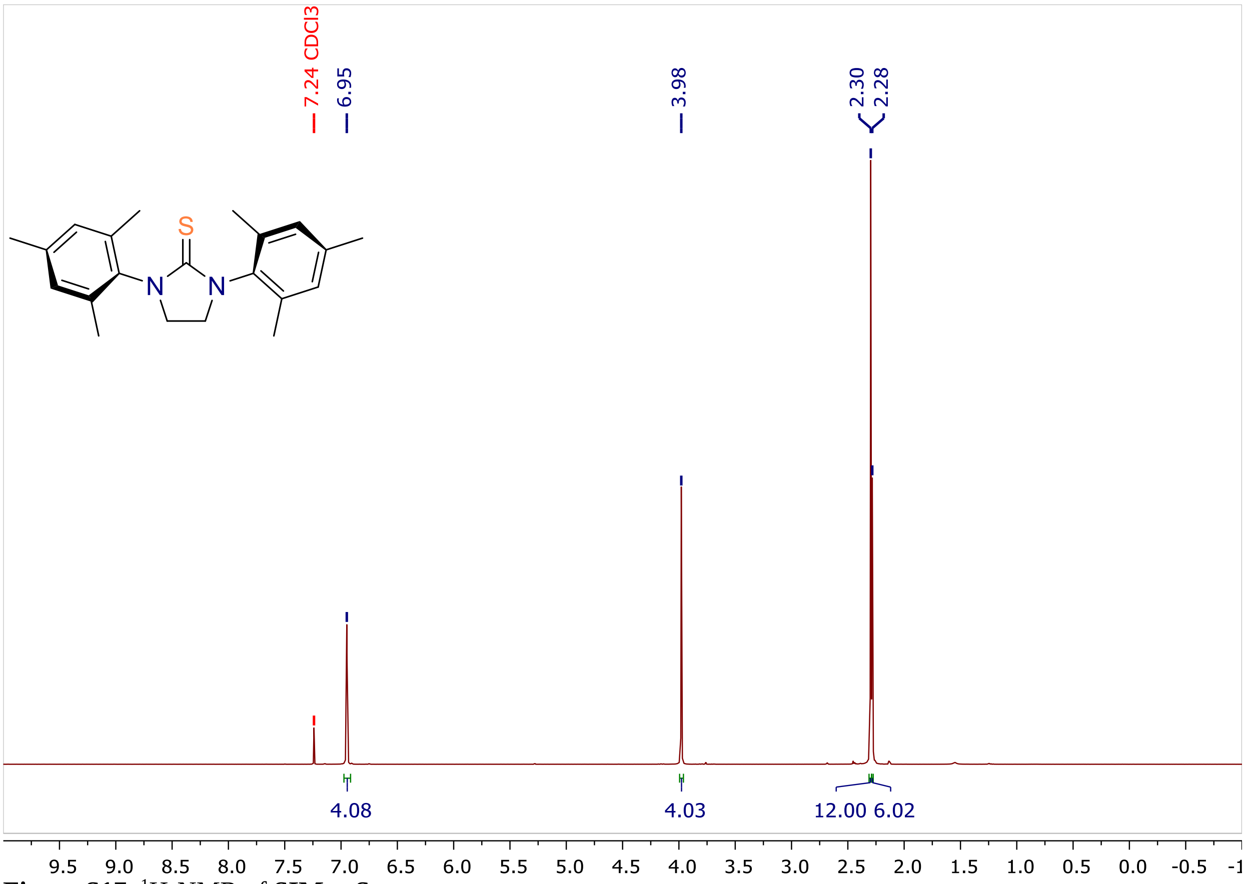

Figure S17. ^{1}H-NMR of **SIMes-S**.

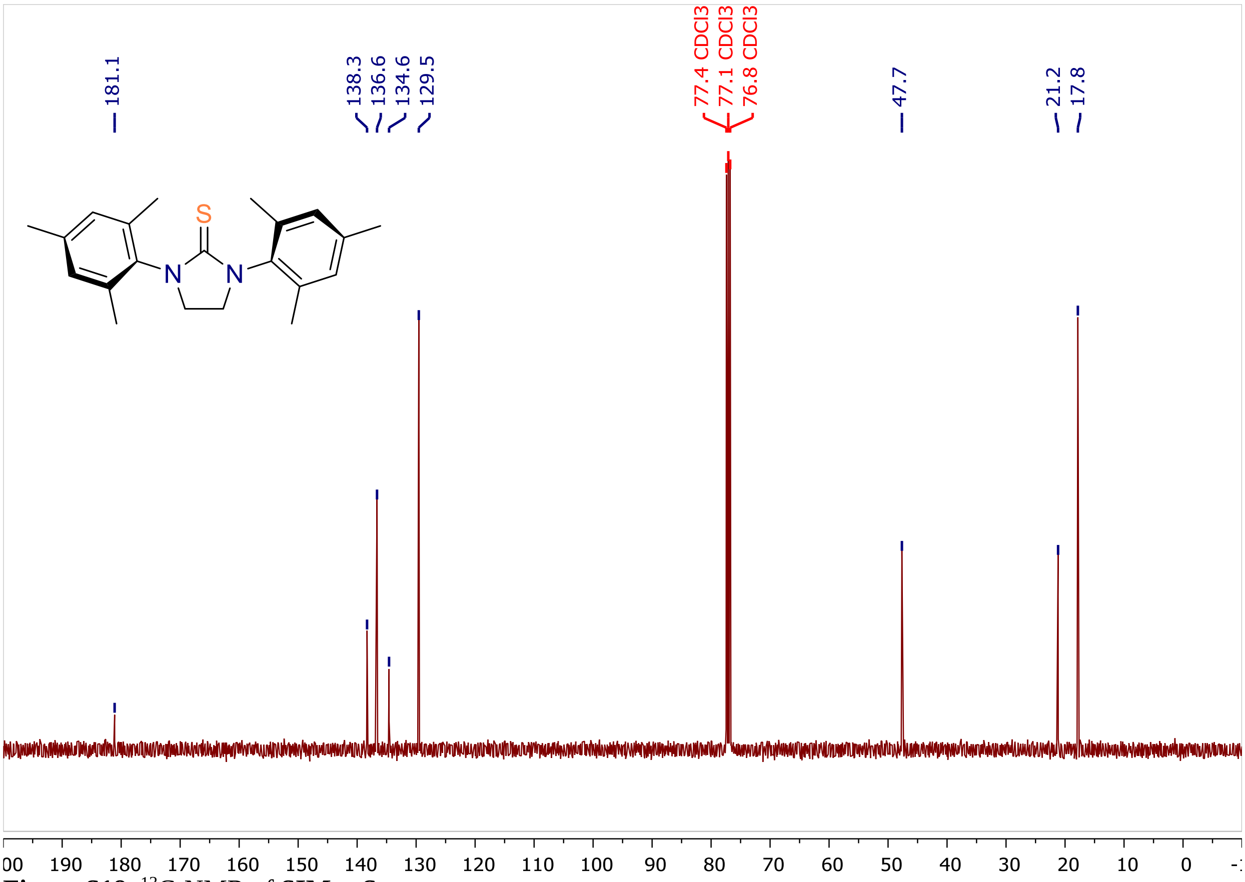

Figure S18. ^{13}C-NMR of **SIMes-S**.

Chromatograms

Fragmentor Voltage **Collision Energy** 0 **Ionization Mode** EI

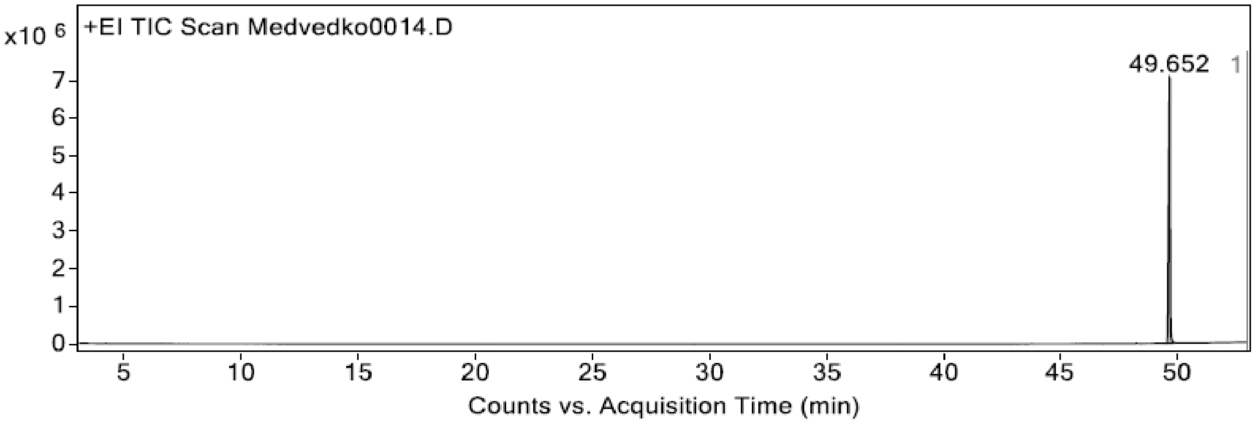

Integration Peak List

Peak	Start	RT	End	Height	Area	AreaSumPercent
1	49.524	49.652	49.815	7056660.81	30331110.31	100

Spectra

**Spectrum Source**	**Collision Energy**	**Ionization Mode**
Peak (1) in "+ TIC Scan"	0	EI

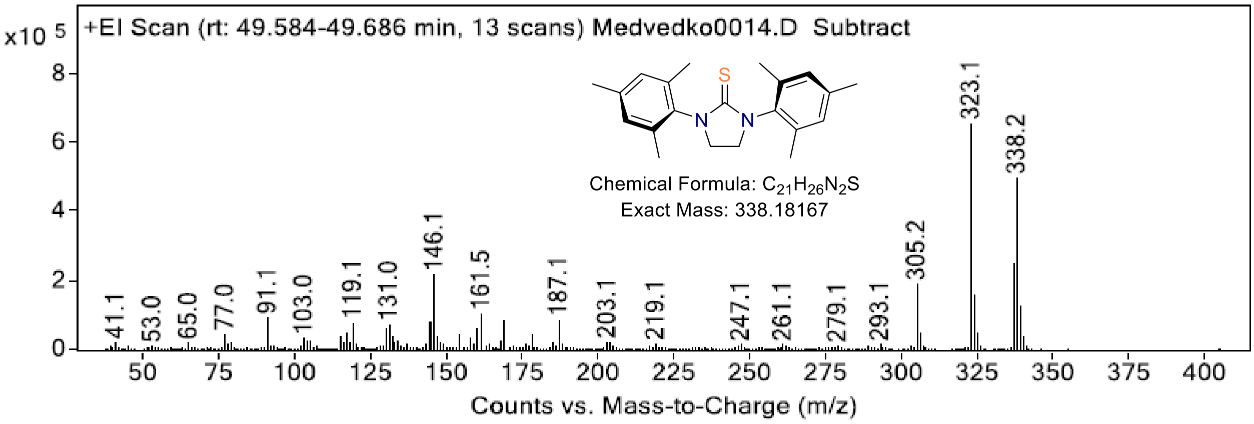

Figure S19. GC-MS of **SIMes-S**.

7. *N,N′*-Bis(2,6-diisopropylphenyl)ethanediimine (B-Dipp)

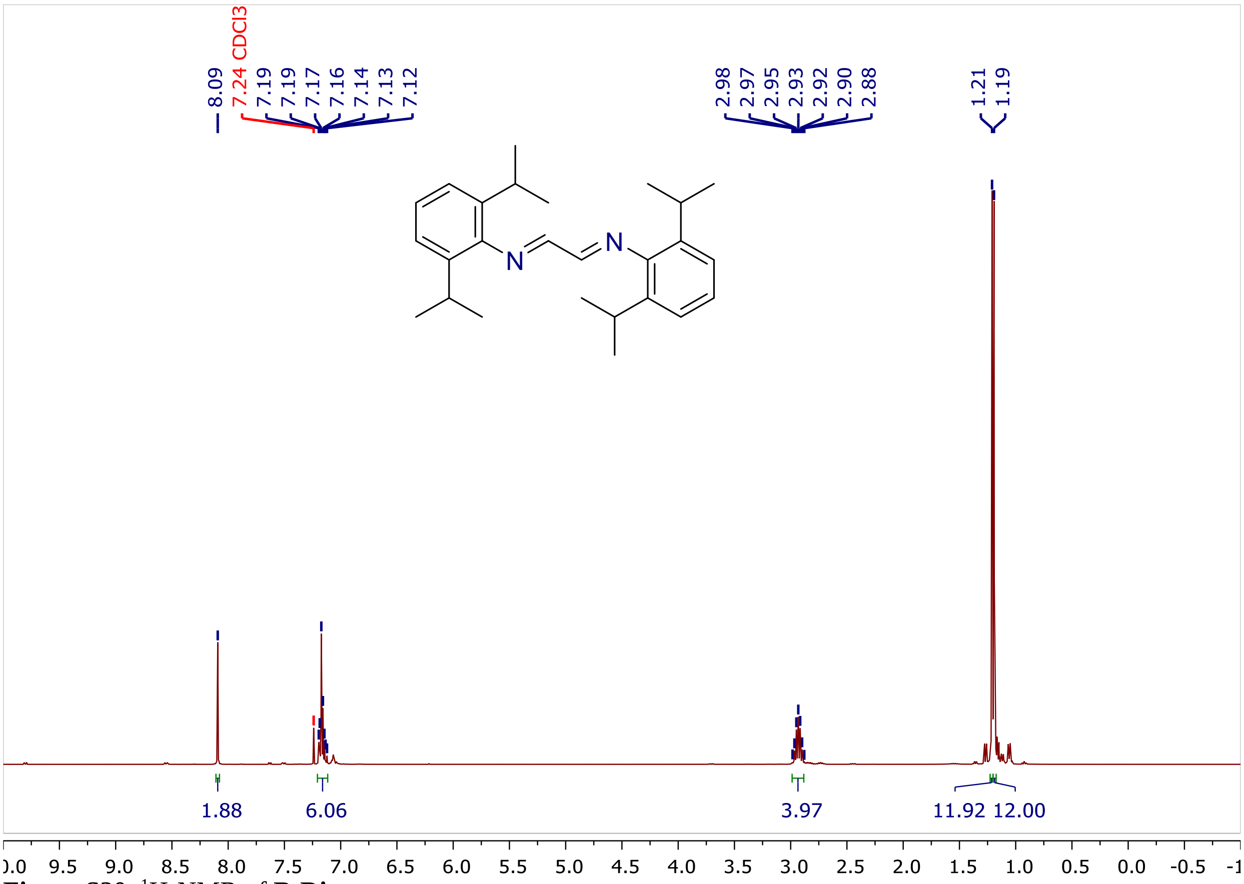

Figure S20. ^{1}H-NMR of **B-Dipp**.

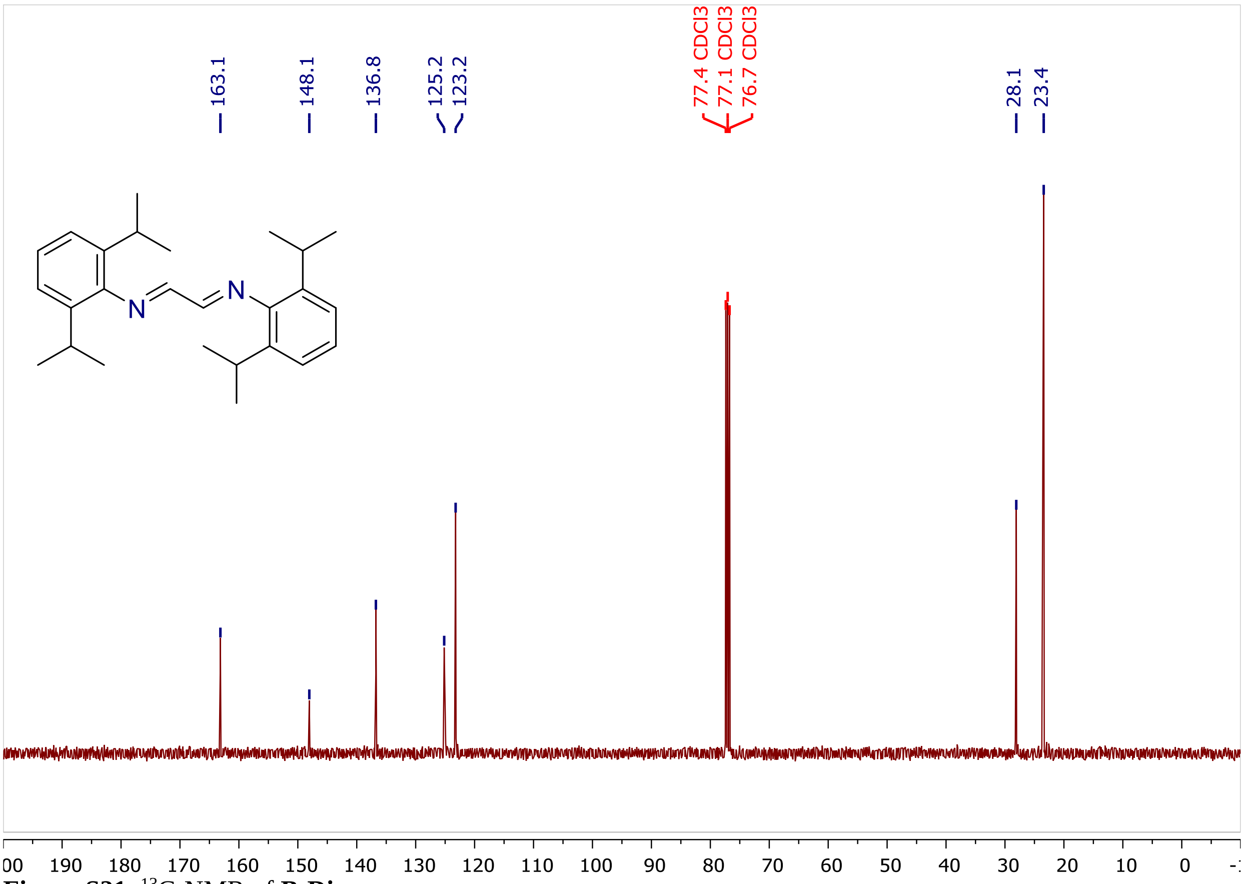

Figure S21. ^{13}C-NMR of **B-Dipp**.

Chromatograms

Fragmentor Voltage **Collision Energy** 0 **Ionization Mode** EI

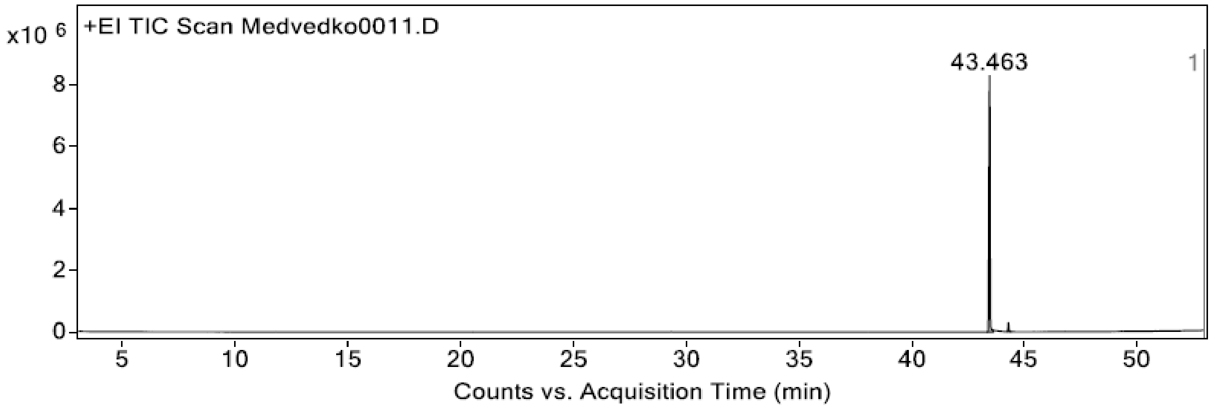

Integration Peak List

Peak	Start	RT	End	Height	Area	AreaSumPercent
1	43.36	43.463	43.625	8319732.83	26956205.09	97.17
2	44.237	44.284	44.425	275987.62	785259.22	2.83

Spectra

Spectrum Source	Collision Energy	Ionization Mode
Peak (1) in "+ TIC Scan"	0	EI

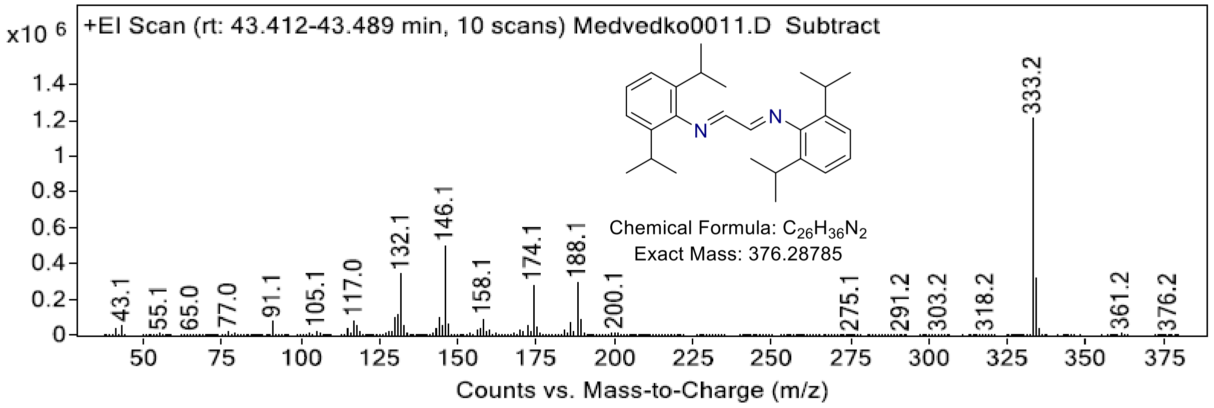

Figure S22. GC-MS of **B-Dipp**.

8. *N,N′*-Bis(2,6-diisopropylphenyl)ethanediamine (C-Dipp)

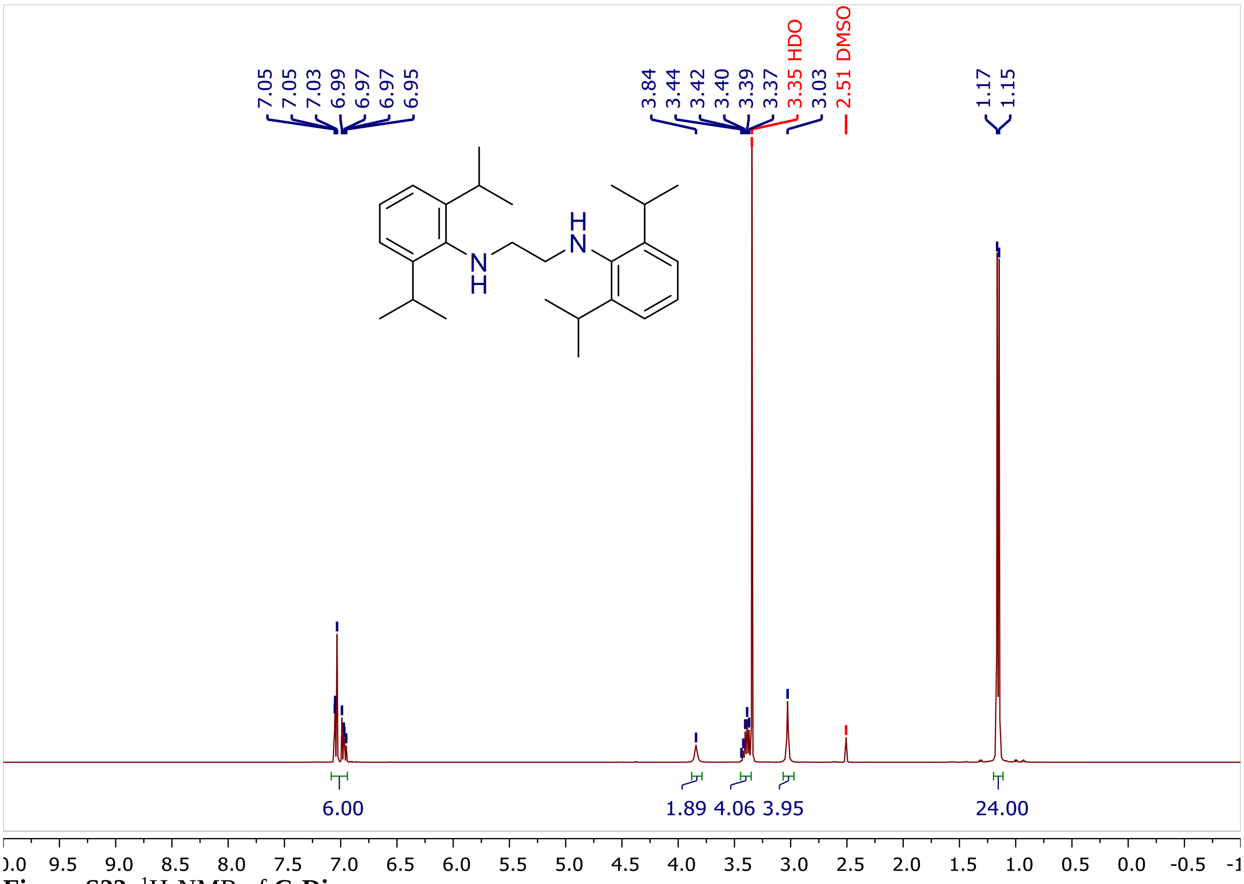

Figure S23. ^{1}H-NMR of **C-Dipp**.

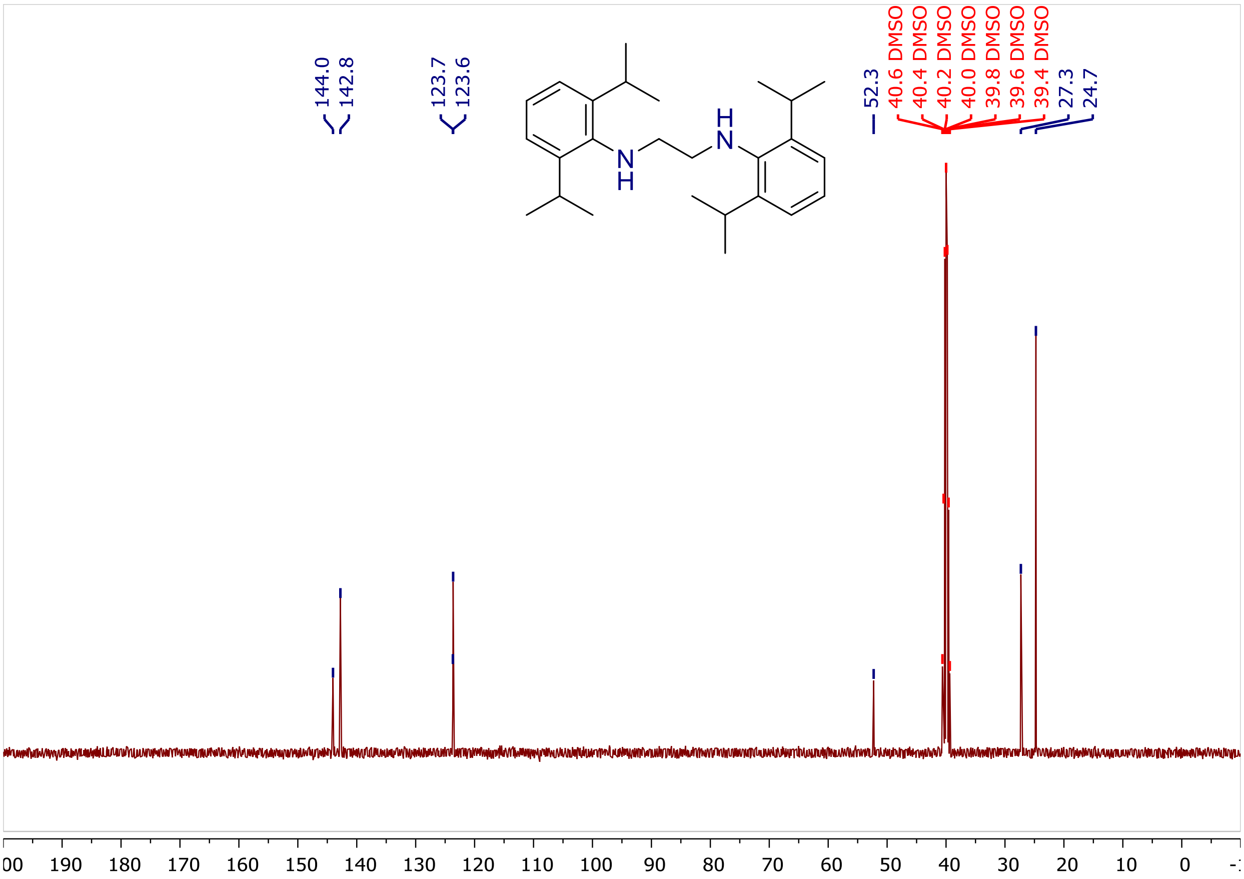

Figure S24. ^{13}C-NMR of **C-Dipp**.

Chromatograms

Fragmentor Voltage **Collision Energy** 0 **Ionization Mode** EI

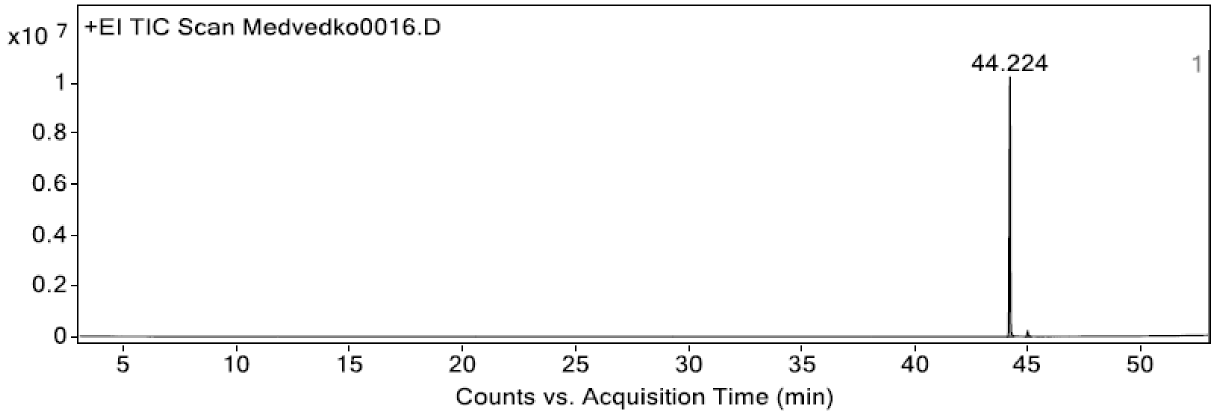

Integration Peak List

Peak	Start	RT	End	Height	Area	AreaSumPercent
1	44.121	44.224	44.386	10229838.34	34245147.79	98.05
2	44.925	44.993	45.138	204212.62	680671.69	1.95

Spectra

**Spectrum Source**	**Collision Energy**	**Ionization Mode**
Peak (1) in "+ TIC Scan"	0	EI

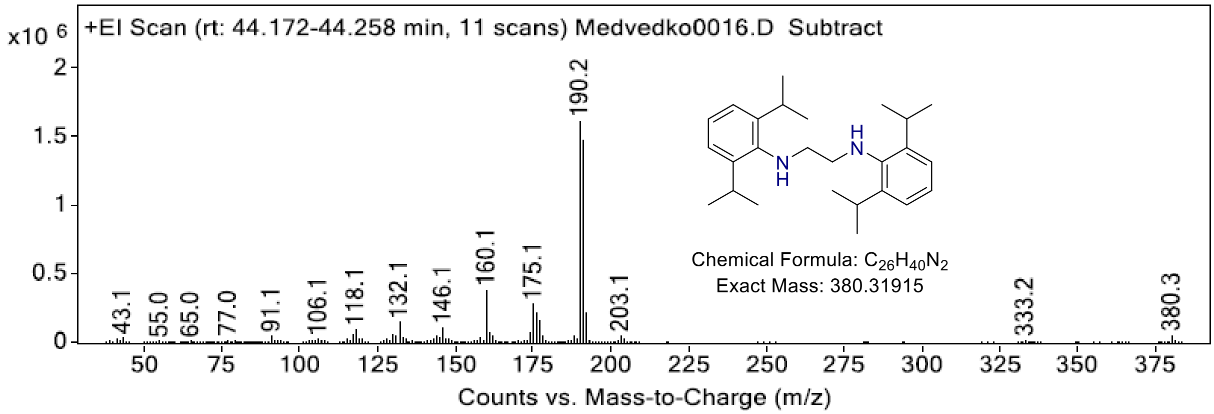

Figure S25. GC-MS of **C-Dipp**.

9. 1,3-Bis(2,6-diisopropylphenyl)imidazolidine-2-thione (SIDipp-S)

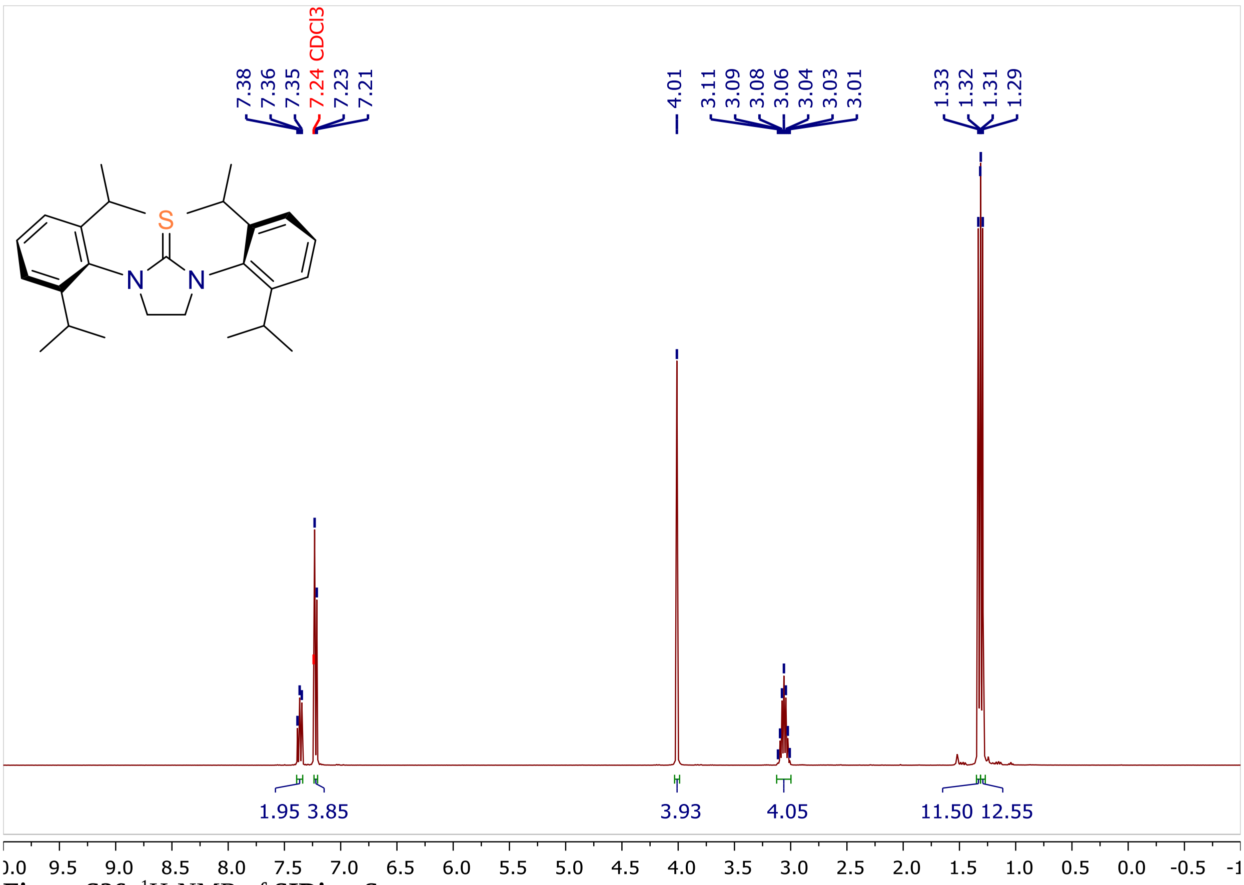

Figure S26. ^{1}H-NMR of **SIDipp-S**.

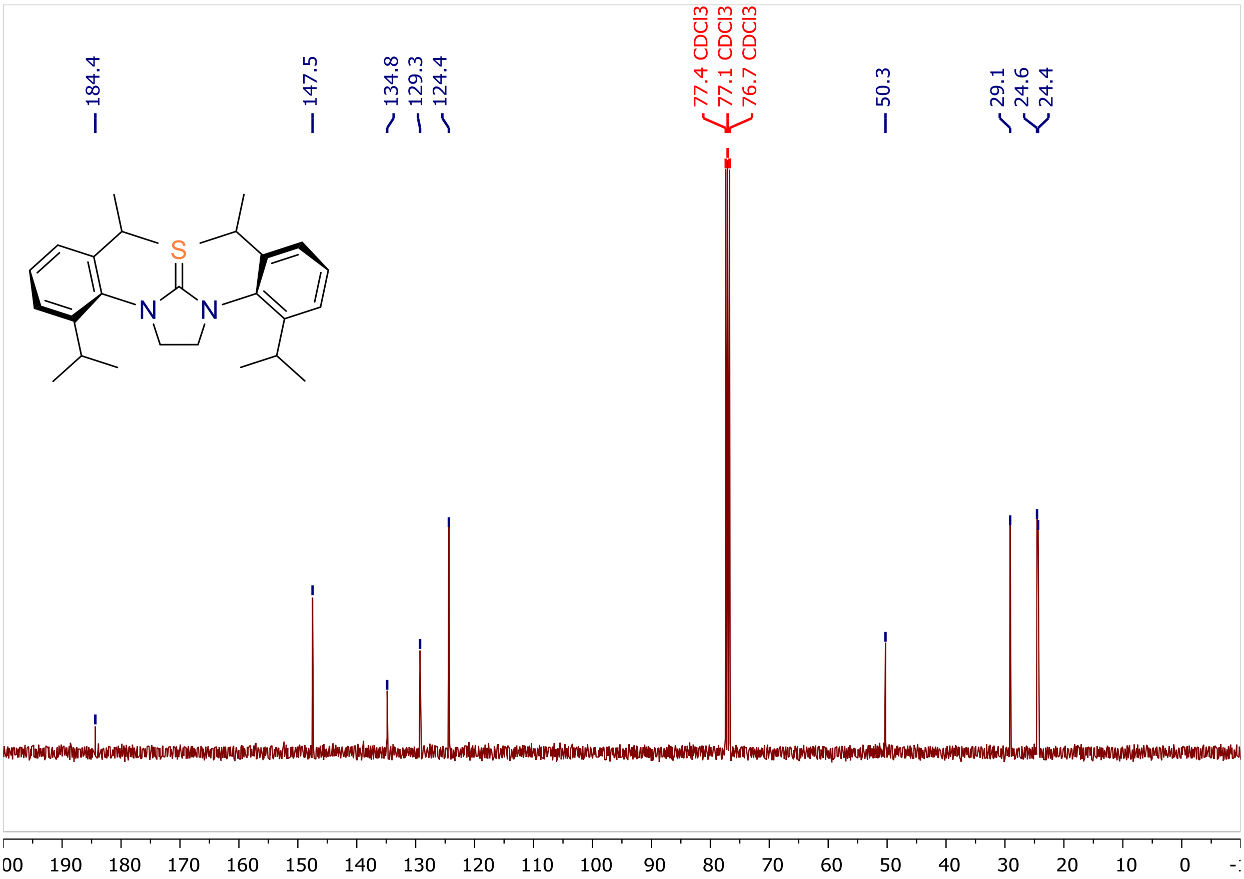

Figure S27. ^{13}C-NMR of **SIDipp-S**.

Acquisition Parameter

Ion Source Type	ESI	Ion Polarity	Positive	Alternating Ion Polarity	off
Mass Range Mode	UltraScan	Scan Begin	50 m/z	Scan End	800 m/z
Accumulation Time	1034 µs	RF Level	63 %	Trap Drive	49.7
SPS Target Mass	400 m/z	Averages	10 Spectra	n/a	n/a

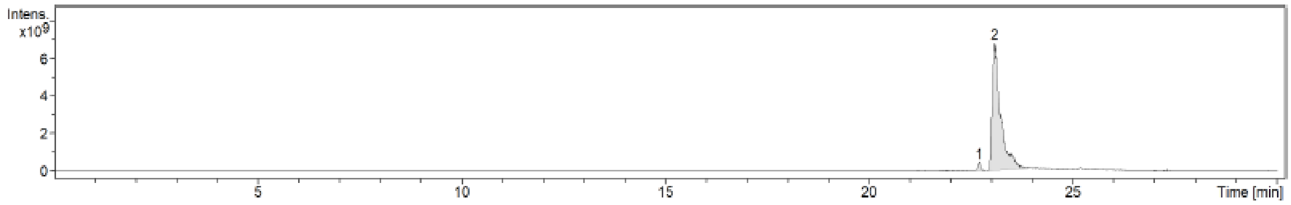

RT [min]	Area
22.70	2438710784
23.07	97544200192

Cmpd 1, 22.70 min

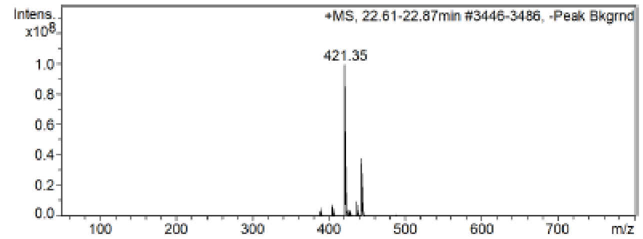

Cmpd 2, 23.07 min

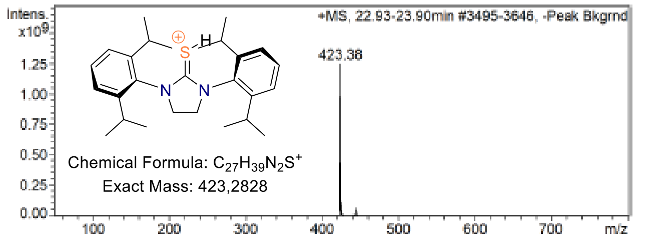

Figure S28. HPLC-ESI-MS of **SIDipp-S**.

10. 1,3-Dimethylimidazole-2-thione (IMe-S)

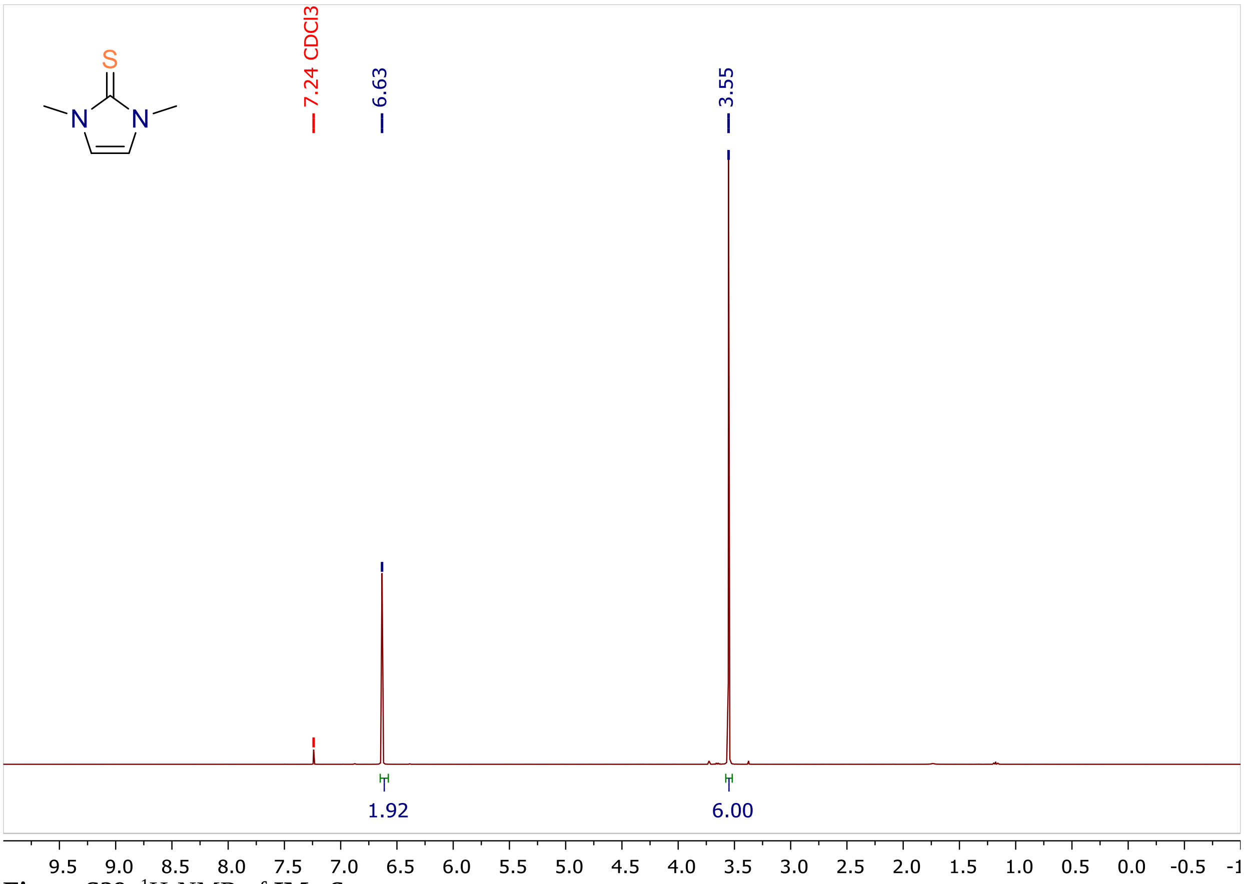

Figure S29. ^{1}H-NMR of **IMe-S**.

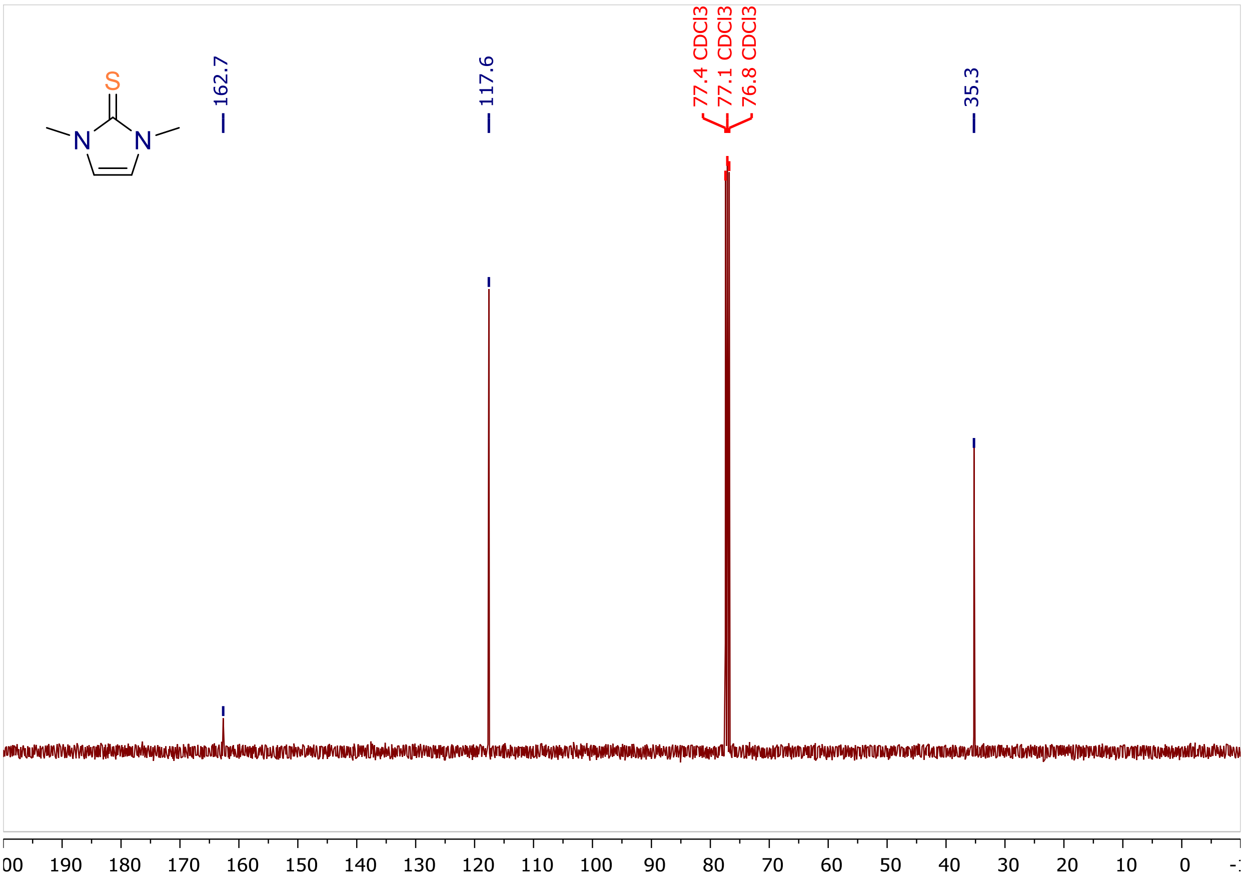

Figure S30. ^{13}C-NMR of **IMe-S**.

Chromatograms

Fragmentor Voltage **Collision Energy** 0 **Ionization Mode** EI

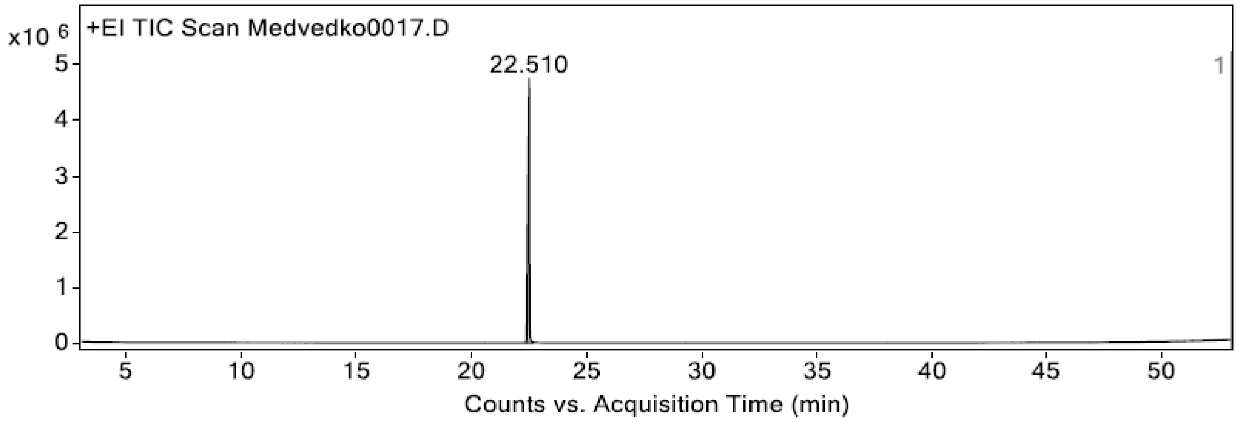

Integration Peak List

Peak	Start	RT	End	Height	Area	AreaSumPercent
1	22.374	22.51	22.672	4757835.31	21383049.6	100

Spectra

Spectrum Source Peak (1) in "+ TIC Scan"
Collision Energy 0
Ionization Mode EI

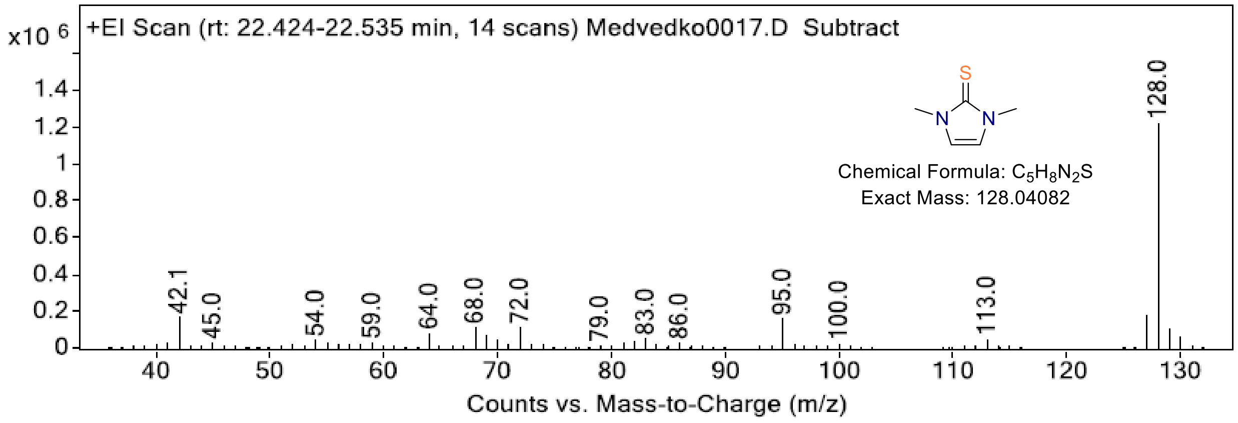

Figure S31. GC-MS of **IMe-S**.

11. *N,N′*-Di-*tert*-butylethanediimine (B-*ᵗ*Bu)

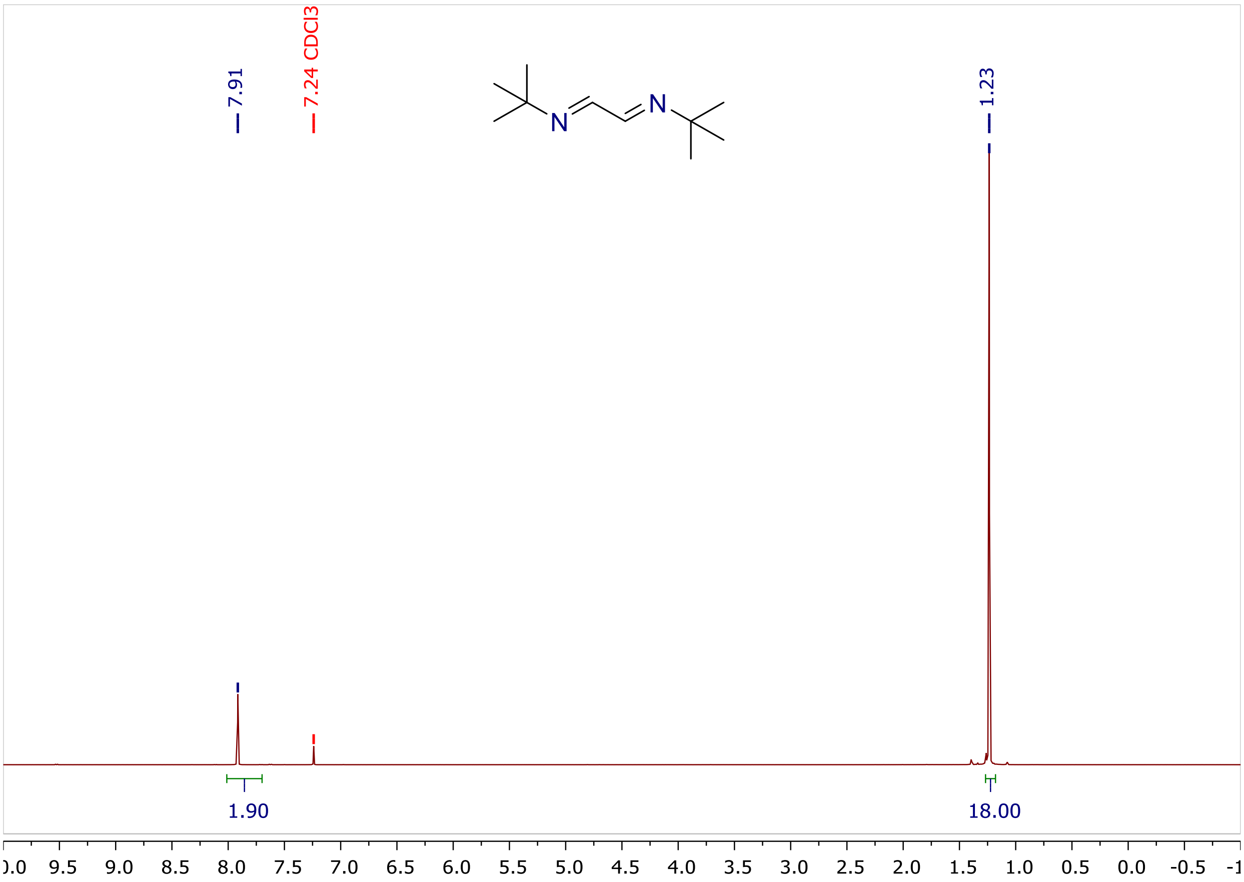

Figure S32. ^{1}H-NMR of **B-^{t}Bu**.

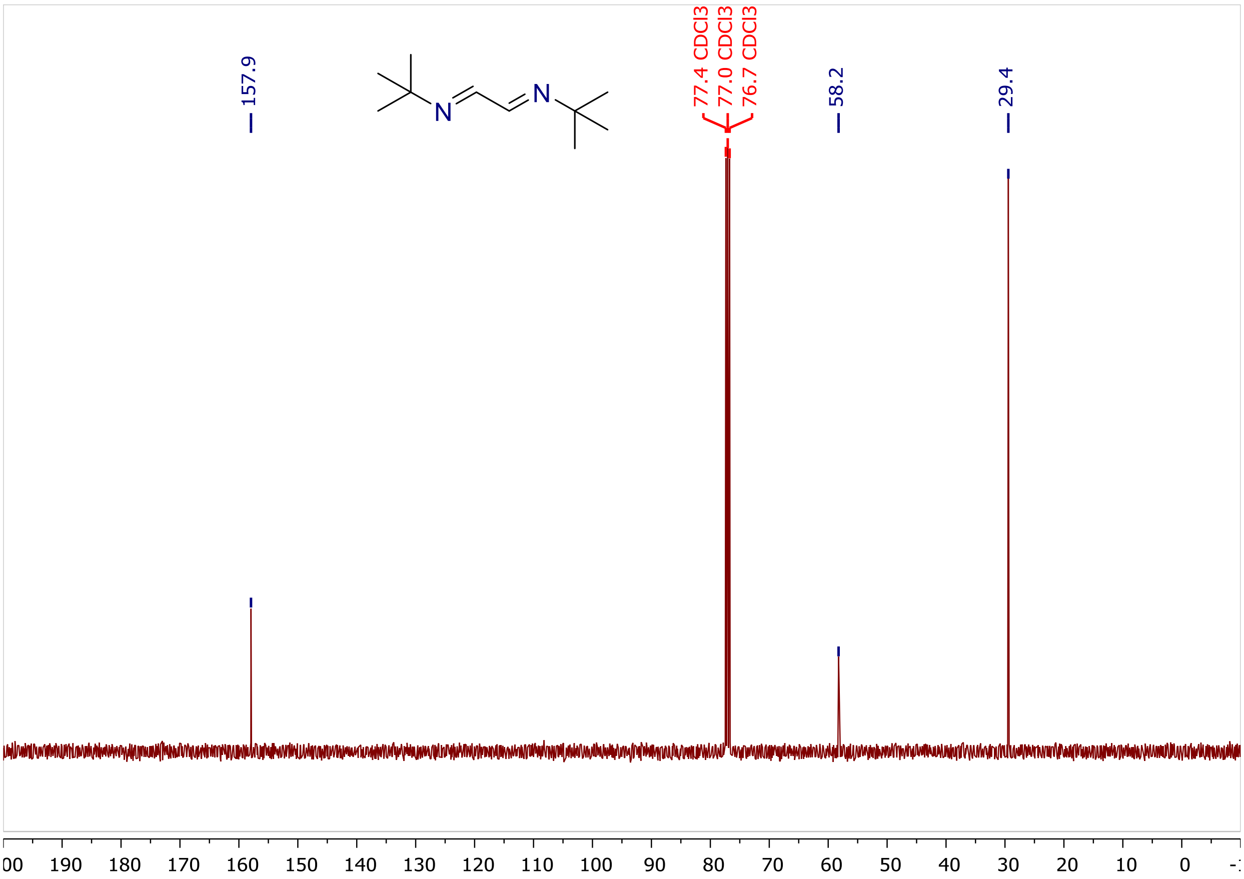

Figure S33. ^{13}C-NMR of **B-^{t}Bu**.

Chromatograms

Fragmentor Voltage **Collision Energy** 0 **Ionization Mode** EI

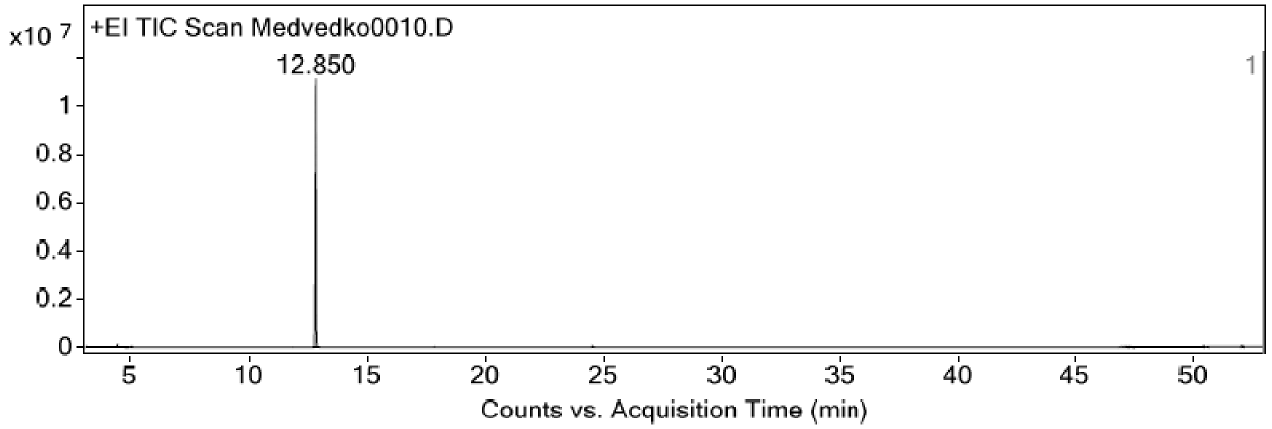

Integration Peak List

Peak	Start	RT	End	Height	Area	AreaSumPercent
1	12.764	12.85	12.961	11146148.11	30368519.87	100

Spectra

Spectrum Source Peak (1) in "+ TIC Scan"

Collision Energy 0

Ionization Mode EI

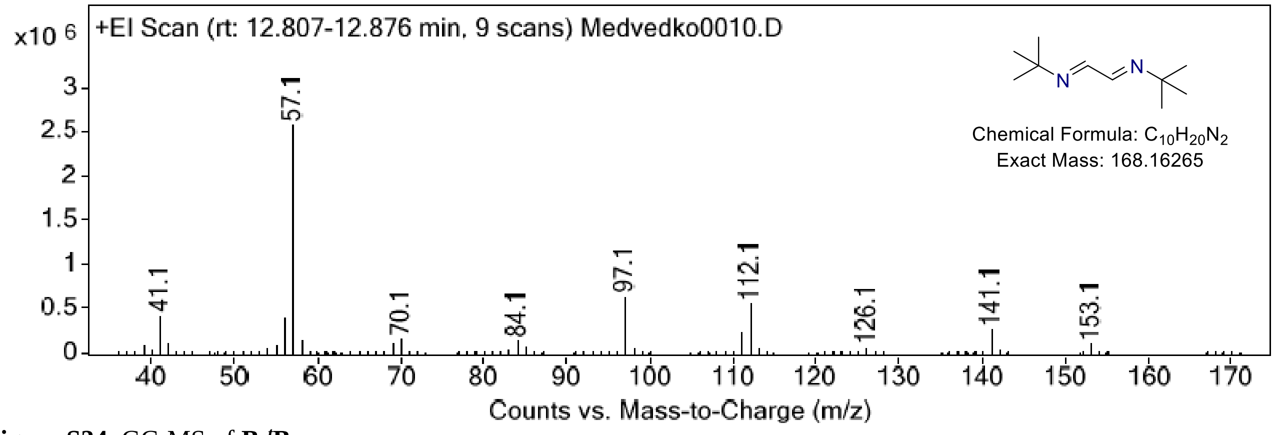

Figure S34. GC-MS of **B-^{t}Bu**.

12. 1,3-Di-*tert*-butylimidazolium chloride (I^{t}Bu-H$^+$)

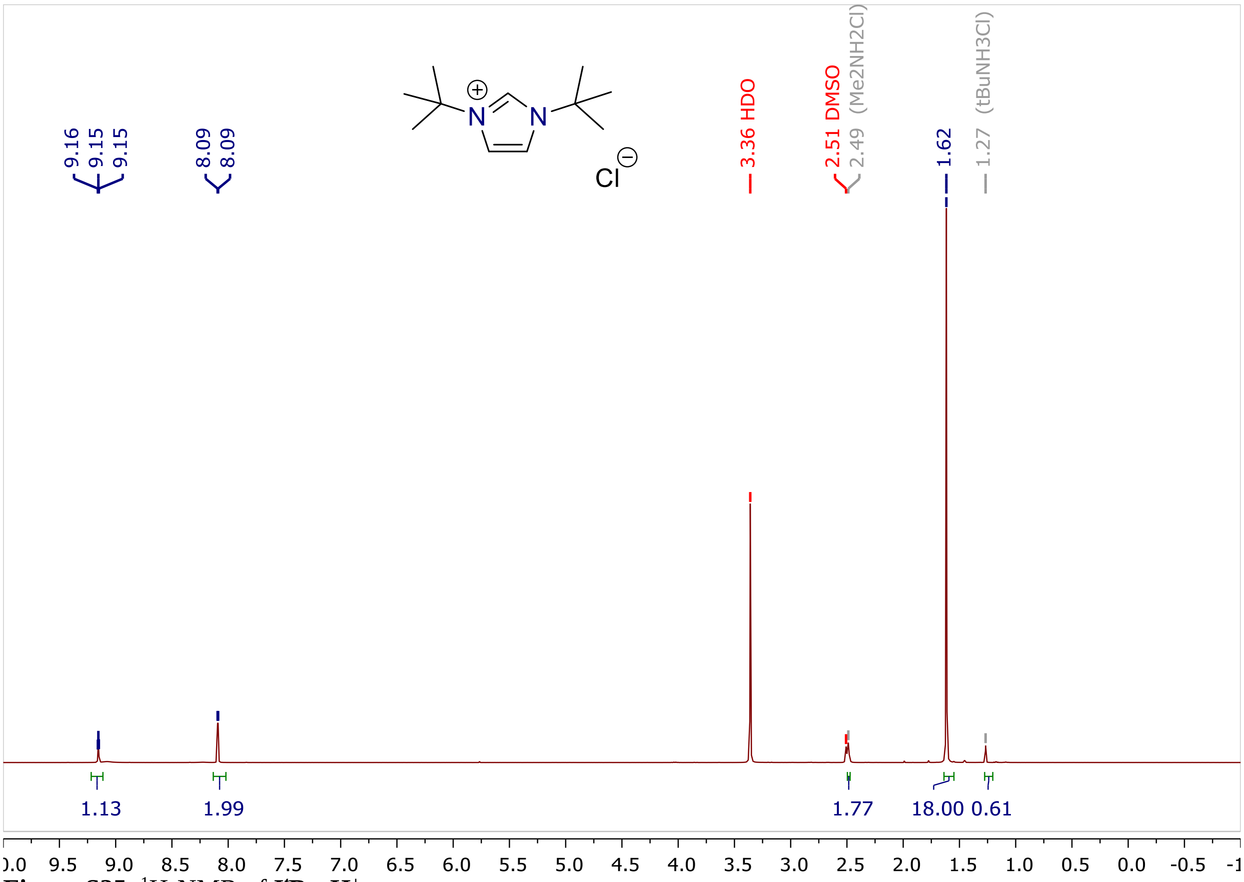

Figure S35. ^{1}H-NMR of **I^{t}Bu-H$^+$**.

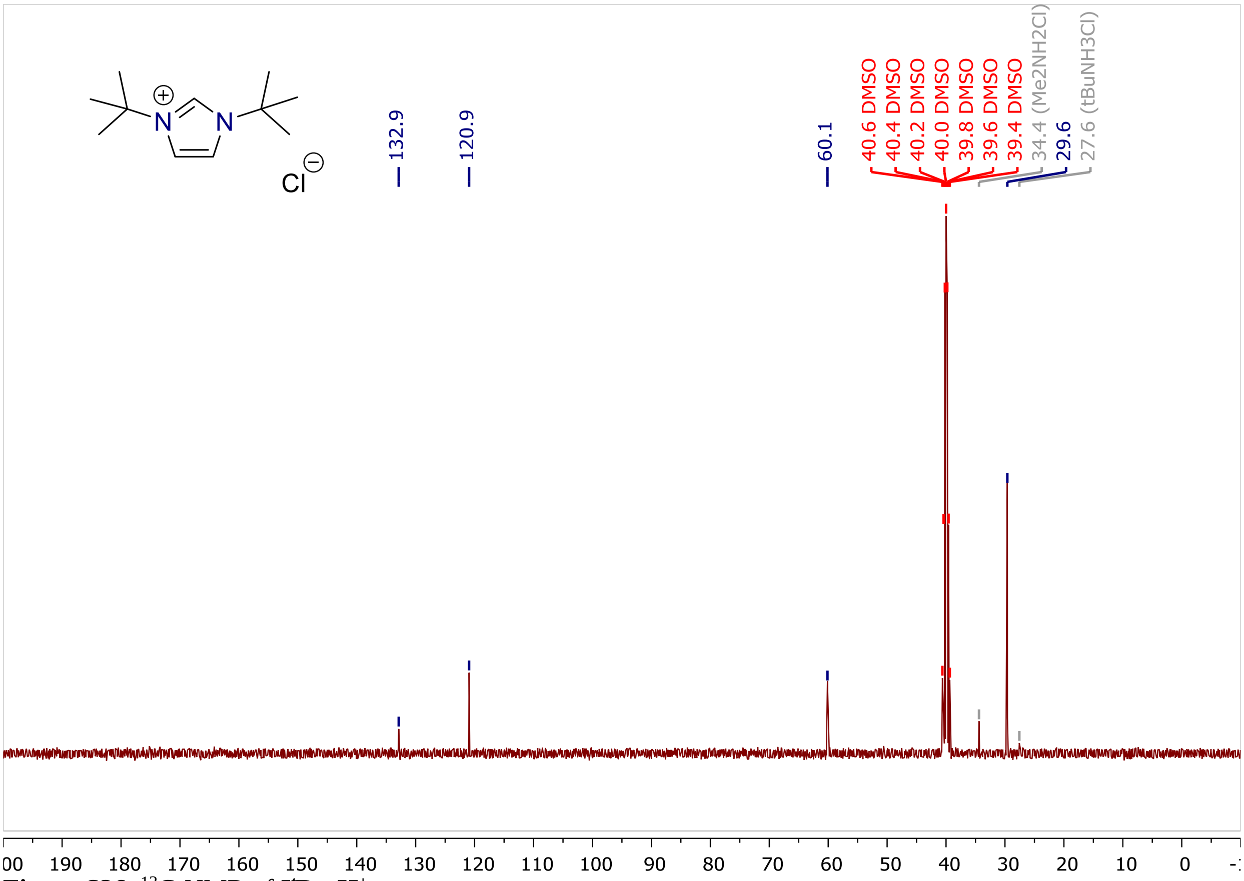

Figure S36. ^{13}C-NMR of **I^{t}Bu-H$^+$**.

Acquisition Parameter

Ion Source Type	ESI	Ion Polarity	Positive	Alternating Ion Polarity	off
Mass Range Mode	UltraScan	Scan Begin	50 m/z	Scan End	800 m/z
Accumulation Time	1162 µs	RF Level	39 %	Trap Drive	40.5
SPS Target Mass	150 m/z	Averages	10 Spectra	n/a	n/a

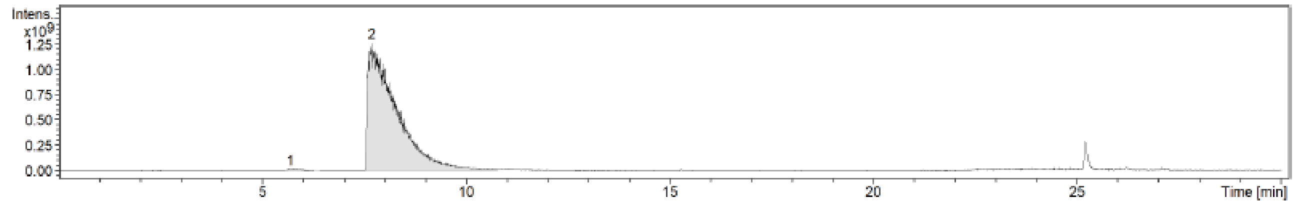

RT [min]	Area
5.69	426974720
7.69	59830595584

Cmpd 1, 5.69 min

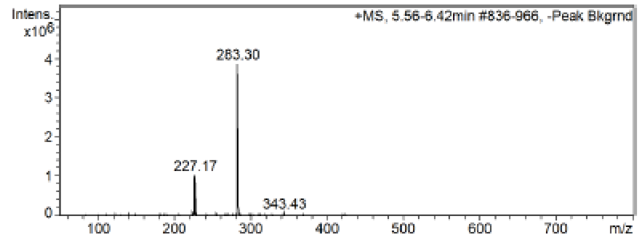

Cmpd 2, 7.69 min

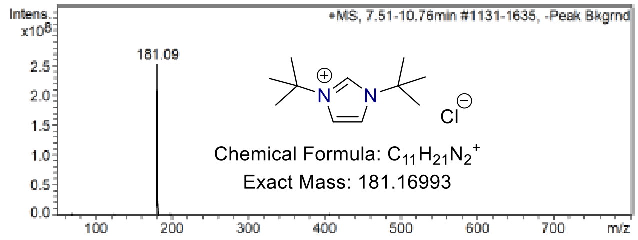

Figure S37. HPLC-ESI-MS of **I^{t}Bu-H$^+$**.

13. 1,3-Di-*tert*-butylimidazole-2-thione (I^{t}Bu-S)

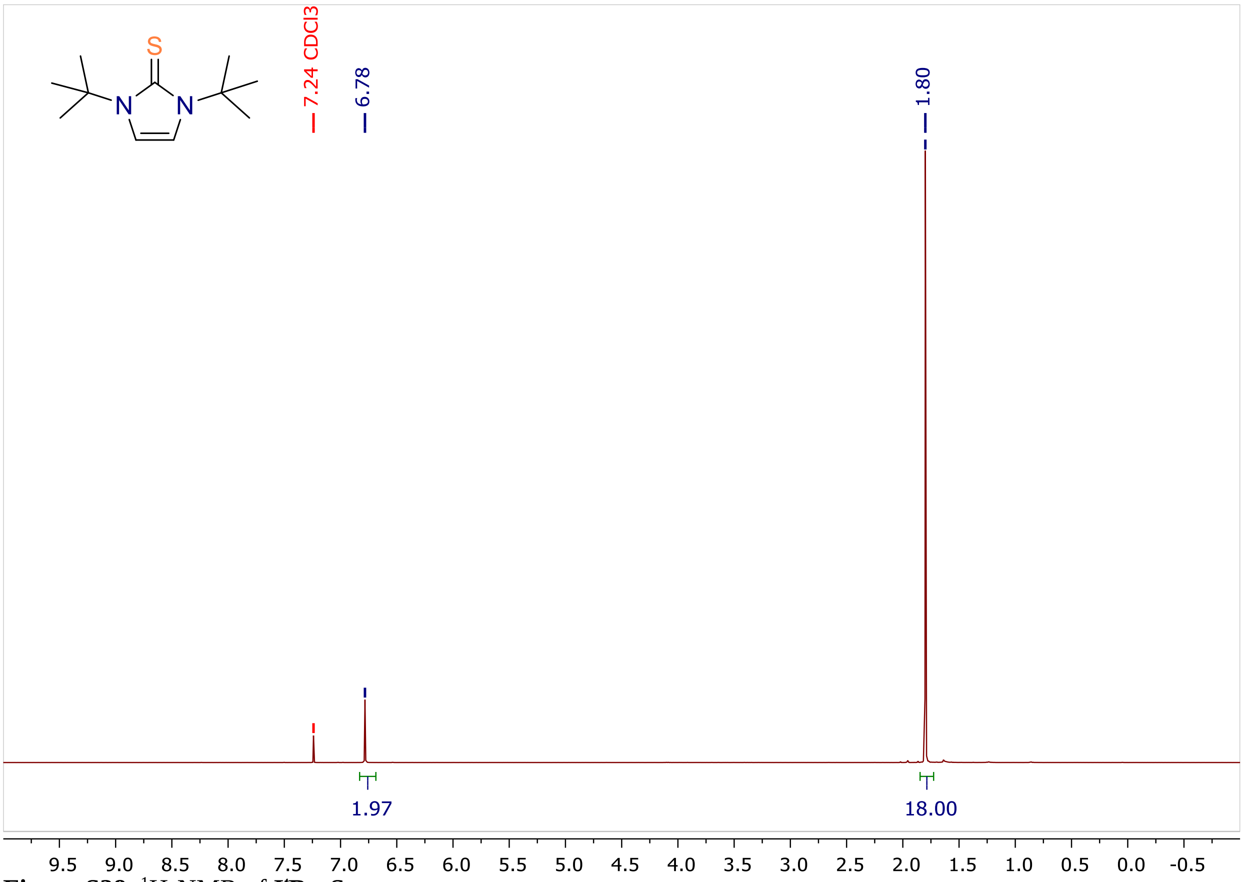

Figure S38. ^{1}H-NMR of **I^{t}Bu-S**.

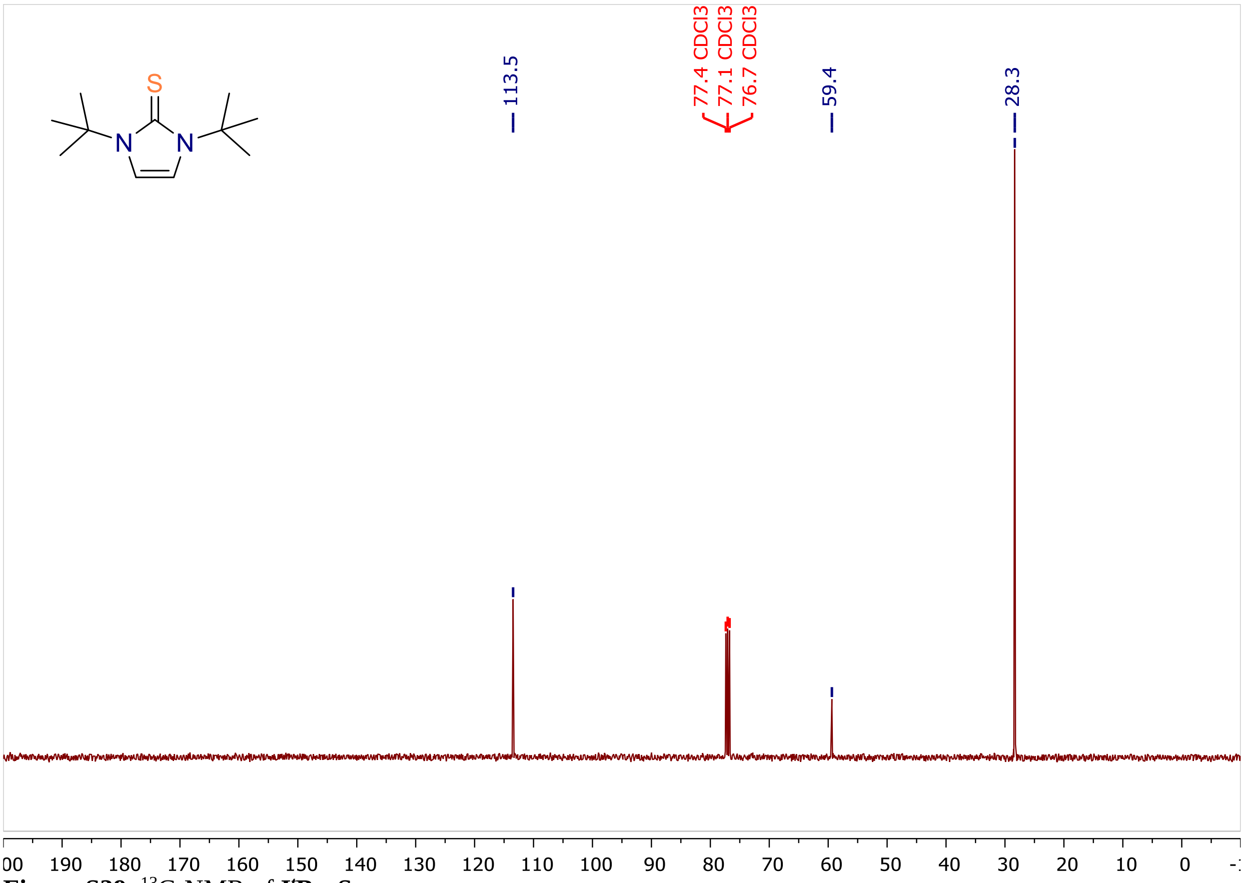

Figure S39. ^{13}C-NMR of **I^{t}Bu-S**.

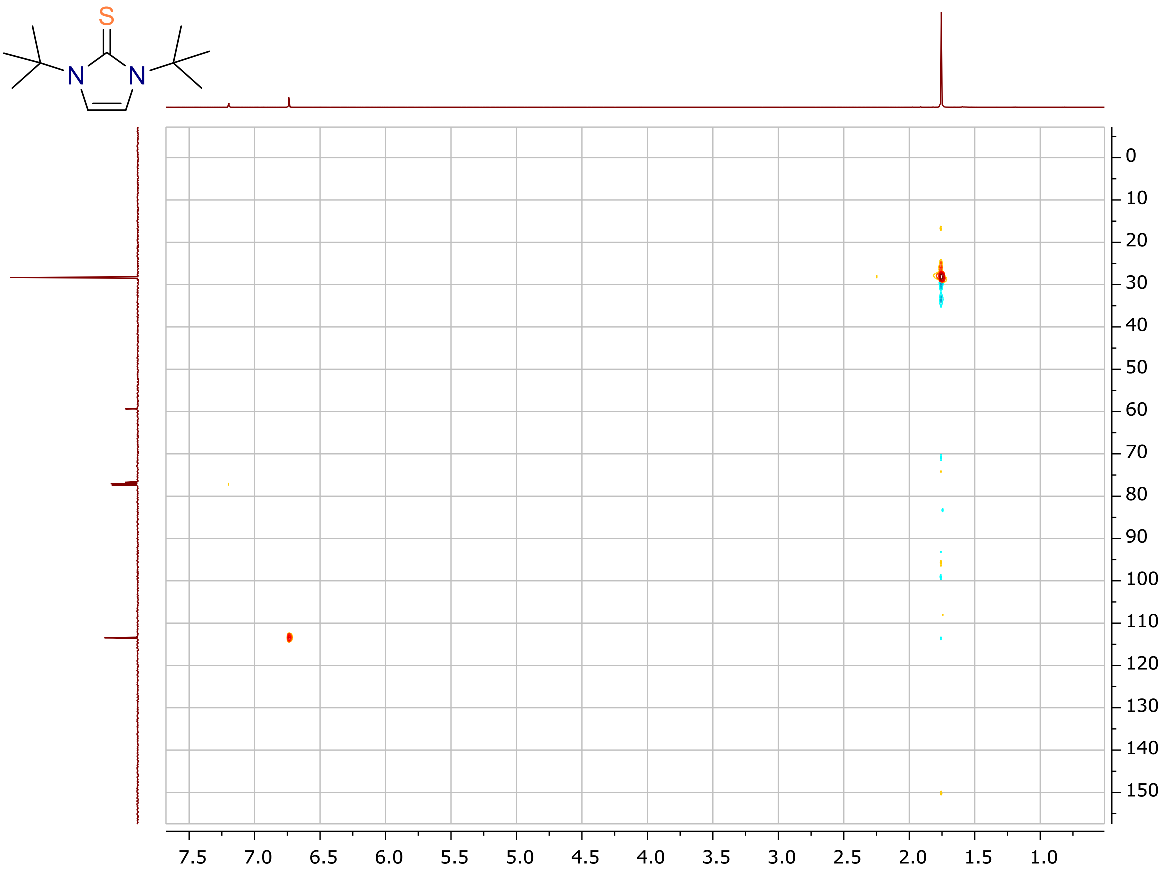

Figure S40. HSQC of **I^{t}Bu-S**.

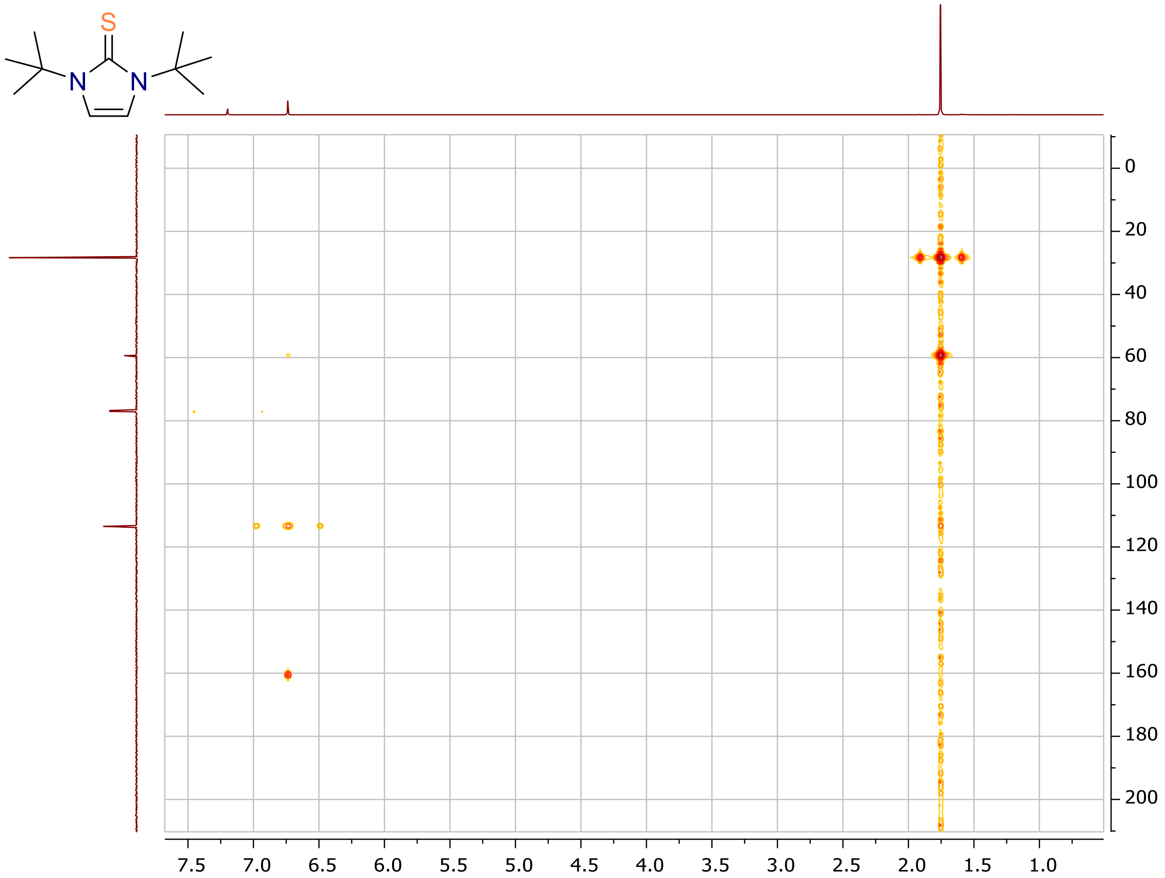

Figure S41. HMBC of **I^{t}Bu-S**.

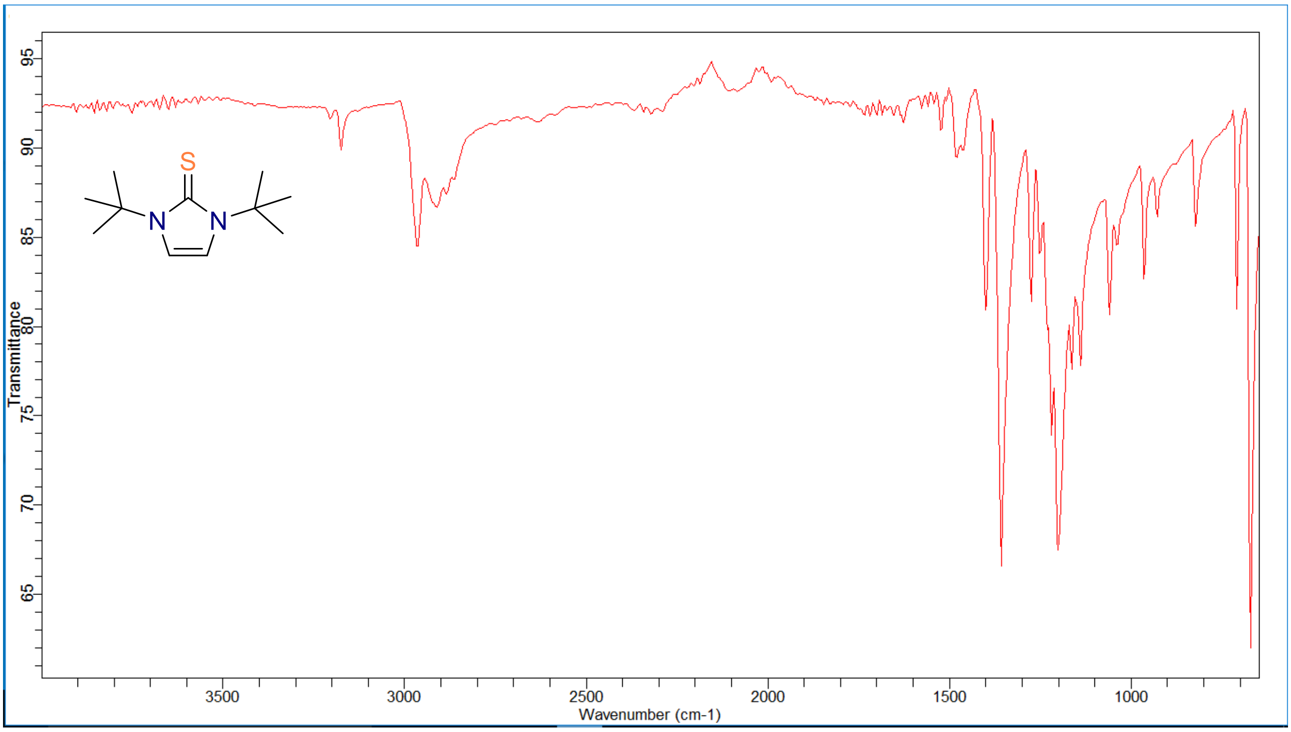

Figure S42. FTIR of **I^{t}Bu-S**.

Chromatograms

Fragmentor Voltage **Collision Energy** 0 **Ionization Mode** EI

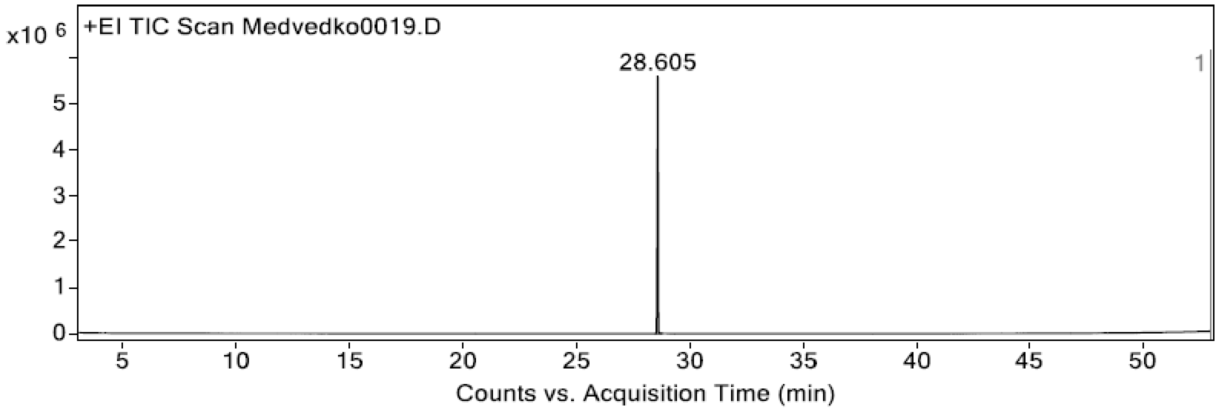

Integration Peak List

Peak	Start	RT	End	Height	Area	AreaSumPercent
1	28.52	28.605	28.768	5618064.34	16279000.12	100

Spectra

Spectrum Source Peak (1) in "+ TIC Scan"
Collision Energy 0
Ionization Mode EI

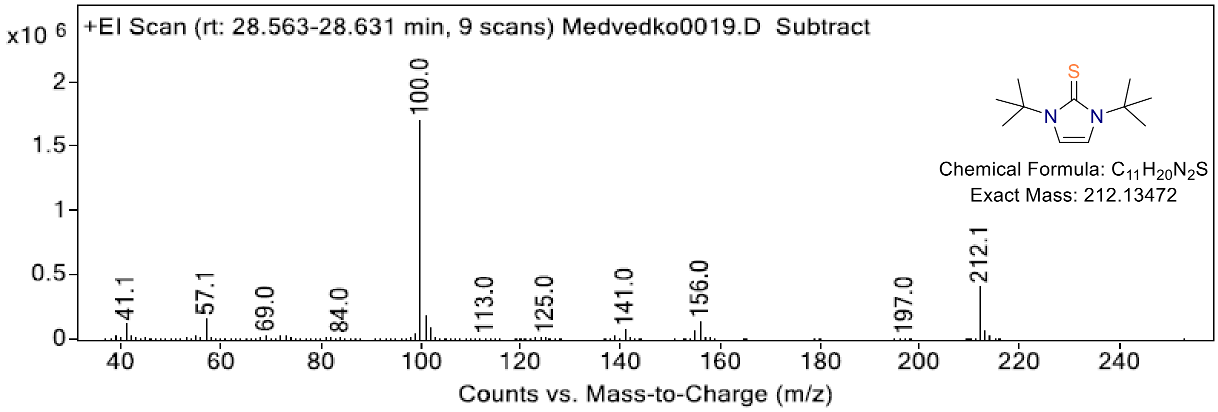

Figure S43. GC-MS of **I^{t}Bu-S**.

Analysis Info

Analysis Name	D:\Data\oi\Wagner_Medvedko_medv0621 HR_GC1_01_56968.d	Acquisition Date	5/11/2022 2:01:52 PM
Method	fia_ms_80-1000_pos_neu.m	Operator	BDAL@DE
Sample Name	Wagner_Medvedko_medv0621 HR	Instrument	maXis 288882.21253
Comment			

Acquisition Parameter

Source Type	ESI	Ion Polarity	Positive	Set Nebulizer	1.2 Bar
Focus	Not active	Set Capillary	4500 V	Set Dry Heater	200 °C
Scan Begin	80 m/z	Set End Plate Offset	-500 V	Set Dry Gas	6.0 l/min
Scan End	1100 m/z	Set Charging Voltage	0 V	Set Divert Valve	Waste
		Set Corona	0 nA	Set APCI Heater	0 °C

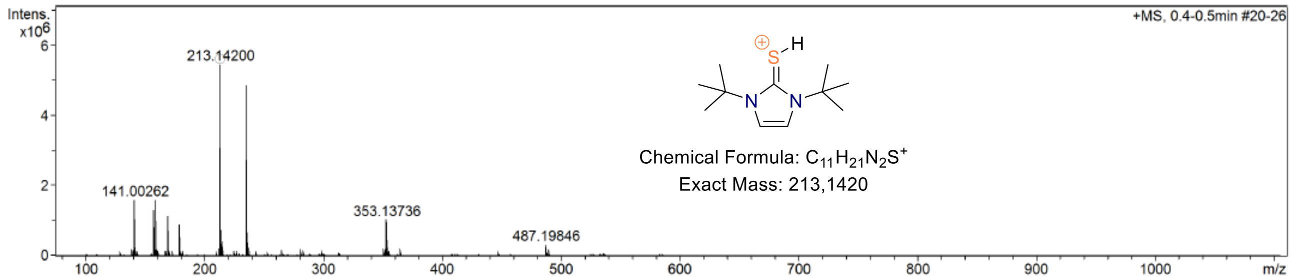

Chemical Formula: $C_{11}H_{21}N_2S^+$
Exact Mass: 213,1420

Meas. m/z	#	Ion Formula	m/z	err [ppm]	mSigma	# mSigma	Score	rdb	e⁻ Conf	N-Rule
213.14200	1	C11H21N2S	213.14200	-0.01	4.1	1	100.00	2.5	even	ok

Figure S44. ESI-MS of **I^{t}Bu-S**.

14. 1,3-Dimesitylimidazolium chloride (IMes-H$^+$)

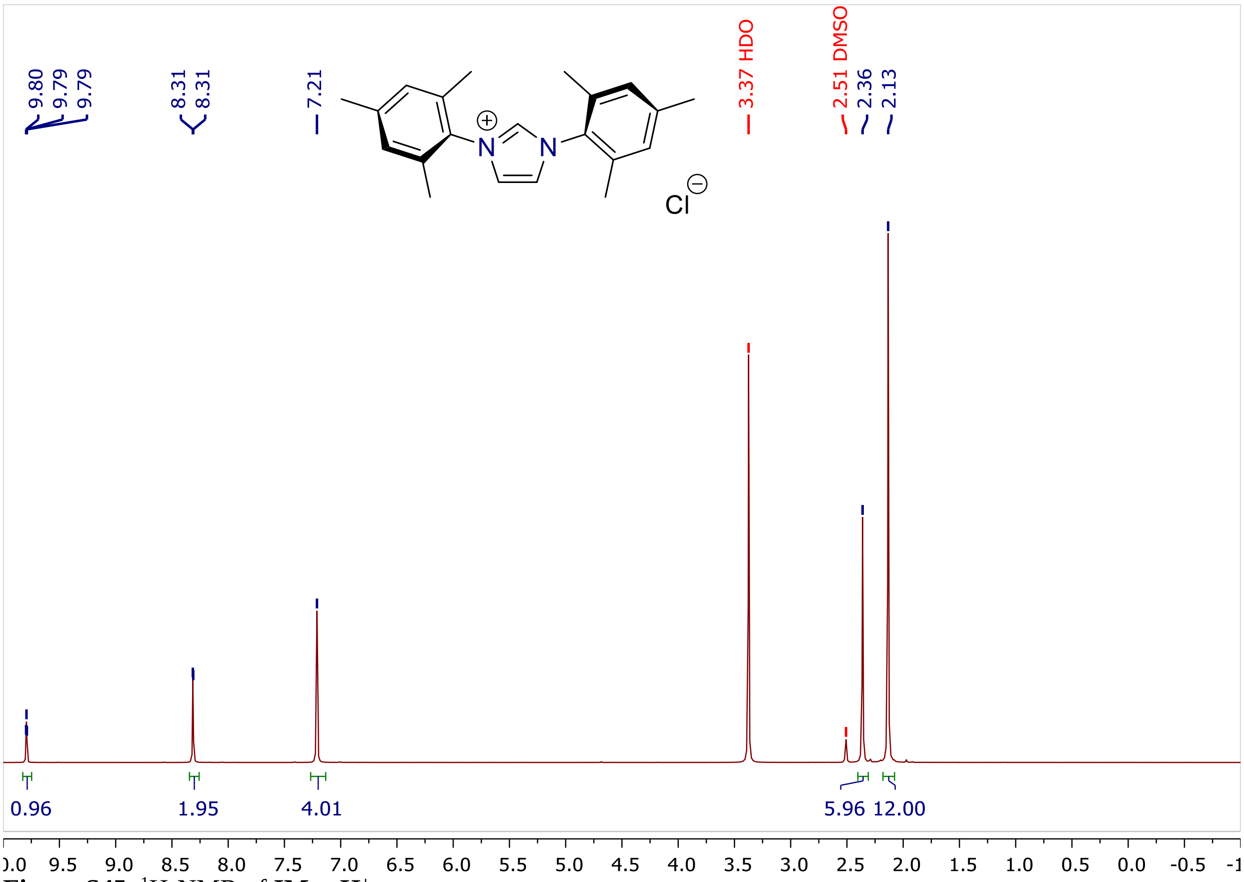

Figure S45. ^{1}H-NMR of **IMes-H$^+$**.

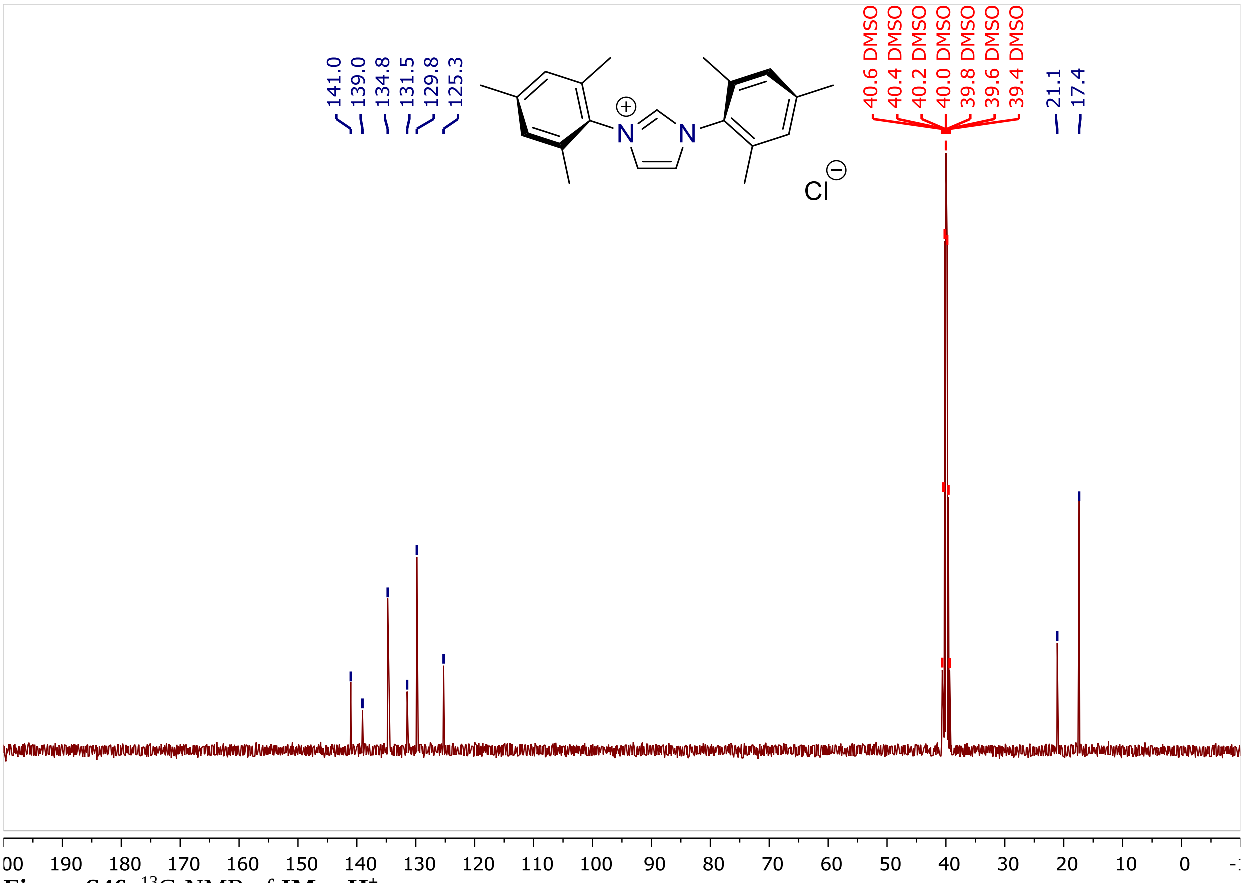

Figure S46. ^{13}C-NMR of **IMes-H$^+$**.

Acquisition Parameter

Ion Source Type	ESI	Ion Polarity	Positive	Alternating Ion Polarity	off
Mass Range Mode	UltraScan	Scan Begin	50 m/z	Scan End	800 m/z
Accumulation Time	1227 µs	RF Level	55 %	Trap Drive	46.0
SPS Target Mass	300 m/z	Averages	10 Spectra	n/a	n/a

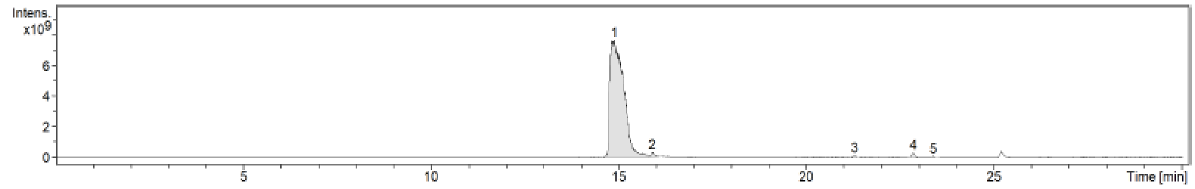

RT [min]	Area
14.89	197858410496
15.89	2056262912
21.28	647340416
22.85	1626171264
23.37	296186816

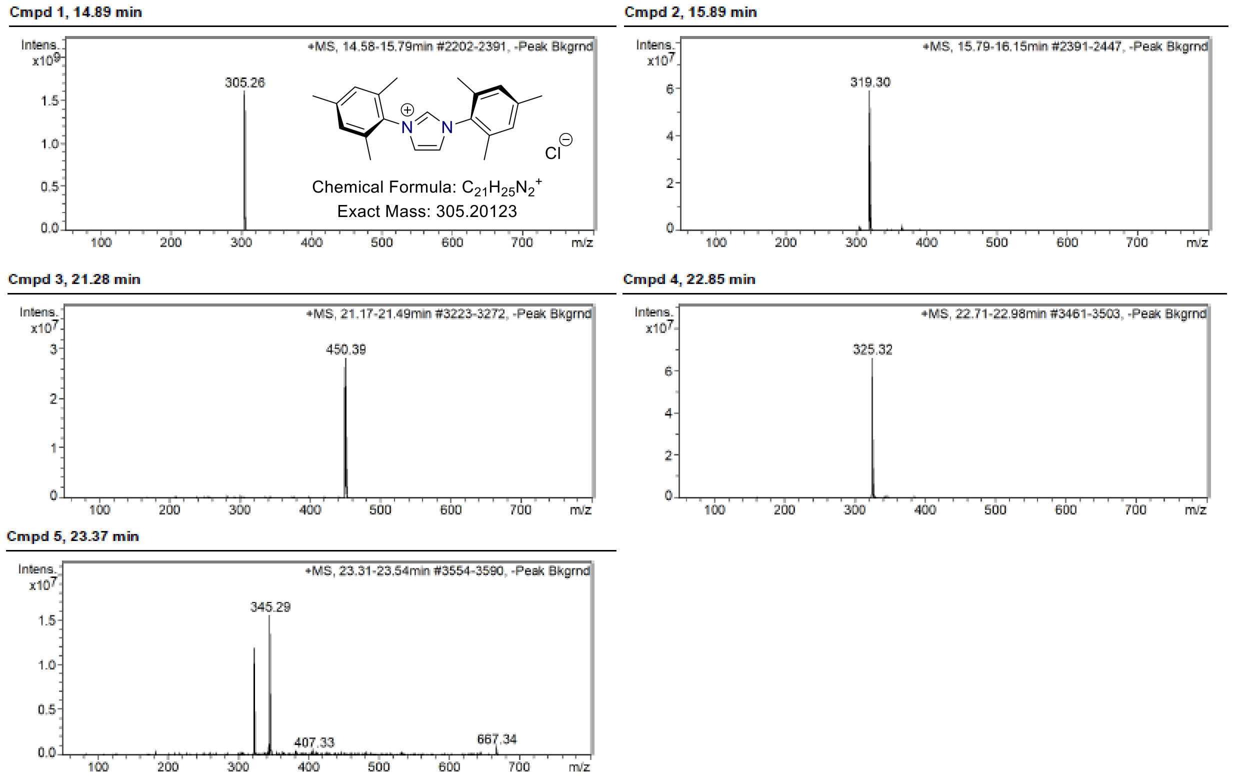

Figure S47. HPLC-ESI-MS of **IMes-H**$^+$.

15. 1,3-Dimesitylimidazole-2-thione (IMes-S)

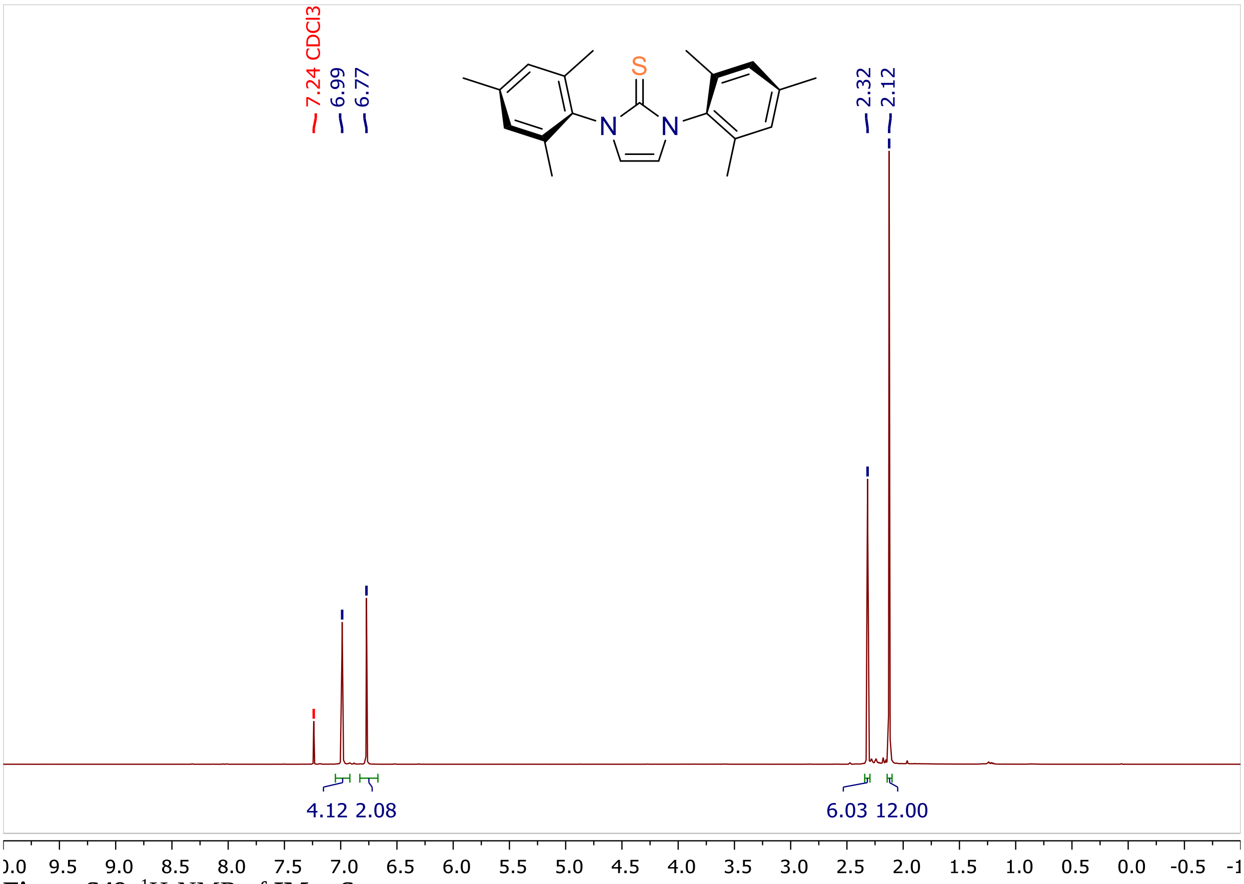

Figure S48. ^{1}H-NMR of **IMes-S**.

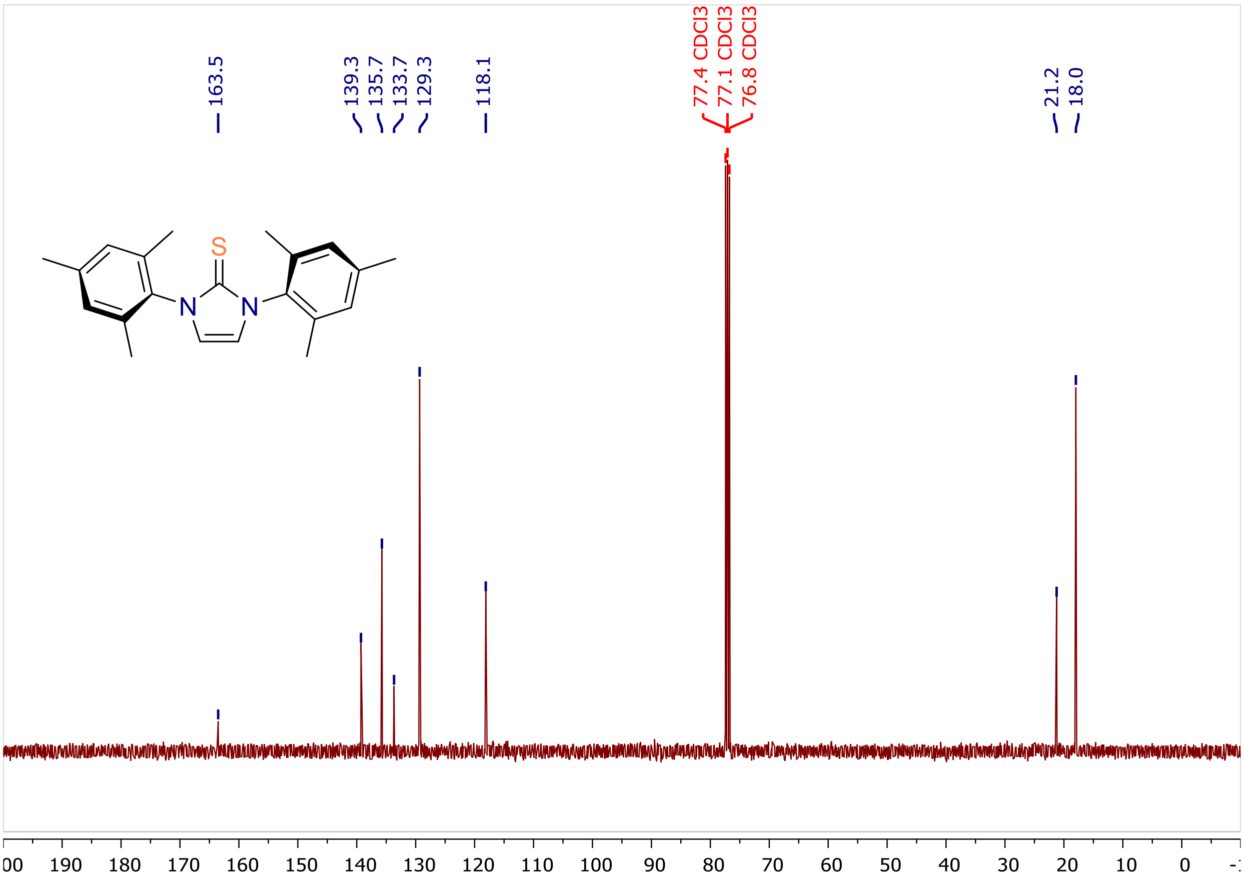

Figure S49. ^{13}C-NMR of **IMes-S**.

Chromatograms

Fragmentor Voltage **Collision Energy** 0 **Ionization Mode** EI

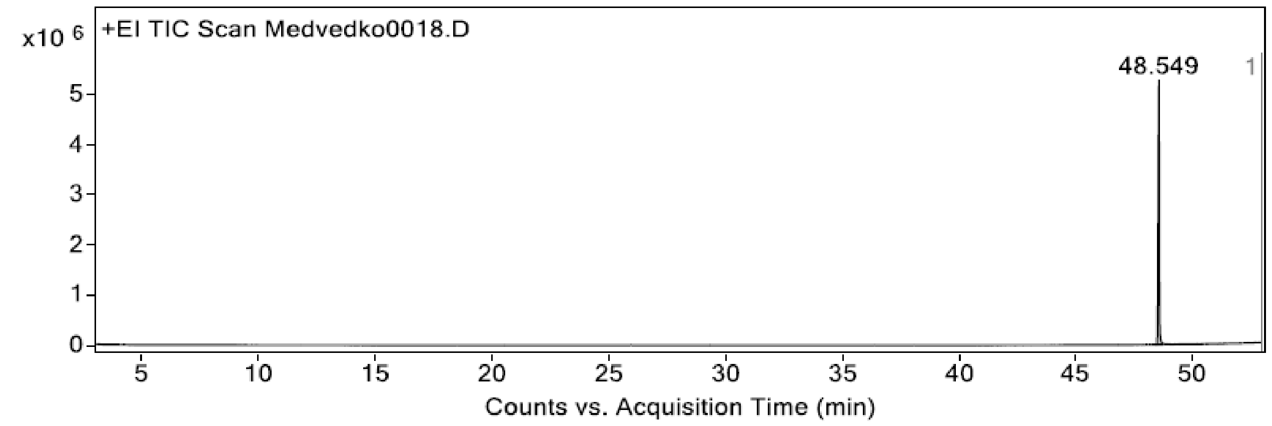

Integration Peak List

Peak	Start	RT	End	Height	Area	AreaSumPercent
1	48.438	48.549	48.712	5289690.09	19123898.45	100

Spectra

Spectrum Source Peak (1) in "+ TIC Scan" **Collision Energy** 0 **Ionization Mode** EI

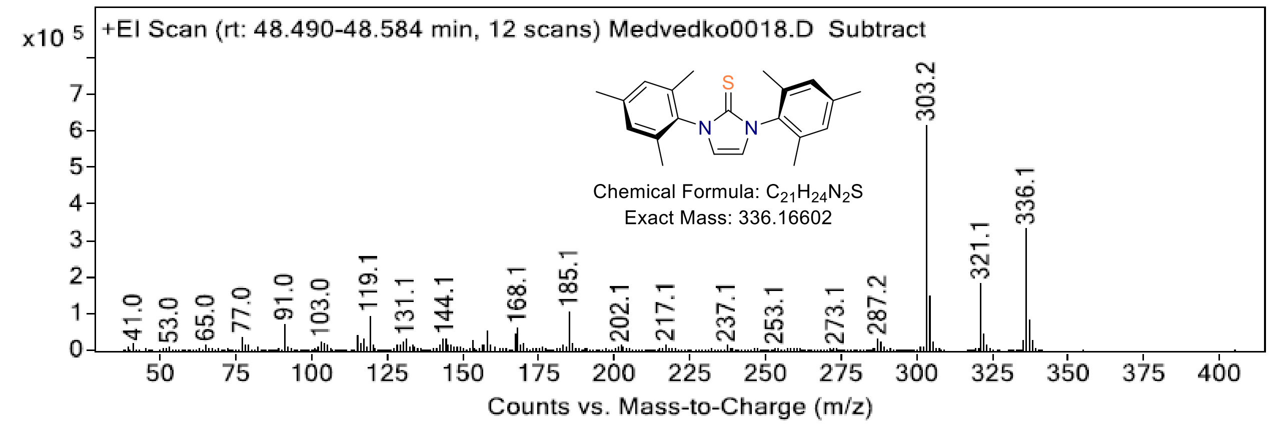

Figure S50. GC-MS of **IMes-S**.

16. 1,3-Bis(2,6-diisopropylphenyl)imidazolium chloride (IDipp-H$^+$)

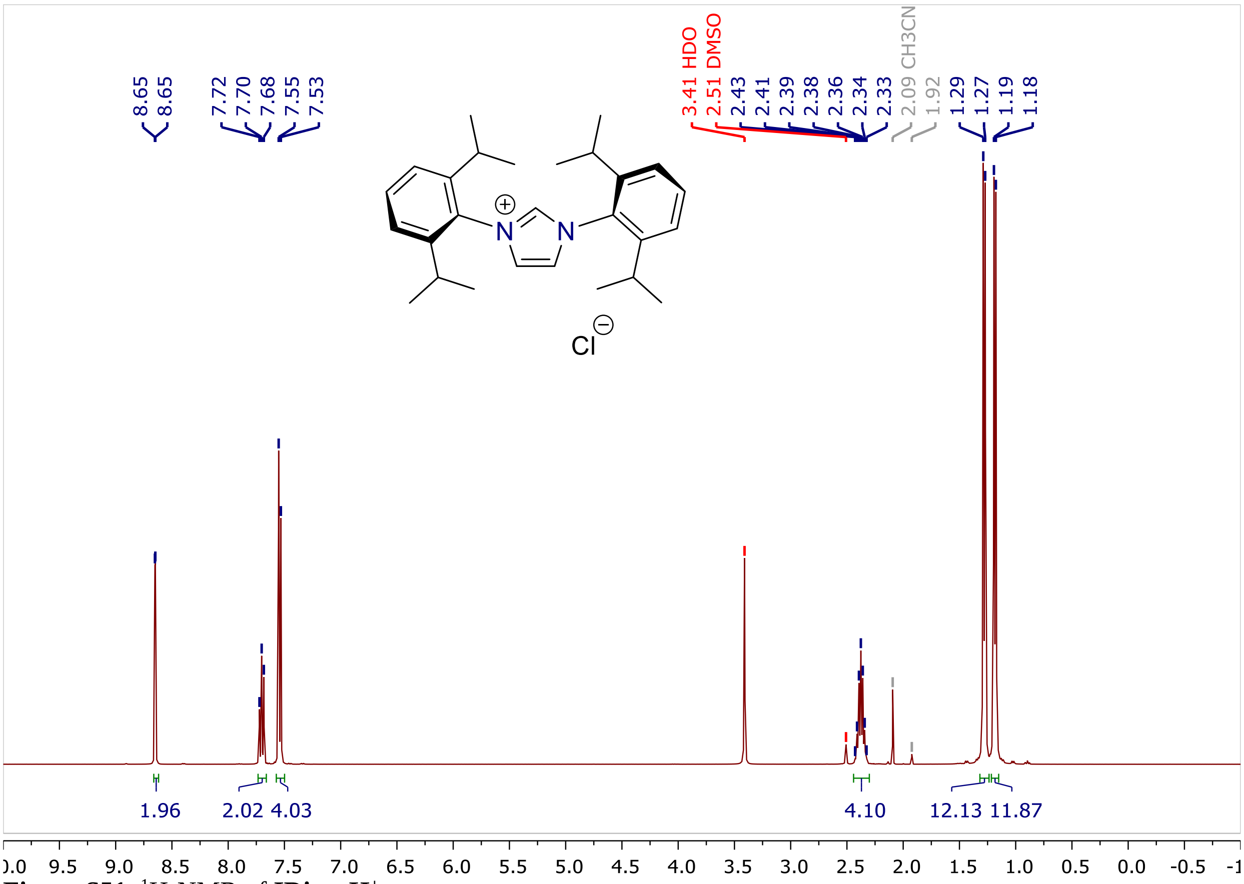

Figure S51. ^{1}H-NMR of **IDipp-H$^+$**.

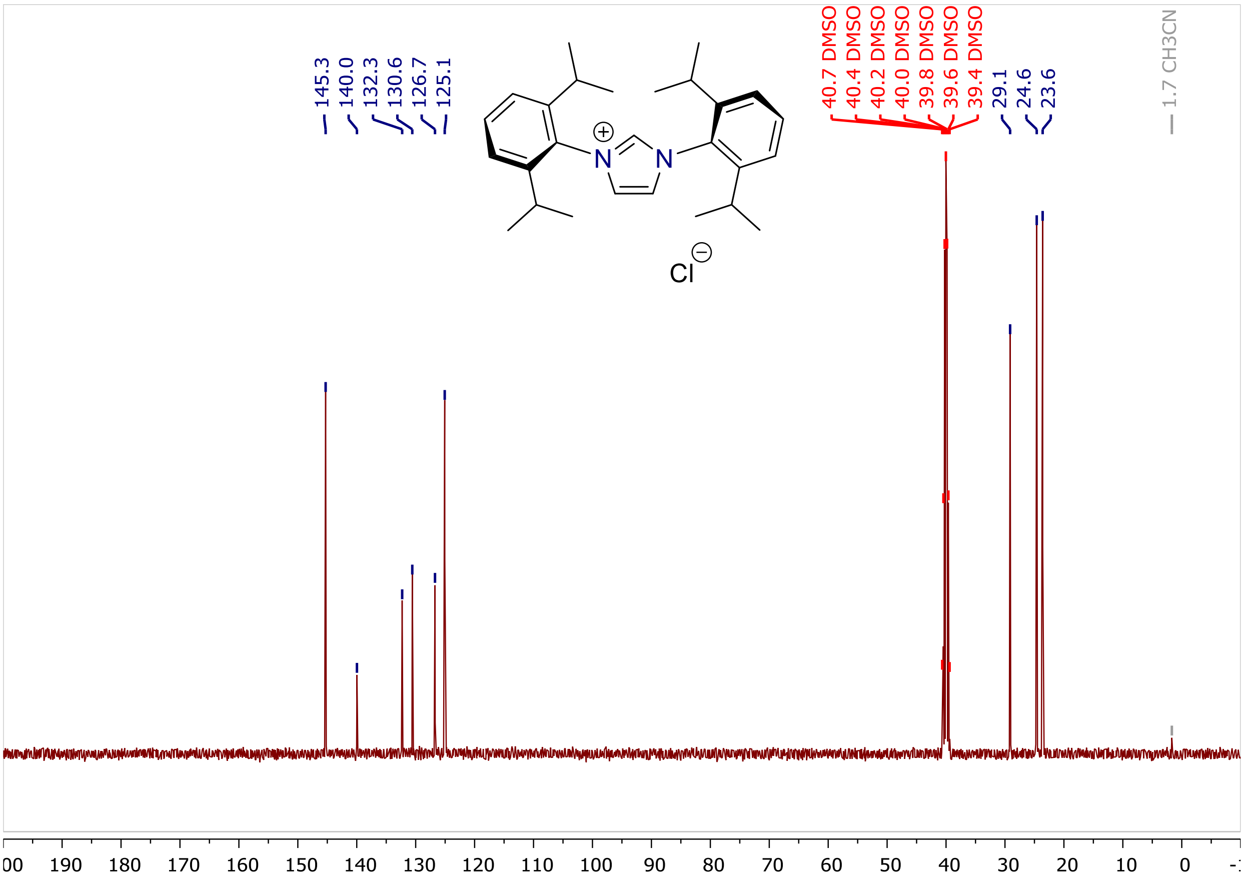

Figure S52. ^{13}C-NMR of **IDipp-H$^+$**.

Acquisition Parameter

Ion Source Type	ESI	Ion Polarity	Positive	Alternating Ion Polarity	off
Mass Range Mode	UltraScan	Scan Begin	50 m/z	Scan End	800 m/z
Accumulation Time	1184 µs	RF Level	63 %	Trap Drive	49.7
SPS Target Mass	400 m/z	Averages	10 Spectra	n/a	n/a

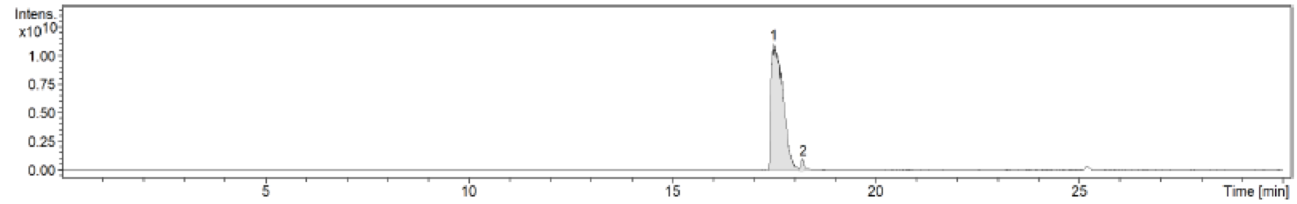

RT [min]	Area
17.50	225381236736
18.21	6090550272

Cmpd 1, 17.50 min

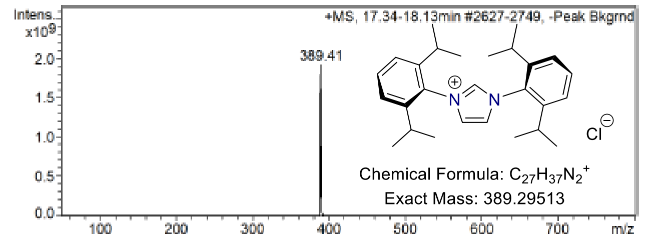

Cmpd 2, 18.21 min

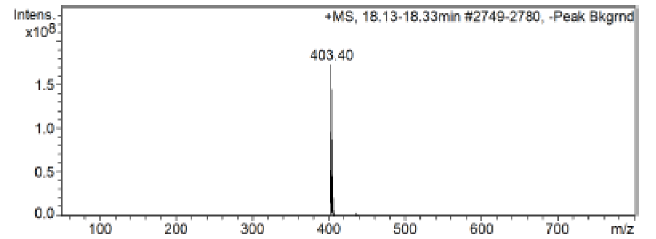

Figure S53. HPLC-ESI-MS of **IDipp-H$^+$**.

17. 1,3-Bis(2,6-diisopropylphenyl)imidazole-2-thione (IDipp-S)

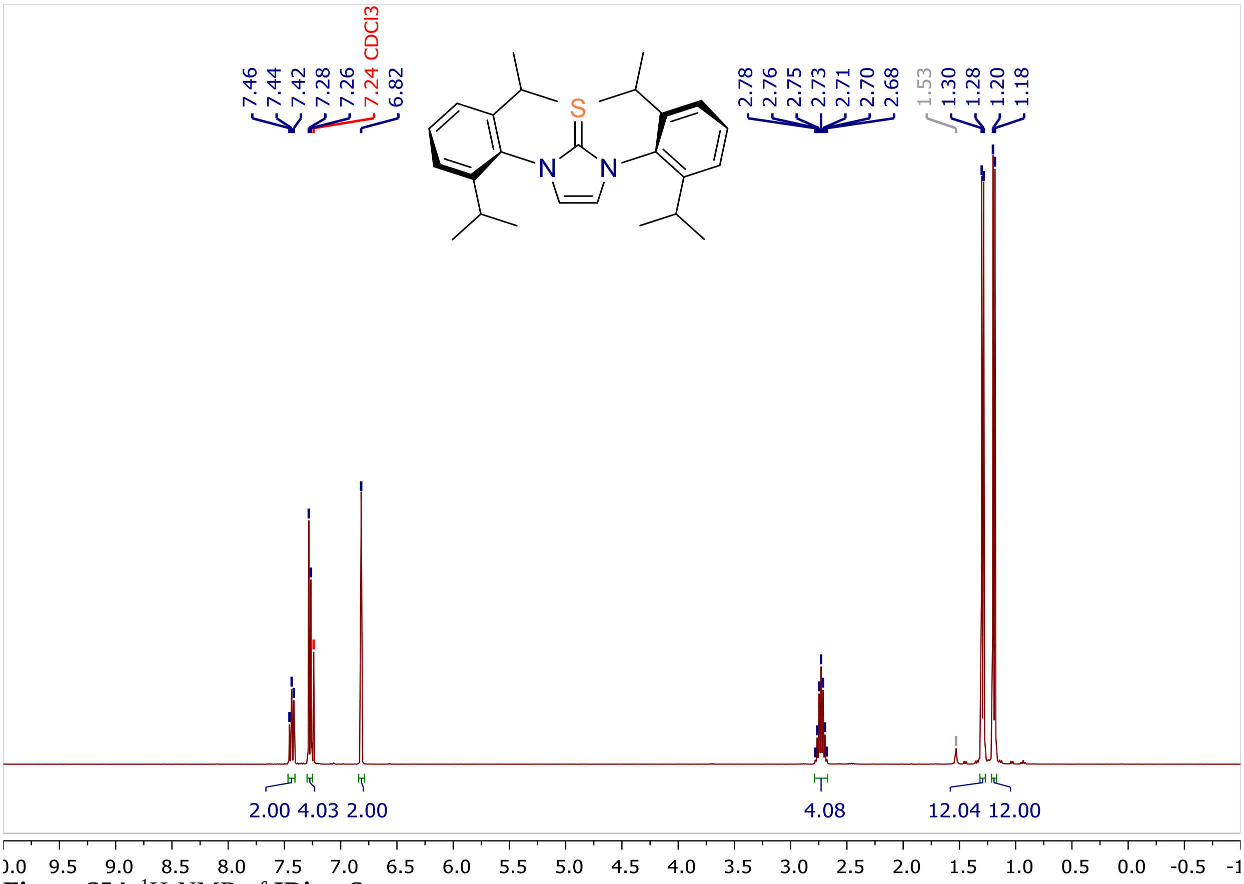

Figure S54. ^{1}H-NMR of **IDipp-S**.

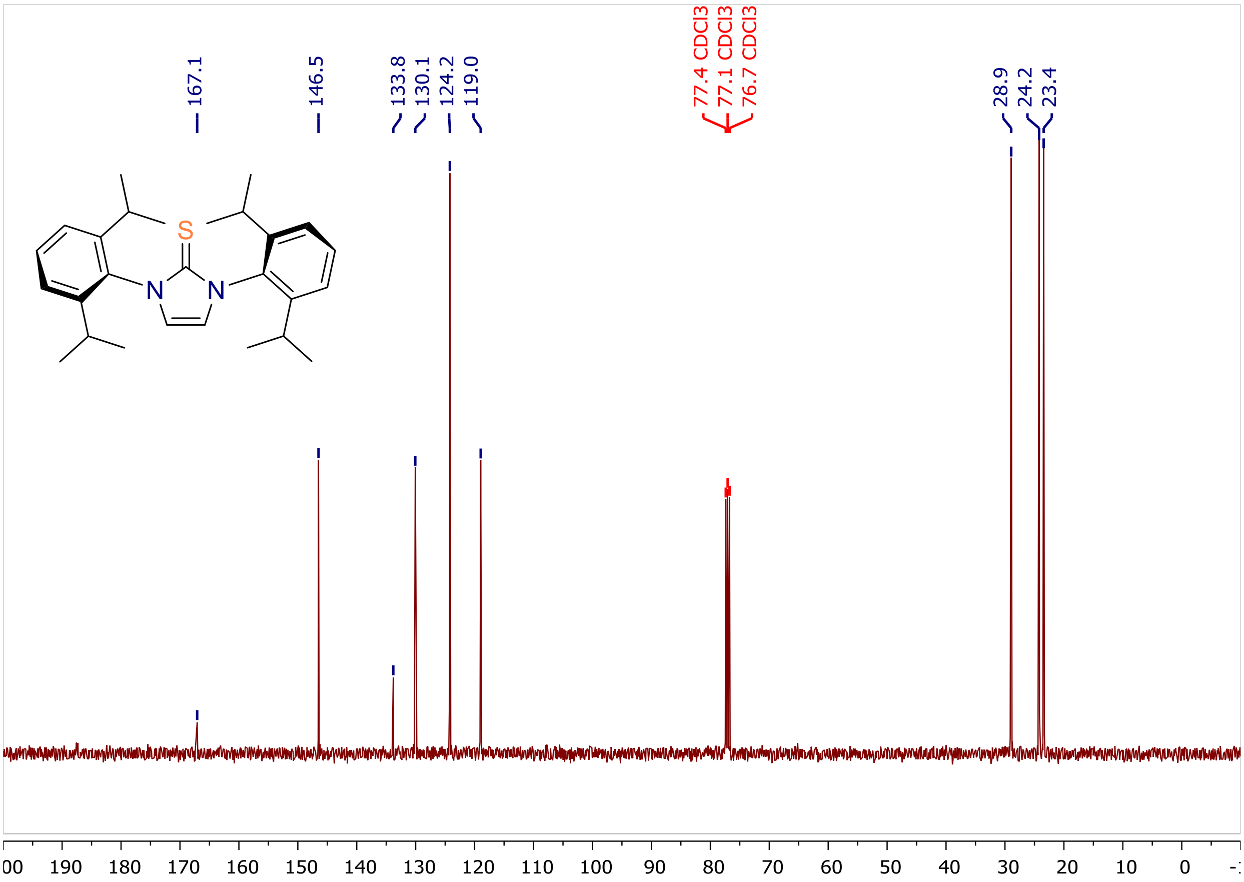

Figure S55. ^{13}C-NMR of **IDipp-S**.

Acquisition Parameter

Ion Source Type	ESI	Ion Polarity	Positive	Alternating Ion Polarity	off
Mass Range Mode	UltraScan	Scan Begin	50 m/z	Scan End	800 m/z
Accumulation Time	1159 µs	RF Level	63 %	Trap Drive	49.7
SPS Target Mass	400 m/z	Averages	10 Spectra	n/a	n/a

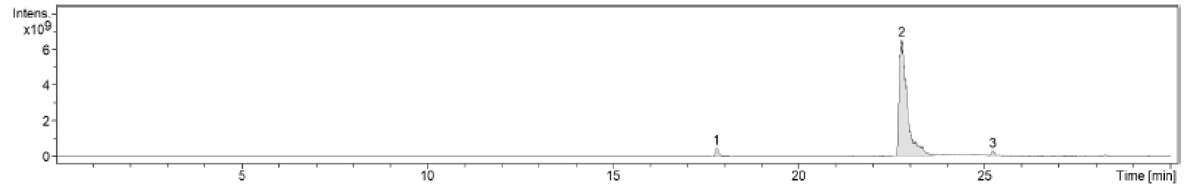

RT [min]	Area
17.78	3111915776
22.77	99610517504
25.22	1619024768

Cmpd 1, 17.78 min

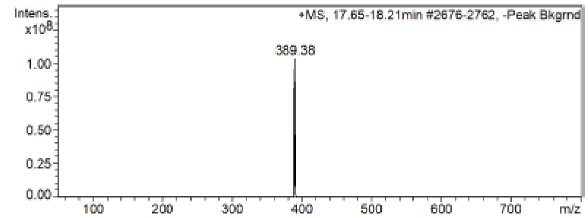

Cmpd 2, 22.77 min

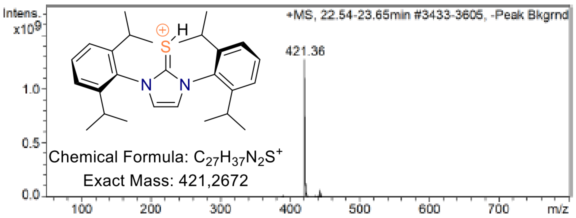

Cmpd 3, 25.22 min

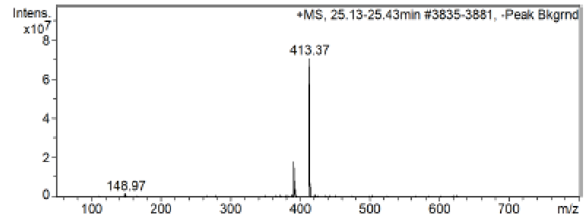

Figure S56. HPLC-ESI-MS of **IDipp-S**.

18. IMe-SO_3

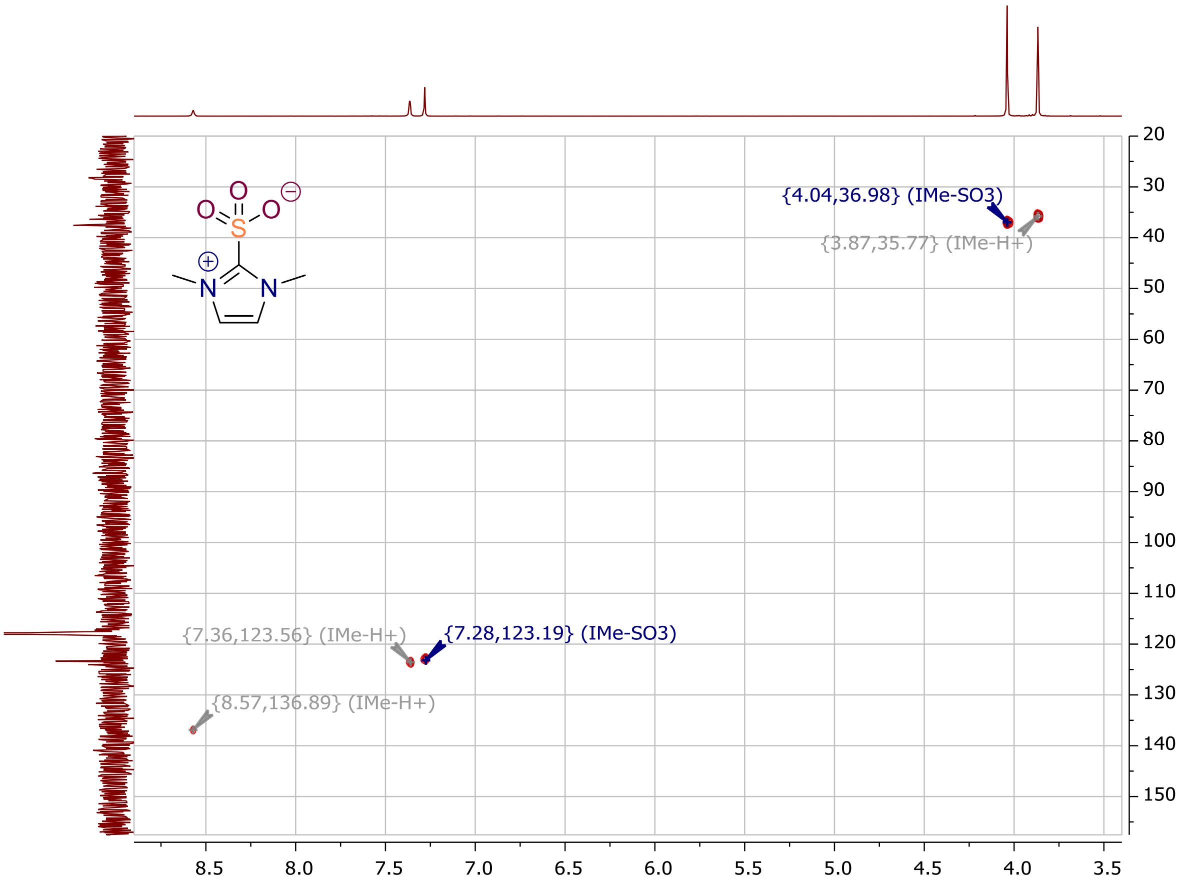

Figure S57. HSQC of **IMe-SO_3**.

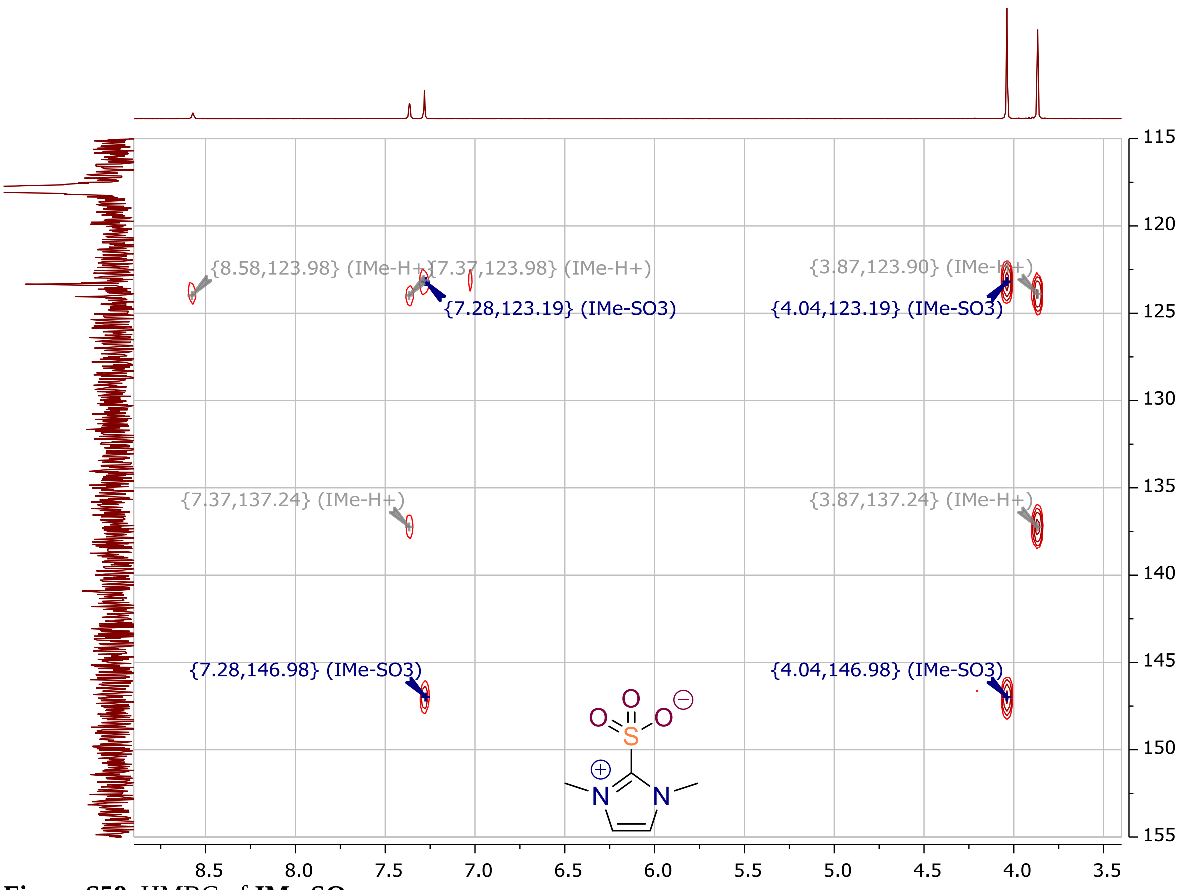

Figure S58. HMBC of **IMe-SO_3**.

Mass Spectrum SmartFormula Report

Analysis Info

Analysis Name	D:\Data\oi\Wagner_Medvedko_medv0600 HR_GC2_01_56975.d	Acquisition Date	5/11/2022 3:09:45 PM
Method	fia_ms_80-1000_pos_neu.m	Operator	BDAL@DE
Sample Name	Wagner_Medvedko_medv0600 HR	Instrument	maXis 288882.21253
Comment			

Acquisition Parameter

Source Type	ESI	Ion Polarity	Positive	Set Nebulizer	1.2 Bar
Focus	Not active	Set Capillary	4500 V	Set Dry Heater	200 °C
Scan Begin	80 m/z	Set End Plate Offset	-500 V	Set Dry Gas	6.0 l/min
Scan End	1100 m/z	Set Charging Voltage	0 V	Set Divert Valve	Waste
		Set Corona	0 nA	Set APCI Heater	0 °C

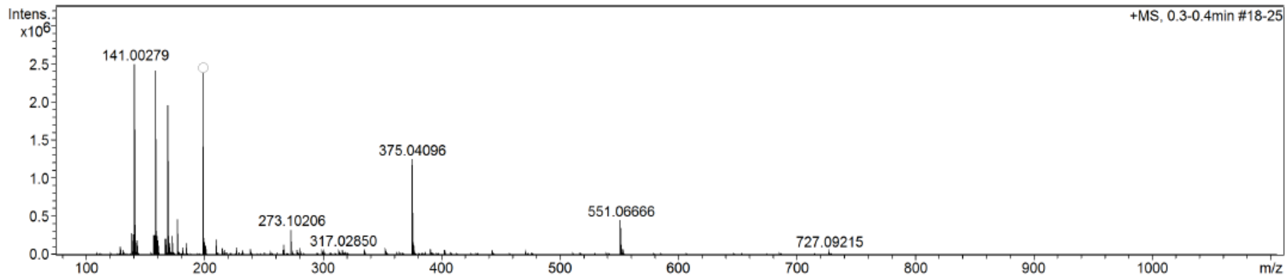

Meas. m/z	#	Ion Formula	m/z	err [ppm]	mSigma	# mSigma	Score	rdb	e⁻ Conf	N-Rule
199.01514	1	C5H8N2NaO3S	199.01478	-1.78	3.3	1	100.00	2.5	even	ok

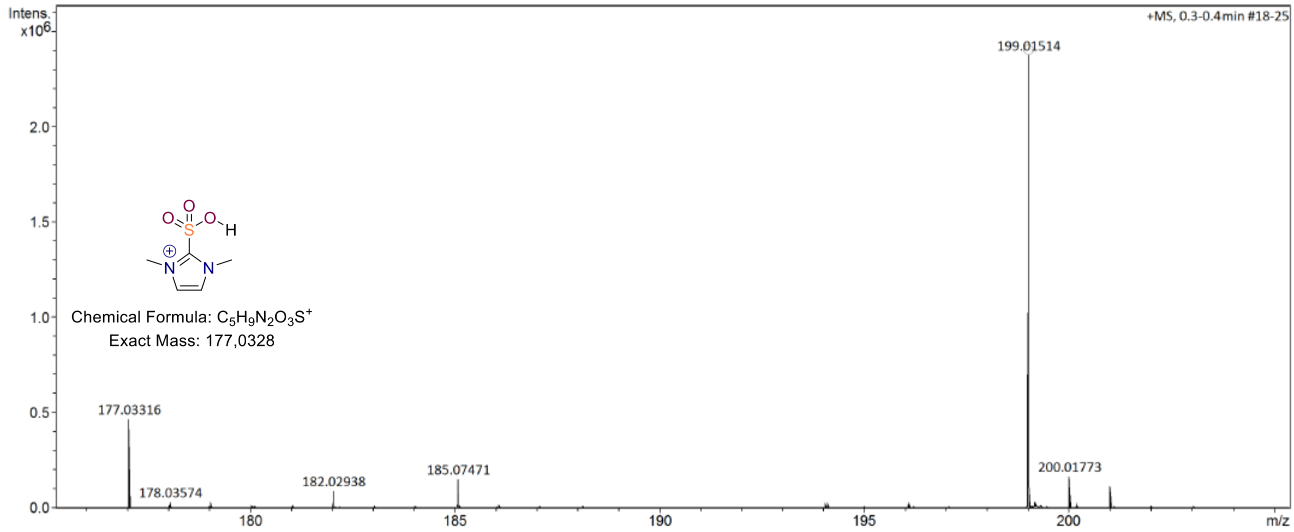

Figure S59. ESI-MS of **IMe-SO₃**.

19. SI^{t}Bu-SO

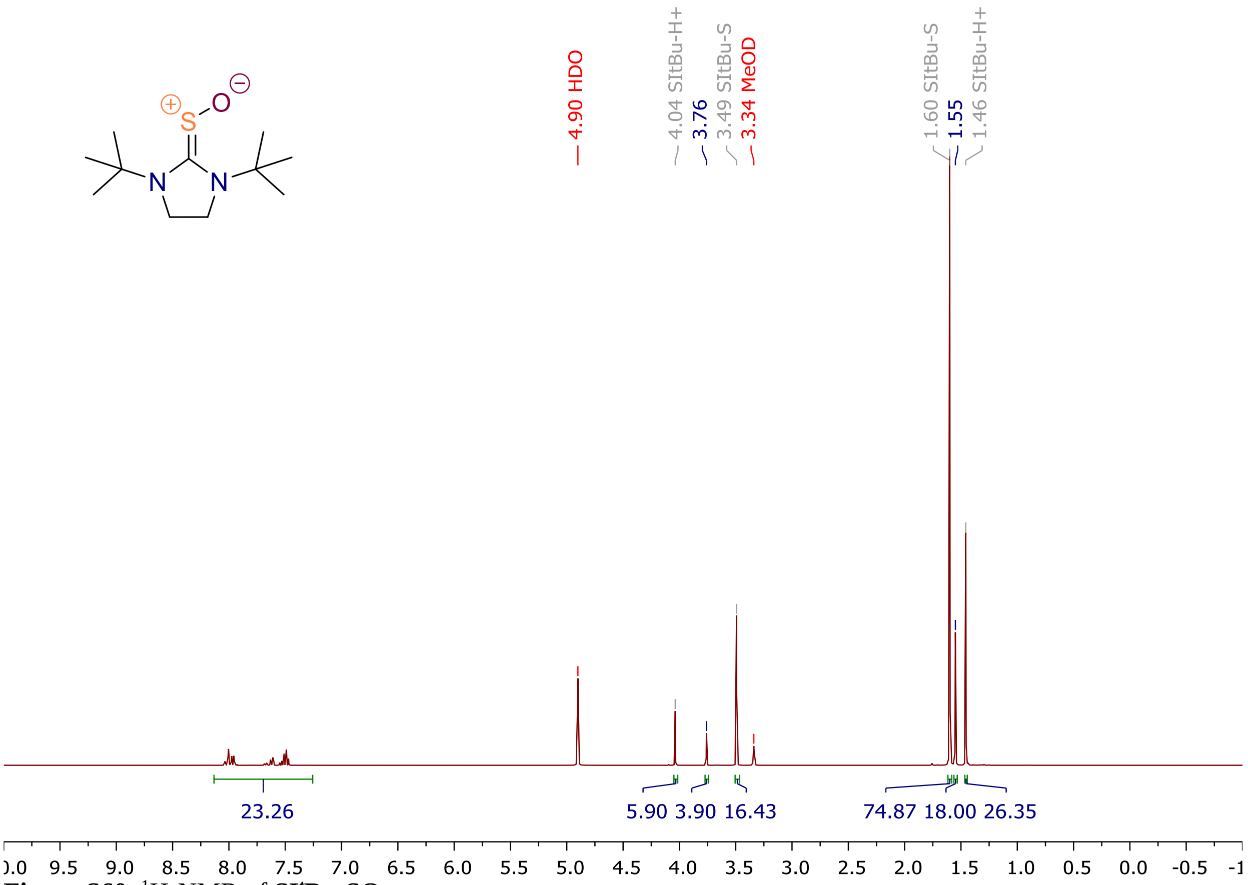

Figure S60. ^{1}H-NMR of **SI^{t}Bu-SO**.

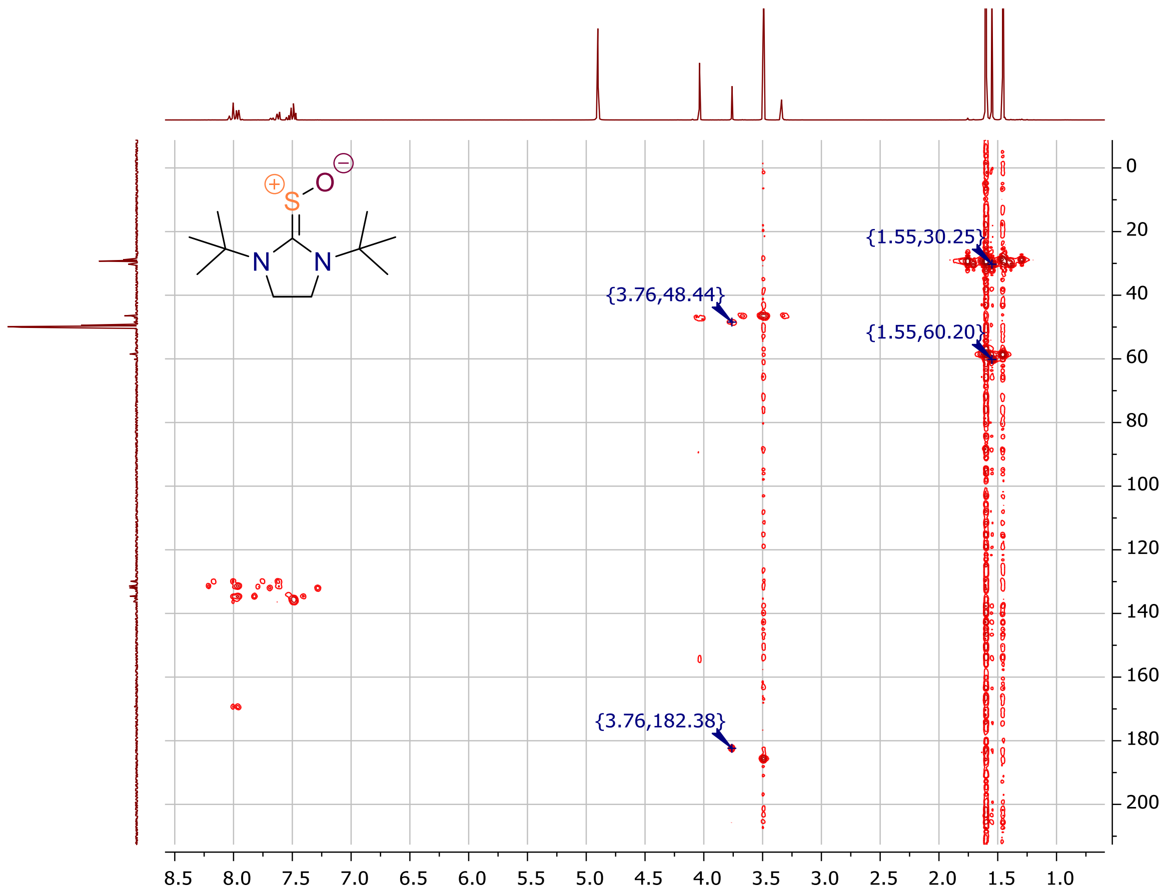

Figure S61. HMBC of **SI^{t}Bu-SO**.

Analysis Info

Analysis Name	D:\Data\oi\HPLC-MS\Wagner\Medvedko\medv0767_P2-B2_01_4314.d	Acquisition Date	6/21/2022 10:20:30 AM
Method	4314.m	Operator	user
Sample Name	medv0767	Instrument	amaZon SL
Comment			

Acquisition Parameter

Ion Source Type	ESI	Ion Polarity	Positive	Alternating Ion Polarity	off
Mass Range Mode	UltraScan	Scan Begin	50 m/z	Scan End	800 m/z
Accumulation Time	407 µs	RF Level	48 %	Trap Drive	43.3
SPS Target Mass	230 m/z	Averages	10 Spectra	n/a	n/a

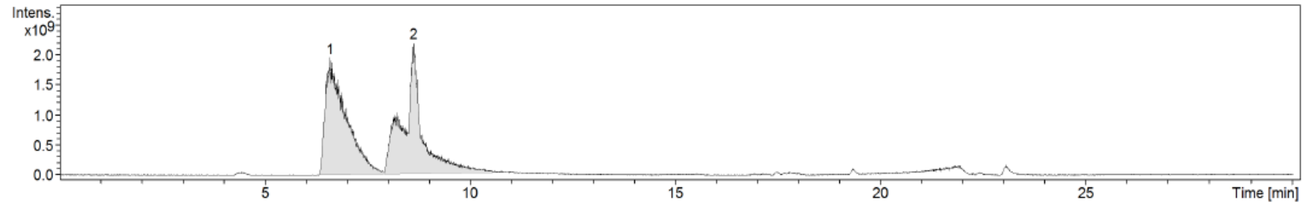

RT [min]	Area
6.59	66595086336
8.63	68683571200

Cmpd 1, 6.59 min

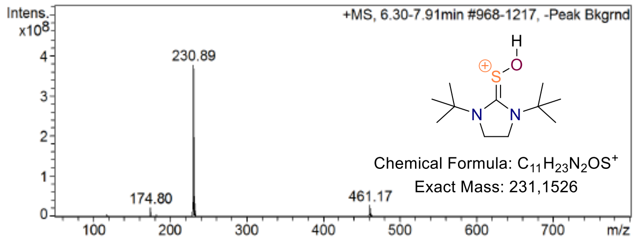

Cmpd 2, 8.63 min

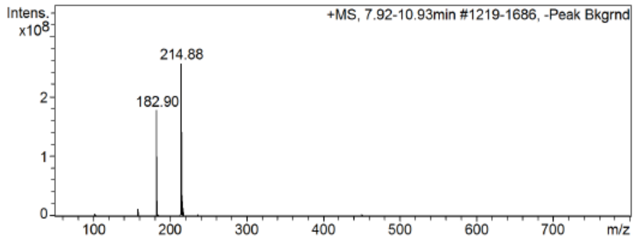

Figure S62. HPLC-ESI-MS of **SI^{t}Bu-SO**.

Mass Spectrum SmartFormula Report

Analysis Info

Analysis Name	D:\Data\oi\Medvedko_medv0767_GA3_01_57968.d	Acquisition Date	7/7/2022 11:48:26 AM	
Method	fia_ms_80-1000_pos_neu.m	Operator	BDAL@DE	
Sample Name	Medvedko_medv0767	Instrument	maXis	288882.21253
Comment				

Acquisition Parameter

Source Type	ESI	Ion Polarity	Positive	Set Nebulizer	1.2 Bar
Focus	Not active	Set Capillary	4500 V	Set Dry Heater	200 °C
Scan Begin	80 m/z	Set End Plate Offset	-500 V	Set Dry Gas	6.0 l/min
Scan End	1000 m/z	Set Charging Voltage	0 V	Set Divert Valve	Waste
		Set Corona	0 nA	Set APCI Heater	0 °C

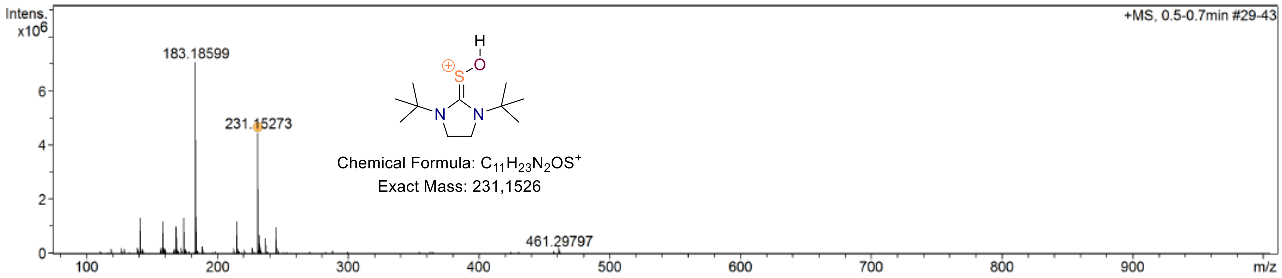

Meas. m/z	#	Ion Formula	m/z	err [ppm]	mSigma	# mSigma	Score	rdb	e⁻ Conf	N-Rule
231.15273	1	C11H23N2OS	231.15256	-0.74	6.7	1	100.00	1.5	even	ok

Figure S63. ESI-MS of **SI^{t}Bu-SO**.

20. SIMes-SO

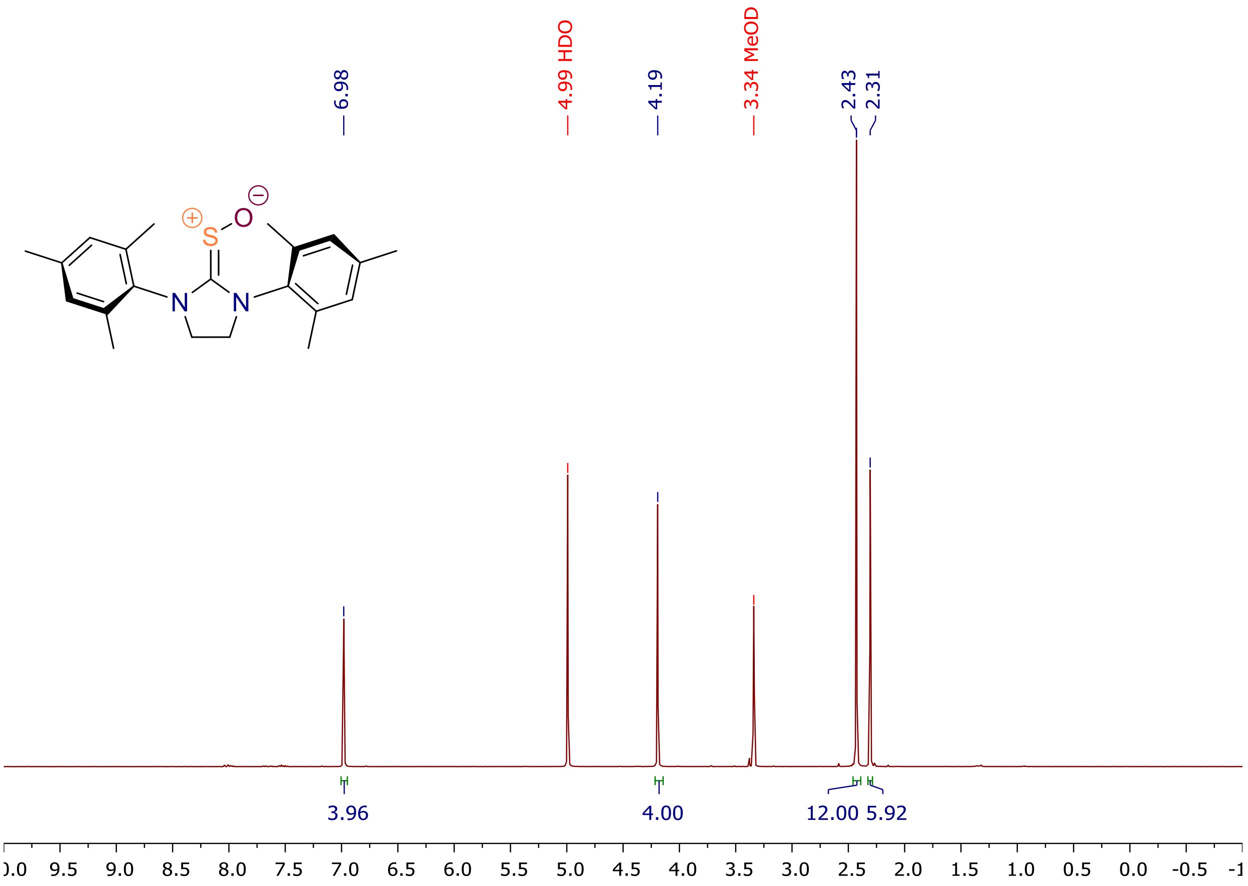

Figure S64. ^{1}H-NMR of **SIMes-SO**.

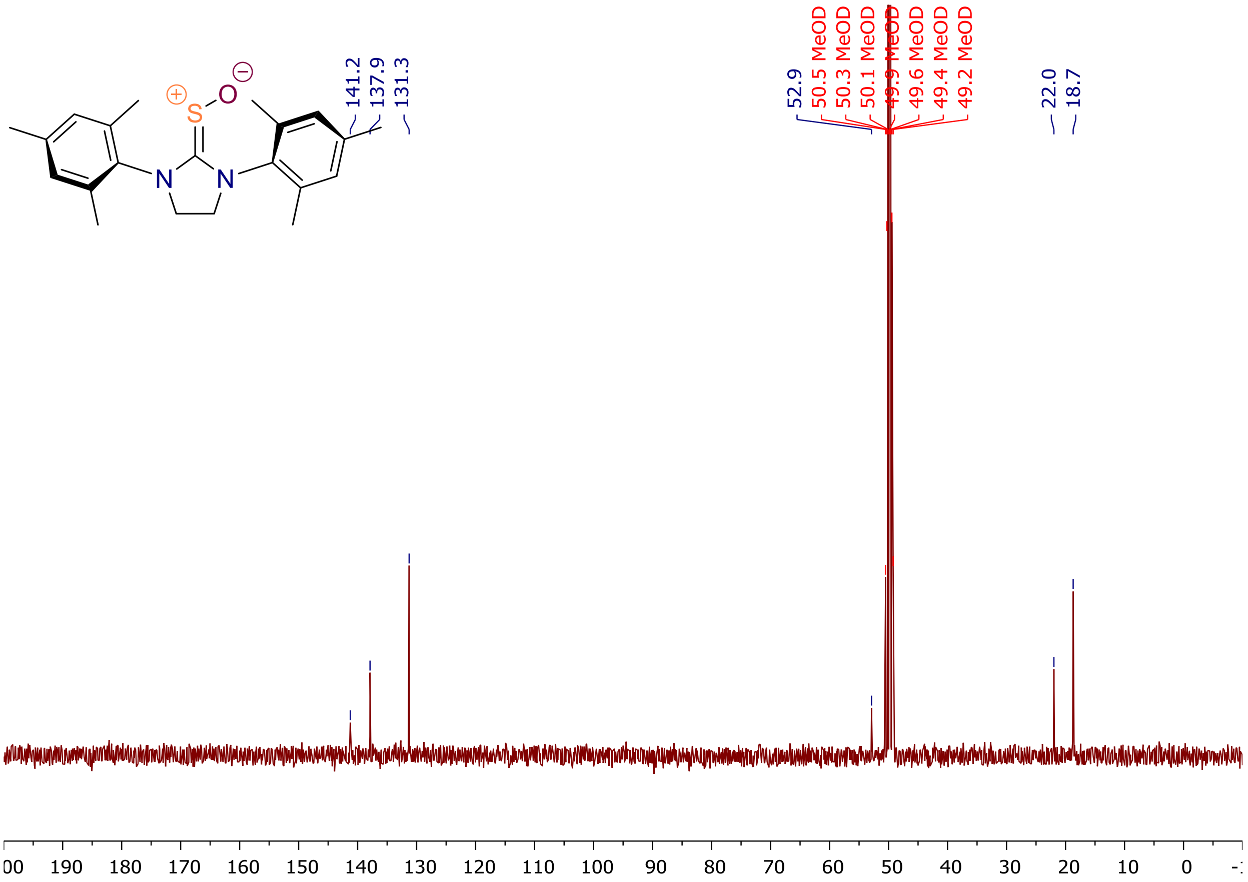

Figure S65. ^{13}C-NMR of **SIMes-SO**.

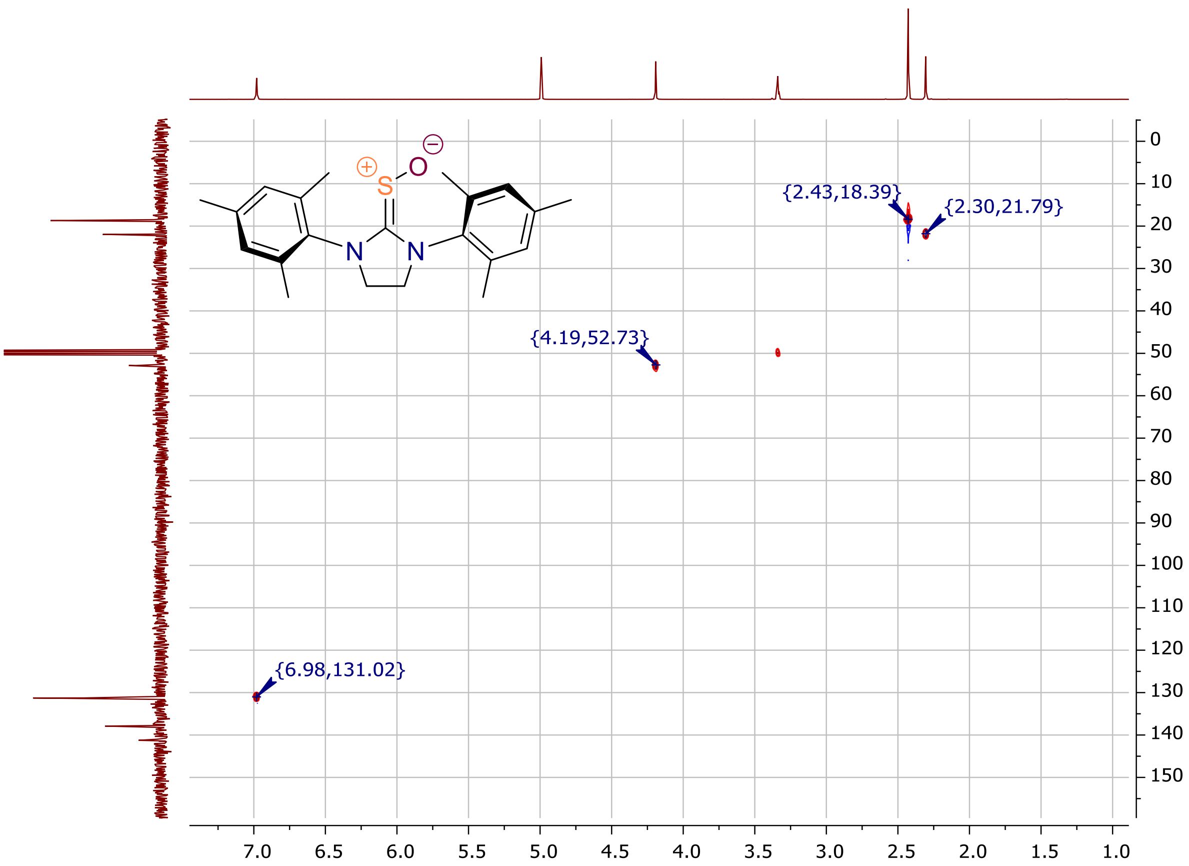

Figure S66. HSQC of **SIMes-SO**.

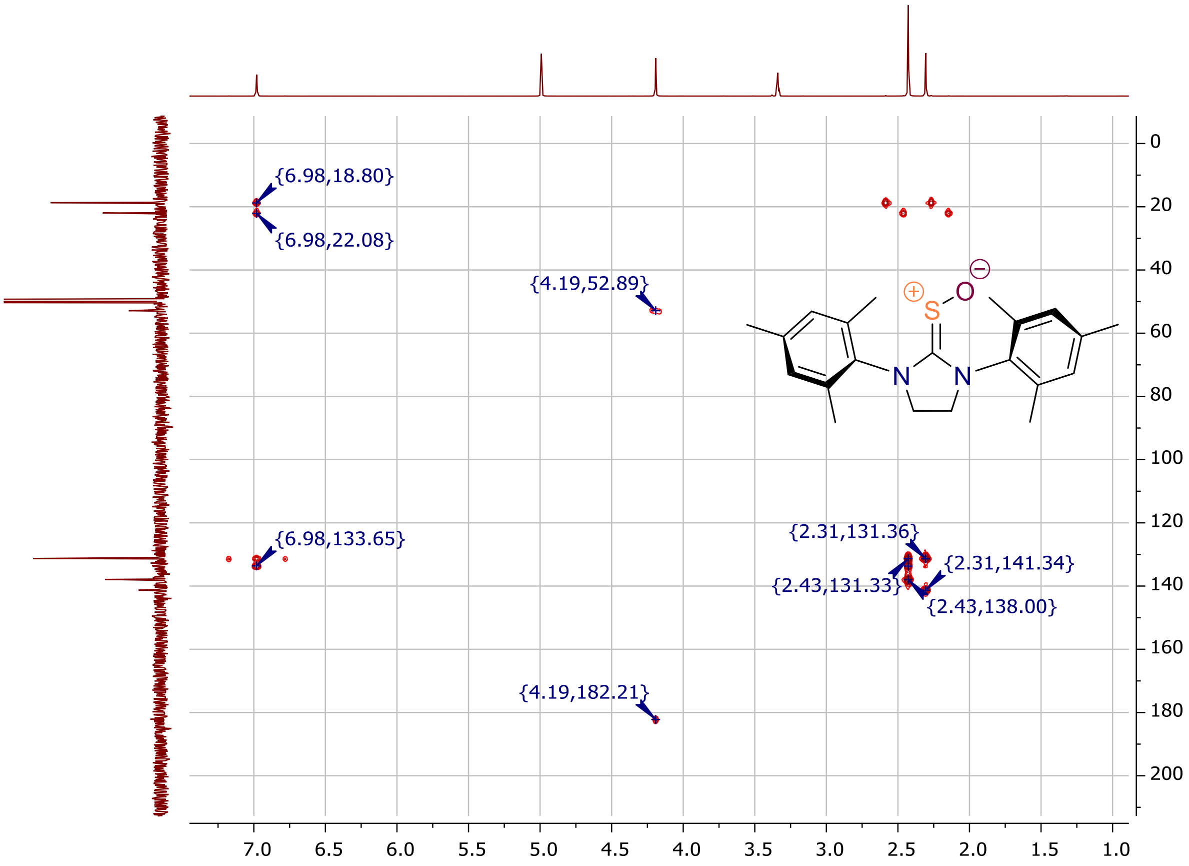

Figure S67. HMBC of **SIMes-SO**.

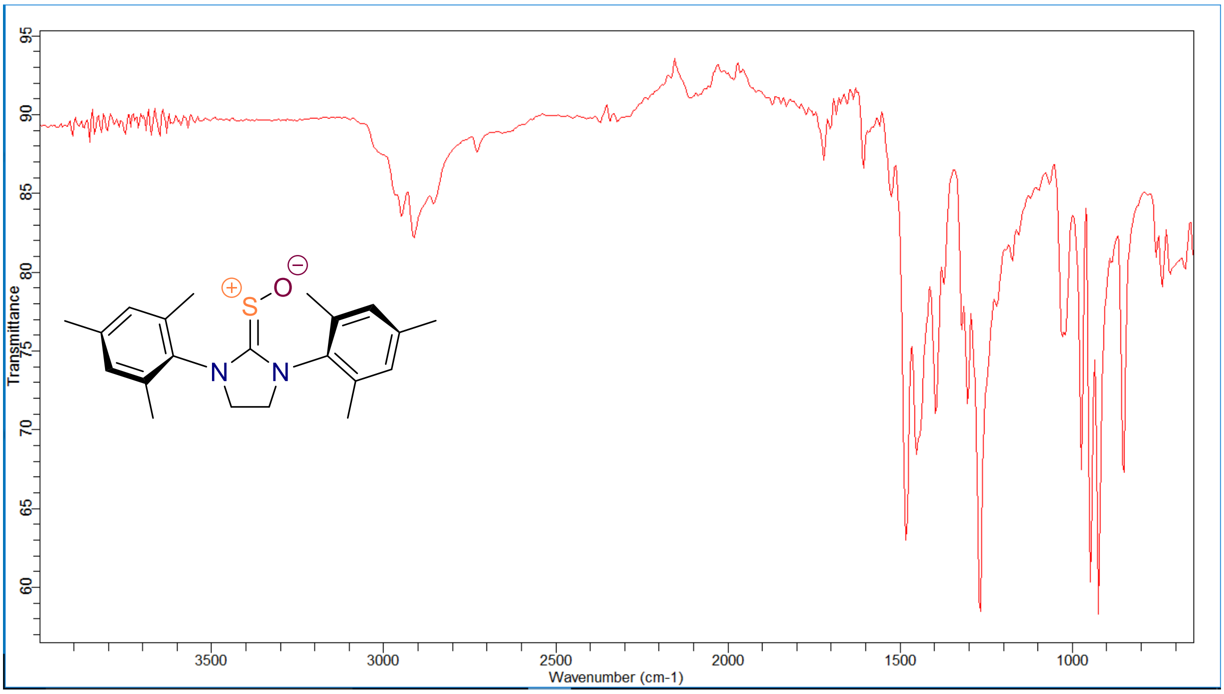

Figure S68. FTIR of **SIMes-SO**.

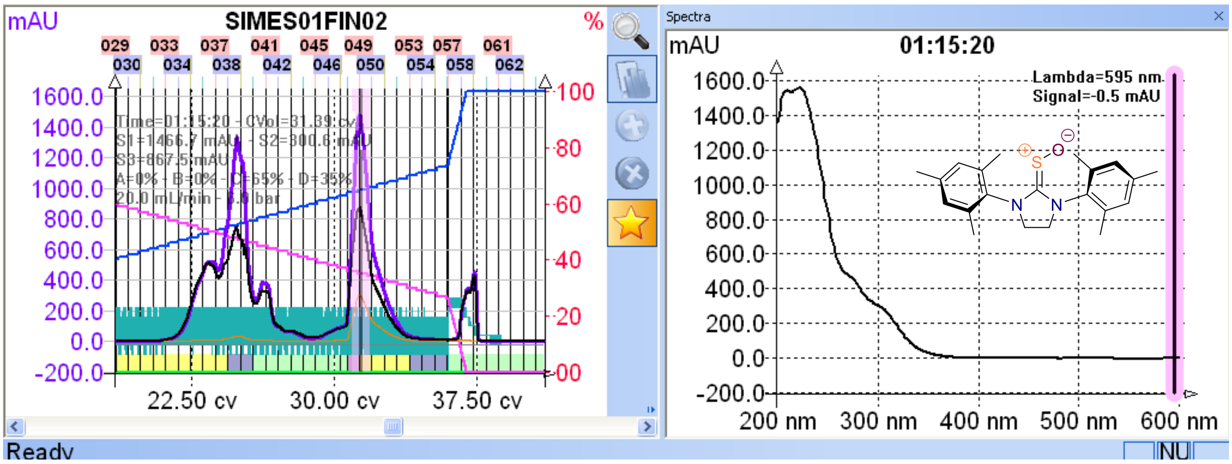

Figure S69. UV/visible data obtained during MPLC separation of **SIMes-SO**.

Analysis Info

Analysis Name	D:\Data\oi\Wagner_Medvedko_medv0847_GB2_01_58975.d	Acquisition Date	8/30/2022 10:28:51 AM
Method	fia_ms_80-1000_pos_neu.m	Operator	BDAL@DE
Sample Name	Wagner_Medvedko_medv0847	Instrument	maXis 288882.21253
Comment			

Acquisition Parameter

Source Type	ESI	Ion Polarity	Positive	Set Nebulizer	1.2 Bar
Focus	Not active	Set Capillary	4500 V	Set Dry Heater	200 °C
Scan Begin	80 m/z	Set End Plate Offset	-500 V	Set Dry Gas	6.0 l/min
Scan End	1000 m/z	Set Charging Voltage	0 V	Set Divert Valve	Waste
		Set Corona	0 nA	Set APCI Heater	0 °C

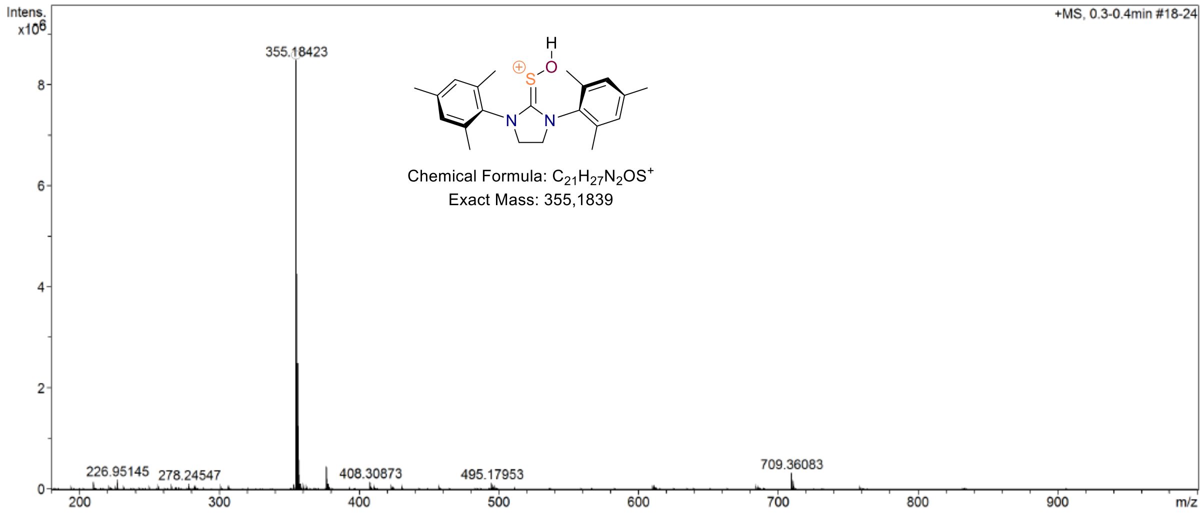

Meas. m/z	#	Ion Formula	m/z	err [ppm]	mSigma	# mSigma	Score	rdb	e⁻ Conf	N-Rule
355.18423	1	C21H27N2OS	355.18386	-1.04	24.9	1	100.00	9.5	even	ok

Figure S70. ESI-MS of **SIMes-SO**.

21. SIDipp-SO

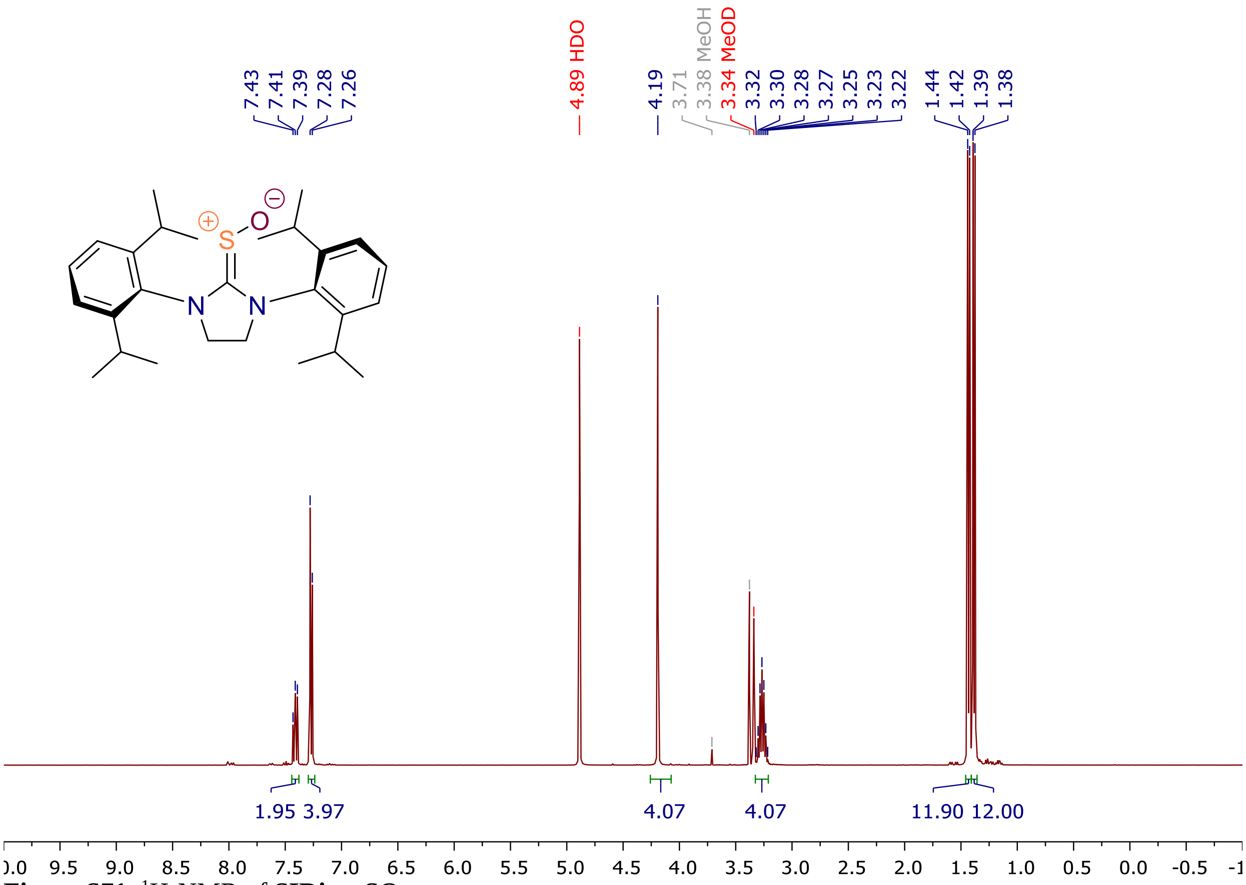

Figure S71. ^{1}H-NMR of **SIDipp-SO**.

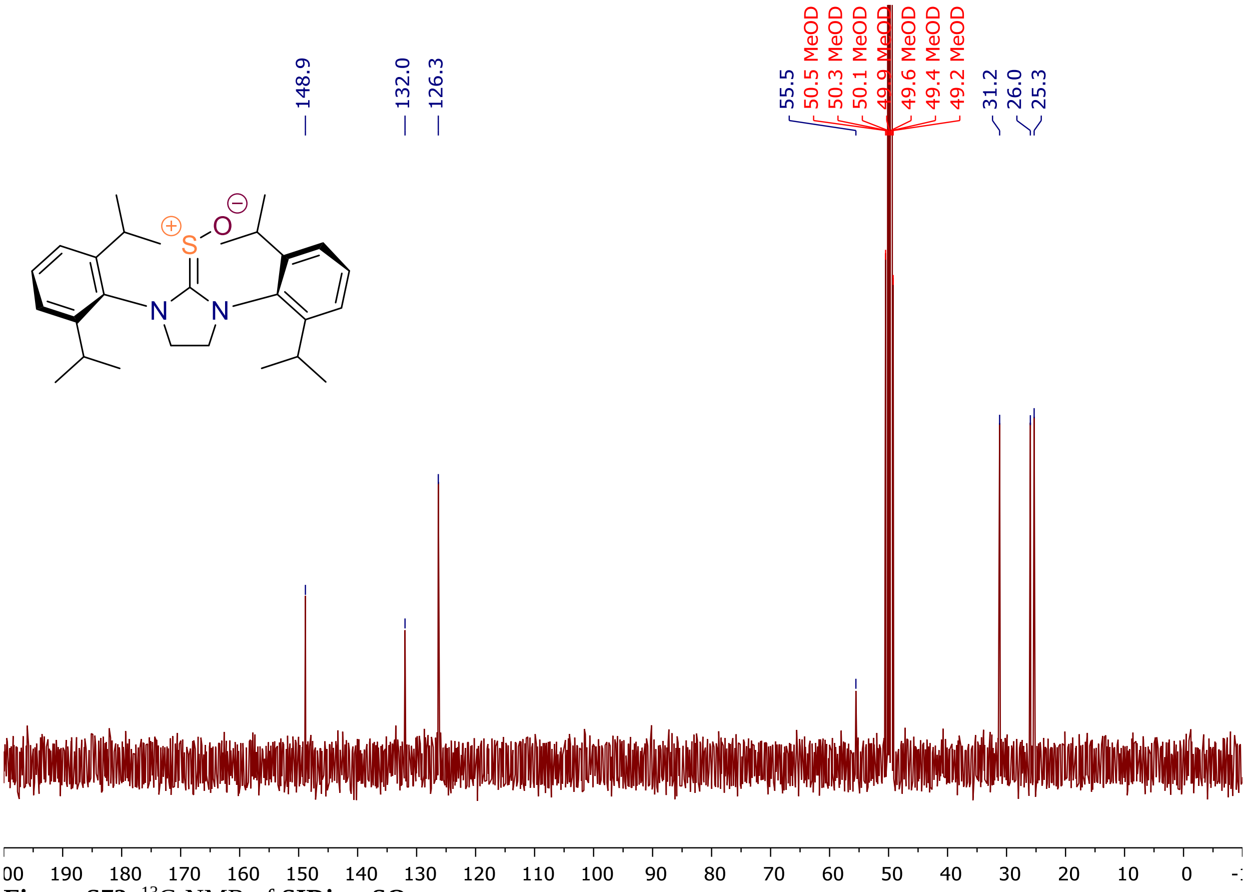

Figure S72. ^{13}C-NMR of **SIDipp-SO**.

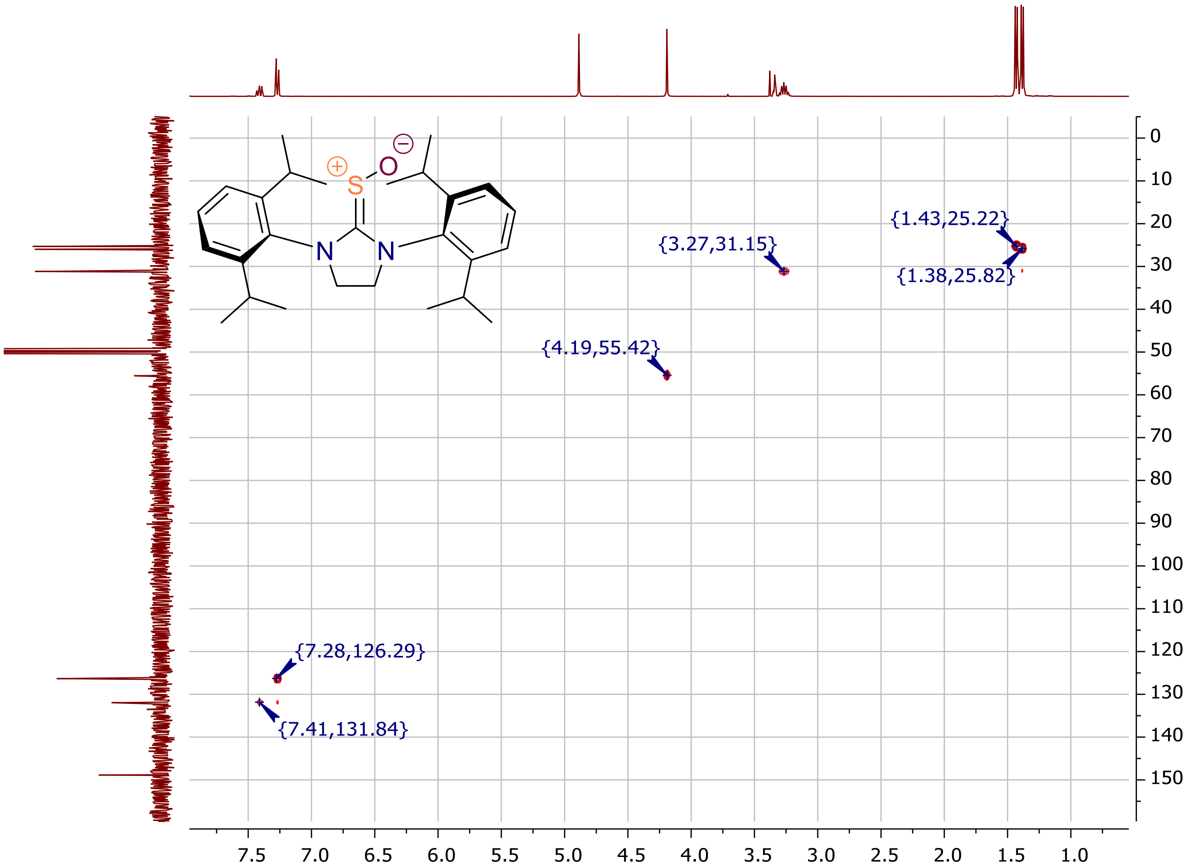

Figure S73. HSQC of **SIDipp-SO**.

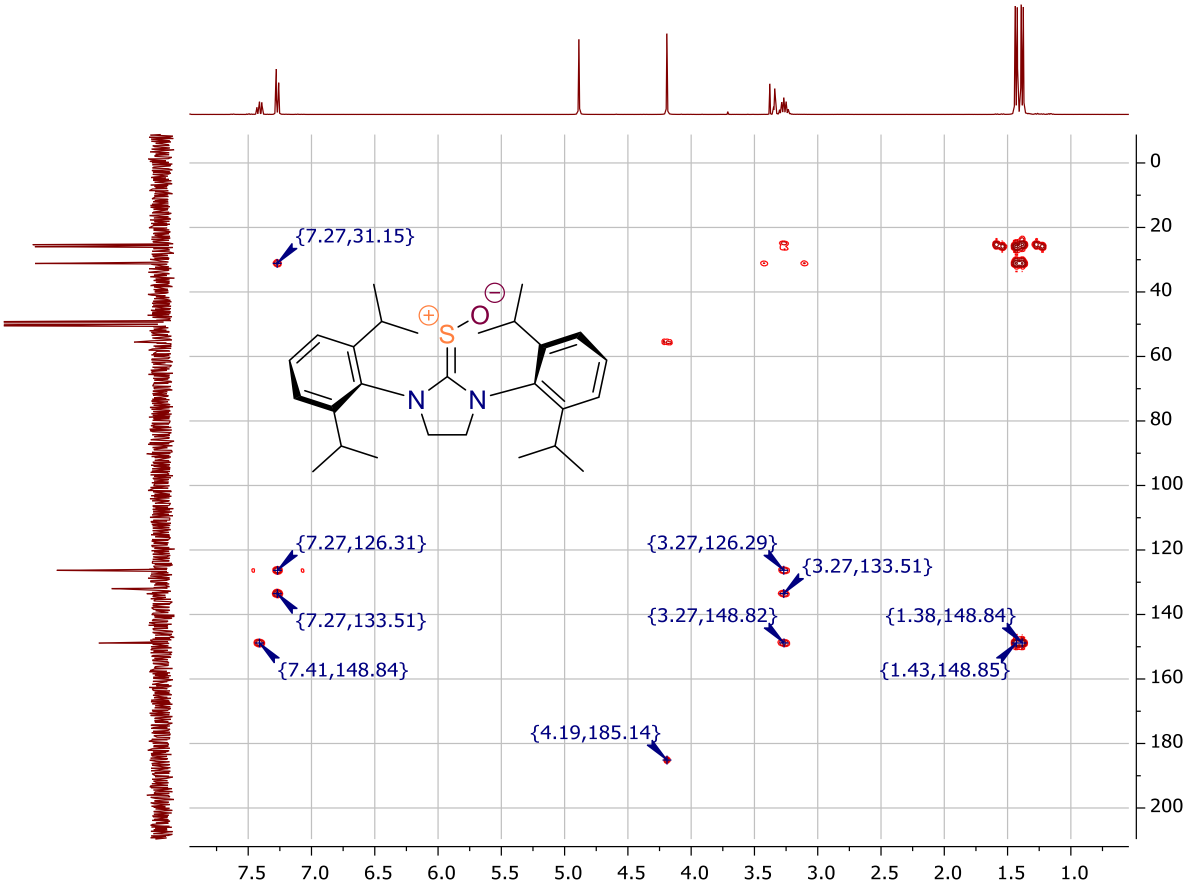

Figure S74. HMBC of **SIDipp-SO**.

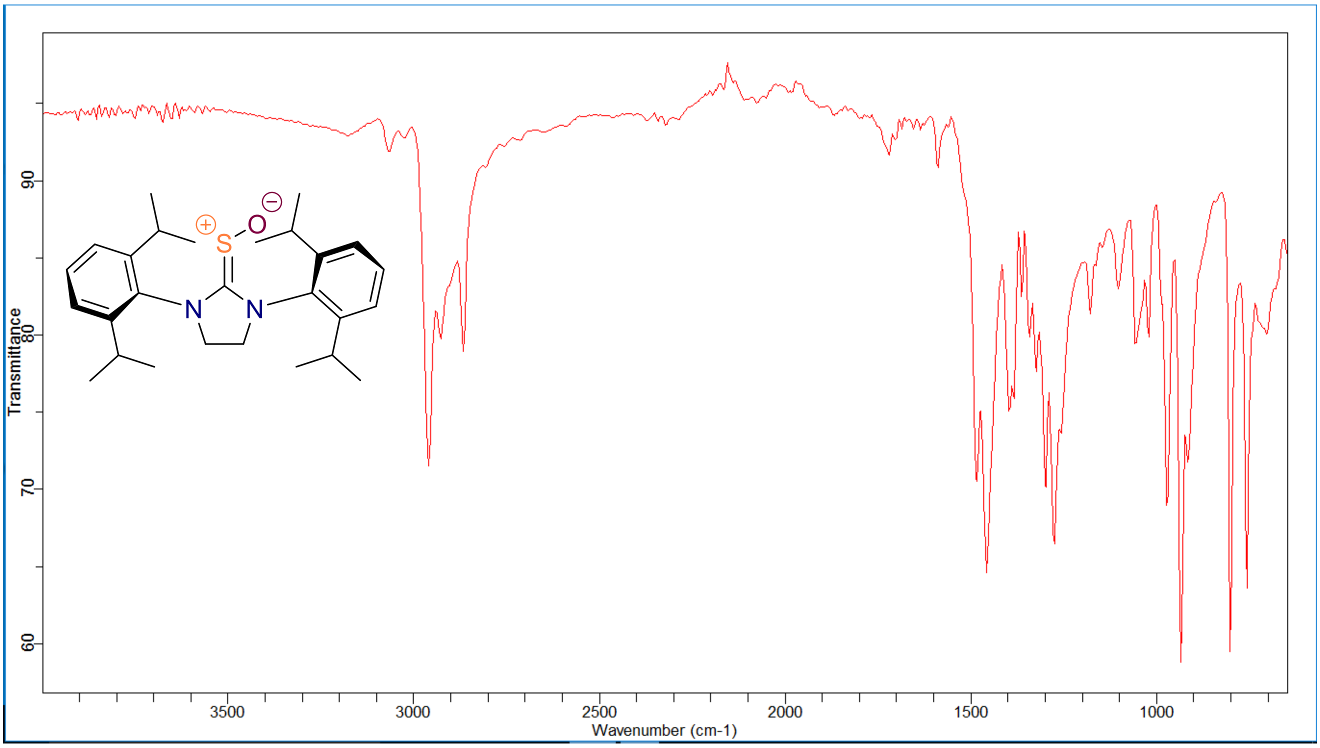

Figure S75. FTIR of **SIDipp-SO**.

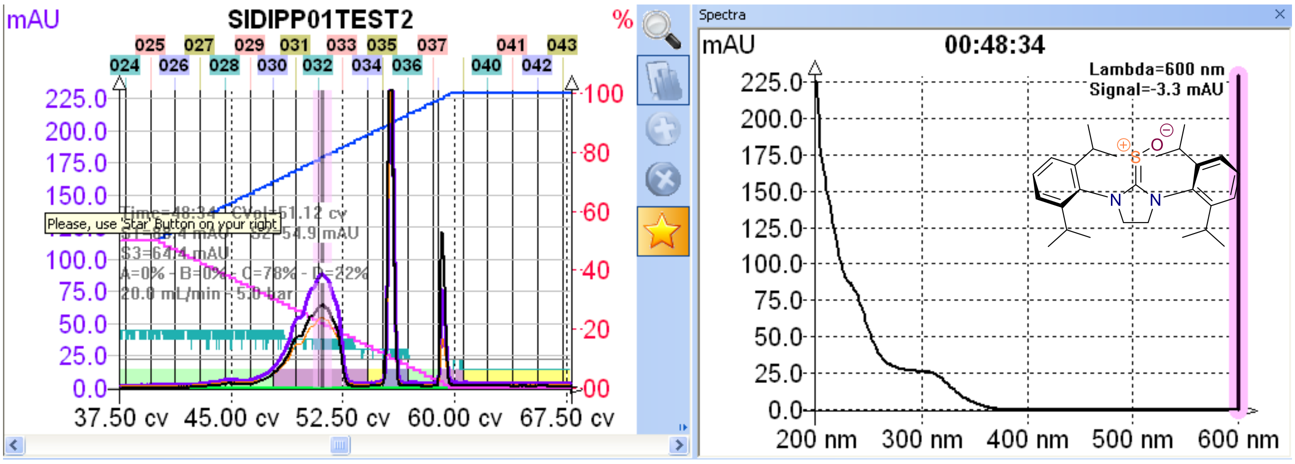

Figure S75. UV/visible data obtained during MPLC separation of **SIDipp-SO**.

Mass Spectrum SmartFormula Report

Analysis Info

Analysis Name F:\Daten\Maxis_2022\Wagner\Medvedko\Wagner_Medvedko_SID05-29-31_GE1_01_58509.d
Method fia_ms_80-1000_pos_neu.m
Sample Name Wagner_Medvedko_SID05-29-31
Comment

Acquisition Date 8/2/2022 12:40:43 PM
Operator BDAL@DE
Instrument maXis 288882.21253

Acquisition Parameter

Source Type	ESI	Ion Polarity	Positive	Set Nebulizer	1.2 Bar
Focus	Not active	Set Capillary	4500 V	Set Dry Heater	200 °C
Scan Begin	80 m/z	Set End Plate Offset	-500 V	Set Dry Gas	6.0 l/min
Scan End	1000 m/z	Set Charging Voltage	0 V	Set Divert Valve	Waste
		Set Corona	0 nA	Set APCI Heater	0 °C

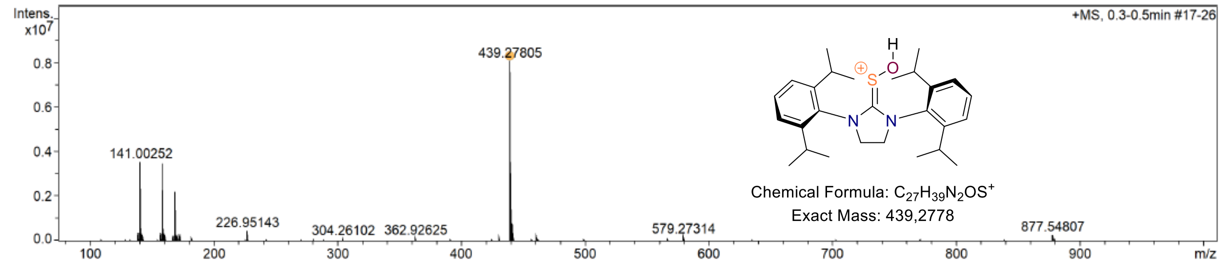

	Meas. m/z	#	Ion Formula	m/z	err [ppm]	mSigma	# mSigma	Score	rdb	e⁻ Conf	N-Rule
[M+H]+	439.27805	1	C27H39N2OS	439.27776	-0.66	45.3	1	100.00	9.5	even	ok

Figure S76. ESI-MS of **SIDipp-SO**.

22. Temperature-dependent ^{1}H-NMR experiment of SIDipp-SO in MeOD

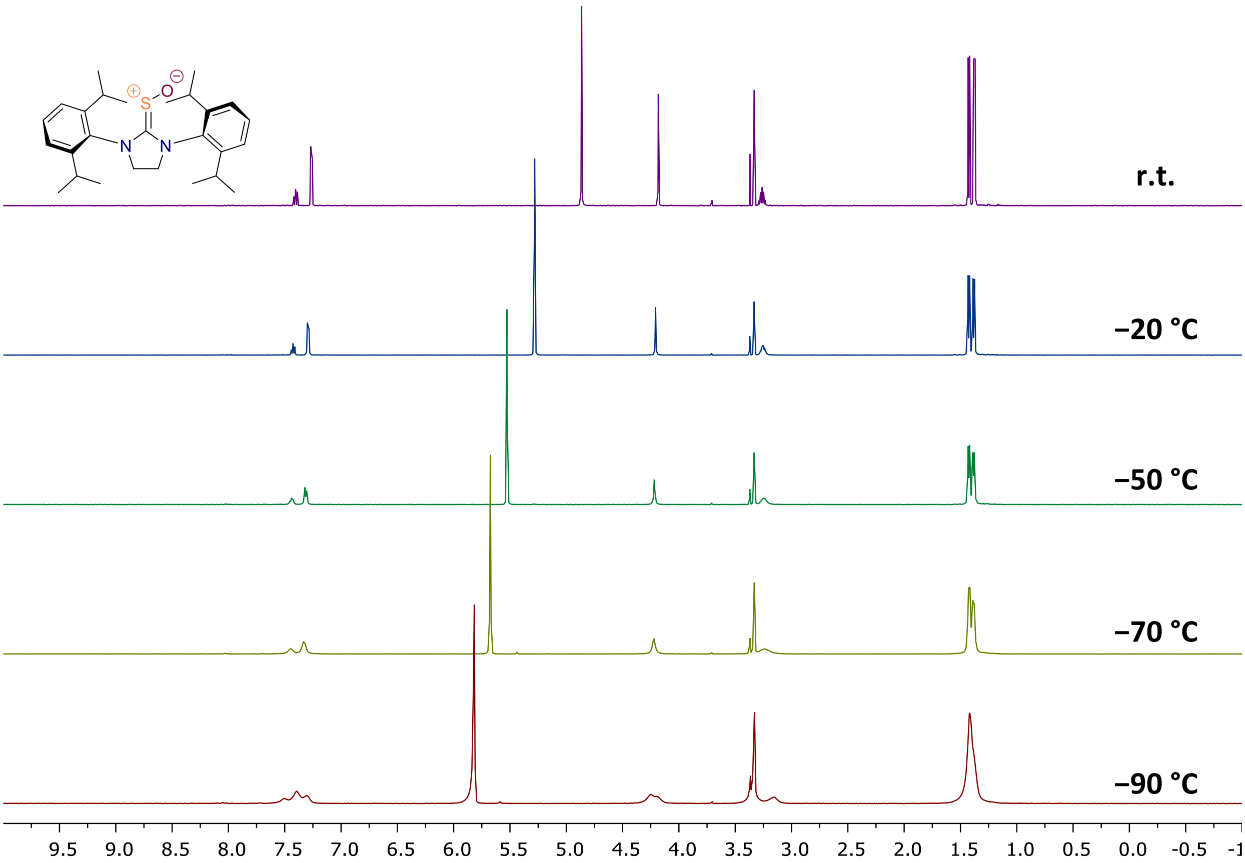

Figure S77. VT-NMR of **SIDipp-SO**.

23. Decay of SIDipp-SO in $CDCl_3$

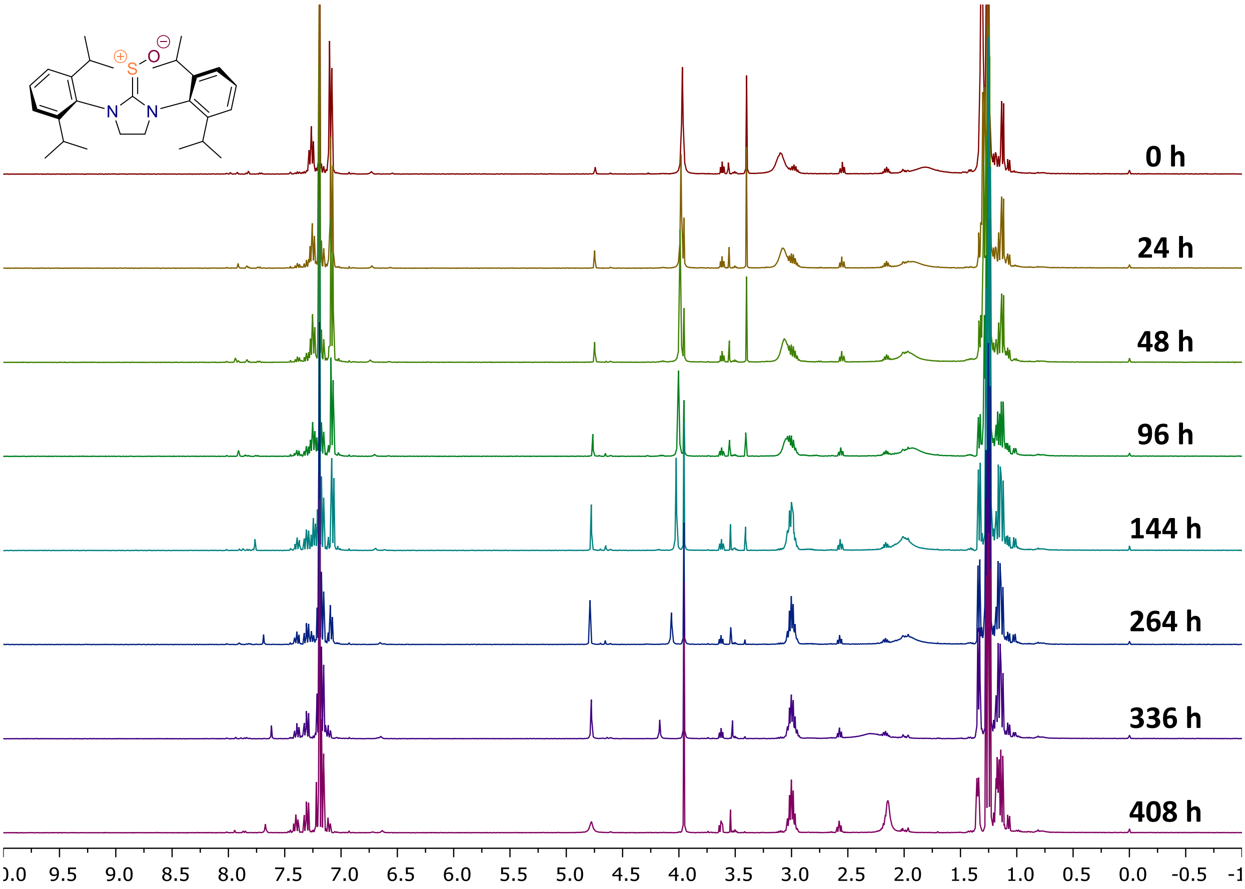

Figure S78. Decay of **SIDipp-SO** in $CDCl_3$.

24. Oxidation of SIDipp-SO with equimolar H_2O_2 in MeOD

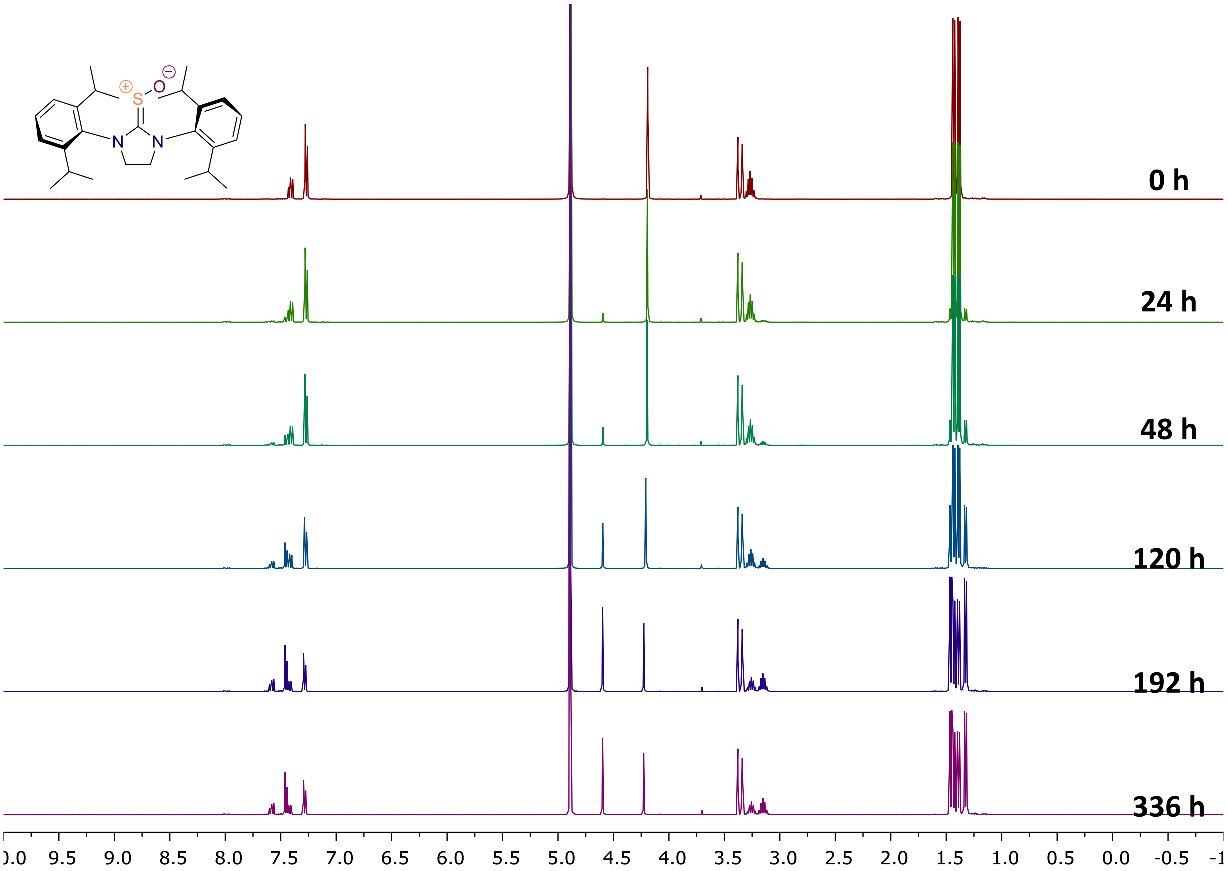

Figure S79. Oxidation of **SIDipp-SO** with H_2O_2.

25. Reaction of SIDipp-SO with aqueous HCl in MeOD

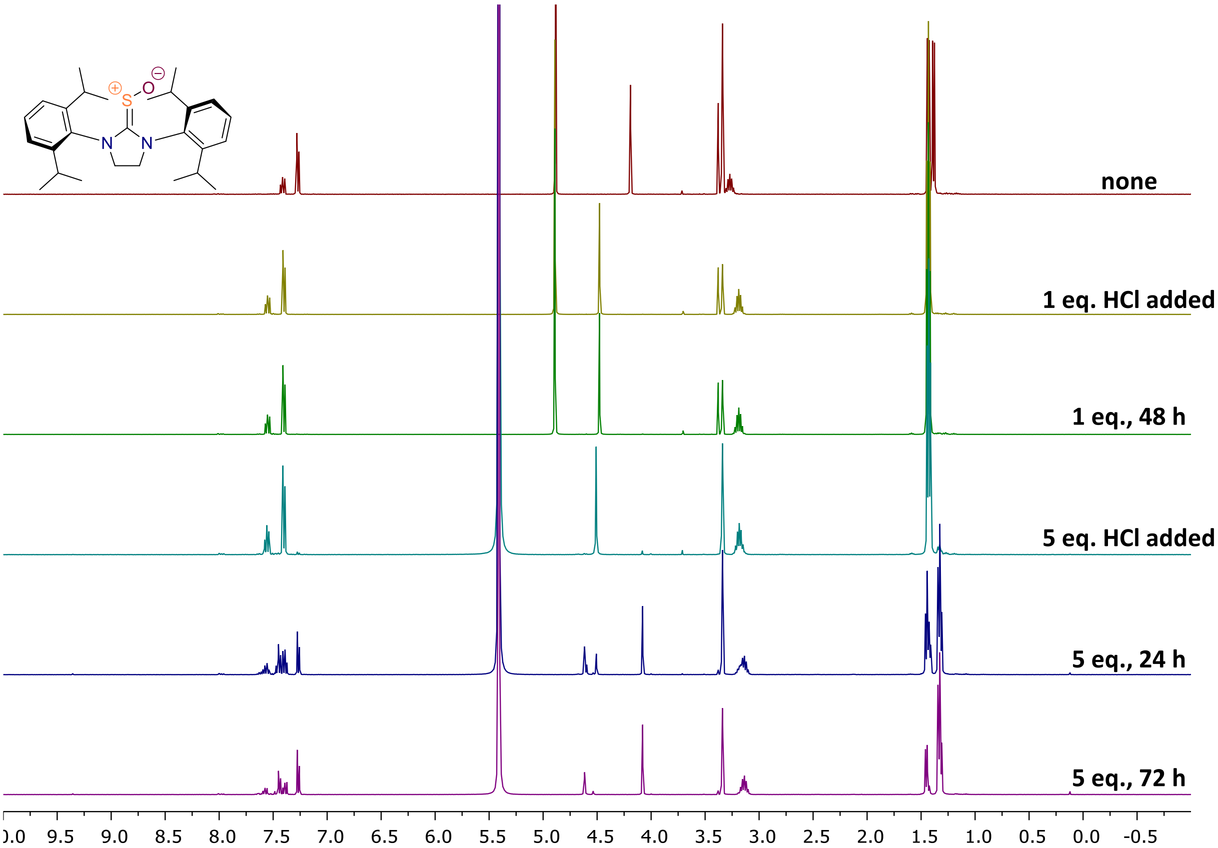

Figure S80. Reaction of **SIDipp-SO** with HCl.

25. SIDipp-SO before and after addition of 1 eq. aqueous HCl in MeOD

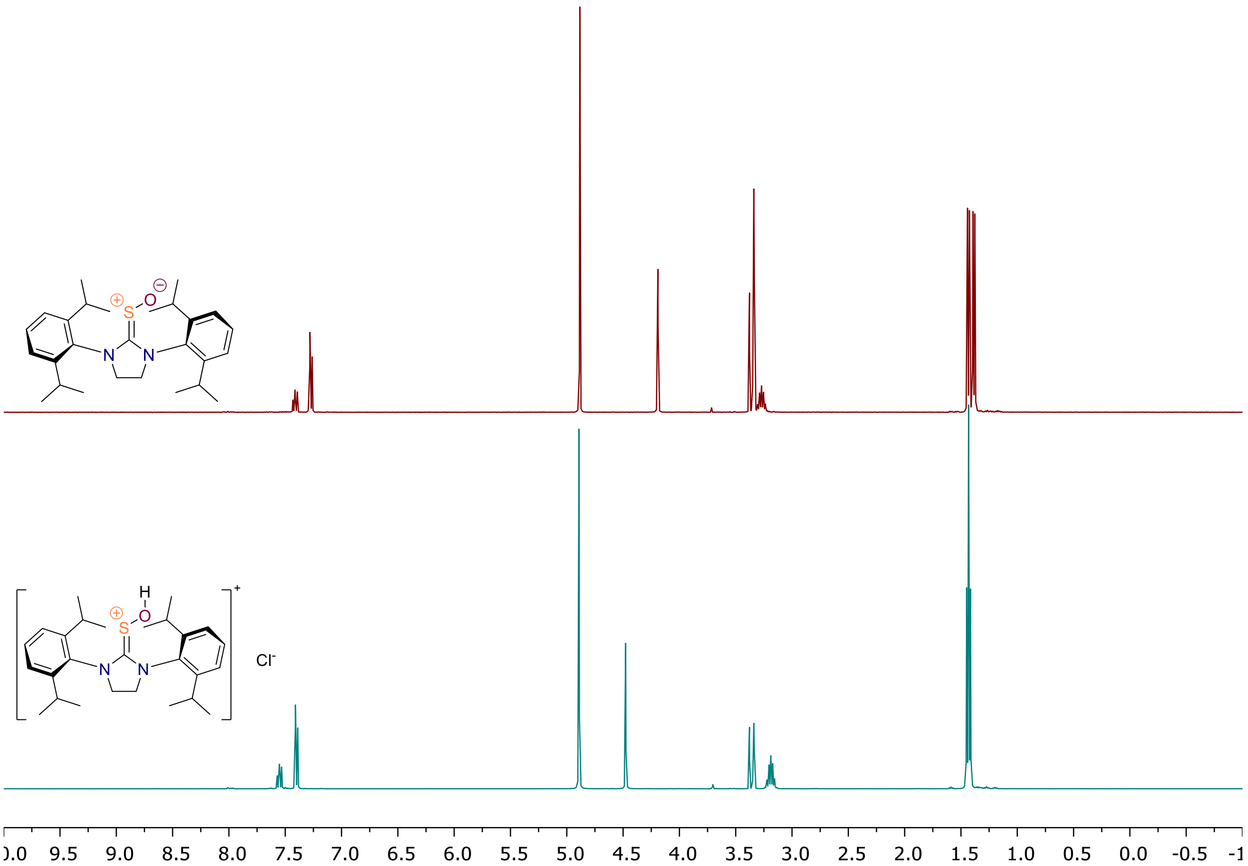

Figure S81. Protonation of **SIDipp-SO** with HCl.

IV. X-ray crystallography data and experimental description

SM72049V2

R_1=1.92%

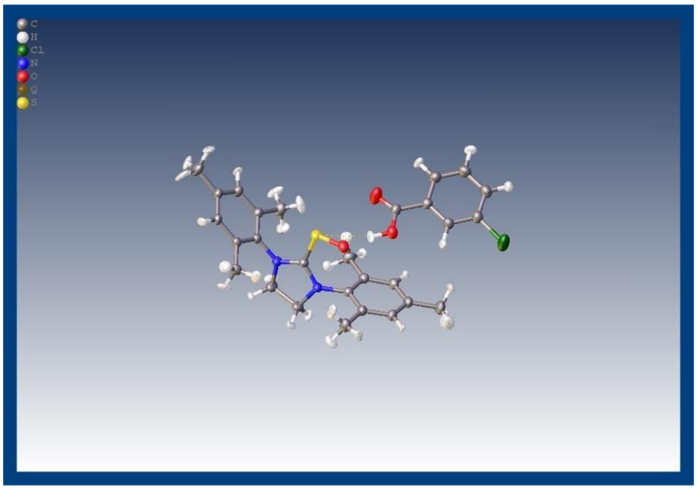

**Compound**	**SM72049V2**
Formula	$C_{28}H_{31}ClN_2O_3S$
CCDC	2201282
$D_{calc.}$/ g cm^{-3}	1.310
m/mm^{-1}	2.317
Formula Weight	511.088
Colour	clear colourless
Shape	block-shaped
Size/mm^3	0.11×0.10×0.08
T/K	149.99(10)
Crystal System	monoclinic
Flack Parameter	-0.0136(16)
Hooft Parameter	-0.0136(16)
Space Group	$P2_1$
a/Å	7.4860(2)
b/Å	15.7942(3)
c/Å	11.4580(3)
a/°	90
b/°	107.022(2)
g/°	90
V/Å^3	1295.39(6)
Z	2
Z'	1
Wavelength/Å	1.54184
Radiation type	Cu K_a
Q_{min}/°	4.03
Q_{max}/°	80.17
Measured Refl's.	43978
Indep't Refl's	5613
Refl's I≥2 *s*(I)	5571
R_{int}	0.0214
Parameters	595
Restraints	1
Largest Peak	0.2916
Deepest Hole	-0.3542
GooF	1.0945
wR_2 (all data)	0.0509
wR_2	0.0509
R_1 (all data)	0.0193
R_1	0.0192

Experimental. Single clear colourless block-shaped crystals of **SM72049V2** were grown by a slow evaporation of ethanol from solution containing roughly equimolar amounts of **SIMes-SO** and residual *m*CBA, and used in X-ray study as supplied. A suitable crystal with dimensions 0.11 × 0.10 × 0.08 mm^3 was selected and mounted on a XtaLAB Synergy, Dualflex, HyPix diffractometer. The crystal was kept at a steady T = 149.99(10) K during data collection. The structure was solved with the **ShelXT**[20] solution program using dual methods and by using **Olex2** 1.5[21] as the graphical interface. The model was refined with **olex2.refine** 1.5[22] using full matrix least squares minimisation on $\boldsymbol{F}^2$.

Crystal Data. $C_{28}H_{31}ClN_2O_3S$, M_r = 511.088, monoclinic, $P2_1$ (No. 4), a = 7.4860(2) Å, b = 15.7942(3) Å, c = 11.4580(3) Å, b = 107.022(2)°, $a = g$ = 90°, V = 1295.39(6) Å^3, T = 149.99(10) K, Z = 2, Z' = 1, m(Cu K_a) = 2.317, 43978 reflections measured, 5613 unique (R_{int} = 0.0214) which were used in all calculations. The final wR_2 was 0.0509 (all data) and R_1 was 0.0192 (I≥2 s(I)).

Structure Quality Indicators

**Reflections:**	d min (Cu\a) 2Θ=160.3° 0.78	I/σ(I) 111.1	Rint 2.14%	Full 135.4° 100
**Refinement:**	Shift 0.001	Max Peak 0.3	Min Peak -0.4	GooF 1.095	Hooft -.0136(16)

A clear colourless block-shaped-shaped crystal with dimensions 0.11 × 0.10 × 0.08 mm^3 was mounted. Data were collected using a XtaLAB Synergy, Dualflex, HyPix diffractometer operating at T = 149.99(10) K.

Data were measured using w scans with Cu K_a radiation. The diffraction pattern was indexed and the total number of runs and images was based on the strategy calculation from the program CrysAlisPro 1.171.42.61a (Rigaku OD, 2022). The maximum resolution that was achieved was Q = 80.17° (0.78 Å).

The unit cell was refined using CrysAlisPro 1.171.42.61a (Rigaku OD, 2022) on 9266 reflections, 21% of the observed reflections. Data reduction, scaling and absorption corrections were performed using CrysAlisPro 1.171.42.61a (Rigaku OD, 2022). The final completeness is 100.00 % out to 80.17° in Q. An analytical absorption correction was performed using CrysAlisPro 1.171.42.61a (Rigaku Oxford Diffraction, 2022) Analytical numeric absorption correction using a multifaceted crystal model based on expressions derived by R.C. Clark & J.S. Reid.[23] Empirical absorption correction using spherical harmonics, implemented in SCALE3 ABSPACK scaling algorithm. The absorption coefficient m of this material is 2.317 mm^{-1} at this wavelength (l = 1.54184Å) and the minimum and maximum transmissions are 0.831 and 0.871.

The structure was solved and the space group $P2_1$ (# 4) determined by the ShelXT[20] structure solution program using using dual methods and refined by full matrix least squares minimisation on $\boldsymbol{F^2}$ using version of **olex2.refine** 1.5.[21] All non-hydrogen atoms were refined anisotropically. Hydrogen atom positions were calculated geometrically and refined using the riding model.

_refine_special_details: Refinement using NoSpherA2, an implementation of non-spherical atom-form-factors in Olex2.[24] NoSpherA2 implementation of HAR makes use of tailor-made aspherical atomic form factors calculated on-the-fly from a Hirshfeld-partitioned electron density (ED) - not from spherical-atom form factors. The ED is calculated from a gaussian basis set single determinant SCF wavefunction - either Hartree-Fock or DFT using selected funtionals - for a fragment of the crystal. This fragment can be embedded in an electrostatic crystal field by employing cluster charges or modelled using implicit solvation models, depending on the software used. The following options were used: SOFTWARE: ORCA 5.0 PARTITIONING: NoSpherA2 INT ACCURACY: Normal METHOD: M062X BASIS SET: 6-311G(d,p) CHARGE: 0 MULTIPLICITY: 1 DATE: 2022-08-13_14-24-59

_exptl_absorpt_process_details: CrysAlisPro 1.171.42.61a (Rigaku Oxford Diffraction, 2022) Analytical numeric absorption correction using a multifaceted crystal model based on expressions derived by R.C. Clark & J.S. Reid[23] using spherical harmonics, implemented in SCALE3 ABSPACK scaling algorithm.

There is a single molecule in the asymmetric unit, which is represented by the reported sum formula. In other words: Z is 2 and Z' is 1.

The Flack parameter was refined to -0.0136(16). Determination of absolute structure using Bayesian statistics on Bijvoet differences using the Olex2 results in -0.0136(16). Note: The Flack parameter is used to determine chirality of the crystal studied, the value should be near 0, a value of 1 means that the stereochemistry is wrong and the model should be inverted. A value of 0.5 means that the crystal consists of a racemic mixture of the two enantiomers.

Table S1: Fractional Atomic Coordinates (×10^4) and Equivalent Isotropic Displacement Parameters (Å^2×10^3) for **SM72049V2**. U_{eq} is defined as 1/3 of the trace of the orthogonalised U_{ij}.

Atom	x	y	z	U_{eq}
C1	2983.9(13)	4529.2(6)	6472.2(8)	20.96(19)
C2	-8.1(15)	5046.1(8)	5696.8(12)	31.6(2)
C3	-76.1(15)	4214.2(8)	6370.7(13)	34.6(3)
C4	2694.2(14)	6070.2(6)	6049.5(9)	23.2(2)
C5	3054.1(14)	6482.0(7)	7178.3(9)	25.4(2)
C6	3601.0(16)	7327.2(7)	7245.6(11)	28.5(2)
C7	3811.3(15)	7761.7(7)	6234.1(11)	27.6(2)
C8	3442.9(15)	7331.8(7)	5121.5(10)	26.8(2)
C9	2865.1(14)	6487.5(7)	5006.6(10)	24.63(19)
C10	2911(2)	6023.1(8)	8295.0(11)	33.1(2)

Atom	x	y	z	U_{eq}
C11	4417(2)	8671.8(8)	6351.1(15)	39.9(3)
C12	2420(2)	6045.0(9)	3801.0(11)	34.2(2)
C13	2506.1(14)	3095.3(6)	7116.3(9)	23.31(19)
C14	2447.8(14)	2446.5(7)	6270.5(10)	25.4(2)
C15	3119.3(16)	1648.5(7)	6719.4(11)	28.8(2)
C16	3804.0(16)	1492.5(7)	7969.9(11)	30.1(2)
C17	3808.0(16)	2149.4(7)	8781.8(10)	30.0(2)
C18	3185.1(16)	2960.1(7)	8377.9(10)	25.8(2)
C19	1714.2(18)	2599.4(9)	4922.0(11)	33.4(2)
C20	4562(3)	636.8(9)	8440.3(15)	44.3(3)
C21	3282(2)	3663.3(8)	9282.1(12)	35.2(3)
N1	2013.0(11)	5213.8(5)	5948.9(8)	23.64(17)
N2	1867.4(11)	3919.7(5)	6666.6(8)	25.09(18)
O1	6138.6(11)	5085.5(5)	6203.9(8)	30.38(16)
S1	5343.6(3)	4368.36(18)	6831.5(2)	25.82(6)
C22	8300.1(17)	6273.8(7)	8599.4(12)	32.4(2)
C23	9341.1(16)	7056.0(7)	9169.6(11)	29.3(2)
C24	9311.2(17)	7768.8(8)	8449.6(11)	31.4(2)
C25	10291.3(18)	8484.6(8)	8977.3(12)	36.8(3)
C26	11323.1(18)	8510.5(9)	10194.8(12)	38.8(3)
C27	11355(2)	7788.4(10)	10907.8(13)	42.0(3)
C28	10352.7(19)	7064.8(9)	10401.4(12)	37.5(3)
Cl1	10187.7(7)	9377.5(3)	8068.3(4)	66.78(12)
O2	8046(2)	5685.2(7)	9204.5(11)	64.5(3)
O3	7701.0(13)	6311.4(6)	7412.2(8)	37.40(19)

Table S2: Anisotropic Displacement Parameters (×10^4) for **SM72049V2**. The anisotropic displacement factor exponent takes the form: $-2p^2[h^2a^{*2} \times U_{11} + ... + 2hka^* \times b^* \times U_{12}]$

Atom	U_{11}	U_{22}	U_{33}	U_{23}	U_{13}	U_{12}
C1	18.5(4)	20.8(5)	22.9(4)	0.2(3)	5.1(3)	0.1(3)
C2	19.1(5)	31.7(6)	41.1(7)	1.7(4)	4.1(5)	7.6(5)
H2a	24(8)	78(13)	59(12)	-20(8)	-4(8)	21(10)
H2b	51(10)	32(9)	88(14)	4(8)	34(10)	-2(9)
C3	18.3(4)	33.2(7)	51.0(7)	0.1(4)	8.3(5)	11.6(5)
H3a	44(10)	119(19)	65(12)	18(12)	30(9)	37(14)
H3b	27(8)	43(9)	65(11)	-4(7)	-9(8)	-1(8)
C4	21.9(4)	22.0(5)	24.5(5)	0.6(4)	4.8(4)	0.1(4)
C5	26.5(5)	24.2(5)	24.7(5)	2.2(4)	6.4(4)	-1.4(4)
C6	32.0(5)	24.5(5)	27.2(5)	1.0(4)	6.1(4)	-5.6(4)
H6	54(10)	36(9)	53(10)	-12(8)	3(8)	7(8)
C7	28.9(5)	20.0(5)	32.2(5)	0.4(4)	6.1(4)	-2.6(4)
C8	28.9(5)	22.0(5)	29.0(5)	-1.2(4)	7.9(4)	0.4(4)
H8	64(11)	17(7)	38(9)	-6(7)	-3(8)	10(7)
C9	26.3(5)	21.0(5)	26.0(5)	-0.6(4)	6.8(4)	-0.7(4)
C10	39.2(7)	35.0(6)	26.8(6)	3.1(6)	12.2(5)	1.7(5)
H10a	66(13)	72(14)	50(11)	13(11)	25(10)	-9(10)
H10b	73(12)	42(9)	27(8)	-3(9)	14(8)	-16(8)
H10c	55(12)	93(16)	49(11)	-15(11)	17(9)	-2(11)
C11	48.5(8)	22.0(6)	45.9(8)	-3.4(5)	8.6(6)	-4.2(5)
H11a	130(20)	29(11)	220(40)	-8(13)	70(20)	-2(16)
H11b	102(18)	61(14)	120(20)	-35(13)	-65(17)	17(14)
H11c	150(20)	110(20)	44(12)	-47(17)	15(14)	15(12)
C12	44.7(7)	30.3(6)	28.8(6)	-5.8(5)	13.0(5)	-4.2(5)
H12a	36(11)	113(19)	84(14)	-24(11)	27(10)	-22(14)
H12b	76(12)	57(10)	24(8)	-35(9)	20(8)	5(8)
H12c	190(30)	36(11)	38(10)	-23(14)	19(13)	-19(9)
C13	21.1(4)	21.6(5)	26.5(5)	-1.7(3)	5.8(4)	1.7(4)
C14	24.7(5)	23.8(5)	25.5(5)	-3.4(4)	3.7(4)	-1.4(4)
C15	32.0(5)	22.2(5)	29.6(5)	-2.6(4)	4.7(4)	-2.5(4)

Atom	U_{11}	U_{22}	U_{33}	U_{23}	U_{13}	U_{12}
H15	70(12)	44(10)	31(9)	3(9)	-14(8)	-20(8)
C16	35.6(5)	21.8(5)	30.0(6)	-0.4(4)	5.2(4)	0.6(4)
C17	37.5(6)	24.1(5)	27.3(6)	0.3(4)	7.6(4)	1.8(4)
H17	89(14)	41(9)	14(8)	-4(9)	5(8)	13(7)
C18	29.0(5)	22.7(5)	26.2(5)	0.5(4)	8.7(4)	-0.1(4)
C19	34.4(6)	36.4(7)	25.9(6)	-1.7(5)	3.2(5)	-1.2(5)
H19a	41(11)	150(20)	38(10)	5(13)	-11(8)	19(12)
H19b	140(20)	68(15)	33(10)	-22(14)	2(11)	-15(10)
H19c	80(14)	81(15)	68(13)	12(12)	27(11)	-14(12)
C20	64.0(10)	23.7(6)	39.9(7)	3.4(6)	7.0(7)	0.8(6)
H20a	71(16)	65(16)	140(20)	21(12)	-31(16)	46(16)
H20b	80(15)	23(9)	140(20)	9(9)	57(15)	17(11)
H20c	200(30)	32(11)	61(13)	37(14)	-2(16)	18(11)
C21	45.3(7)	30.4(6)	31.5(6)	2.6(6)	13.8(5)	-3.6(5)
H21a	107(17)	100(18)	84(15)	-65(15)	61(14)	-49(13)
H21b	97(16)	59(12)	58(12)	37(12)	16(11)	-16(9)
H21c	180(30)	67(14)	35(10)	57(15)	39(13)	13(10)
N1	19.7(4)	21.7(4)	27.9(4)	0.3(3)	4.4(3)	1.8(3)
N2	19.1(4)	23.3(4)	31.7(5)	-0.6(3)	5.6(3)	3.5(3)
O1	24.6(3)	30.0(4)	37.8(4)	-2.8(3)	11.1(3)	-3.1(3)
S1	18.24(10)	24.57(11)	34.54(12)	2.09(9)	7.57(8)	1.26(10)
C22	36.0(6)	25.7(6)	36.0(6)	-1.7(4)	11.2(5)	0.7(4)
C23	29.9(5)	27.7(5)	29.8(5)	-0.0(4)	8.1(4)	-1.4(4)
C24	35.3(5)	30.1(6)	27.0(5)	-3.7(5)	6.3(5)	-1.4(4)
H24	61(12)	27(9)	70(13)	-1(8)	-4(10)	-7(8)
C25	40.7(6)	32.3(6)	34.6(6)	-6.2(5)	6.7(5)	-1.6(5)
C26	37.7(6)	37.7(7)	36.3(6)	-6.1(5)	3.7(5)	-7.8(5)
H26	75(13)	46(11)	56(11)	-9(10)	8(10)	-5(9)
C27	43.9(6)	44.2(7)	30.4(6)	-0.4(6)	-0.8(5)	-4.2(5)
H27	93(15)	70(14)	25(9)	13(11)	6(10)	-4(9)
C28	44.1(7)	34.9(6)	29.5(6)	2.4(5)	4.7(5)	2.0(5)
H28	125(18)	30(9)	30(9)	-1(10)	3(10)	3(8)
Cl1	97.7(3)	40.06(17)	53.36(19)	-25.5(2)	7.65(18)	7.81(17)
O2	99.8(10)	39.7(6)	46.4(6)	-21.7(6)	9.7(6)	7.2(5)
O3	41.5(5)	33.3(5)	35.0(4)	-10.0(4)	7.3(4)	-2.6(4)
H3	47(14)	21(12)	88(17)	2(11)	18(12)	-34(12)

Table S3: Bond Lengths in Å for **SM72049V2**.

Atom	Atom	Length/Å
C1	N1	1.3423(12)
C1	N2	1.3356(12)
C1	S1	1.7113(9)
C2	C3	1.5324(16)
C2	N1	1.4788(13)
C3	N2	1.4694(13)
C4	C5	1.4016(14)
C4	C9	1.4028(14)
C4	N1	1.4384(13)
C5	C6	1.3918(15)
C5	C10	1.5014(15)
C6	C7	1.3950(16)
C7	C8	1.3987(16)
C7	C11	1.5015(15)
C8	C9	1.3961(15)
C9	C12	1.4961(16)
C13	C14	1.4023(15)
C13	C18	1.4011(15)
C13	N2	1.4301(13)

Atom	Atom	Length/Å
C14	C15	1.3980(15)
C14	C19	1.5003(16)
C15	C16	1.3951(16)
C16	C17	1.3930(16)
C16	C20	1.5034(17)
C17	C18	1.3946(16)
C18	C21	1.5060(16)
O1	S1	1.5507(8)
C22	C23	1.5040(16)
C22	O2	1.2076(16)
C22	O3	1.3030(16)
C23	C24	1.3922(17)
C23	C28	1.3928(17)
C24	C25	1.3864(17)
C25	C26	1.3833(18)
C25	Cl1	1.7414(13)
C26	C27	1.399(2)
C27	C28	1.397(2)

Table S4: Bond Angles in ° for **SM72049V2**.

Atom	Atom	Atom	Angle/°
N2	C1	N1	111.82(8)
S1	C1	N1	127.80(7)
S1	C1	N2	120.34(7)
N1	C2	C3	103.78(9)
N2	C3	C2	102.18(9)
C9	C4	C5	121.82(9)
N1	C4	C5	118.57(9)
N1	C4	C9	119.48(9)
C6	C5	C4	118.04(10)
C10	C5	C4	121.34(10)
C10	C5	C6	120.60(10)
C7	C6	C5	121.92(10)
C8	C7	C6	118.57(10)
C11	C7	C6	120.09(11)
C11	C7	C8	121.33(11)
C9	C8	C7	121.51(11)
C8	C9	C4	118.12(10)
C12	C9	C4	121.05(10)
C12	C9	C8	120.82(10)
C18	C13	C14	122.01(10)
N2	C13	C14	118.48(9)
N2	C13	C18	119.51(9)
C15	C14	C13	118.00(10)
C19	C14	C13	121.47(10)
C19	C14	C15	120.52(10)
C16	C15	C14	121.44(10)
C17	C16	C15	118.86(10)
C20	C16	C15	120.91(11)
C20	C16	C17	120.22(11)
C18	C17	C16	121.81(10)
C17	C18	C13	117.87(10)
C21	C18	C13	121.82(10)
C21	C18	C17	120.31(10)
C2	N1	C1	109.40(8)
C4	N1	C1	126.07(8)
C4	N1	C2	120.17(8)
C3	N2	C1	111.04(8)
C13	N2	C1	123.83(8)
C13	N2	C3	125.12(9)
O1	S1	C1	106.81(5)
O2	C22	C23	122.11(12)
O3	C22	C23	113.10(10)
O3	C22	O2	124.78(12)
C24	C23	C22	119.37(10)
C28	C23	C22	120.49(11)
C28	C23	C24	120.13(12)
C25	C24	C23	119.10(11)
C26	C25	C24	122.14(12)
Cl1	C25	C24	118.31(10)
Cl1	C25	C26	119.54(10)
C27	C26	C25	118.28(12)
C28	C27	C26	120.61(12)
C27	C28	C23	119.72(13)

Table S5: Torsion Angles in ° for **SM72049V2**.

Atom	Atom	Atom	Atom	Angle/°
C1	N1	C2	C3	10.27(10)
C1	N1	C4	C5	-69.14(11)
C1	N1	C4	C9	114.94(11)
C1	N2	C3	C2	11.96(10)
C1	N2	C13	C14	-93.17(11)
C1	N2	C13	C18	86.19(11)
C2	C3	N2	C13	-168.66(9)
C2	N1	C4	C5	84.88(10)
C2	N1	C4	C9	-91.04(11)
C3	N2	C13	C14	87.53(12)
C3	N2	C13	C18	-93.10(12)
C4	C5	C6	C7	-0.62(12)
C4	C9	C8	C7	-1.20(12)
C5	C6	C7	C8	0.56(13)
C5	C6	C7	C11	-179.57(12)
C6	C7	C8	C9	0.38(12)
C7	C8	C9	C12	178.09(11)
C13	C14	C15	C16	0.93(12)
C13	C18	C17	C16	1.40(13)
C14	C15	C16	C17	0.19(13)
C14	C15	C16	C20	-178.61(12)
C15	C16	C17	C18	-1.40(13)
C16	C17	C18	C21	-177.53(12)
C22	C23	C24	C25	-179.26(11)
C22	C23	C28	C27	178.16(12)
C23	C24	C25	C26	0.95(14)
C23	C24	C25	Cl1	-178.45(10)
C23	C28	C27	C26	1.26(15)

Atom	Atom	Atom	Atom	Angle/°
C24	C25	C26	C27	-0.46(16)
C25	C26	C27	C28	-0.65(15)

Table S6: Hydrogen Fractional Atomic Coordinates (×10^4) and Equivalent Isotropic Displacement Parameters (Å^2×10^3) for **SM72049V2**. U_{eq} is defined as 1/3 of the trace of the orthogonalised U_{ij}.

Atom	x	y	z	U_{eq}
H2a	-690(20)	5007(12)	4744(16)	57(5)
H2b	-670(20)	5566(10)	5984(18)	54(4)
H3a	-460(20)	4282(16)	7210(17)	73(6)
H3b	-990(20)	3755(11)	5804(17)	50(4)
H6	3850(20)	7667(10)	8074(16)	50(4)
H8	3660(20)	7629(9)	4320(14)	43(4)
H10a	4130(30)	5650(12)	8648(16)	61(5)
H10b	2910(20)	6462(10)	9029(14)	48(4)
H10c	1660(30)	5642(14)	8099(16)	65(5)
H11a	3420(50)	9051(14)	6570(30)	121(11)
H11b	5580(40)	8755(14)	7090(20)	115(11)
H11c	4370(40)	8968(16)	5542(19)	105(9)
H12a	940(30)	5938(16)	3455(19)	76(6)
H12b	2840(30)	6399(11)	3147(13)	51(4)
H12c	2990(40)	5467(12)	3873(17)	89(8)
H15	3130(30)	1147(11)	6057(14)	54(5)
H17	4310(30)	2026(11)	9768(12)	50(5)
H19a	230(30)	2595(17)	4598(16)	82(7)
H19b	2140(40)	3184(15)	4689(16)	85(7)
H19c	2150(30)	2159(14)	4421(19)	75(6)
H20a	6080(30)	651(14)	8900(30)	105(10)
H20b	3920(30)	455(11)	9110(30)	77(7)
H20c	4260(40)	211(12)	7770(20)	107(9)
H21a	4370(40)	4073(14)	9330(20)	90(8)
H21b	2110(30)	4041(12)	9027(18)	73(6)
H21c	3480(40)	3453(13)	10168(16)	92(8)
H24	8500(30)	7784(10)	7501(18)	57(5)
H26	12050(30)	9078(11)	10625(17)	61(5)
H27	12210(30)	7787(13)	11895(15)	65(6)
H28	10350(30)	6521(10)	10942(15)	66(6)
H3	7000(30)	5765(13)	7010(20)	52(6)

V. Computational details

1. General remarks

Geometry optimizations were performed with the B3LYP hybrid density functional[25] and dispersion effects were accounted for by adding Grimme's D3 dispersion correction.[26] The def2-TZVPP basis set was used for all atoms.[27] A natural resonance theory (NRT) analysis was carried out using the NBO 6 program package at the same level of theory.[28] We additionally computed the harmonic vibrational frequencies for all structures, allowing the nature of the stationary points to be determined and providing thermodynamic corrections to the free energy at 298 K. The reaction paths associated with the optimized transition states were further confirmed by following the intrinsic reaction coordinate (IRC) which also aided in the determination of the nature of the overoxidation product (*S*,*S*-dioxide vs. oxathiirane *S*-oxide).[29] Final single point energy computations were performed using the revDSD-PBEP86 functional[30] with Grimme's D4 dispersion correction[31] and the def2-QZVPP basis set was used in combination with the RIJK approximation.[27, 32] Solvent effects were considered implicitly in the final single point computations using the SMD model for dichloromethane.[33] The free energy barrier was defined as the free energy difference between the transition state involved in the reaction and the reactants. The computed free energies were corrected regarding the 1 M standard state by adding RT ln(c_{0s}/c_{0g}) (i.e., about 1.89 kcal mol^{-1}) to the energies of all structures. The final single point energies were computed with the ORCA 4 program.[34] Otherwise, the *Gaussian16* program package was used throughout.[35]

2. Comparison of energetics of Me-S oxidation by acetic acid (Ox) and *m*-chloroperoxybenzoic acid (*m*CPBA).

Table S7: Free reaction energies and barrier heights of the two-step **Me-S** oxidation by peracetic (**Ox**) and *m*-chloroperoxybenzoic (***m*CPBA**) acids at the revDSD-PBEP86-D4/def2-QZVPP(SMD:dichloromethane) //B3LYP-D3/def2-TZVPP level of theory given in units of kcal mol^{-1}.

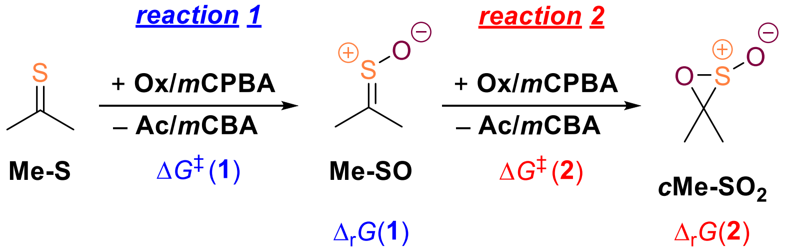

Oxidant	$\Delta G^{\ddagger}$(**1**)	$\Delta_r G$(**1**)	$\Delta G^{\ddagger}$(**2**)	$\Delta_r G$(**2**)
**Ox**	17.7	−55.3	24.5[a]	−54.3
***m*CPBA**[b]	16.3	−56.6	23.5	−55.5

[a]The barrier relates to the TS structure with the lowest energy; [b]The energetics here relate to thermodynamically preferred oxidation pathway with *syn*-conformer of *m*-chloroperoxybenzoic acid.

3. Optimized geometries (in Å) and energies (in E_h)

Ox

6	0.421962000	0.167453000	-0.000002000
8	-0.530907000	-0.795545000	-0.000005000
8	0.133727000	1.341691000	0.000001000
8	-1.856633000	-0.237358000	0.000004000
1	-1.657128000	0.725847000	-0.000001000
6	1.791175000	-0.425801000	0.000001000
1	2.320135000	-0.066743000	-0.883193000
1	2.320127000	-0.066753000	0.883204000
1	1.768542000	-1.512567000	-0.000005000

Gtherm = 0.035827
E(B3LYP) = -304.354696160
E(revDSD-PBEP86) = -303.911115481408

Ac

6	-0.087929000	0.123524000	0.000008000
8	-0.623038000	1.206896000	-0.000008000
8	-0.791246000	-1.028799000	-0.000001000

1	-1.737367000	-0.808117000	-0.000005000
6	1.388507000	-0.125960000	0.000002000
1	1.662055000	-0.710345000	-0.879629000
1	1.924043000	0.818562000	-0.000047000
1	1.662067000	-0.710256000	0.879690000

Gtherm = 0.034451
E(B3LYP) = -229.195034468
E(revDSD-PBEP86) = -228.853314154733

***m*CPBA**

6	-1.963176000	0.175171000	0.000302000
8	-2.201605000	-1.156549000	0.000060000
8	-2.866085000	0.981663000	0.000337000
8	-3.606915000	-1.434914000	-0.000125000
1	-3.973273000	-0.522550000	-0.000451000
6	-0.514857000	0.489392000	0.000134000
6	0.458828000	-0.511582000	0.000052000
6	-0.145921000	1.834891000	0.000064000
6	1.795953000	-0.146294000	-0.000086000
6	1.197847000	2.178037000	-0.000078000
6	2.176817000	1.191334000	-0.000153000
1	0.182249000	-1.554352000	0.000106000
1	-0.917330000	2.590918000	0.000130000
1	1.490307000	3.219011000	-0.000129000
1	3.225743000	1.450174000	-0.000262000
17	3.020953000	-1.386500000	-0.000175000

Gtherm = 0.072936
E(B3LYP) = -955.792712462
E(revDSD-PBEP86) = -954.687291635496

***m*CBA**

6	2.305002000	-0.398518000	-0.000307000
8	2.300130000	-1.752751000	-0.000215000
8	3.326400000	0.242798000	-0.000195000
6	0.937741000	0.188282000	-0.000118000
6	-0.203928000	-0.614304000	-0.000062000
6	0.819651000	1.578466000	0.000003000
6	-1.452299000	-0.010377000	0.000107000
6	-0.437666000	2.163446000	0.000171000
6	-1.582360000	1.373493000	0.000225000
1	-0.119416000	-1.689552000	-0.000153000
1	1.717925000	2.178544000	-0.000042000
1	-0.534337000	3.240548000	0.000262000
1	-2.565895000	1.820736000	0.000356000
17	-2.885386000	-1.006846000	0.000174000
1	3.224206000	-2.037195000	-0.000201000

Gtherm = 0.071754
E(B3LYP) = -880.634714938
E(revDSD-PBEP86) = -879.631660732096

IMe-S

6	0.000000000	0.333009000	0.000000000
7	1.086734000	-0.504716000	0.000001000
7	-1.086735000	-0.504713000	-0.000005000
6	0.675308000	-1.825365000	0.000002000
1	1.373118000	-2.641830000	-0.000002000
6	-0.675312000	-1.825364000	-0.000001000
1	-1.373124000	-2.641827000	-0.000002000
6	-2.454700000	-0.029688000	0.000001000
1	-2.637321000	0.584254000	0.881124000
1	-2.637313000	0.584294000	-0.881093000
1	-3.123352000	-0.887132000	-0.000015000
6	2.454700000	-0.029693000	0.000001000

1 3.123351000 -0.887137000 -0.000010000
1 2.637314000 0.584275000 -0.881105000
1 2.637320000 0.584265000 0.881112000
16 0.000002000 2.003090000 0.000000000
Gtherm = 0.095948
E(B3LYP) = -703.207559404
E(revDSD-PBEP86) = -702.383717198180

IMe-TS01
6 -1.512484000 -0.013288000 0.350724000
7 -2.054689000 -0.414902000 -0.830695000
7 -1.324521000 1.329986000 0.234645000
6 -2.187454000 0.663770000 -1.677184000
1 -2.596083000 0.568438000 -2.665747000
6 -1.729886000 1.751469000 -1.012958000
1 -1.662862000 2.779348000 -1.316625000
6 -0.643173000 2.155973000 1.221737000
1 -0.942141000 1.839226000 2.216912000
1 0.435690000 2.039747000 1.115597000
1 -0.927729000 3.193058000 1.061776000
6 -2.316814000 -1.805984000 -1.162915000
1 -2.744111000 -1.848521000 -2.161234000
1 -1.384551000 -2.366914000 -1.128360000
1 -3.010633000 -2.235996000 -0.444245000
16 -1.068013000 -1.015679000 1.641121000
6 2.924547000 -0.001044000 -0.234454000
8 2.452986000 -1.197624000 -0.381372000
8 2.403277000 0.885479000 0.444145000
8 0.922930000 -1.264299000 0.525275000
1 1.151540000 -0.398502000 0.921558000
6 4.225673000 0.238075000 -0.977751000
1 5.003647000 0.450084000 -0.244419000
1 4.514659000 -0.617320000 -1.582096000
1 4.109252000 1.120374000 -1.606281000
Gtherm = 0.148364
E(B3LYP) = -1007.55742150
E(revDSD-PBEP86) = -1006.280589809950

IMe-SO
6 -0.025326000 -0.115637000 0.000002000
7 -1.307331000 -0.598520000 0.000002000
7 -0.129052000 1.244350000 -0.000004000
6 -2.199021000 0.462116000 -0.000003000
1 -3.261923000 0.306966000 -0.000005000
6 -1.474011000 1.597703000 -0.000006000
1 -1.783558000 2.626238000 -0.000007000
16 1.354947000 -1.138300000 0.000004000
8 2.624623000 -0.295054000 -0.000013000
6 -1.625951000 -2.008587000 0.000002000
1 -1.204018000 -2.489241000 -0.885006000
1 -1.204018000 -2.489241000 0.885009000
1 -2.705644000 -2.130162000 0.000002000
6 0.964449000 2.211206000 0.000009000
1 0.895486000 2.838650000 0.889161000
1 1.905906000 1.669716000 -0.000009000
1 0.895471000 2.838689000 -0.889113000
Gtherm = 0.097022
E(B3LYP) = -778.413093137
E(revDSD-PBEP86) = -777.492005596943

IMe-TS02
6 -1.416576000 0.199558000 -0.278992000
7 -0.895329000 1.327876000 -0.823680000

7	-2.000980000	0.554636000	0.888222000
6	-1.128626000	2.391909000	0.028163000
1	-0.797975000	3.386456000	-0.206321000
6	-1.818643000	1.915188000	1.085475000
1	-2.206887000	2.413734000	1.953931000
16	-1.249277000	-1.382422000	-0.997951000
8	-2.179199000	-2.344721000	-0.301139000
6	-0.112386000	1.366129000	-2.048113000
1	0.896333000	0.991685000	-1.868310000
1	-0.598952000	0.749752000	-2.803686000
1	-0.064558000	2.393258000	-2.400123000
6	-2.526624000	-0.351625000	1.908158000
1	-3.365577000	0.133331000	2.403142000
1	-2.840691000	-1.275532000	1.434806000
1	-1.744870000	-0.571004000	2.635042000
6	3.151182000	-0.163342000	0.225185000
8	2.330079000	-0.679762000	1.107145000
8	2.896767000	-0.020349000	-0.961960000
8	0.891972000	-1.054582000	0.348450000
1	1.264723000	-0.996847000	-0.552020000
6	4.480039000	0.230198000	0.832455000
1	5.255501000	-0.390846000	0.384382000
1	4.686594000	1.266704000	0.568729000
1	4.491792000	0.107695000	1.911895000

Gtherm = 0.149603
E(B3LYP) = -1082.76963462
E(revDSD-PBEP86) = -1081.392454025771

IMe-SO_2

6	-0.318661000	-0.098750000	-0.064894000
7	-1.451987000	-0.816440000	-0.017091000
7	-0.692476000	1.189271000	-0.044833000
6	-2.553406000	0.022824000	0.035043000
1	-3.562220000	-0.345903000	0.070644000
6	-2.069948000	1.287735000	0.020922000
1	-2.579049000	2.234011000	0.045131000
16	1.718491000	-0.712234000	0.017999000
8	2.051724000	-0.019720000	1.268371000
6	-1.504062000	-2.271704000	-0.028142000
1	-2.024616000	-2.621800000	-0.918397000
1	-0.487990000	-2.654151000	-0.032640000
1	-2.020465000	-2.632502000	0.860034000
6	0.244760000	2.312960000	-0.027211000
1	0.563821000	2.510895000	0.993722000
1	1.114847000	2.053835000	-0.623129000
1	-0.250312000	3.186303000	-0.444854000
8	2.194435000	-0.038174000	-1.195787000

Gtherm = 0.097461
E(B3LYP) = -853.674842275
E(revDSD-PBEP86) = -852.660830535162

SIMe-S

6	0.000000000	0.000000000	0.392424000
7	-0.321086000	1.057276000	-0.416361000
7	0.321086000	-1.057276000	-0.416361000
6	0.000000000	0.763404000	-1.805367000
1	0.987395000	1.165858000	-2.066771000
1	-0.737651000	1.193471000	-2.482245000
6	0.000000000	-0.763404000	-1.805367000
1	-0.987395000	-1.165858000	-2.066771000
1	0.737651000	-1.193471000	-2.482245000
6	0.374658000	-2.427462000	0.034212000
1	0.709555000	-2.448014000	1.067571000

1 -0.608887000 -2.908149000 -0.024602000
1 1.075549000 -2.984388000 -0.588425000
6 -0.374658000 2.427462000 0.034212000
1 -1.075549000 2.984388000 -0.588425000
1 -0.709555000 2.448014000 1.067571000
1 0.608887000 2.908149000 -0.024602000
16 0.000000000 0.000000000 2.057332000
Gtherm = 0.119681
E(B3LYP) = -704.418521646
E(revDSD-PBEP86) = -703.587644083382

SIMe-TS01
6 -1.552948000 -0.199570000 0.272869000
7 -1.180819000 1.074326000 0.515648000
7 -2.704367000 -0.195721000 -0.444702000
6 -2.004340000 2.004411000 -0.251380000
1 -1.484026000 2.301034000 -1.168539000
1 -2.224960000 2.898565000 0.329273000
6 -3.241938000 1.156344000 -0.547852000
1 -4.031719000 1.311265000 0.196810000
1 -3.658746000 1.335571000 -1.537764000
6 -3.541206000 -1.346207000 -0.692167000
1 -2.922806000 -2.239576000 -0.713019000
1 -4.303695000 -1.461625000 0.085116000
1 -4.036501000 -1.227136000 -1.655787000
6 0.077222000 1.505113000 1.088626000
1 -0.080612000 2.462123000 1.585538000
1 0.416205000 0.776497000 1.818312000
1 0.848403000 1.605855000 0.324019000
16 -0.726376000 -1.593471000 0.755443000
6 3.444123000 -0.014381000 -0.199064000
8 2.603188000 -0.331463000 -1.134881000
8 3.218127000 -0.105090000 1.005756000
8 1.127286000 -0.922000000 -0.319856000
1 1.601194000 -0.841749000 0.537848000
6 4.780072000 0.480597000 -0.722873000
1 5.051369000 1.389422000 -0.187820000
1 5.532317000 -0.276792000 -0.500779000
1 4.757182000 0.662428000 -1.794016000
Gtherm = 0.171764
E(B3LYP) = -1008.76500434
E(revDSD-PBEP86) = -1007.483564957458

SIMe-SO
6 -0.034901000 -0.132092000 -0.058547000
7 0.133543000 1.203050000 0.147092000
7 1.194028000 -0.727251000 -0.238199000
16 -1.472504000 -1.031392000 -0.031055000
8 -2.677540000 -0.142835000 0.197095000
6 1.546876000 1.542826000 -0.012714000
1 1.843752000 2.311992000 0.699279000
1 1.737917000 1.912107000 -1.028365000
6 2.224040000 0.199227000 0.227659000
1 2.435557000 0.047303000 1.294609000
1 3.146862000 0.070721000 -0.335671000
6 -0.859057000 2.222161000 -0.156377000
1 -0.684863000 3.084829000 0.486849000
1 -0.775737000 2.542203000 -1.201769000
1 -1.853237000 1.823387000 0.018671000
6 1.379813000 -2.150466000 -0.054016000
1 2.400541000 -2.408606000 -0.330370000
1 1.196218000 -2.459344000 0.982119000
1 0.699756000 -2.700152000 -0.703517000

Gtherm = 0.121395
E(B3LYP) = -779.628267201
E(revDSD-PBEP86) = -778.700502494030

SIMe-TS02

6	1.439352000	0.096477000	0.335136000
7	0.975906000	1.090108000	1.134424000
7	1.978590000	0.601305000	-0.775267000
6	0.847661000	2.310259000	0.334596000
1	1.070177000	3.190802000	0.934458000
1	-0.170385000	2.391653000	-0.061320000
6	1.865462000	2.061725000	-0.779891000
1	1.523121000	2.410702000	-1.753330000
1	2.838110000	2.515762000	-0.566848000
16	1.237785000	-1.576741000	0.773477000
8	2.041473000	-2.468846000	-0.128621000
6	0.075823000	0.870281000	2.250514000
1	-0.944409000	0.661185000	1.915733000
1	0.431793000	0.034374000	2.850333000
1	0.075897000	1.762300000	2.874903000
6	2.582343000	-0.096443000	-1.895603000
1	3.592515000	0.284953000	-2.061825000
1	2.621484000	-1.159376000	-1.685401000
1	1.987717000	0.076225000	-2.794922000
6	-3.182350000	-0.153425000	-0.277943000
8	-2.241960000	-0.229907000	-1.185850000
8	-3.036804000	-0.450815000	0.899016000
8	-0.822886000	-0.796236000	-0.434248000
1	-1.295366000	-1.048967000	0.385005000
6	-4.501265000	0.331793000	-0.842319000
1	-5.205141000	-0.500307000	-0.818497000
1	-4.887853000	1.117491000	-0.195019000
1	-4.404438000	0.693601000	-1.862310000

Gtherm = 0.172699
E(B3LYP) = -1083.98172424
E(revDSD-PBEP86) = -1082.597665200503

SIMe-SO_2

6	0.267777000	-0.098152000	0.118294000
7	1.298079000	-0.923743000	-0.002473000
7	0.679460000	1.157092000	0.123715000
6	2.583734000	-0.203384000	-0.008493000
1	3.205787000	-0.529855000	-0.841540000
1	3.123119000	-0.399461000	0.922019000
6	2.128185000	1.259728000	-0.129058000
1	2.601240000	1.918609000	0.598196000
1	2.298631000	1.669027000	-1.128306000
16	-1.807661000	-0.642632000	-0.019881000
8	-2.038739000	-0.000282000	-1.318790000
6	1.241319000	-2.365844000	0.088149000
1	1.714811000	-2.715194000	1.009708000
1	0.202782000	-2.684486000	0.081486000
1	1.757094000	-2.817597000	-0.760995000
6	-0.174024000	2.330287000	0.077139000
1	-0.186915000	2.751586000	-0.930532000
1	-1.184077000	2.053146000	0.361050000
1	0.205425000	3.079557000	0.773118000
8	-2.328767000	0.098724000	1.132416000

Gtherm = 0.120290
E(B3LYP) = -854.882906019
E(revDSD-PBEP86) = -853.861452219196

SIMe

6	0.000000000	0.000000000	1.013541000
7	0.000000000	-1.072272000	0.207483000
7	0.000000000	1.072272000	0.207483000
6	-0.102232000	-0.758840000	-1.228719000
1	-1.086488000	-1.048663000	-1.611634000
1	0.652857000	-1.295520000	-1.805628000
6	0.102232000	0.758840000	-1.228719000
1	1.086488000	1.048663000	-1.611634000
1	-0.652857000	1.295520000	-1.805628000
6	0.137221000	2.433138000	0.658808000
1	0.051629000	2.447722000	1.741797000
1	1.109137000	2.852134000	0.373591000
1	-0.643000000	3.067943000	0.228339000
6	-0.137221000	-2.433138000	0.658808000
1	0.643000000	-3.067943000	0.228339000
1	-0.051629000	-2.447722000	1.741797000
1	-1.109137000	-2.852134000	0.373591000

Gtherm = 0.117741
E(B3LYP) = -306.131160581
E(revDSD-PBEP86) = -305.611394809533

SO_2

16	0.000000000	0.000000000	0.365001000
8	0.000000000	1.239648000	-0.365001000
8	0.000000000	-1.239648000	-0.365001000

Gtherm = -0.017162
E(B3LYP) = -548.722715223
E(revDSD-PBEP86) = -548.223082789361

NMP-S

6	0.416461000	-0.341158000	0.011864000
7	-0.336005000	0.774207000	-0.030055000
6	-1.910115000	-0.955381000	-0.211058000
1	-2.726242000	-1.429263000	0.330515000
1	-2.100258000	-1.072484000	-1.278224000
6	-1.768374000	0.531382000	0.127286000
1	-2.336993000	1.184786000	-0.536141000
1	-2.074422000	0.753606000	1.156530000
16	2.065995000	-0.442794000	-0.025831000
6	0.175152000	2.125604000	-0.020292000
1	0.055034000	2.577022000	0.969027000
1	1.232309000	2.104242000	-0.271388000
1	-0.368050000	2.730548000	-0.747839000
6	-0.528754000	-1.521774000	0.143236000
1	-0.207326000	-2.348593000	-0.484371000
1	-0.484158000	-1.866649000	1.179346000

Gtherm = 0.104276
E(B3LYP) = -649.039662540
E(revDSD-PBEP86) = -648.292435301546

***E*-NMP-TS01**

6	1.441507000	-0.215427000	-0.077232000
7	2.723485000	0.116342000	0.068713000
6	1.678370000	2.134516000	-0.520139000
1	1.399849000	3.097441000	-0.098566000
1	1.831589000	2.266802000	-1.591043000
6	2.948693000	1.566288000	0.117031000
1	3.861526000	1.819156000	-0.422206000
1	3.072178000	1.881100000	1.158504000
16	0.826384000	-1.766258000	-0.010973000
6	3.788551000	-0.811135000	0.393674000
1	3.591679000	-1.772225000	-0.075552000

1	4.733036000	-0.412519000	0.027830000
1	3.857836000	-0.960584000	1.474159000
6	0.627793000	1.042481000	-0.268632000
1	-0.096375000	0.912053000	-1.067318000
1	0.045658000	1.211893000	0.640594000
6	-3.242995000	0.182634000	0.218248000
8	-2.933110000	-0.541536000	-0.813212000
8	-2.520599000	0.347229000	1.200424000
8	-1.311919000	-1.181094000	-0.519739000
1	-1.375165000	-0.850673000	0.403242000
6	-4.615670000	0.820830000	0.124384000
1	-5.250004000	0.378658000	0.892832000
1	-4.521500000	1.884369000	0.340043000
1	-5.069300000	0.672362000	-0.851745000

Gtherm = 0.156006
E(B3LYP) = -953.387610825
E(revDSD-PBEP86) = -952.189274358970

***E*-NMP-SO**

6	0.141784000	-0.079045000	-0.040685000
7	-0.969157000	0.691408000	-0.122090000
6	-2.172574000	-0.077439000	0.197931000
1	-3.023054000	0.298639000	-0.371196000
1	-2.412748000	0.007397000	1.265973000
6	-1.763507000	-1.505140000	-0.178437000
1	-2.300787000	-2.256923000	0.395581000
1	-1.966222000	-1.676386000	-1.235605000
16	1.716904000	0.461407000	-0.008571000
8	2.649906000	-0.718761000	0.033936000
6	-0.925257000	2.129879000	-0.021200000
1	-0.628808000	2.456283000	0.983066000
1	-0.198263000	2.526244000	-0.732795000
1	-1.904834000	2.541243000	-0.255604000
6	-0.243846000	-1.525787000	0.076404000
1	0.323698000	-2.146913000	-0.614023000
1	0.005808000	-1.876682000	1.080798000

Gtherm = 0.106136
E(B3LYP) = -724.257942109
E(revDSD-PBEP86) = -723.413974576132

***Z*-NMP-TS01**

6	1.821638000	0.501883000	-0.088106000
7	1.557363000	-0.782405000	-0.303284000
6	3.820913000	-0.768350000	0.331974000
1	4.484487000	-1.065738000	1.140742000
1	4.389257000	-0.789115000	-0.597921000
6	2.598544000	-1.681218000	0.210807000
1	2.741119000	-2.514295000	-0.476711000
1	2.283599000	-2.089198000	1.176297000
16	0.831770000	1.817433000	-0.365624000
6	0.336219000	-1.326977000	-0.871500000
1	-0.410431000	-1.499441000	-0.097752000
1	-0.079491000	-0.624624000	-1.587918000
1	0.576890000	-2.262383000	-1.373967000
6	3.195284000	0.617354000	0.538793000
1	3.761177000	1.436946000	0.102070000
1	3.054487000	0.851806000	1.596768000
6	-3.264767000	-0.163948000	0.056354000
8	-2.526245000	0.141397000	1.082252000
8	-2.956497000	0.047541000	-1.113171000
8	-1.061486000	0.900815000	0.452076000
1	-1.451039000	0.881817000	-0.449844000
6	-4.585140000	-0.808694000	0.432857000

1	-4.732573000	-1.691830000	-0.186939000
1	-4.626037000	-1.072416000	1.486237000
1	-5.383625000	-0.101945000	0.205582000

Gtherm = 0.157720
E(B3LYP) = -953.386807639
E(revDSD-PBEP86) = -952.188173305104

***Z*-NMP-SO**

6	0.041404000	-0.398669000	0.063503000
7	-0.571301000	0.798540000	-0.014657000
6	-2.277950000	-0.784648000	-0.294058000
1	-3.189703000	-1.174185000	0.153883000
1	-2.349620000	-0.903426000	-1.375493000
6	-2.028228000	0.685204000	0.049561000
1	-2.488566000	1.380849000	-0.654219000
1	-2.384822000	0.941484000	1.054997000
16	1.652429000	-0.806019000	0.002340000
8	2.571052000	0.381181000	-0.138186000
6	0.051586000	2.107403000	0.070060000
1	-0.185063000	2.574708000	1.031435000
1	1.127943000	2.006305000	-0.026567000
1	-0.333686000	2.747453000	-0.726211000
6	-1.008616000	-1.476892000	0.229833000
1	-0.760860000	-2.398009000	-0.292574000
1	-1.112969000	-1.712487000	1.292012000

Gtherm = 0.106733
E(B3LYP) = -724.261064859
E(revDSD-PBEP86) = -723.415499322634

***E*-NMP-TS02**

6	1.564045000	0.012794000	0.067114000
7	1.227769000	1.098975000	0.749061000
6	1.193809000	2.288718000	-0.113517000
1	1.517622000	3.164768000	0.447587000
1	0.166670000	2.453417000	-0.454397000
6	2.130606000	1.900476000	-1.261932000
1	1.848970000	2.371080000	-2.200661000
1	3.150972000	2.200538000	-1.024382000
16	1.401191000	-1.604525000	0.592636000
8	2.054320000	-2.491577000	-0.411031000
6	0.556786000	1.086631000	2.034354000
1	-0.518762000	0.928754000	1.909170000
1	0.965096000	0.286599000	2.651452000
1	0.726193000	2.038388000	2.534011000
6	2.026587000	0.364028000	-1.310251000
1	2.949512000	-0.150902000	-1.568213000
1	1.260102000	0.012359000	-2.004751000
6	-2.993090000	-0.056259000	-0.096440000
8	-2.270678000	-0.545079000	-1.062564000
8	-2.613636000	0.069081000	1.064810000
8	-0.713786000	-0.956344000	-0.389663000
1	-1.065888000	-0.813885000	0.514280000
6	-4.389011000	0.336474000	-0.534968000
1	-5.093325000	-0.362101000	-0.082780000
1	-4.607893000	1.331308000	-0.150119000
1	-4.500872000	0.313439000	-1.615386000

Gtherm = 0.159267
E(B3LYP) = -1028.60825974
E(revDSD-PBEP86) = -1027.310228514068

NMP-SO_2

6	0.256217000	-0.362295000	-0.253605000
7	0.860656000	0.755889000	-0.051197000

6	2.572031000	-0.863558000	0.149161000
1	3.433880000	-1.188223000	-0.428646000
1	2.740364000	-1.143543000	1.187782000
6	2.338520000	0.648737000	0.043106000
1	2.685877000	1.213383000	0.907817000
1	2.773118000	1.095469000	-0.853528000
16	-1.745048000	-0.610646000	-0.080506000
8	-2.252849000	0.482111000	-0.929775000
6	0.225249000	2.063832000	0.097248000
1	0.712572000	2.766407000	-0.578706000
1	-0.830988000	1.983035000	-0.138390000
1	0.357903000	2.401964000	1.124526000
8	-1.744694000	-0.304579000	1.367139000
6	1.242590000	-1.475818000	-0.351737000
1	0.908913000	-2.341252000	0.219852000
1	1.287232000	-1.793772000	-1.398176000

Gtherm = 0.105603
E(B3LYP) = -799.494174442
E(revDSD-PBEP86) = -798.562215969091

DO-S

6	0.000000000	0.000000000	0.416253000
8	0.000000000	1.105468000	-0.352160000
8	0.000000000	-1.105468000	-0.352160000
6	0.189947000	0.737573000	-1.732105000
1	1.235185000	0.906375000	-1.994827000
1	-0.452474000	1.361937000	-2.347264000
6	-0.189947000	-0.737573000	-1.732105000
1	-1.235185000	-0.906375000	-1.994827000
1	0.452474000	-1.361937000	-2.347264000
16	0.000000000	0.000000000	2.037905000

Gtherm = 0.043250
E(B3LYP) = -665.491385114
E(revDSD-PBEP86) = -664.778762014482

DO-TS01

6	-1.744926000	0.375678000	-0.037339000
8	-3.059342000	0.228884000	-0.180252000
8	-1.136697000	-0.761525000	0.246432000
6	-3.424445000	-1.130447000	0.177153000
1	-4.147286000	-1.487070000	-0.550448000
1	-3.868974000	-1.108065000	1.171319000
6	-2.081680000	-1.853071000	0.132612000
1	-1.912164000	-2.531285000	0.963252000
1	-1.892641000	-2.360117000	-0.812518000
16	-0.973555000	1.820428000	-0.210059000
6	2.920118000	-0.325413000	-0.096219000
8	2.671292000	0.586403000	0.778112000
8	2.147690000	-0.654459000	-1.004642000
8	0.983368000	1.203657000	0.342306000
1	1.065130000	0.563125000	-0.413398000
6	4.276789000	-0.991259000	0.050384000
1	4.879759000	-0.734275000	-0.820813000
1	4.788921000	-0.673941000	0.954653000
1	4.138510000	-2.071814000	0.053716000

Gtherm = 0.093580
E(B3LYP) = -969.828099875
E(revDSD-PBEP86) = -968.668065271167

DO-SO

6	-0.017620000	-0.160703000	-0.028195000
8	0.908719000	-1.131949000	-0.145621000
8	0.516774000	1.058864000	0.069952000

6	2.185433000	-0.539434000	0.184096000
1	2.944824000	-1.009183000	-0.434141000
1	2.395582000	-0.714804000	1.240657000
6	1.944624000	0.931345000	-0.127492000
1	2.442033000	1.618453000	0.550953000
1	2.176640000	1.187229000	-1.161996000
16	-1.647743000	-0.496390000	-0.006237000
8	-2.459219000	0.757247000	0.042403000

Gtherm = 0.044019
E(B3LYP) = -740.700290813
E(revDSD-PBEP86) = -739.892386852406

DO-TS02

6	1.535735000	0.336261000	0.153982000
8	1.116026000	1.168799000	1.085850000
8	1.609311000	0.846210000	-1.045912000
6	0.455967000	2.280435000	0.403280000
1	0.705193000	3.187931000	0.943571000
1	-0.613856000	2.080151000	0.429843000
6	1.061181000	2.200204000	-0.999517000
1	0.327156000	2.280788000	-1.795431000
1	1.885112000	2.891026000	-1.166277000
16	1.897851000	-1.298549000	0.544402000
8	2.470514000	-1.983707000	-0.629803000
6	-2.706847000	-0.273182000	0.046348000
8	-2.061955000	-1.213367000	-0.565149000
8	-2.214132000	0.499111000	0.870813000
8	-0.348226000	-1.136547000	-0.011092000
1	-0.657925000	-0.616146000	0.761007000
6	-4.176055000	-0.204498000	-0.327006000
1	-4.417297000	-0.858303000	-1.160361000
1	-4.760140000	-0.495568000	0.546235000
1	-4.426052000	0.827588000	-0.569196000

Gtherm = 0.096564
E(B3LYP) = -1045.04486567
E(revDSD-PBEP86) = -1043.783351882619

DO-SO_2

6	0.602113000	0.034620000	0.537222000
8	1.444890000	-0.955382000	0.631419000
8	1.125542000	1.051629000	-0.084022000
6	2.742205000	-0.634820000	-0.010260000
1	2.901913000	-1.371419000	-0.792086000
1	3.509266000	-0.721039000	0.753755000
6	2.515701000	0.777302000	-0.516071000
1	3.150013000	1.527767000	-0.053627000
1	2.538686000	0.876136000	-1.597282000
16	-2.071245000	-0.258727000	0.233933000
8	-2.453747000	1.132883000	0.245205000
8	-1.881695000	-0.883432000	-1.057481000

Gtherm = 0.038017
E(B3LYP) = -815.947630519
E(revDSD-PBEP86) = -815.037600873169

DT-S

6	0.000000000	0.000000000	0.681258000
16	0.000000000	1.468902000	-0.280711000
16	0.000000000	-1.468902000	-0.280711000
6	0.336171000	0.682560000	-1.893744000
1	1.414557000	0.592354000	-2.020818000
1	-0.062728000	1.326142000	-2.677328000
6	-0.336171000	-0.682560000	-1.893744000
1	-1.414557000	-0.592354000	-2.020818000

1	0.062728000	-1.326142000	-2.677328000
16	0.000000000	0.000000000	2.313527000

Gtherm = 0.034784
E(B3LYP) = -1311.43685055
E(revDSD-PBEP86) = -1310.302868604215

DT-TS01

6	-1.404614000	0.547569000	0.011224000
16	-3.138288000	0.634611000	0.146342000
16	-0.733412000	-1.052521000	-0.069595000
6	-3.453121000	-1.129205000	-0.217717000
1	-3.529895000	-1.249333000	-1.297748000
1	-4.400924000	-1.403708000	0.243617000
6	-2.290173000	-1.919572000	0.353238000
1	-2.357835000	-1.999399000	1.437988000
1	-2.236656000	-2.920381000	-0.074299000
16	-0.487181000	1.917847000	-0.047374000
6	3.501516000	-0.183955000	0.122444000
8	2.739836000	-0.239160000	-0.925960000
8	3.238852000	0.457225000	1.138526000
8	1.318989000	0.793566000	-0.543398000
1	1.751736000	0.984119000	0.327898000
6	4.783950000	-0.983699000	-0.004719000
1	5.621801000	-0.286383000	0.001127000
1	4.879064000	-1.629078000	0.867481000
1	4.808054000	-1.574707000	-0.916197000

Gtherm = 0.086129
E(B3LYP) = -1615.77932063
E(revDSD-PBEP86) = -1614.196859689372

DT-SO

6	-0.310973000	-0.210600000	-0.044692000
16	0.922866000	-1.449044000	-0.187109000
16	0.246283000	1.444096000	-0.027289000
6	2.241559000	-0.351916000	0.446691000
1	3.197431000	-0.786292000	0.155589000
1	2.181322000	-0.303661000	1.533058000
6	2.021653000	1.013262000	-0.184785000
1	2.590272000	1.789203000	0.327276000
1	2.291302000	1.008216000	-1.239841000
16	-1.914369000	-0.590569000	0.066513000
8	-2.756280000	0.639542000	0.035849000

Gtherm = 0.035975
E(B3LYP) = -1386.66291088
E(revDSD-PBEP86) = -1385.434083946348

DT-TS02

6	-1.352603000	0.267127000	0.257032000
16	-1.183209000	-0.864288000	1.552120000
16	-1.847241000	-0.335258000	-1.278832000
6	-0.952833000	-2.280764000	0.412646000
1	-1.156529000	-3.194340000	0.969353000
1	0.083653000	-2.262566000	0.077252000
6	-1.926689000	-2.088440000	-0.737851000
1	-1.653971000	-2.701340000	-1.596067000
1	-2.952762000	-2.309393000	-0.446462000
16	-0.977119000	1.916691000	0.443107000
8	-1.375898000	2.633455000	-0.784352000
6	2.935616000	-0.248697000	-0.196965000
8	2.873176000	0.978139000	0.202648000
8	1.954482000	-0.954506000	-0.452076000
8	1.108606000	1.353640000	0.329617000
1	0.965923000	0.462739000	-0.067873000

6	4.348894000	-0.778083000	-0.339020000
1	4.487661000	-1.110311000	-1.367379000
1	5.092548000	-0.027772000	-0.086616000
1	4.457338000	-1.644009000	0.313724000

Gtherm = 0.089459
E(B3LYP) = -1691.00387761
E(revDSD-PBEP86) = -1689.323321651033

DT-SO_2

6	0.232833000	-0.070919000	-0.319105000
16	1.194283000	-1.413464000	-0.263963000
16	0.952092000	1.382877000	-0.004760000
6	2.787030000	-0.650530000	0.370866000
1	3.619786000	-1.137156000	-0.132484000
1	2.829000000	-0.875945000	1.435295000
6	2.745401000	0.842834000	0.101000000
1	3.222546000	1.416889000	0.892519000
1	3.200130000	1.101463000	-0.854016000
16	-2.483001000	-0.102740000	-0.296641000
8	-2.638899000	1.261326000	0.157155000
8	-2.620730000	-1.148867000	0.691338000

Gtherm = 0.030452
E(B3LYP) = -1461.89278747
E(revDSD-PBEP86) = -1460.560718828731

DAN-S

7	0.000000000	1.140986000	-0.259478000
6	1.199105000	0.787026000	-1.070736000
6	1.199105000	-0.787026000	-1.070736000
7	0.000000000	-1.140986000	-0.259478000
6	-1.199105000	-0.787026000	-1.070736000
6	-1.199105000	0.787026000	-1.070736000
1	2.084056000	1.188877000	-0.581767000
1	1.117296000	1.229074000	-2.061341000
1	1.117296000	-1.229074000	-2.061341000
1	2.084056000	-1.188877000	-0.581767000
6	0.000000000	0.000000000	0.651604000
1	-1.117296000	-1.229074000	-2.061341000
1	-2.084056000	-1.188877000	-0.581767000
1	-2.084056000	1.188877000	-0.581767000
1	-1.117296000	1.229074000	-2.061341000
16	0.000000000	0.000000000	2.249573000

Gtherm = 0.101080
E(B3LYP) = -703.123203623
E(revDSD-PBEP86) = -702.309249247842

DAN-TS01

7	-2.618662000	0.219049000	-0.773778000
6	-2.105736000	-1.050636000	-1.389119000
6	-1.080014000	-1.598507000	-0.326921000
7	-1.120486000	-0.566986000	0.758237000
6	-2.436112000	-0.696121000	1.450994000
6	-3.470610000	-0.169138000	0.390032000
1	-1.618748000	-0.807918000	-2.330707000
1	-2.942468000	-1.716158000	-1.588230000
1	-1.348207000	-2.567160000	0.087403000
1	-0.065005000	-1.649481000	-0.710977000
6	-1.413927000	0.584504000	-0.060242000
1	-2.590770000	-1.731164000	1.745366000
1	-2.424404000	-0.076594000	2.345144000
1	-4.000544000	0.711749000	0.745634000
1	-4.200351000	-0.911602000	0.076344000
16	-0.542145000	1.924525000	-0.219139000

6	3.192208000	-0.395341000	0.045608000
8	3.196544000	0.895389000	0.107035000
8	2.177263000	-1.094001000	0.084173000
8	1.445799000	1.392860000	0.184591000
1	1.249400000	0.433601000	0.342091000
6	4.572614000	-1.014334000	-0.074892000
1	4.715472000	-1.700392000	0.759338000
1	4.608709000	-1.594555000	-0.996773000
1	5.357883000	-0.263717000	-0.078777000

Gtherm = 0.152881
E(B3LYP) = -1007.46207693
E(revDSD-PBEP86) = -1006.194262287488

DAN-SO

7	-0.565646000	-1.052156000	0.000000000
6	-1.381832000	-0.695662000	1.202651000
6	-1.381832000	0.878700000	1.202354000
7	-0.563556000	1.227687000	0.000000000
6	-1.381832000	0.878700000	-1.202354000
6	-1.381832000	-0.695662000	-1.202651000
1	-0.893092000	-1.098374000	2.086798000
1	-2.372488000	-1.137291000	1.118845000
1	-2.372367000	1.320966000	1.119290000
1	-0.892459000	1.280887000	2.086499000
6	0.313938000	0.081248000	0.000000000
1	-2.372367000	1.320966000	-1.119290000
1	-0.892459000	1.280887000	-2.086499000
1	-0.893092000	-1.098374000	-2.086798000
1	-2.372488000	-1.137291000	-1.118845000
16	1.928037000	0.230810000	0.000000000
8	2.674622000	-1.042250000	0.000000000

Gtherm = 0.102895
E(B3LYP) = -778.356142042
E(revDSD-PBEP86) = -777.445473694929

DAN-TS02

7	-1.561301000	-0.491143000	-1.105846000
6	-0.636254000	-1.667168000	-1.107625000
6	-0.313912000	-1.895995000	0.410221000
7	-1.095066000	-0.822367000	1.100377000
6	-2.541169000	-1.209963000	0.987220000
6	-2.865493000	-0.965202000	-0.539318000
1	0.255622000	-1.408049000	-1.668000000
1	-1.131940000	-2.509038000	-1.584707000
1	-0.627235000	-2.865365000	0.789441000
1	0.741191000	-1.759941000	0.625133000
6	-1.101797000	0.186580000	0.067287000
1	-2.670890000	-2.240367000	1.309199000
1	-3.130910000	-0.563833000	1.633089000
1	-3.609944000	-0.183615000	-0.672784000
1	-3.191050000	-1.855590000	-1.071188000
16	-1.255481000	1.803623000	0.475331000
6	3.156582000	0.033224000	0.126804000
8	2.405014000	0.226954000	-0.927695000
8	2.749157000	0.124456000	1.277156000
8	0.824959000	0.642350000	-0.331374000
1	1.171574000	0.604070000	0.599494000
6	4.590886000	-0.299083000	-0.217764000
1	5.204295000	0.568358000	0.027134000
1	4.913968000	-1.130256000	0.406461000
1	4.713315000	-0.540107000	-1.270016000
8	-1.690227000	2.646988000	-0.649488000

Gtherm = 0.157128

E(B3LYP) = -1082.69348827
E(revDSD-PBEP86) = -1081.331093742023

***c*DAN-SO_2**

7	0.917458000	-0.219858000	-1.110333000
6	1.306295000	1.208422000	-0.985078000
6	1.013171000	1.545672000	0.525790000
7	0.513679000	0.264718000	1.081244000
6	1.663552000	-0.686398000	1.134971000
6	1.937564000	-1.028667000	-0.377996000
1	0.688977000	1.805501000	-1.654562000
1	2.346918000	1.337276000	-1.273563000
1	1.885933000	1.879592000	1.081969000
1	0.232571000	2.298279000	0.623799000
6	-0.136763000	-0.261851000	-0.105903000
1	2.503521000	-0.217623000	1.642186000
1	1.363621000	-1.568408000	1.695860000
1	1.764375000	-2.082325000	-0.585365000
1	2.935077000	-0.764512000	-0.721211000
16	-1.883844000	-0.047897000	-0.441582000
8	-0.915512000	-1.406705000	0.001924000
8	-2.622033000	0.544390000	0.661716000

Gtherm = 0.106833
E(B3LYP) = -853.611309286
E(revDSD-PBEP86) = -852.603553080498

Im-S

6	-0.008171000	0.750270000	0.021718000
16	-0.009645000	2.383262000	0.013821000
7	-2.604849000	-1.635418000	-0.520738000
6	-3.272653000	-0.625036000	0.154920000
6	-2.422302000	0.380418000	0.479348000
7	-1.170507000	-0.021588000	0.024080000
6	-1.367133000	-1.262138000	-0.586038000
1	-4.326112000	-0.693834000	0.364450000
1	-2.555241000	1.320512000	0.979603000
1	-0.564001000	-1.787647000	-1.072034000
7	3.301814000	-0.515566000	-0.300557000
6	2.704451000	-1.512408000	0.452842000
6	1.392813000	-1.237030000	0.662643000
7	1.160850000	-0.013202000	0.025883000
6	2.377371000	0.365024000	-0.524358000
1	3.263202000	-2.363768000	0.802103000
1	0.625096000	-1.744792000	1.214576000
1	2.473974000	1.283157000	-1.076950000

Gtherm = 0.093915
E(B3LYP) = -887.745074815
E(revDSD-PBEP86) = -886.633503408970

Im-TS01

6	-3.345290000	-0.498939000	0.737891000
8	-3.408234000	-1.045907000	-0.427917000
8	-2.294711000	-0.366761000	1.380834000
8	-1.650331000	-1.434201000	-0.818776000
1	-1.518483000	-1.111405000	0.118776000
6	-4.666815000	-0.007628000	1.289588000
1	-5.493840000	-0.235151000	0.623084000
1	-4.829771000	-0.470856000	2.262001000
1	-4.595711000	1.069485000	1.440379000
6	1.048964000	-0.319835000	-0.469543000
16	0.445819000	-1.757818000	-1.032594000
7	4.170734000	-0.924888000	1.109656000
6	4.356487000	0.356739000	0.612683000

6	3.238216000	0.799792000	-0.011067000
7	2.316897000	-0.251341000	0.083609000
6	2.971206000	-1.271133000	0.774434000
1	5.291973000	0.874985000	0.734998000
1	3.007654000	1.708840000	-0.533181000
1	2.472107000	-2.197121000	1.001996000
7	-0.406939000	2.843667000	0.182460000
6	-1.079920000	2.448913000	-0.971294000
6	-0.644457000	1.242265000	-1.397315000
7	0.367779000	0.872712000	-0.508015000
6	0.439130000	1.910040000	0.442940000
1	-1.840448000	3.068751000	-1.412869000
1	-0.947364000	0.597726000	-2.198572000
1	1.102546000	1.852443000	1.287900000

Gtherm = 0.146059
E(B3LYP) = -1192.08008560
E(revDSD-PBEP86) = -1190.516847255554

Im-SO

6	-0.028596000	0.472858000	0.040977000
16	-0.125764000	2.126899000	0.108609000
7	3.362497000	-0.422624000	-0.603112000
6	3.113309000	-1.102672000	0.570957000
6	1.835308000	-0.890769000	0.990148000
7	1.273147000	-0.042903000	0.042208000
6	2.260410000	0.200416000	-0.896200000
1	3.871928000	-1.701052000	1.045935000
1	1.275521000	-1.232994000	1.841284000
1	2.070961000	0.826231000	-1.752491000
8	-1.508300000	2.645550000	-0.026627000
7	-3.130227000	-1.265137000	0.069626000
6	-2.241734000	-2.259730000	-0.302129000
6	-0.975602000	-1.779165000	-0.367119000
7	-1.072286000	-0.426652000	-0.022634000
6	-2.426244000	-0.187636000	0.224481000
1	-2.575992000	-3.262988000	-0.503457000
1	-0.036177000	-2.233974000	-0.617131000
1	-2.780633000	0.791380000	0.491830000

Gtherm = 0.094912
E(B3LYP) = -962.970859754
E(revDSD-PBEP86) = -961.761889198836

Im-TS02

6	3.399504000	-0.479406000	-0.467419000
8	2.702302000	-1.526059000	-0.174073000
8	3.043708000	0.676573000	-0.215010000
8	1.183594000	-0.811726000	0.632268000
1	1.666696000	0.058330000	0.490988000
6	4.718950000	-0.772956000	-1.151424000
1	4.776284000	-0.177429000	-2.061532000
1	5.521982000	-0.453184000	-0.487360000
1	4.831677000	-1.828331000	-1.382218000
6	-1.038418000	0.148370000	0.491320000
16	-0.315924000	-0.272899000	1.988887000
7	-0.707679000	3.668799000	0.220884000
6	-0.307963000	3.249462000	-1.036708000
6	-0.400362000	1.900445000	-1.151085000
7	-0.861926000	1.455768000	0.089360000
6	-1.016707000	2.597381000	0.876723000
1	0.035090000	3.950433000	-1.777863000
1	-0.148816000	1.209639000	-1.933793000
1	-1.392618000	2.533178000	1.884082000
7	-2.431907000	-2.606879000	-1.227684000

6	-3.025369000	-1.529170000	-1.875989000
6	-2.604822000	-0.356234000	-1.352264000
7	-1.718494000	-0.705617000	-0.319147000
6	-1.673243000	-2.110907000	-0.309422000
1	-3.726191000	-1.678364000	-2.679168000
1	-2.840421000	0.667422000	-1.571705000
1	-1.050233000	-2.642799000	0.386080000
8	-0.790607000	-1.568650000	2.484818000

Gtherm = 0.146133
E(B3LYP) = -1267.30329127
E(revDSD-PBEP86) = -1265.643357961678

***c*Im-SO_2**

6	-0.006239000	0.403047000	0.264822000
16	0.472791000	2.118394000	-0.231996000
7	-3.337726000	-0.364166000	-0.794174000
6	-3.298937000	-0.939844000	0.457091000
6	-2.085517000	-0.735935000	1.044563000
7	-1.354887000	-0.001852000	0.123685000
6	-2.170037000	0.185877000	-0.969147000
1	-4.147905000	-1.463599000	0.861937000
1	-1.673706000	-1.021790000	1.995185000
1	-1.834898000	0.722472000	-1.841625000
8	1.862504000	2.174854000	-0.647670000
7	2.861040000	-1.730105000	0.215581000
6	1.971744000	-2.430637000	-0.574088000
6	0.782999000	-1.775866000	-0.662205000
7	0.938734000	-0.630029000	0.111598000
6	2.231154000	-0.659802000	0.602640000
1	2.240619000	-3.369776000	-1.026540000
1	-0.141340000	-2.004537000	-1.158992000
1	2.615432000	0.129611000	1.222639000
8	0.271997000	1.314562000	1.282725000

Gtherm = 0.097390
E(B3LYP) = -1038.21156847
E(revDSD-PBEP86) = -1036.907190649401

Me-S

6	0.256385000	0.000000000	0.000000000
16	-1.379049000	-0.000001000	0.000000000
6	1.063126000	-1.259950000	0.000000000
1	0.443804000	-2.152690000	-0.000003000
1	1.720347000	-1.272702000	-0.875222000
1	1.720341000	-1.272705000	0.875227000
6	1.063124000	1.259950000	0.000000000
1	0.443801000	2.152690000	0.000002000
1	1.720345000	1.272704000	0.875222000
1	1.720342000	1.272707000	-0.875224000

Gtherm = 0.053477
E(B3LYP) = -516.193844264
E(revDSD-PBEP86) = -515.663264416237

Me-TS01

6	2.513063000	0.212215000	0.097991000
16	1.594639000	-1.110650000	-0.146641000
6	-2.791495000	-0.015466000	0.067516000
8	-1.941760000	0.641194000	-0.663727000
8	-2.495685000	-0.966368000	0.787560000
8	-0.362714000	-0.138125000	-0.391481000
1	-0.818154000	-0.765195000	0.225093000
6	-4.216083000	0.491067000	-0.042168000
1	-4.288871000	1.374670000	-0.670225000
1	-4.831160000	-0.307403000	-0.456940000

1	-4.583283000	0.710477000	0.959665000
6	1.951906000	1.551421000	0.447448000
1	2.547655000	2.010002000	1.240178000
1	2.042598000	2.201848000	-0.428862000
1	0.904116000	1.500901000	0.721516000
6	4.002760000	0.142982000	-0.049536000
1	4.334650000	0.901579000	-0.764297000
1	4.471018000	0.391718000	0.906926000
1	4.347567000	-0.835120000	-0.373122000

Gtherm = 0.103755
E(B3LYP) = -820.537874611
E(revDSD-PBEP86) = -819.557212277563

Me-TS01-*m*CPBA

6	4.781809000	0.545755000	0.263510000
16	4.178369000	-0.620870000	-0.700580000
6	-0.325271000	-0.887714000	-0.087336000
8	0.291388000	0.221256000	-0.383840000
8	0.241659000	-1.965962000	0.075768000
8	1.995576000	-0.169601000	-0.494626000
1	1.783285000	-1.111767000	-0.284895000
6	3.956381000	1.322035000	1.236651000
1	4.475233000	1.383899000	2.196380000
1	3.860888000	2.348519000	0.867649000
1	2.961225000	0.909293000	1.356720000
6	6.232428000	0.916007000	0.194659000
1	6.326780000	1.989609000	0.007995000
1	6.698529000	0.727858000	1.165913000
1	6.769728000	0.366230000	-0.572932000
6	-1.814984000	-0.749066000	0.047100000
6	-2.449109000	0.476847000	-0.153400000
6	-2.562014000	-1.878209000	0.379210000
6	-3.826579000	0.554192000	-0.017058000
6	-3.940180000	-1.780331000	0.510943000
6	-4.583286000	-0.563473000	0.314047000
1	-1.873084000	1.351622000	-0.412377000
1	-2.048395000	-2.816586000	0.528143000
1	-4.523069000	-2.654500000	0.768224000
1	-5.655636000	-0.477567000	0.414106000
17	-4.627856000	2.087971000	-0.266707000

Gtherm = 0.14116
E(B3LYP) = -1471.97816319
E(revDSD-PBEP86) = -1470.336302404247

Me-SO

6	-0.642582000	0.060734000	0.000000000
16	0.842320000	-0.610150000	-0.000001000
8	1.964646000	0.376740000	0.000001000
6	-0.871635000	1.529365000	0.000000000
1	0.061970000	2.088561000	-0.000002000
1	-1.458828000	1.811166000	-0.878730000
1	-1.458828000	1.811164000	0.878730000
6	-1.811731000	-0.869637000	0.000001000
1	-1.518695000	-1.920312000	-0.000011000
1	-2.432104000	-0.682439000	0.880516000
1	-2.432114000	-0.682428000	-0.880504000

Gtherm = 0.055889
E(B3LYP) = -591.437183255
E(revDSD-PBEP86) = -590.810280250803

Me-TS02

6	-1.518203000	0.723841000	0.097661000

16	-1.982949000	-0.675154000	-0.713706000
6	2.639317000	-0.129704000	-0.066221000
8	1.881481000	-0.412850000	0.950861000
8	2.207968000	0.159769000	-1.181785000
8	0.204046000	-0.270094000	0.324907000
1	0.625945000	-0.081458000	-0.558930000
6	4.121412000	-0.197013000	0.232425000
1	4.317798000	-0.364299000	1.287589000
1	4.547690000	-1.010875000	-0.354016000
1	4.582677000	0.733159000	-0.096528000
6	-1.114793000	1.894480000	-0.746605000
1	-0.879237000	1.609619000	-1.772543000
1	-1.925955000	2.626068000	-0.767500000
1	-0.236344000	2.371728000	-0.313249000
6	-1.888877000	0.932878000	1.524702000
1	-1.103644000	1.494649000	2.028736000
1	-2.815166000	1.511378000	1.576217000
1	-2.043277000	-0.016163000	2.032326000
8	-2.640550000	-1.654104000	0.169194000

Gtherm = 0.109115
E(B3LYP) = -895.772375412
E(revDSD-PBEP86) = -894.696384766413

Me-TS02-*m*CPBA

6	-3.754688000	-0.122695000	0.730357000
16	-4.258786000	0.125585000	-0.853075000
6	0.349668000	0.658466000	-0.149593000
8	-0.177450000	-0.532027000	-0.153688000
8	-0.317627000	1.694852000	-0.180036000
8	-1.939580000	-0.257450000	-0.174072000
1	-1.722410000	0.713415000	-0.239253000
6	-3.596775000	1.091762000	1.592771000
1	-3.559392000	2.015284000	1.014058000
1	-4.435004000	1.154396000	2.290851000
1	-2.677411000	1.012242000	2.172410000
6	-3.833788000	-1.471745000	1.353914000
1	-2.993574000	-1.611740000	2.032861000
1	-4.759046000	-1.548711000	1.931526000
1	-3.829126000	-2.254350000	0.599075000
8	-4.655062000	-1.120760000	-1.528594000
6	1.847469000	0.681445000	-0.105185000
6	2.496244000	1.915223000	-0.134528000
6	2.584996000	-0.500426000	-0.035823000
6	3.882465000	1.964962000	-0.093868000
6	3.968903000	-0.429204000	0.005465000
6	4.628651000	0.794046000	-0.023091000
1	1.903560000	2.816338000	-0.189607000
1	2.084101000	-1.455943000	-0.015847000
1	4.390851000	2.919346000	-0.117424000
1	5.708183000	0.823624000	0.009126000
17	4.900514000	-1.903599000	0.094708000

Gtherm = 0.146565
E(B3LYP) = -1547.21188391
E(revDSD-PBEP86) = -1545.474854100487

***c*Me-SO_2**

6	0.731618000	0.077495000	0.020931000
6	1.900804000	-0.758618000	-0.421046000
16	-0.952182000	-0.547258000	-0.209084000
8	0.030518000	-0.450812000	1.163168000
6	0.897647000	1.570970000	0.016894000
1	1.302951000	1.902232000	-0.939393000
1	-0.051548000	2.072247000	0.195069000

1	1.596876000	1.856557000	0.805980000
1	1.637849000	-1.814564000	-0.471747000
1	2.261023000	-0.432746000	-1.396693000
8	-1.956501000	0.511121000	-0.269202000
1	2.715206000	-0.649154000	0.299715000

Gtherm = 0.059461
E(B3LYP) = -666.679282235
E(revDSD-PBEP86) = -665.956776178000

***f*Me-TS02**

6	2.301081000	0.570938000	-0.075968000
16	1.345014000	-0.741529000	-0.346440000
6	-2.911883000	0.053498000	0.009076000
8	-2.077356000	0.524038000	0.873804000
8	-2.581823000	-0.590350000	-0.990921000
8	-0.425343000	-0.036739000	0.203112000
1	-0.991482000	-0.452567000	-0.518113000
6	-4.367911000	0.346107000	0.317328000
1	-4.820832000	0.801110000	-0.562382000
1	-4.869533000	-0.603288000	0.504108000
1	-4.479312000	0.996742000	1.179817000
6	1.860610000	1.882596000	-0.624087000
1	0.932868000	1.812320000	-1.187034000
1	1.692701000	2.573058000	0.208991000
1	2.640030000	2.317994000	-1.254399000
6	3.594319000	0.442681000	0.642902000
1	4.426706000	0.723567000	-0.008637000
1	3.604231000	1.143198000	1.484356000
1	3.754085000	-0.566171000	1.016131000
8	1.801150000	-1.979001000	0.297092000

Gtherm = 0.106854
E(B3LYP) = -895.764487820
E(revDSD-PBEP86) = -894.686556809606

***f*Me-SO$_2$**

6	-0.853732000	0.000000000	0.000000000
6	-1.602710000	1.299267000	0.000000000
16	0.746991000	0.000000000	0.000000000
8	1.454692000	1.259523000	0.000000000
6	-1.602710000	-1.299267000	0.000000000
1	-2.247314000	-1.355394000	-0.880990000
1	-0.941378000	-2.161307000	0.000000000
1	-2.247314000	-1.355394000	0.880990000
1	-0.941378000	2.161307000	-0.000001000
1	-2.247315000	1.355393000	-0.880989000
8	1.454692000	-1.259523000	0.000000000
1	-2.247314000	1.355394000	0.880990000

Gtherm = 0.059402
E(B3LYP) = -666.664379715
E(revDSD-PBEP86) = -665.941526832927

Me-SO$_2$-TSiso

6	-0.875043000	0.080014000	-0.037906000
6	-1.866159000	-0.958948000	-0.316838000
16	0.860354000	-0.269224000	-0.347556000
8	1.037811000	-1.128579000	0.875987000
6	-1.233519000	1.412378000	0.421463000
1	-2.205122000	1.462727000	0.910887000
1	-0.411207000	1.820852000	1.023313000
1	-1.255496000	2.064312000	-0.463303000
1	-2.365334000	-1.193927000	0.631915000
1	-2.655601000	-0.584261000	-0.978074000
8	1.512006000	1.053891000	-0.281096000

1	-1.423105000	-1.865278000	-0.723296000

Gtherm = 0.054781
E(B3LYP) = -666.613430759
E(revDSD-PBEP86) = -665.899441424639

Ph-S

16	0.000031000	2.581723000	0.000119000
6	0.000043000	0.937629000	-0.000504000
6	1.265676000	0.169048000	0.031649000
6	1.357146000	-1.017464000	0.773589000
6	2.408356000	0.629615000	-0.635642000
6	2.557515000	-1.709226000	0.859563000
6	3.598297000	-0.077491000	-0.570867000
6	3.679648000	-1.246198000	0.181964000
1	0.489309000	-1.383305000	1.303640000
1	2.344787000	1.544031000	-1.207954000
1	2.615296000	-2.610793000	1.454797000
1	4.466219000	0.283471000	-1.106475000
1	4.612146000	-1.792006000	0.238664000
6	-1.265621000	0.169084000	-0.032133000
6	-2.407995000	0.629695000	0.635651000
6	-1.357464000	-1.017406000	-0.774063000
6	-3.598003000	-0.077341000	0.571356000
6	-2.557910000	-1.709094000	-0.859554000
6	-3.679733000	-1.246024000	-0.181470000
1	-2.344137000	1.544100000	1.207951000
1	-0.489876000	-1.383277000	-1.304498000
1	-4.465682000	0.283659000	1.107333000
1	-2.615993000	-2.610640000	-1.454792000
1	-4.612286000	-1.791778000	-0.237795000

Gtherm = 0.150758
E(B3LYP) = -899.822797265
E(revDSD-PBEP86) = -898.604682040941

Ph-TS01

6	-4.063492000	-0.823210000	0.139612000
8	-3.296719000	-1.375947000	1.029201000
8	-3.730823000	-0.593293000	-1.022132000
8	-1.729273000	-1.619912000	0.247150000
1	-2.137153000	-1.328252000	-0.599908000
6	-5.437328000	-0.456066000	0.664588000
1	-5.627948000	-0.886968000	1.643720000
1	-5.496211000	0.630917000	0.730448000
1	-6.185775000	-0.791866000	-0.051246000
16	0.262284000	-1.850766000	-0.659183000
6	0.986080000	-0.409753000	-0.299093000
6	0.220418000	0.837961000	-0.182900000
6	0.559992000	1.774516000	0.807015000
6	-0.885128000	1.096969000	-1.004269000
6	-0.198570000	2.921530000	0.980023000
6	-1.629308000	2.252066000	-0.840840000
6	-1.292244000	3.164710000	0.154762000
1	1.399164000	1.581375000	1.459770000
1	-1.157014000	0.391036000	-1.774315000
1	0.059539000	3.622775000	1.762038000
1	-2.482479000	2.428175000	-1.480743000
1	-1.881889000	4.061980000	0.288843000
6	2.443037000	-0.368852000	-0.077713000
6	3.112331000	-1.416833000	0.571346000
6	3.192914000	0.718762000	-0.551724000
6	4.486159000	-1.373569000	0.744446000
6	4.570814000	0.745855000	-0.397341000
6	5.220666000	-0.296539000	0.255311000

1 2.540216000 -2.250152000 0.953321000
1 2.691390000 1.528577000 -1.061920000
1 4.986259000 -2.179759000 1.263928000
1 5.137539000 1.581336000 -0.785639000
1 6.294300000 -0.268974000 0.385544000
Gtherm = 0.203248
E(B3LYP) = -1204.16833836
E(revDSD-PBEP86) = -1202.498255290297

Ph-SO
16 0.056154000 2.312145000 0.082994000
6 -0.020880000 0.663387000 0.016512000
6 1.323146000 0.033676000 0.026922000
6 1.635477000 -0.918865000 1.006870000
6 2.303129000 0.381637000 -0.907826000
6 2.894876000 -1.494732000 1.054443000
6 3.567660000 -0.195618000 -0.855161000
6 3.867159000 -1.132838000 0.124213000
1 0.883236000 -1.196469000 1.732696000
1 2.063698000 1.090932000 -1.687802000
1 3.122260000 -2.223940000 1.820657000
1 4.313246000 0.082529000 -1.587977000
1 4.849162000 -1.585052000 0.162121000
6 -1.231369000 -0.156480000 -0.037105000
6 -2.482645000 0.320833000 0.392983000
6 -1.156687000 -1.465230000 -0.546948000
6 -3.604174000 -0.491677000 0.325484000
6 -2.285452000 -2.265044000 -0.622900000
6 -3.514445000 -1.783330000 -0.183094000
1 -2.570272000 1.325527000 0.772975000
1 -0.207713000 -1.849464000 -0.891890000
1 -4.556601000 -0.110432000 0.668961000
1 -2.206167000 -3.265910000 -1.026027000
1 -4.395378000 -2.409009000 -0.238841000
8 -1.246087000 3.021582000 0.022607000
Gtherm = 0.152745
E(B3LYP) = -975.064677080
E(revDSD-PBEP86) = -973.748770351569

Ph-TS02
6 3.119719000 -1.729988000 -0.653192000
8 2.174139000 -2.481557000 -0.193403000
8 3.161823000 -0.505241000 -0.514810000
8 1.010160000 -1.285714000 0.620409000
1 1.687775000 -0.600969000 0.357508000
6 4.208147000 -2.482051000 -1.395299000
1 3.999457000 -3.546313000 -1.457556000
1 4.298806000 -2.057877000 -2.394712000
1 5.150504000 -2.318695000 -0.872823000
16 -0.100092000 -0.288985000 2.118254000
6 -0.749338000 0.355311000 0.680270000
6 -0.081225000 1.581937000 0.230846000
6 0.319416000 1.697708000 -1.109881000
6 0.212645000 2.627470000 1.118176000
6 1.002210000 2.820044000 -1.542993000
6 0.890743000 3.754869000 0.674903000
6 1.289563000 3.852401000 -0.652517000
1 0.121485000 0.882058000 -1.790415000
1 -0.115784000 2.566221000 2.146238000
1 1.325302000 2.886238000 -2.572837000
1 1.103051000 4.558257000 1.367106000
1 1.823726000 4.728676000 -0.994433000
6 -1.855275000 -0.231548000 -0.052874000

6	-2.134509000	-1.610447000	-0.009006000
6	-2.686760000	0.602907000	-0.824025000
6	-3.202780000	-2.125444000	-0.722359000
6	-3.764577000	0.079422000	-1.518035000
6	-4.023909000	-1.286947000	-1.472396000
1	-1.497664000	-2.267074000	0.559734000
1	-2.490024000	1.664668000	-0.859320000
1	-3.396546000	-3.189106000	-0.694970000
1	-4.401262000	0.735774000	-2.095729000
1	-4.861722000	-1.697592000	-2.020208000
8	-0.897377000	-1.371783000	2.702883000

Gtherm = 0.204473
E(B3LYP) = -1279.39849919
E(revDSD-PBEP86) = -1277.633355349923

***c*Ph-SO_2**

16	-0.084354000	2.254834000	-0.491180000
6	0.084351000	0.546611000	0.202425000
6	-1.183538000	-0.237080000	0.155237000
6	-1.659315000	-0.745169000	-1.052651000
6	-1.906731000	-0.454715000	1.325672000
6	-2.832568000	-1.487173000	-1.085573000
6	-3.078740000	-1.199397000	1.291842000
6	-3.541837000	-1.719531000	0.087967000
1	-1.117095000	-0.545763000	-1.967680000
1	-1.551384000	-0.032246000	2.255133000
1	-3.198979000	-1.872364000	-2.027627000
1	-3.634054000	-1.368041000	2.204804000
1	-4.458083000	-2.294161000	0.062328000
6	1.401663000	-0.141757000	0.105163000
6	2.565256000	0.521241000	0.512322000
6	1.509930000	-1.440945000	-0.401290000
6	3.803952000	-0.096257000	0.402747000
6	2.751136000	-2.052915000	-0.510643000
6	3.903187000	-1.383477000	-0.112671000
1	2.494253000	1.513762000	0.932095000
1	0.623106000	-1.980008000	-0.699121000
1	4.692738000	0.429027000	0.726174000
1	2.816392000	-3.059519000	-0.901767000
1	4.868680000	-1.864180000	-0.195962000
8	0.063046000	1.673317000	1.088859000
8	-1.448845000	2.619125000	-0.845458000

Gtherm = 0.155385
E(B3LYP) = -1050.30099561
E(revDSD-PBEP86) = -1048.890250845521

H-S

6	0.000000000	0.000000000	-1.022340000
16	0.000000000	0.000000000	0.583839000
1	0.000000000	0.920194000	-1.603692000
1	0.000000000	-0.920194000	-1.603692000

Gtherm = 0.002330
E(B3LYP) = -437.511257820
E(revDSD-PBEP86) = -437.126062869729

H-TS01

6	-2.015623000	0.064625000	0.040091000
8	-1.225385000	-0.915641000	-0.269181000
8	-1.628849000	1.196755000	0.331560000
8	0.435376000	-0.220979000	-0.171040000
1	-0.006998000	0.630033000	0.095573000
6	-3.488194000	-0.291835000	0.014930000
1	-3.981119000	0.342362000	-0.721561000

1 -3.650755000 -1.337741000 -0.229817000
1 -3.914331000 -0.064829000 0.991539000
6 3.087558000 -0.781916000 0.419369000
16 2.423559000 0.531070000 -0.210787000
1 4.149213000 -0.982353000 0.317338000
1 2.475475000 -1.510910000 0.942458000
Gtherm = 0.052829
E(B3LYP) = -741.851396817
E(revDSD-PBEP86) = -741.017663322960

H-SO
6 1.327757000 0.335409000 -0.000050000
16 -0.089612000 -0.423409000 -0.000013000
1 2.204196000 -0.298894000 -0.000128000
8 -1.270368000 0.456042000 0.000081000
1 1.425996000 1.412643000 -0.000010000
Gtherm = 0.003801
E(B3LYP) = -512.758580900
E(revDSD-PBEP86) = -512.278018179372

H-TS02
6 -2.338670000 0.064921000 -0.093398000
8 -1.614090000 -0.948652000 0.283685000
8 -1.878931000 1.176410000 -0.345188000
8 0.055433000 -0.320971000 0.317975000
1 -0.329490000 0.538487000 -0.015626000
6 -3.814089000 -0.249743000 -0.206430000
1 -4.041667000 -1.263384000 0.110333000
1 -4.363883000 0.467837000 0.401287000
1 -4.110438000 -0.110877000 -1.245785000
6 1.628590000 0.359878000 1.134977000
16 2.444762000 0.383891000 -0.311626000
1 1.282522000 1.319374000 1.497207000
1 1.795501000 -0.437556000 1.844645000
8 3.162121000 -0.870097000 -0.583588000
Gtherm = 0.05668
E(B3LYP) = -817.089518546
E(revDSD-PBEP86) = -816.161893706246

***c*H-SO_2**
6 -1.163193000 0.647507000 0.013092000
16 0.376033000 -0.157897000 0.425724000
1 -1.935273000 0.795859000 0.761830000
8 1.473322000 0.257270000 -0.435940000
1 -1.075019000 1.400362000 -0.764193000
8 -0.976707000 -0.701635000 -0.425032000
Gtherm = 0.007855
E(B3LYP) = -587.996832319
E(revDSD-PBEP86) = -587.421920647530

CN-S
6 0.000000000 0.000000000 0.051174000
16 0.000000000 0.000000000 1.676751000
6 0.000000000 1.209212000 -0.716481000
7 0.000000000 2.188806000 -1.324092000
6 0.000000000 -1.209212000 -0.716481000
7 0.000000000 -2.188806000 -1.324092000
Gtherm = -0.005900
E(B3LYP) = -622.047531349
E(revDSD-PBEP86) = -621.372281915607

CN-TS01
6 3.059575000 -0.028640000 -0.101007000

8	2.039525000	-0.351156000	-0.863407000
8	2.936939000	0.402505000	1.036217000
8	0.643750000	0.002456000	0.108097000
1	1.241034000	0.328038000	0.846988000
6	4.390101000	-0.236640000	-0.783162000
1	4.874003000	0.735304000	-0.875307000
1	5.003859000	-0.865776000	-0.139811000
1	4.283467000	-0.690967000	-1.763864000
6	-2.175098000	0.031404000	0.131879000
16	-0.970094000	-0.617201000	1.035963000
6	-2.226112000	1.422699000	-0.165525000
7	-2.263122000	2.551228000	-0.399660000
6	-3.191530000	-0.794551000	-0.426902000
7	-4.020335000	-1.469438000	-0.860677000

Gtherm = 0.045464
E(B3LYP) = -926.385155243
E(revDSD-PBEP86) = -925.259082984220

CN-SO

6	-0.362106000	0.100335000	0.000023000
16	0.865954000	-1.020374000	-0.000023000
8	2.192339000	-0.409048000	-0.000125000
6	-1.687148000	-0.404712000	0.000117000
7	-2.755312000	-0.839447000	0.000194000
6	-0.110318000	1.491907000	-0.000037000
7	0.121520000	2.621331000	-0.000087000

Gtherm = -0.004241
E(B3LYP) = -697.294619173
E(revDSD-PBEP86) = -696.523977373738

CN-TS02

6	-2.673303000	0.050459000	0.361238000
8	-2.147908000	1.087811000	-0.219827000
8	-1.980277000	-0.852907000	0.837921000
8	-0.378533000	0.712003000	-0.050936000
1	-0.732586000	-0.173697000	0.414354000
6	-4.178056000	0.043424000	0.405803000
1	-4.524410000	-0.858808000	-0.097413000
1	-4.483875000	-0.008180000	1.450176000
1	-4.602484000	0.923954000	-0.066795000
6	1.801673000	-0.018188000	0.160744000
16	0.975705000	0.265408000	-1.331261000
8	0.596444000	-0.985130000	-1.956990000
6	1.873484000	-1.317525000	0.696427000
7	1.929954000	-2.394900000	1.108114000
6	2.448219000	1.076653000	0.767376000
7	2.981785000	1.990193000	1.230312000

Gtherm = 0.047161
E(B3LYP) = -1001.61830750
E(revDSD-PBEP86) = -1000.400618913169

***c*CN-SO$_2$**

6	0.494137000	0.079490000	0.167634000
16	-1.100429000	-0.847157000	-0.216198000
8	-2.233038000	0.020534000	-0.415735000
8	-0.264336000	-0.525538000	1.211248000
6	1.762099000	-0.527331000	-0.131924000
7	2.755591000	-1.043086000	-0.398547000
6	0.409760000	1.507831000	0.044571000
7	0.328677000	2.648029000	-0.085256000

Gtherm = -0.001482
E(B3LYP) = -772.522580041
E(revDSD-PBEP86) = -771.656196981702

CF_3-S

6	0.000003000	0.503347000	-0.000009000
16	0.000011000	2.100219000	-0.000004000
6	1.291200000	-0.335287000	-0.003482000
9	1.165814000	-1.408772000	-0.800542000
9	2.343826000	0.365479000	-0.421245000
9	1.546327000	-0.767845000	1.243979000
6	-1.291201000	-0.335283000	0.003477000
9	-2.343822000	0.365513000	0.421217000
9	-1.546335000	-0.767876000	-1.243964000
9	-1.165830000	-1.408740000	0.800571000

Gtherm = -0.000189
E(B3LYP) = -1111.88863801
E(revDSD-PBEP86) = -1110.684872797690

CF_3-TS01

6	-3.995131000	-0.102892000	0.106877000
8	-2.977554000	-0.532597000	0.807544000
8	-3.881705000	0.456191000	-0.977277000
8	-1.571419000	-0.088127000	-0.164216000
1	-2.191611000	0.324016000	-0.838466000
6	-5.327094000	-0.356760000	0.774201000
1	-5.223294000	-0.916403000	1.699176000
1	-5.791351000	0.608479000	0.974306000
1	-5.958124000	-0.900787000	0.072564000
6	1.258501000	-0.076122000	-0.341281000
16	-0.004302000	-0.616071000	-1.181189000
6	2.333103000	-1.039666000	0.156387000
9	2.404146000	-2.140784000	-0.602845000
9	3.544204000	-0.463981000	0.160269000
9	2.060719000	-1.430313000	1.415279000
6	1.425962000	1.376942000	0.093898000
9	0.344712000	2.115148000	-0.161898000
9	1.685969000	1.455980000	1.409788000
9	2.460982000	1.936070000	-0.562534000

Gtherm = 0.051719
E(B3LYP) = -1416.22311065
E(revDSD-PBEP86) = -1414.568260706116

CF_3-SO

6	-0.008648000	0.222351000	0.000009000
16	-0.716778000	1.691384000	0.000003000
8	-2.178202000	1.636998000	-0.000005000
6	1.497990000	0.156796000	-0.000001000
9	1.959765000	-0.486222000	-1.084203000
9	2.038898000	1.391698000	-0.000005000
9	1.959773000	-0.486213000	1.084204000
6	-0.810581000	-1.065635000	0.000001000
9	-1.596410000	-1.141736000	1.084763000
9	-1.596354000	-1.141756000	-1.084797000
9	-0.007727000	-2.140129000	0.000030000

Gtherm = 0.002068
E(B3LYP) = -1187.13037981
E(revDSD-PBEP86) = -1185.830046114763

CF_3-TS02

6	3.235960000	-0.119739000	-0.137422000
8	2.412277000	-1.132957000	-0.186932000
8	2.886028000	1.038326000	-0.334915000
8	0.846274000	-0.394605000	-0.575138000
1	1.326814000	0.495048000	-0.557562000
6	4.657303000	-0.512672000	0.190137000

1	4.927794000	-0.036066000	1.132117000
1	4.776480000	-1.589027000	0.272243000
1	5.306441000	-0.118895000	-0.590661000
6	-0.881478000	0.053401000	-0.160429000
16	-1.146486000	1.005788000	-1.543074000
8	-0.962622000	2.436463000	-1.273420000
6	-0.807184000	0.711519000	1.220698000
9	0.145276000	1.644215000	1.256819000
9	-1.981228000	1.292253000	1.515588000
9	-0.543478000	-0.191733000	2.171642000
6	-1.499262000	-1.346089000	-0.215367000
9	-1.631839000	-1.750731000	-1.490644000
9	-0.782025000	-2.254638000	0.434510000
9	-2.726866000	-1.310478000	0.335483000

Gtherm = 0.055756
E(B3LYP) = -1491.45595808
E(revDSD-PBEP86) = -1489.707536230607

cCF$_3$-SO$_2$

6	0.011849000	-0.154384000	0.186910000
16	-1.207663000	-1.467386000	-0.144978000
8	-2.537821000	-0.971498000	-0.409970000
8	-0.506153000	-0.857913000	1.287005000
6	1.494691000	-0.416103000	-0.081716000
9	1.898109000	0.219661000	-1.187659000
9	2.248626000	-0.010264000	0.946492000
9	1.709681000	-1.732491000	-0.250508000
6	-0.442945000	1.306148000	0.019982000
9	-0.629841000	1.583327000	-1.279193000
9	-1.575974000	1.535732000	0.675331000
9	0.493046000	2.148425000	0.490238000

Gtherm = 0.005904
E(B3LYP) = -1262.37289642
E(revDSD-PBEP86) = -1260.975624284529

F-S

6	0.000000000	0.000000000	-0.286026000
16	0.000000000	0.000000000	1.308739000
9	0.000000000	1.059545000	-1.067982000
9	0.000000000	-1.059545000	-1.067982000

Gtherm = -0.015017
E(B3LYP) = -636.093941491
E(revDSD-PBEP86) = -635.481158641885

F-TS01

6	-2.625866000	0.060898000	0.059111000
8	-1.969178000	0.046681000	-1.043499000
8	-2.123163000	-0.208175000	1.162450000
8	-0.234104000	-0.451701000	-0.453069000
1	-0.713954000	-0.457880000	0.442951000
6	-4.090024000	0.433334000	-0.055648000
1	-4.686293000	-0.394035000	0.328257000
1	-4.275785000	1.301456000	0.576054000
1	-4.372272000	0.650666000	-1.081756000
6	2.376524000	0.292305000	0.063221000
16	1.558660000	-1.103365000	0.086187000
9	3.659821000	0.387613000	-0.162175000
9	1.868790000	1.472383000	0.231992000

Gtherm = 0.035271
E(B3LYP) = -940.418705016
E(revDSD-PBEP86) = -939.356189687406

F-SO

6	0.678711000	0.030191000	-0.000015000
16	-0.859288000	-0.564286000	0.000008000
9	1.724852000	-0.767341000	-0.000075000
8	-1.868398000	0.516473000	0.000065000
9	1.011096000	1.291302000	0.000013000

Gtherm = -0.014035
E(B3LYP) = -711.303104126
E(revDSD-PBEP86) = -710.594423940984

F-TS02

6	2.627908000	-0.231657000	0.041529000
8	1.841450000	0.192011000	-0.924478000
8	2.212873000	-0.722284000	1.080096000
8	0.248178000	-0.101459000	-0.384529000
1	0.596297000	-0.506509000	0.458184000
6	4.092759000	-0.054404000	-0.276743000
1	4.249152000	0.443989000	-1.229034000
1	4.547536000	0.521128000	0.528534000
1	4.555501000	-1.040690000	-0.293650000
6	-1.378699000	0.712281000	0.159641000
16	-2.152662000	-0.794936000	0.408126000
9	-0.994676000	1.408294000	1.207111000
9	-1.606523000	1.451497000	-0.882028000
8	-2.820866000	-1.242565000	-0.829383000

Gtherm = 0.038827
E(B3LYP) = -1015.63951851
E(revDSD-PBEP86) = -1014.480954781719

***c*F-SO$_2$**

6	0.737311000	0.040826000	0.020289000
16	-0.982341000	-0.484561000	-0.225053000
9	1.769455000	-0.607746000	-0.526748000
8	-1.917691000	0.620063000	-0.158079000
9	1.010891000	1.342436000	0.058370000
8	0.201501000	-0.508088000	1.119892000

Gtherm = -0.010614
E(B3LYP) = -786.567537326
E(revDSD-PBEP86) = -785.764062492572

Cl-S

6	0.000000000	0.000000000	0.143316000
16	0.000000000	0.000000000	1.743051000
17	0.000000000	1.433574000	-0.845550000
17	0.000000000	-1.433574000	-0.845550000

Gtherm = -0.020340
E(B3LYP) = -1356.77056854
E(revDSD-PBEP86) = -1355.752573164339

Cl-TS01

6	-3.124400000	0.042301000	0.005904000
8	-2.490665000	-0.558187000	-0.946184000
8	-2.631624000	0.294564000	1.109877000
8	-0.827942000	-0.866618000	-0.221953000
1	-1.206348000	-0.434345000	0.599869000
6	-4.552172000	0.423435000	-0.328772000
1	-5.214653000	-0.093863000	0.364899000
1	-4.669052000	1.494777000	-0.169185000
1	-4.813269000	0.166116000	-1.351344000
6	1.893443000	0.013329000	0.086370000
16	1.035211000	-1.318251000	0.428209000
17	3.565472000	-0.054831000	-0.331237000
17	1.236914000	1.591706000	0.071794000

Gtherm = 0.029157

E(B3LYP) = -1661.10240963
E(revDSD-PBEP86) = -1659.634283389971

Cl-SO

6	0.268892000	0.001056000	-0.000505000
16	-1.118625000	-0.890384000	-0.000275000
17	1.770453000	-0.839548000	0.000274000
8	-2.333837000	-0.056475000	0.000336000
17	0.285744000	1.703760000	0.000004000

Gtherm = -0.018805
E(B3LYP) = -1431.99953540
E(revDSD-PBEP86) = -1430.886936092573

Cl-TS02

6	-2.999818000	0.105022000	0.125355000
8	-2.330898000	-0.845941000	0.685407000
8	-2.491482000	0.954804000	-0.614667000
8	-0.582911000	-0.515453000	0.090977000
1	-1.040237000	0.256705000	-0.382009000
6	-4.480134000	0.110761000	0.443901000
1	-5.029309000	0.027645000	-0.493538000
1	-4.755676000	-0.700926000	1.110756000
1	-4.727792000	1.069781000	0.897650000
6	1.630257000	0.306007000	-0.008423000
16	1.029835000	-0.882027000	-1.074186000
17	1.479788000	1.943882000	-0.454551000
17	2.401205000	-0.101561000	1.433038000
8	1.429910000	-2.217280000	-0.654864000

Gtherm = 0.033031
E(B3LYP) = -1736.32515674
E(revDSD-PBEP86) = -1734.764832002985

***c*Cl-SO$_2$**

6	0.351837000	-0.012830000	0.149295000
16	-1.282583000	-0.761603000	-0.189804000
17	1.832699000	-0.881908000	-0.254018000
8	-2.332139000	0.216470000	-0.384294000
17	0.501185000	1.723324000	-0.025396000
8	-0.326076000	-0.471650000	1.245686000

Gtherm = -0.015939
E(B3LYP) = -1507.24293144
E(revDSD-PBEP86) = -1506.034856979468

Br-S

6	0.000000000	0.000000000	0.549867000
16	0.000000000	0.000000000	2.142489000
35	0.000000000	1.582677000	-0.536843000
35	0.000000000	-1.582677000	-0.536843000

Gtherm = -0.023742
E(B3LYP) = -5584.67985633
E(revDSD-PBEP86) = -5582.822391713985

Br-TS01

6	3.775293000	0.137333000	-0.044789000
8	3.194572000	0.962963000	-0.852076000
8	3.246993000	-0.315879000	0.974765000
8	1.538924000	1.203185000	-0.103902000
1	1.870539000	0.591168000	0.617022000
6	5.186086000	-0.244501000	-0.443726000
1	5.860730000	0.056365000	0.357377000
1	5.235695000	-1.329032000	-0.535568000
1	5.486293000	0.223626000	-1.376878000
6	-1.202459000	0.245725000	0.173604000

16	-0.347860000	1.558730000	0.558200000
35	-3.057721000	0.337374000	-0.232703000
35	-0.464705000	-1.483525000	0.054018000

Gtherm = 0.026520
E(B3LYP) = -5889.01245072
E(revDSD-PBEP86) = -5886.704699411491

Br-SO

6	0.000344000	0.230122000	0.000065000
16	0.496746000	1.797069000	0.000211000
35	-1.862664000	-0.088234000	-0.000118000
8	1.962080000	1.946484000	-0.000108000
35	1.187046000	-1.217644000	0.000035000

Gtherm = -0.022168
E(B3LYP) = -5659.91101132
E(revDSD-PBEP86) = -5657.959726387473

Br-TS02

6	3.533426000	-0.023765000	-0.325385000
8	2.890204000	-1.138679000	-0.424557000
8	3.031197000	1.021695000	0.100122000
8	1.176630000	-0.672820000	0.183065000
1	1.609994000	0.236099000	0.292431000
6	4.977000000	-0.086912000	-0.779727000
1	5.255059000	-1.081669000	-1.115290000
1	5.111271000	0.632068000	-1.587243000
1	5.608580000	0.217540000	0.054287000
6	-1.111987000	0.054521000	0.256250000
16	-0.285941000	-0.675308000	1.551083000
35	-1.040252000	1.921139000	0.121076000
35	-2.082851000	-0.944698000	-0.965364000
8	-0.609516000	-2.089900000	1.664089000

Gtherm = 0.02962
E(B3LYP) = -5964.23613107
E(revDSD-PBEP86) = -5961.837819978854

***c*Br-SO_2**

6	0.004307000	-0.200161000	0.220140000
16	-0.959710000	-1.695825000	-0.189711000
35	1.918064000	-0.160286000	-0.121475000
8	-2.356321000	-1.428611000	-0.459596000
35	-0.852690000	1.512750000	-0.019619000
8	-0.388499000	-0.946647000	1.291200000

Gtherm = -0.019410
E(B3LYP) = -5735.15240198
E(revDSD-PBEP86) = -5733.104485770041

REFERENCES

[1] M. K. Denk, S. Gupta, J. Brownie, S. Tajammul, A. J. Lough, *Chem. Eur. J.* **2001**, *7*, 4477-4486.

[2] Y.-Q. Li, J. Zhou, R. Xiao, Z.-G. Cai, *Chin. J. Polym. Sci.* **2020**, *38*, 941-949.

[3] D. W. Karkhanis, L. Field, *Phosphorus, Sulfur Relat. Elem.* **1985**, *22*, 49-57.

[4] A. J. Arduengo, R. Krafczyk, R. Schmutzler, H. A. Craig, J. R. Goerlich, W. J. Marshall, M. Unverzagt, *Tetrahedron* **1999**, *55*, 14523-14534.

[5] R. Kia, V. Mirkhani, A. Kálmán, A. Deák, *Polyhedron* **2007**, *26*, 2906-2910.

[6] Y. A. Ibrahim, N. A. Al-Awadi, T. F. Al-Azemi, E. John, *RSC Adv.* **2014**, *4*, 38869-38876.

[7] D. Yang, Y.-C. Chen, N.-Y. Zhu, *Org. Lett.* **2004**, *6*, 1577-1580.

[8] (a) V. N. Valaeva, A. F. Asachenko, P. S. Kulyabin, V. R. Flid, A. Z. Voskoboinikov, *Russ. J. Org. Chem* **2011**, *47*, 1774-1776; (b) R. Chen, K. Tatsumi, *J. Coord. Chem* **2002**, *55*, 1219-1222.

[9] J. E. Thomson, C. D. Campbell, C. Concellón, N. Duguet, K. Rix, A. M. Z. Slawin, A. D. Smith, *J. Org. Chem.* **2008**, *73*, 2784-2791.

[10] M. Paas, B. Wibbeling, R. Fröhlich, F. E. Hahn, *Eur. J. Inorg. Chem.* **2006**, 158-162.

[11] B. Benac, E. Burgess, A. Arduengo, D. Brittelli, *Org. Synth.* **1986**, *64*, 92.

[12] L. Carroccia, M. Delfini, S. Fioravanti, L. Pellacani, F. Sciubba, *J. Org. Chem.* **2012**, *77*, 2069-2073.

[13] M. Haaf, A. Schmiedl, T. A. Schmedake, D. R. Powell, A. J. Millevolte, M. Denk, R. West, *J. Am. Chem. Soc.* **1998**, *120*, 12714-12719.

[14] E. A. Mistryukov, *Mendeleev Commun.* **2006**, *16*, 258-259.

[15] C. J. Serpell, J. Cookson, A. L. Thompson, C. M. Brown, P. D. Beer, *Dalton Trans.* **2013**, *42*, 1385-1393.

[16] M. Ruamps, S. Bastin, L. Rechignat, A. Sournia-Saquet, D. A. Valyaev, J.-M. Mouesca, N. Lugan, V. Maurel, V. César, *Chem. Commun.* **2018**, *54*, 7653-7656.

[17] L. Hintermann, *Beilstein J. Org. Chem.* **2007**, *3*, 22.

[18] M. Tretiakov, Y. G. Shermolovich, A. P. Singh, P. P. Samuel, H. W. Roesky, B. Niepötter, A. Visscher, D. Stalke, *Dalton Trans.* **2013**, *42*, 12940-12946.

[19] L. E. Longobardi, V. Wolter, D. W. Stephan, *Angew. Chem., Int. Ed. Engl.* **2015**, *54*, 809-812.

[20] G. Sheldrick, *Acta Crystallogr., Sect. A: Found. Adv.* **2015**, *71*, 3-8.

[21] O. V. Dolomanov, L. J. Bourhis, R. J. Gildea, J. A. K. Howard, H. Puschmann, *J. Appl. Crystallogr.* **2009**, *42*, 339-341.

[22] L. J. Bourhis, O. V. Dolomanov, R. J. Gildea, J. A. K. Howard, H. Puschmann, *Acta Crystallogr., Sect. A: Found. Adv.* **2015**, *71*, 59-75.

[23] R. C. Clark, J. S. Reid, *Acta Crystallogr., Sect. A: Found. Crystallogr.* **1995**, *51*, 887-897.

[24] F. Kleemiss, O. V. Dolomanov, M. Bodensteiner, N. Peyerimhoff, L. Midgley, L. J. Bourhis, A. Genoni, L. A. Malaspina, D. Jayatilaka, J. L. Spencer, F. White, B. Grundkötter-Stock, S. Steinhauer, D. Lentz, H. Puschmann, S. Grabowsky, *Chem. Sci.* **2021**, *12*, 1675-1692.

[25] (a) A. D. Becke, *J. Chem. Phys.* **1993**, *98*, 5648-5652; (b) P. J. Stephens, F. J. Devlin, C. F. Chabalowski, M. J. Frisch, *J. Phys. Chem.* **1994**, *98*, 11623-11627.

[26] S. Grimme, J. Antony, S. Ehrlich, H. Krieg, *J. Chem. Phys.* **2010**, *132*, 154104.

[27] F. Weigend, R. Ahlrichs, *Phys. Chem. Chem. Phys.* **2005**, *7*, 3297-3305.

[28] (a) E. D. Glendening, C. R. Landis, F. Weinhold, *Wiley Interdiscip. Rev.: Comput. Mol. Sci.* **2012**, *2*, 1-42; (b) E. D. Glendening, C. R. Landis, F. Weinhold, *J. Comput. Chem.* **2013**, *34*, 1429-1437.

[29] K. Fukui, *Acc. Chem. Res.* **1981**, *14*, 363-368.

[30] G. Santra, N. Sylvetsky, J. M. Martin, *J. Phys. Chem. A* **2019**, *123*, 5129-5143.

[31] (a) E. Caldeweyher, C. Bannwarth, S. Grimme, *J. Chem. Phys.* **2017**, *147*, 034112; (b) E. Caldeweyher, S. Ehlert, A. Hansen, H. Neugebauer, S. Spicher, C. Bannwarth, S. Grimme, *J. Chem. Phys.* **2019**, *150*, 154122.

[32] (a) A. Hellweg, C. Hättig, S. Höfener, W. Klopper, *Theor. Chem. Acc.* **2007**, *117*, 587-597; (b) F. Weigend, *J. Comput. Chem.* **2008**, *29*, 167-175.

[33] A. V. Marenich, C. J. Cramer, D. G. Truhlar, *J. Phys. Chem. B* **2009**, *113*, 6378-6396.

[34] F. Neese, *Wiley Interdiscip. Rev.: Comput. Mol. Sci.* **2018**, *8*, e1327.

[35] M. J. Frisch, G. W. Trucks, H. B. Schlegel, G. E.Scuseria, M. A. Robb, J. R. Cheeseman, G. Scalmani, V. Barone, B. Mennucci, G. A. Petersson, H. Nakatsuji, M. Caricato, X. Li, H. P. Hratchian, A. F. Izmaylov, J. Bloino, G. Zheng, J. L. Sonnenberg, M. Hada, M. Ehara, K. Toyota, R. Fukuda, J. Hasegawa, M. Ishida, T. Nakajima, Y. Honda, O. Kitao, H. Nakai, T. Vreven, J. A. Montgomery, J. E. Peralta, F. Ogliaro, M. Bearpark, J. J. Heyd, E. Brothers, K. N.

Kudin, V. N. Staroverov, R. Kobayashi, J. Normand, K. Raghavachari, A. Rendell, J. C. Burant, S. S. Iyengar, J. Tomasi, M. Cossi, N. Rega, J. M. Millam, M. Klene, J. E. Knox, J. B. Cross, V. Bakken, C. Adamo, J. Jaramillo, R. Gomperts, R. E. Stratmann, O. Yazyev, A. J. Austin, R. Cammi, C. Pomelli, J. W. Ochterski, R. L. Martin, K. Morokuma, V. G. Zakrzewski, G. A. Voth, P. Salvador, J. J. Dannenberg, S. Dapprich, A. D. Daniels, Ö. Farkas, J. B. Foresman, J. V. Ortiz, J. Cioslowski, D. J. Fox, *Gaussian 16, Revision C.01, Gaussian, Inc., Wallingford CT, 2016*.
